# Using combined single-cell gene expression, TCR sequencing and cell surface protein barcoding to characterize and track CD4+ T cell clones from murine tissues

**DOI:** 10.3389/fimmu.2023.1241283

**Published:** 2023-10-12

**Authors:** Annekathrin Silvia Nedwed, Sara Salome Helbich, Kathrin Luise Braband, Michael Volkmar, Michael Delacher, Federico Marini

**Affiliations:** ^1^Institute of Medical Biostatistics, Epidemiology and Informatics (IMBEI), University Medical Center Mainz, Mainz, Germany; ^2^Institute of Immunology, University Medical Center Mainz, Mainz, Germany; ^3^Research Center for Immunotherapy, University Medical Center Mainz, Mainz, Germany; ^4^Helmholtz-Institute for Translational Oncology Mainz (HI-TRON Mainz), Mainz, Germany

**Keywords:** scRNA seq, scTCR seq, TCR (T-cell receptor), CD4 T cell, tissue CD4 T cell

## Abstract

Single-cell gene expression analysis using sequencing (scRNA-seq) has gained increased attention in the past decades for studying cellular transcriptional programs and their heterogeneity in an unbiased manner, and novel protocols allow the simultaneous measurement of gene expression, T-cell receptor clonality and cell surface protein expression. In this article, we describe the methods to isolate scRNA/TCR-seq-compatible CD4^+^ T cells from murine tissues, such as skin, spleen, and lymph nodes. We describe the processing of cells and quality control parameters during library preparation, protocols for multiplexing of samples, and strategies for sequencing. Moreover, we describe a step-by-step bioinformatic analysis pipeline from sequencing data generated using these protocols. This includes quality control, preprocessing of sequencing data and demultiplexing of individual samples. We perform quantification of gene expression and extraction of T-cell receptor alpha and beta chain sequences, followed by quality control and doublet detection, and methods for harmonization and integration of datasets. Next, we describe the identification of highly variable genes and dimensionality reduction, clustering and pseudotemporal ordering of data, and we demonstrate how to visualize the results with interactive and reproducible dashboards. We will combine different analytic R-based frameworks such as *Bioconductor* and *Seurat*, illustrating how these can be interoperable to optimally analyze scRNA/TCR-seq data of CD4^+^ T cells from murine tissues.

## Introduction

Single-cell sequencing-based technologies have significantly changed our view on cellular architecture and heterogeneity of samples (1–4). One particular example includes single-cell sequencing-based gene expression profiling (scRNA-seq) of individual cells (5, 6), which is based on the linear amplification of RNA derived from individual cells, followed by complex bioinformatic processing steps and identification of cell types in an unbiased way (7–9). Despite differences in technology and chemistry (benchmarked in (10)), single-cell sequencing experiments generally require four main steps (11).

First, tissues or organs have to be processed and digested to liberate target cells from the extracellular matrix in the tissue network. This yields a single-cell suspension where our target cells are present in varying frequencies, based on the tissue itself and its state, often dependent on the experimental conditions under investigation (Inflamed? Tumor-bearing? Virus-infected? Necrotic? Hypoxic)?. These steps have to be optimized to yield viable, intact cells without causing too much stress or hypoxic damage (12). While experimental procedures are now established for various cell and tissue types, no detailed workflow is available for tissue T cells, covering not only the wet-lab steps but also providing comprehensive guidance on the bioinformatic analyses for the datasets generated. In previous work, we have developed protocols for isolating T cells from a wide array of murine and human tissues such as skin, visceral adipose tissue, colon, lungs, liver, or different lymphoid tissues, and used them for downstream sequencing-based analysis (13–16). In the methods paper presented here, we will describe protocols to isolate target cells from murine skin and secondary lymphoid tissues such as spleen and lymph nodes (LN). To promote best data quality, we pre-enrich for viable, high-quality target cells using fluorescence-activated cell sorting (FACS) before performing single-cell barcoding. This allows the removal of unwanted cells, dead cells, dying cells, and cellular debris that might otherwise compromise quality. We will provide advice on cell sorting and sample multiplexing using barcoded antibodies.

In the second critical step, highly pure target cells are processed (“barcoded”) and genetic material is amplified. Single-cell isolation and library preparation can be based on several different technologies. This begins with limiting dilution technologies, magnetic cell sorting, micromanipulation using microscope-guided capillary pipettes or laser microdissection, sorting of single cells into a 96- or 384-well plate using FACS, to microfluidic systems that combine droplets and cells, and new technologies and adaptations are developed rapidly (12, 17). Importantly, all different technologies aim to capture a single cell in an isolated reaction volume to add a unique barcode specific for this cell.

In a third step, a sequencing library is prepared. In our case, we prepare not only one, but three libraries: a gene expression library that contains sequencing reads allowing to identify and quantify genes expressed on a cell-individual level (GEX library); a second library that contains quantitative information about cell surface protein expression and sample multiplexing (hashtag oligo) information (CSP library); and a library that contains the T-cell receptor usage information as nucleotide sequence (VDJ library). We will provide examples of all three libraries including PCR cycles, concentration, and electrophoresis-based size profiles.

The last step of the wet-lab procedure is the sequencing of all three libraries using high-throughput next-generation sequencing technology. At the end of the run, *FastQ* data are demultiplexed and copied from the sequencing instrument, and are now ready to undergo bioinformatic processing. In this methods paper, we provide an example dataset which we generated for this publication, where we applied the above-mentioned protocols to combine single-cell gene expression, TCR sequencing and cell surface protein barcoding to characterize and track CD4^+^ T-cell clones from murine tissues, and which can be downloaded by the reader for reproducing our bioinformatics workflow. The datasets include several thousand CD4^+^CD25^+^ Treg cells from murine spleen, mesenteric LN (mLN), inguinal LN (iLN) as well as CD3^+^ immune cells from skin, for all of which GEX, CSP and VDJ libraries have been generated and sequenced.

**Figure 1:**
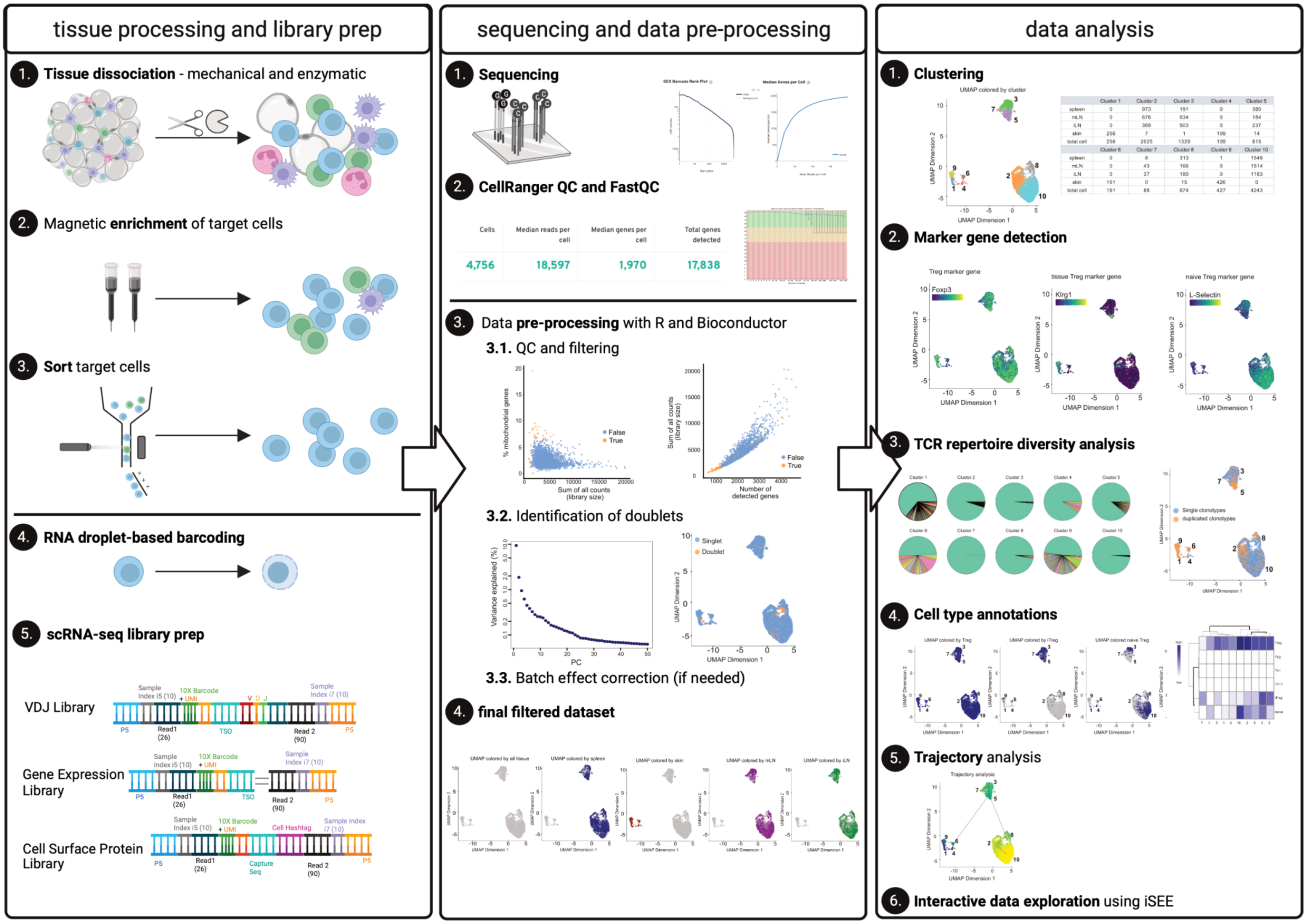
Graphical abstract. The left panel describes tissue processing and library prep: Tissues harvested from an individual mouse are enzymatically and mechanically digested (1) and material is magnetically enriched for target cells (2) to make cell sorting (3) more efficient. After obtaining a pure target population (3), cells (labelled with Biolegend TotalSeqC anti-mouse Hashtagging antibodies) and 10X beads are loaded on the 10X Chromium controller (4) followed by scRNA-seq library preparation (5). The middle panel describes sequencing (1) quality control using *CellRangerMulti* and *FastQC* (2). Using R and Bioconductor, data can be pre-processed. These steps include QC and filtering (3.1), the identification of doublets (3.2), and, if necessary, batch effect correction (3.3) to yield the final, filtered dataset (4). The right panel describes data analysis, comprising the clustering (1), marker gene detection (2) as well as TCR repertoire diversity analysis (3). Furthermore, cell type annotations (4) and trajectory analysis can be performed (5). Moreover, an interactive data exploration by using iSEE can be done (6). Elements of this figure have been created with Biorender using figures and plots generated in this manuscript.

Using this dataset, we will describe a step-by-step bioinformatic workflow to help repeat and reproduce the results achieved using the methods described in this paper. First, we apply *FastQC* and *CellRangerMulti* to enable a combined analysis of all individual samples and determine overall sequencing quality and identify individual cells. Here, we discuss critical quality-related parameters that *CellRanger* delivers, and discuss typical results obtained with CD4^+^ T cells from tissues. In a next step, we create the count matrix from *CellRangerMulti* output. We describe the pre-processing of scRNA-seq data using a variety of freely available R packages to perform quality control (QC) and filtering, dimensionality reduction, removal of doublets, evaluation of batch effect correction, and generating the final filtered dataset for analysis (following best practices outlined in (8) and (7)). We will also provide guidance on clustering, marker gene detection, cell type annotation, and interactive data exploration, accompanying this manuscript with a notebook containing all code and output from the analysis of our test dataset, which we refer to in the corresponding paragraphs. All essential steps for this end-to-end workflow are summarized in **Figure 1**.

## Methods – experimental procedures

### Isolation of T cells from murine spleen, mLN and iLN

To isolate T cells from murine secondary lymphoid tissues such as spleen or lymph nodes, a midline excision is performed to open the skin and abdominal wall, and forceps are used to expose the peritoneal cavity. The spleen is harvested immediately and stored at 4°C until use. To isolate mLNs, the cecum is located, the small intestine is moved to the side and the chain of mLNs are exposed. Using forceps, the tissue is harvested, placed in FACS buffer (**Table 1**) and stored at 4°C. Inguinal lymph nodes are collected from both hemispheres beneath the skin, placed in FACS buffer and stored at 4°C until use. To process the spleen, it is placed on a 100 µM filter unit and is mechanically dissociated using a plunger or forceps. Following centrifugation (2 min, 1000g, 4°C), red blood cells are lysed using a commercially available ACK lysis buffer (e.g., Thermo Fisher #A1049201). The cell suspension is filtered using a 70 µm strainer, resuspended in 500 µl FACS buffer, and cells are counted. To process LNs, the individual nodes are placed on a 100 µM filter unit and are mechanically dissociated using a plunger or forceps. Following centrifugation (2 min, 1000g, 4°C), the suspension is filtered using a 70 µm strainer, resuspended in 500 µl FACS buffer, and counted.

**Table 1:** Formulation for FACS buffer.

Ingredient	Manufacturer	Final concentration
Phosphate-buffer saline 10X	Gibco #10010023 or other	1X
FCS 100%	Sigma #F7524 or other	2%
Deionized water	NA	Up to final volume

Afterwards, we add Fc blocking reagent (Miltenyi Biotec #130-092-575) to prevent unspecific binding of antibodies and beads, followed by specific labeling using 1 µg PE-conjugated anti-mouse CD4 (Clone RM4-5, Biolegend #100512) or 1 µg PE-conjugated anti-mouse CD25 (Clone PC61, Biolegend # 102008) antibodies in 500 µl and stain for 20 min at 4°C. After staining, cells are centrifuged (2 min, 1000g, 4°C), washed using 1000 µl of FACS buffer, and resuspended in MACS buffer (**Table 2**). Next, target cells are bound by anti-PE ultrapure microbeads (Miltenyi Biotec #130-105-639) for 20 min at 4°C, followed again by two centrifugation (2 min, 1000g, 4°C) and washing steps using 1000 µl of FACS buffer. Finally, samples are re-suspended in 500 µl MACS buffer. A 70µl filter unit is placed on an equilibrated MACS column (we recommend working at 4°C to prevent cellular degradation) and the sample is loaded. The column is washed twice with 5 ml MACS buffer.

**Table 2:** Formulation for MACS buffer.

Ingredient	Manufacturer	Final concentration
Phosphate-buffer saline 10X	Gibco #10010023 or other	1X
Bovine Serum Albumin 100%	Sigma #A4503 or other	0,5% (w/v)
Ethylenediaminetetraacetic acid	ThermoFisher #15575020	1 mM
Deionized water	NA	Up to final volume

**Figure 2:**
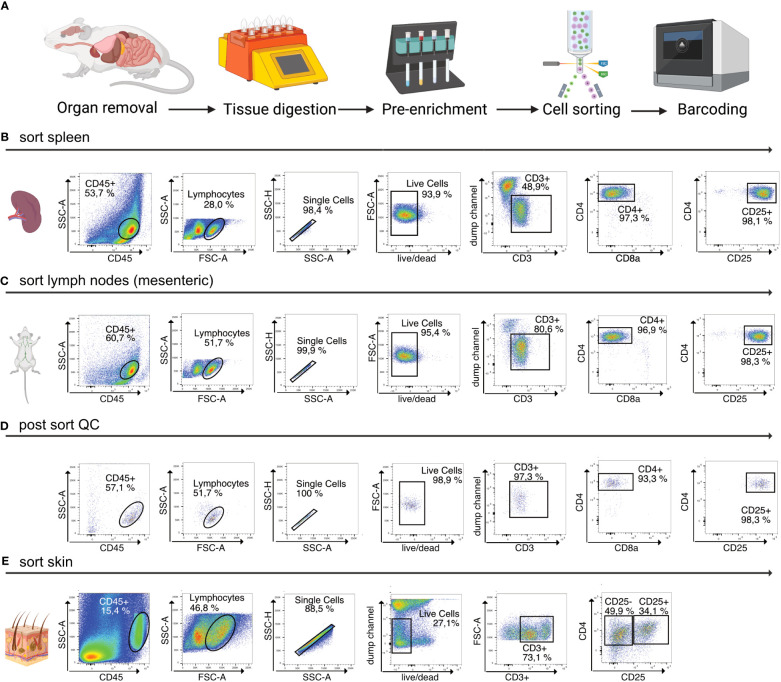
Overview of sample preparation for scRNA-seq of CD4^+^ T cells from murine tissues. **(A)** Procedural overview. Organs are removed, followed by tissue digestion and pre-enrichment for CD4^+^ T cells. These are then sorted, followed by single-cell barcoding using 10X Chromium controller. **(B, C)** Flow cytometry plots illustrating the gating scheme to isolate T cells from lymphoid tissues such as spleen and mLN. **(D)** Post sort QC of spleen, mLN, iLN CD25^+^ sorted into the same collection tube. **(E)** Flow cytometry plots illustrating the gating scheme to isolate T cells from murine skin tissue. Figure elements created with Biorender.

Afterwards, the sample is eluted in 500 µL FACS buffer and stained for 30 min at 4°C using fluorescence-labelled antibodies as well as TotalSeqC anti-mouse Hashtagging antibodies (Biolegend #*155861* (C1), #*155863* (C2), #*155865* (C3), #*155865* (C4)). To increase TotalSeqC antibody labeling, it is recommended to wash cells 3-5 times with 500 µL FACS buffer after staining. For sorting, cells can be resuspended in 200 µL MACS buffer. In order to prevent aggregates during the co-staining of fluorescence-labeled antibodies and Biolegend TotalSeqC antibodies, it’s recommended to centrifuge the antibody mix at 14,000 x g for 10 min at 4°C. Afterwards the supernatant should be transferred to a new tube and maintained at 4°C. The antibody aggregates will stay at the bottom of the original tube. For sorting, an example is shown in **Figure 2**. We recommend a gating strategy where CD4^+^ or CD25^+^ T cells are enriched to high purity using FACS, and dead cells, unwanted cell types and doublets are excluded. The target cells can be sorted into MACS buffer. A small part of the sorted population (target cells) can then be re-analyzed before downstream processing to determine post sort purity, viability, and cell recovery/sort efficiency. If the post sort QC indicates that cells are of good viability and purity (for troubleshooting see **Table 3**), the sample can be subjected to single-cell barcoding, as described later.

**Table 3:** Troubleshooting and Recommendations.

Description	Solution
All cells are dead	Analyze buffer ingredients, optimize erythrocyte lysis procedure
Erythrocyte contamination	Optimize ACK lysis procedure
Low purity of CD4^+^ or CD25^+^ T cells	Use Fc blocking reagent, work at 4°C

### Isolation of T cells from murine skin tissue

To isolate T cells from skin tissue, hair must be removed from the back of the animal with an electric shaver and depilatory cream. The cream is applied for 2 minutes, followed by vigorous washing using tap water to remove hair. It is important that excess hair is completely removed to avoid complications during downstream filtration steps. After cleaning, the skin is separated from the dorsal surface, cut into small pieces, and transferred to a GentleMACS tube (Miltenyi Biotec #130-096-334) containing 10ml of skin digestion buffer (**Table 4**). We recommend 10ml digestion buffer for 0.5 g of skin tissue.

**Table 4:** Formulation for skin digestion buffer.

Ingredient	Manufacturer	Final concentration
DMEM media	Gibco #41965	1X
Collagenase Type II	Sigma #C6885	4 mg/ml
Bovine Serum Albumin	Sigma #A4503	20 mg/ml
DNAse I	Roche #11284932001	20 µg/ml

Then, the sample is digested using the GentleMACS Dissociator (program: *37_C_Multi_H*) or via orbital shaking in a preheated waterbath (37°C). After 90 minutes of digestion or completion of the GentleMACS program, the single-cell suspension can be cut again, centrifuged (10 min, 400g, 4°C), resuspended in 5000 µl FACS buffer and transferred to a 15 ml tube through a 100 µm filter unit. Then, the sample is centrifuged again (2 min, 1000g, 4°C), resuspended in 1000 µl FACS buffer and filtered into a new 1.5 ml tube using a 70 µm filter unit. The sample can now be stained for 30 min at 4°C using fluorescence-labelled antibodies as well as Biolegend TotalSeqC anti-mouse Hashtagging antibodies, as described before. For sorting, cells can be resuspended in 200 µL MACS buffer. An example of the sorting strategy of T cells from murine skin tissue is shown in **Figure 2E** To increase efficiency, it is beneficial to first enrich for CD45^+^ immune cells (yield sort) by sorting target cells into MACS buffer, followed by a second purity sorting (4-way purity sort) of target cells (**Table 5**).

**Table 5:** Troubleshooting and Recommendations.

Description	Solution
Clogging caused by hair	Additional filter steps after skin digestion get rid of hair and avoid clogging. Repeat hair removal if patches of hair remain.
Clogging during cell sorting	For cell sorting, samples should be filtered again immediately before acquisition and cooled at 4°C to avoid clogging.
Poor cell recovery after sorting	Use a two-step sorting protocol with a pre-sort (“yield”) and a high purity sort (sort strategy “4-way-purity”) mode.
Low expression of CD4^+^ on T cells	Optimize processing time and amount of collagenase enzymes.

### Single droplet barcoding of T cells for combined scRNA/TCR-seq

**Figure 3:**
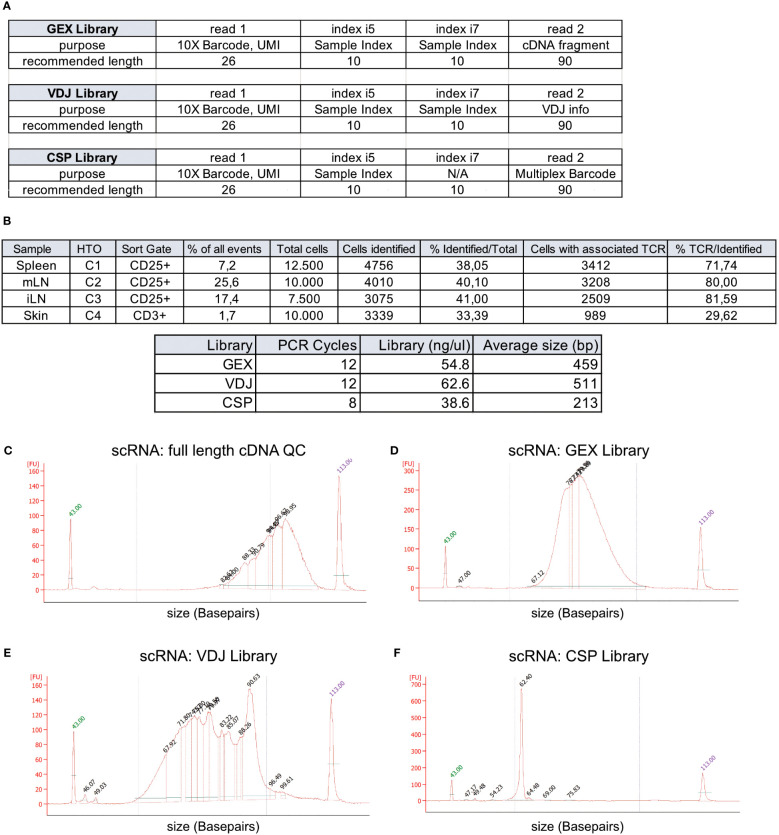
Overview of recovery and typical profiles for scRNA-seq libraries. **(A)** Overview of GEX, VDJ and CSP library and recommended sequencing length (source: 10X Genomics). **(B)** Tabular overview of parameters in scRNA-seq experiments. The percentage of all events indicates the total frequency of target cells (either CD4^+^ or CD25^+^ T cells) in all events from the sample. **(C-F)** Examples for library size profiles for samples with a good library profile listed in **(A)** for either **(C)** full length cDNA, **(D)** GEX Library, **(E)** VDJ Library or **(F)** Cell Surface Protein (CSP) library. Electrophoretic separation was performed on a Bioanalyzer.

Target cells from spleen (12,500 CD3^+^CD4^+^CD25^+^ Treg cells, TotalSeqC1), mLN (10,000 CD3^+^CD4^+^CD25^+^ Treg cells, TotalSeqC2), iLN (7,500 CD3^+^CD4^+^CD25^+^ Treg cells, TotalSeqC3) and skin (10,000 CD3^+^ T cells, TotalSeqC4) have all been sorted into a single 1.5 mL Eppendorf tube containing 350 μL MACS buffer, and the sample collection tube was cooled to 4°C. It is important to process the sample quickly after sorting to decrease the number of dying/dead cells in the collection tube. Therefore, shortly after sorting, cells are pelleted by centrifugation (5 min, 300 xg, 4°C). Supernatant is removed and the sample is supplemented with master mix and beads to a final volume of 70 μL, loaded on a 10X Chromium Next GEM Chip K (10X Genomics #1000287) and processed on the 10X Chromium Controller (10X Genomics #120212), followed by cDNA amplification using the Chromium Next GEM Single Cell 5’ Reagent Kit v2 (10X Genomics #1000263) and 5’ Feature Barcode Kit (10X Genomics #1000256). Afterwards V(D)J amplification was done from cDNA by using the Chromium Single Cell Mouse TCR Amplification Kit (10X Genomics #1000254) and GEX, CSP and VDJ library preparation according to the Library Construction protocol (10X Genomics #1000190). In **Figure 3A**, we show the elements of each library, including the sample indexes i5 and i7, read1 and read2 with their purpose and recommended sequencing length. In **Figure 3B**, cycle numbers and typical library sizes are shown. Upon completion of cDNA amplification and library preparation, the fragment length composition is usually evaluated using electrophoretic separation of the sample, for which we show examples in **Figures 3C–F**.

## Methods – sequencing and QC strategy for scRNA-seq libraries

### Next-generation sequencing of GEX, VDJ and CSP libraries

In **Figure 3B**, we listed the total number tagged and sorted cells and the total number of cells identified after sequencing. The recovery rates were 38.0% for spleen CD25^+^ Treg cells, 40.1% for mLN CD25^+^ Treg cells, 41.0% for iLN CD25^+^ Treg cells, and 33.4% for skin CD4^+^ T cells, with a mean recovery rate of 38.13%. Peripheral tissues that undergo enzymatic digestion, such as skin, liver, lung, or colon tissue, have varying recovery rates based on cell preparation steps, pre-enrichment, duration of processing, sort efficiency and sort setup. This can sometimes lead to recovery rates below 10% and requires optimization. Usually, all samples are sequenced in “one batch”, and varying recovery rates can lead to “under- or over-sequencing” of libraries. Therefore, we recommend performing a pre-sequencing using only the gene expression (GEX) library. This reduces the cost for sequencing, allows for the identification and removal of low-quality and degraded samples, and increases the overall comparability of the datasets due to harmonized sequencing depth. Here, using a rough estimate of a projected cell number recovery (in our case, we estimate about 40% of sorted cells to be recovered later for bioinformatic analysis) helps to estimate the total number of reads required to sequence the GEX library to the desired depth. Now, for pre-sequencing, we only run 5%-10% of the estimated required reads to determine the approximate cell number for each library. These values are then used to sequence all libraries with a rather precise estimate of the required numbers of reads per library. In our lab, we routinely sequence 10X 5’ scRNA-seq libraries using a paired-end run with 26-10-10-90 sequencing strategy with a 150-cycle high-output cartridge on a NextSeq 500/550 sequencing unit. In a typical run, read 1 identifies the i5 index (cell barcode) with 10 nucleotides and reads 26 nucleotides of 10X Barcode and UMI. On the reverse strand (read 2), primer P7 initiates the i7 read (sample index) with 10 nucleotides and reads 90 nucleotides of the cDNA (**Figure 3A**. The remaining 90 reads of read 2 are important for calling the gene (*GEX library*), the cell surface protein and/or hashtag oligo (e.g. TotalseqC), which appears at a fixed position (10th base) in read 2 (*CSP library*) or the VDJ information for the TCR (*VDJ library*). For the samples available as open access download alongside this paper, we used a 300-cycle high-output cartridge with a paired-end run and 26-10-10-149 sequencing strategy. In **Figures 3C–F** examples for library profiles from full length DNA (**c**), GEX (**d**), VDJ (**e**) and CSP (**f**) of a sample containing CD25^+^ cells from spleen, mLN and iLN as well as CD3 skin T cells is shown. Since we used hashtag oligos (TotalseqC1-4) and pooled the different organs into one sample during sort, we only get one cDNA, GEX, VDJ and CSP library for all 4 samples.

### Investigating sequencing quality using *FastQC*

**Figure 4:**
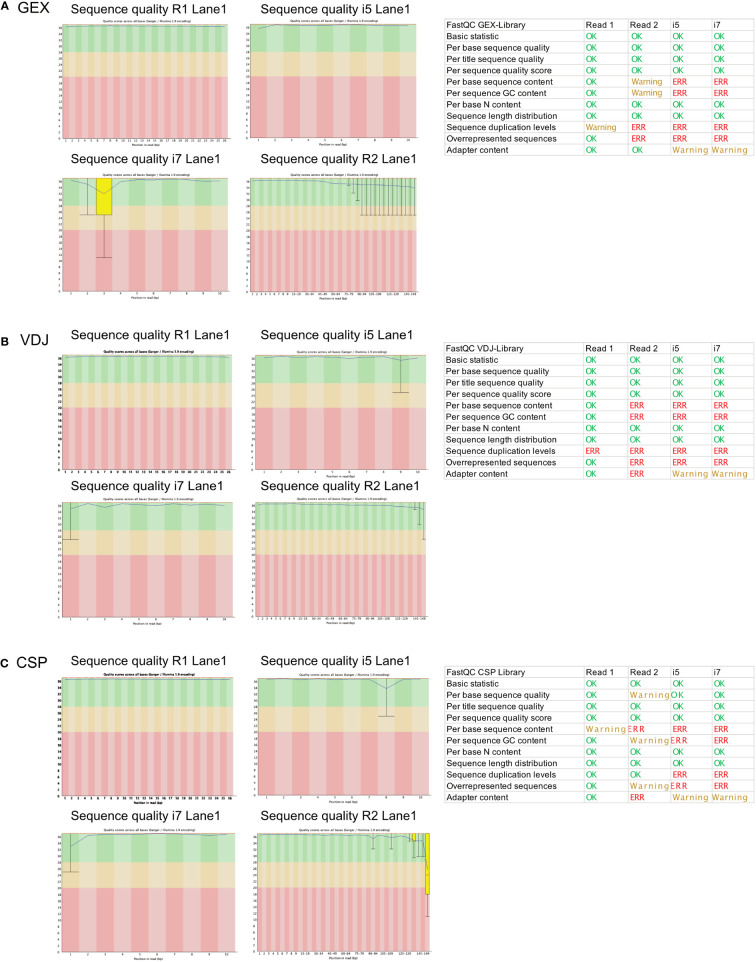
*FastQC* report of the GEX Library, VDJ Library and CSP Library. Statistics of *FastQC* run for the GEX library **(A)**, VDJ library **(B)** and CSP library **(C)** on for read 1 (26 bp), read 2 (149 bp), i5 (10 bp) and i7 (10 bp). Errors and Warnings listed here as reported in *FastQC* documentation. Produced by *FastQC* (version 0.11.9).

To investigate whether we can estimate library quality, we ran FastQC on all L001 files generated from the different libraries. A plot labeled “per base sequence quality” shows the distribution of quality scores at each position in the read across all reads (**Figure 4A**). It can alert to whether there were any problems during sequencing. As the read 2 contains the information for the gene expression, we focus on this read in our analysis. Warnings related to “per base sequence content” are common for RNA-seq data and can be safely ignored in most cases. Also, warnings related to “per sequence GC content” has already been observed in literature (18) and can be ignored according to the manufacturer’s guidelines. The “sequence duplication level” and “overrepresented sequences” error can indicate a low complexity library which could result from too many cycles of PCR amplification or less cDNA concentration before preparing the library. In this data set, we see a low contamination of a known primer sequence. If this contaminating sequence would be very high, it might be useful to get rid of it before downstream analysis. As shown in the schematic overview (**Figure 3A**), the VDJ and CSP Library are very different from the GEX Library because they contain VDJ information and very few cell surface protein barcode sequences. FastQC is not tailored for analysis of such low-complexity libraries, but we included the results for reference (**Figures 4B, C**).

## Methods – use of *CellRanger* to identify cells and investigate quality and quantity

In the previous sections, we described detailed protocols to isolate CD4^+^ T cell populations from murine tissues such as spleen, LN or skin. Next, we provided advice on cell sorting and sample multiplexing using hashtag oligos (e.g. TotalSeqC), followed by single droplet barcoding and library preparation steps using 5’ reagent kits. Sequencing of our three individual libraries (GEX, CSP and VDJ) will generate *FastQ* files ready for analysis using *CellRanger*, a software tool developed for single-cell sequencing-based datasets generated with chemistry from 10X Genomics. In the following paragraphs, we will describe the use of *CellRangerMulti* to extract individual samples and generate output files that allow a first glimpse on data quality and quantity.

### Use of *CellRangerMulti* to enable a combined analysis of all individual samples

**Figure 5:**
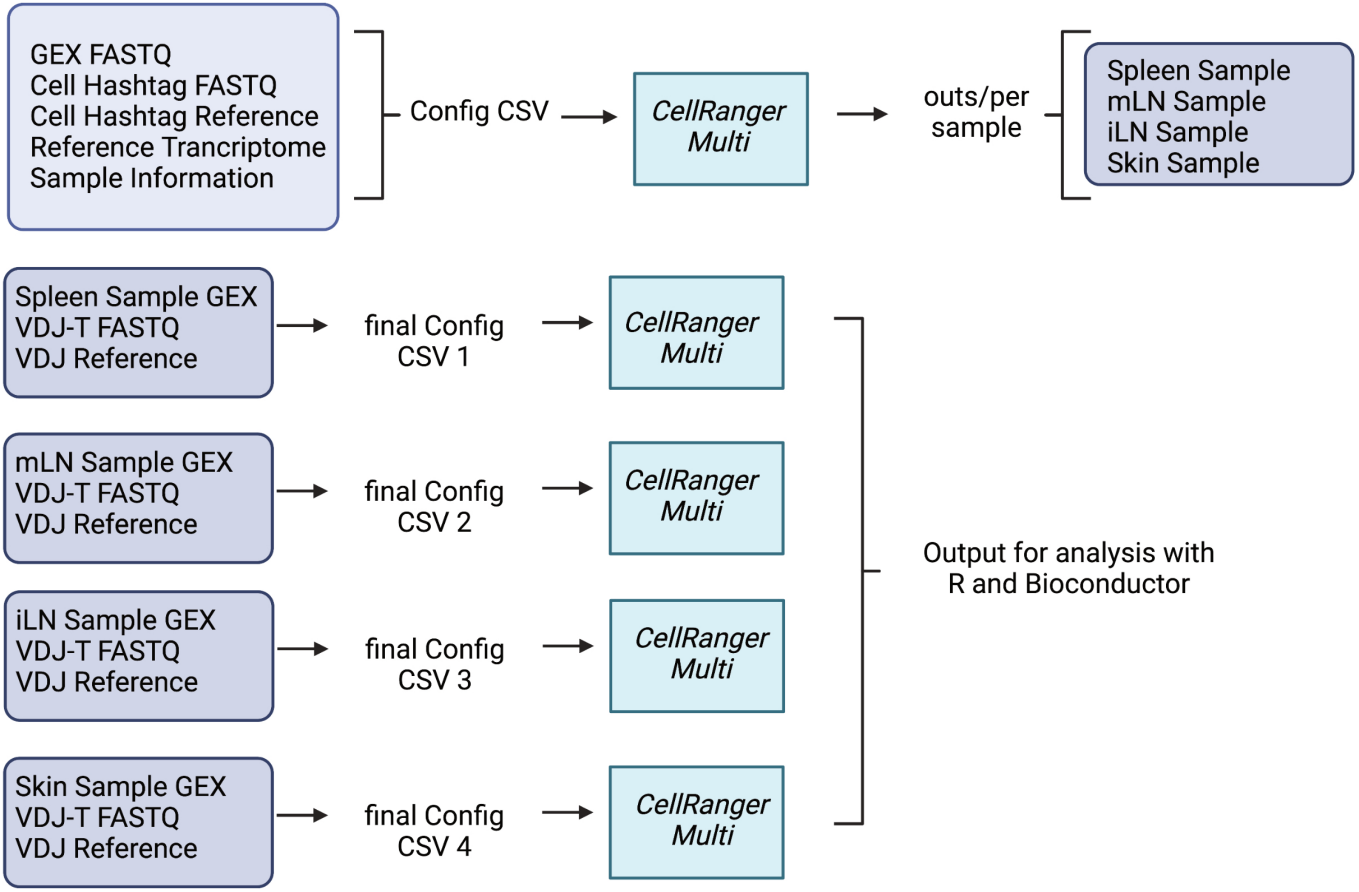
Schematic Overview of the *CellRangerMulti* Pipeline for combining 5’ Single Cell Gene Expression Analysis with Cell Hashtag and VDJ T Cell Analysis. The 5’ Chromium Next GEM Single Cell Immune Profiling cell hashing assay workflow starts with a demultiplexing step to assign pooled cells to individual samples (=hashtags). Afterwards, *CellRangerMulti* can be used to analyze individual samples and combine TCR with the GEX data. Created with Biorender.

*CellRangerMulti* is a method for the combined processing scRNA samples by the use of specific multiplexing antibodies and officially supports the analysis of 3’ multiplexed data. The 3’ and 5’ assays capture different ends of the transcript in the final library, and we used the 5’ chemistry to generate GEX, CSP and VDJ libraries. Therefore, this type of analysis requires editing of the *CellRangerMulti* pipeline to be compatible with our datasets. Our pooled libraries contain four samples: splenic Treg cells (TotalSeqC1), mLN Treg cells (TotalSeqC2), iLN Treg cells (TotalSeqC3) and skin CD3^+^ T cells (TotalSeqC4). In the first demultiplexing step, we use *CellRangerMulti* to assign cells to individual samples, a workflow described in **Figure 5**. First, we need to create a library comma-separated values (CSV) file which declares the input FASTQ data for the libraries that make up a cell multiplexing experiment (**Box 1**). Second, we need to create a cell hashtag reference. It declares the molecule structure and unique cell hashtag sequence of each hashtag (=TotalSeq) antibody present in the experiment. Each line of the CSV declares one unique cell hashtag.

The *CellRangerMulti* pipeline first extracts and corrects the cell barcode and UMI from the CSP library using the same methods as gene expression read processing. It then matches the cell hashtag read against the list of features declared in the cell hashtag reference. This is all described in specific sections of the config CSV file which requires the column [gene expression], [libraries] and [samples]. The [gene expression] section specifies the path to the reference transcriptome and the cell hashtag reference. The [libraries] section shows the path to the GEX FASTQs (GEX library) and cell multiplexing FASTQs (CSP library). The [sample] section includes a list of all samples and the corresponding hashtag. After creating these files, we run *CellRangerMulti* and assign cells to samples. By doing so, we also create BAM files of the individual samples in the pool. Those files are located in the individual directories for each sample. Since *CellRangerMulti* requires FASTQ files as the input, we convert the BAM files to individual FASTQ files. This can be done with the *bamtofastq* software tool which is bundled with *CellRanger*. The output of *bamtofastq* will display two directories per sample. After using *samtools*, which is also a part of the *CellRanger* bundle, we can distinguish the gene expression FASTQ from the cell hashtag FASTQ. In a final step, the T-cell receptor library can now be combined with the gene expression data. To do so, we run the *CellRangerMulti* again for every individual sample. We create a new final config CSV file for every individual sample and include the [vdj] section which describes the path to a VDJ reference. Each run produces output files which can then be used for further analysis with R.

BOX 1 **Terminal input to run *CellRangerMulti* and assign cells.**

**Table:** 

Info: In this manuscript, commands to be entered in the terminal are prepended by the “$” symbol.
# run multi pipeline (combine GEX Library with Cell Surface Library) $ cellranger multi\ –id=ddmmyy_multi\ –csv=./configmulti.csv # configmulti.csv $ cat configmulti.csv [gene-expression] reference,/*path_to*/refdata-gex-mm10-2020-A Cmo-set,/*path_to*/cmo-set.csv force-cells, check-library-compatibility,false [libraries] fastq_id,fastqs,feature_types [samples] sample_id,cmo_ids spleen,HTO_C0301 mLN,HTO_C0302 iLN, HTO_C0303 skin,HTO_C0304 # cmo-set.csv $ cat cmo-set.csv id,name,read,pattern,sequence,feature_type C1,HTO_C0301,R2,5PNNNNNNNNNN(BC)NNNNNNNNN,ACCCACCAGTAAGAC,Antibody Capture C2,HTO_C0302,R2,5PNNNNNNNNNN(BC)NNNNNNNNN,GGTCGAGAGCATTCA,Antibody Capture C3,HTO_C0303,R2,5PNNNNNNNNNN(BC)NNNNNNNNN,CTTGCCGCATGTCAT,Antibody Capture C4,HTO_C0304,R2,5PNNNNNNNNNN(BC)NNNNNNNNN,AAAGCATTCTTCACG,Antibody Capture # Command to change to the directory where the CellRanger executable file lives and put it in your $PATH: $ export PATH=/*path_to*/cellranger-7.0.1:$PATH $ export PATH=${PWD}:$PATH # Command to put other tools bundled with CellRanger in your path: $ source/*path_to*/cellranger-7.0.1/sourceme.bash # Make a new directory mkdir bamtofastq # Run bamtofastq # You will need the path to the individual sample_alignments.bam. In addition, 10X recommends setting the # -–reads-per-fastq= argument higher than the total number of reads recorded. bamtofastq –-reads-per-fastq=2200000000/*path_to*/sample_alignments.bam/*path_to_outputfolder*/bamtofastq/*name_of_new_folder* # after bam to fastq, identify the FASTQ directory corresponding to GEX: cd/*path_to_outputfolder*/bamtofastq/*name_of_new_folder * ls –ltsh # Use samtools to identify the GEX file source/*path_to*/cellranger-7.0.1/sourceme.bash samtools view -H/*path_to* sample_alignments.bam # Look for the @CO library info in the bottom # Run CellRangerMulti final analysis again for each sample (include VDJ Library) cellranger multi\ –id=ddmmyy_multifinal_organ1\ –csv=./configmultifinal.csv # display the content of configmultifinal.csv $ cat configmultifinal.csv [gene-expression] reference,*/path_to*/refdata-gex-mm10-2020-A force-cells,5000 check-library-compatibility,false [vdj] reference,/*path_to*/refdata-cellranger-vdj-GRCm38-alts-ensembl-7.0.0 [libraries] fastq_id,fastqs,feature_types bamtofastq,/*path_to_FASTQ*/, Gene Expression VDJ_FASTQ,/*path_to_FASTQ*/, VDJ

### Using metrics provided by *CellRanger* to evaluate quality and quantity of cells

In addition to creating outputs files which can be used for further analysis with R, *CellRanger* produces a web summary file in the output folder of the specified analysis directory. It is a good starting point for determining sample quality and quantity before starting with the analysis using R (as described in the next paragraphs in detail). Also, web summaries can be used to determine sample complexity and sequencing need (e.g. how many reads are still required per sample to have good coverage and even sequencing depth distribution between all samples).

**Figure 6:**
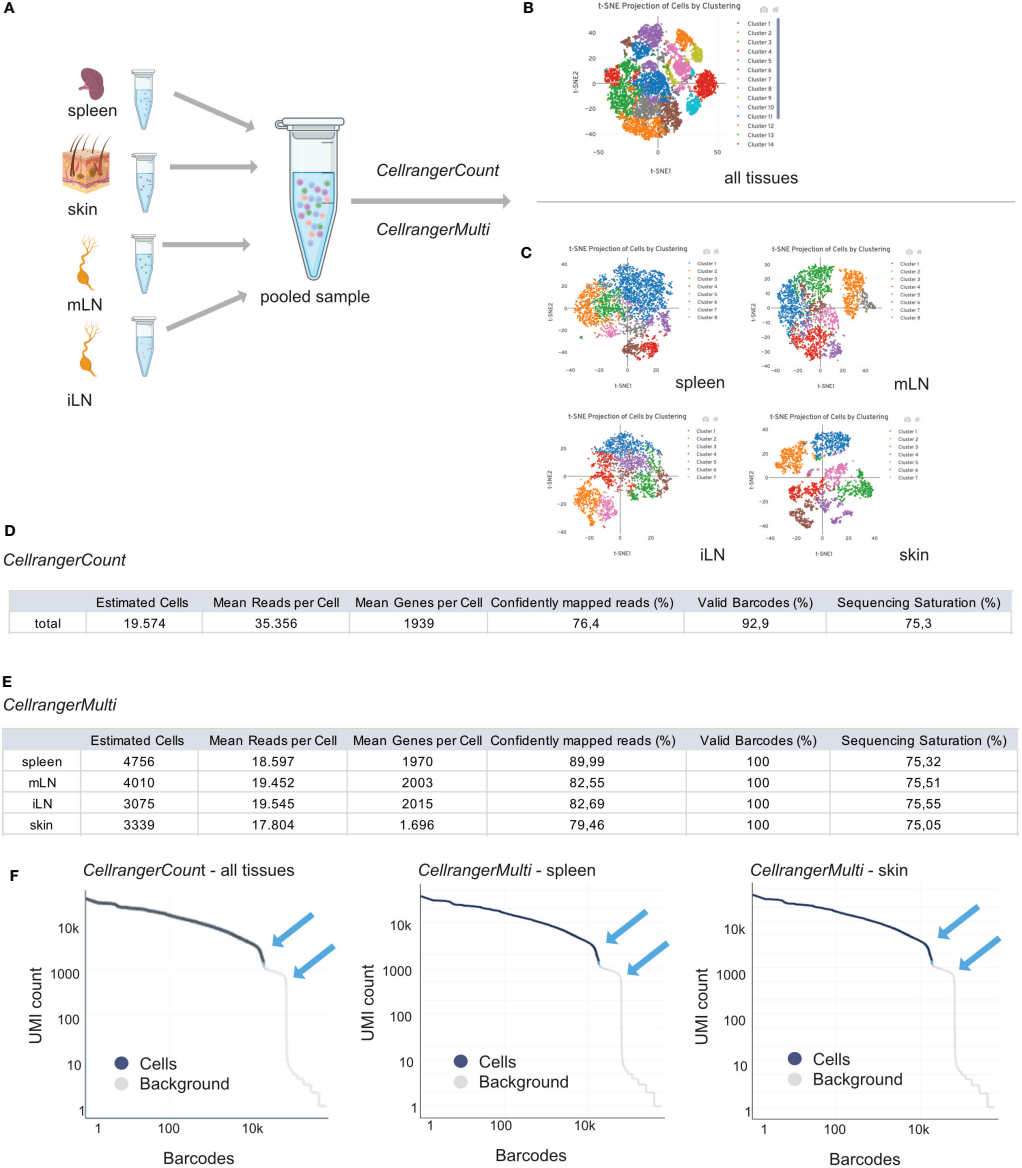
Interpretation of *CellRangerCount* and *CellRangerMulti* Output. Schematic overview of the experimental design **(A)** and *CellRangerCount* **(B)** and *CellRangerMulti* **(C)** output. Metric summaries for the *CellRangerCount* **(D)** and *CellRangerMulti* **(E)** and Rank Barcode plots **(F)** for all tissues, spleen and skin. Figure elements created with BioRender.

Therefore, *CellRanger* is a useful tool for investigating important sample parameters on a first glimpse. In general, we need to distinguish between an output from *CellRangerCount* and *CellRangerMulti*. When performing single cell RNA experiments, it can be useful to first run *CellRangerCount*. This pipeline aligns sequencing reads from the FASTQ files to a reference transcriptome. Then, different filtering steps, barcode counting, and UMI counting allow to determine clusters and perform gene expression analysis. To discriminate *CellRanger count* from *CellRangerMulti*, outputs are shown in **Figure 6A**. The t-SNE plot derived from *CellRangerCount* (**Figure 6B**) gives an overview of the heterogeneity of the sample, which, in our case, contains cells from the different lymphoid and peripheral organs (spleen, mLN, iLN, skin). However, *CellRangerCount* cannot assign cells to the organ of origin, since multiplexing info from the CSP library is not processed. The cells in the t-SNE plot are colored by cluster and show cell-associated barcodes. The clustering analysis is based on grouping cells with similar gene expression profiles and allows a first glimpse of data complexity and quality. In our case, with CD25^+^ or CD4^+^ T cells from the different lymphoid and peripheral organs (spleen, mLN, iLN, skin), *CellRanger*C*ount* generates a t-SNE with many different clusters, not too surprising because it counts all cells from the different organs (**Figure 6B**). In contrast to *CellRanger*C*ount*, *CellRangerMulti* can break down individual samples (= organs) using the hashtag oligo information of the CSP Library. The t-SNE after running *CellRangerMulti* shows less heterogeneity for the Treg cell populations in spleen, mLN and iLN, as expected with a very defined cell type (**Figure 6C**). Within the lymphoid organs, the clustering is more compressed because we enriched and sorted for CD25^+^ Treg cells for this dataset. In contrast to this, the clustering of the skin sample looks more heterogenous because it contains a larger subset of cells. If a complete lack of cluster structure appears in a usually rather heterogenous sample, this could indicate low sample quality or loss of single-cell behavior due to massive overloading or system failures.

In a table, we listed some of the web summary metrics which are shown when running *CellRangerCount* (**Figure 6D**) and *CellRangerMulti* (**Figure 6E**) on our sample dataset. *CellRanger* estimates the number of cells which are defined as the number of barcodes associated with at least one cell. As listed in **Figure 3**, using the protocols described in this paper, we should recover around 40% of original cell input as cells that are identified using *CellRanger*. However, a difference between the number of cells when running *CellRangerCount* compared to *CellRangerMulti* appears, which can be explained by the fact that we generally do not achieve 100% binding of the hashtag antibodies (= TotalSeqC barcodes) to the cells. Another important parameter displayed by *CellRanger* is the median reads per cell, which accounts for the total number of sequenced reads divided by the number of barcodes associated with cell-containing partitions. This information is helpful for planning a re-sequencing of the samples if not enough reads have been acquired, so that the recommended minimum of 20.000 reads/cell can be achieved. Another metric, median genes per cell, defines the median number of genes detected per cell-associated barcode. It also depends on sequencing depth and the total number of cells, and a low number of genes per cell can indicate low sequencing depth, low library quality or low transcriptional diversity of the cells. Another parameter linked to sample quality is the fraction of reads mapped confidently to the reference transcriptome. In our dataset, the lowest fraction of reads mapped to the murine genome is observed for the skin sample (79.46%), which, however, still is well above the lower threshold of 30% given by the manufacturer. Another quality-related parameter is the fraction of valid barcodes matching a whitelist. A value lower than 75% may indicate sequencing issues such as low quality of read 1. Finally, *CellRanger* computes sequencing saturation, which is an indicator of library complexity and sequencing depth. Lower sequencing saturation indicates that much of the library complexity was not captured by sequencing and that re-sequencing the sample could potentially increase gene expression coverage.

The *CellRanger* output files also contain a barcode rank plot where all barcodes detected during sequencing are plotted in decreasing order of UMIs associated with the particular barcode (**Figure 6F**). The shown barcode rank plot originates from the *CellRangerCount* (all tissues) and *CellRangerMulti* (spleen, skin) output. *CellRanger* uses the number of UMIs detected in each gel bead in emulsion (GEM) to determine whether the GEM contains a cell (declared as a cell) or not (declared as background). In a typical sample, a steep drop-off can be found and indicates good separation between the cell-associated barcodes and the barcodes associated with an empty GEMs. As mentioned in manufacturer’s guidelines, every barcode plank plot has a distinctive shape with steep drop-offs indicated by blue arrows (**Figure 6F**). In a very heterogenous sample, the plot can appear bimodal, but a clear separation between the cells and background should always be present. If the separation is not good and the barcode rank plot shows a round curved shape, this may indicate low sample quality or loss of single-cell behavior due to technical failures.

## Methods – data processing with R, Bioconductor and Seurat

In the previous paragraph, we discussed the use of *CellRanger* to produce output files which can then be used for further analysis with R. Now, we describe the pre-processing of scRNA-seq data using a variety of openly available R packages, which can be found on CRAN (https://www.R-project.org/) and Bioconductor (8). The pre-processing steps include quality control (QC) and filtering, dimensionality reduction, removal of doublets, evaluation of batch effect correction, which generates the final filtered dataset for analysis. For data pre-processing and analysis, we provide a rendered notebook file containing all code and output from the analysis of our test dataset in the supplement, which we refer to in the corresponding paragraphs (**Supplementary Material** or downloadable from https://github.com/imbeimainz/scRNAseq_scTCRseq_TissueTcells). In this manuscript, we will mainly discuss the analysis of the data using packages available on Bioconductor. However, the notebook will also provide the code for a pipeline using the Seurat package (19) and discuss the features of this pipeline.

### Creating the count matrix from *CellRangerMulti* output

scRNA-seq data analysis is performed on a count matrix, containing the counts (i.e. number of UMI or reads) per gene in each cell. scRNA-seq data is usually very sparse due to several factors such as dropout events, low mRNA abundance in the cells, and a combination of biological and technical variation (20, 21). In our workflow, the count matrix is constructed from the feature-barcode matrix information generated by *CellRangerMulti*. For scRNA-seq data, *CellRanger* provides an unfiltered feature-barcode matrix and a filtered feature-barcode matrix. The unfiltered feature-barcode matrix contains every barcode from a fixed list of known barcodes that have at least one count. These can contain background and cell-associated barcodes. The filtered feature-barcode matrix, however, only includes detected cell-associated barcodes. In our experience, unfiltered data contain a lot of cellular debris and background noise. However, if desired by the user, there are also R packages like *DropletUtils* (22) which provide methods to process the unfiltered count matrix to remove the unwanted noise. In our workflow, we will present the approach working on the filtered data and refer users to the *DropletUtils* documentation on how to work with unfiltered data. We use the *Read10X()* function from the *Seurat* package (19) to read in the filtered feature-barcode matrix information (**Box 2**, **Table 6**). This function returns a sparse matrix which stores the count information with genes as rows and samples as columns. We further process the resulting count matrix using the *SingleCellExperiment()* constructor from the *SingleCellExperiment* package (8). We repeat this process for all samples in the experiment. In addition to the counts, we store the tissue of origin for each sample as metadata in the respective *SingleCellExperiment* object. This information will be essential for some of the presented downstream analyses steps, especially for compelling and informative data visualizations. See section “1 Create SingleCellExperiment” in the notebook for the respective code of this analysis.

**Table 6:** Troubleshooting and Recommendations.

Description	Solution
Directory provided does not exist	It seems like the directory stated does not exist or is not found. Check if the directory location is spelled correctly and directory hierarchy matches the current working location.
filtered feature-barcode matrix folder does not contain features.tsv file	In the used version of CellRanger (v. 7.1) the features.tsv files is called genes.tsv. Please use this file as features file. Please note that you have to rename the file to features.tsv as the Read10X()expects this filename.
… file not found	The Read10X() function is rather stringent concerning filenames (at least as of v. 4.3.0) and expects the files to be named *matrix.mtx, barcode.tsv* and *features.tsv.* If the files have any other name (e.g. a sample prefix), the function will not find the files. Please rename the files following the mentioned naming convention.

BOX 2 **R code for creating SingleCellExperiment objects.**

**Table:** 

# Function to read in the data # provide all the filepaths to the count data as a list # as well as a list of the respective tissues readDataset <- function(filepath_list, tissue) { sceRNA <- list() # iterate over each sample of the input data for (i in 1:length(filepath_list)) { # read the count data counts <- Read10X(filepath_list[[i]]) # generate a SingleCellExperiment object sce = SingleCellExperiment(assays = list(counts = counts)) # Add the tissue type information as meta data sce$tissue <- rep(tissue[[i]], ncol(sce)) sceRNA <- c(sceRNA, sce) } # return the list of SingleCellExperiment objects return(sceRNA) } # input data is stored in a folder called data filepaths <- c(“./data/iLN”, “./data/mLN”, “./data/skin”, “./data/spleen”) sceRNA <- readDataset(filepaths, tissue = c(“iLN”, “mLN”, “skin”, “spleen”)) # set the names of the objects in the list so that we can easily identify and # access the different tissues names(sceRNA) <- c(“iLN”, “mLN”, “skin”, “spleen”) # Now have a look at the data sceRNA

### Gene level annotation

In a processing step before data analysis, we perform a gene-level annotation based on the input data (**Box 3**, **Table 7**). This gene-level annotation is used to facilitate the downstream applied analysis steps. During the annotation, the gene identifiers of the input data are mapped to their respective gene name using the *AnnotationHub* package (23). Gene names are usually more widely used and discernible and hence facilitate many of the downstream analysis steps, such as marker gene detection and cluster marker identification. Besides the annotation of gene names, we also determine which genes of the input data map to the mitochondrial portion of the genome as this is later used for filtering and quality control. See section “2 Gene level annotation” in the notebook for the respective code of this step.

**Table 7:** Troubleshooting and Recommendations.

Description	Solution
No genes map to the mitochondrial genome	It could be that the pattern used to search for mitochondrial genes does not match the pattern of mitochondrial genes in the data. Please check that these two patterns are identical (usually follow the lines of ‘MT’, ‘mt’, ‘Mt’ or ‘chrM’).
No gene names found/all gene names are ‘NA’	It could be that the species you are using for the annotation does not match your data. Please check that the correct species is specified. Another reason could be that the wrong id type was specified. Please check that the id type matches your gene ids.

BOX 3 **R code for gene level annotation.**

**Table:** 

sce <- sceRNA$iLN # set up the annotation hub ah <- AnnotationHub() # extract the indentifiers and names for mouse data query(ah, c(“musculus”, “Ensembl”, “EnsDb”)) ens.mm.v102 <- ah[[“AH89211”]] genes(ens.mm.v102)[, 2] # search for the mitochondrial genes is.mito <- grepl(“^mt-”, rownames(sce)) chr.loc <- mapIds( ens.mm.v102, keys = rownames(sce), keytype = “GENENAME”, column = “SEQNAME” ) is.mito <- which(chr.loc == “MT”) is.mito

### Extracting T cells from the data using linked TCR information

Before we apply quality control procedures to our data, we would like to filter our dataset for T cells with productive TCR chain information. For this, we have to use the information of the T-cell receptor (TCR) stored in the VDJ library. Only cells with TCR information will be kept in our data. In order to filter our data set for T cells, we add the information on the TCR chains and the clonotype of each cell to our *SingleCellExperiment* objects (**Box 4**). In our specific workflow, we also must transform the clonotypes as we have processed each sample individually using *CellRanger*. In order to work with shared clonotypes between tissues, we first apply a transformation step to assign identical TCR chains the same clonotype id (**Box 5**). Afterwards, we save the harmonized TCR chain and clonotype information as meta data in our *SingleCellExperiment* objects. We also provide a list of the transformed TCR chain and clonotype information with the data of this manuscript for follow-up. If the information of the TCR is not available, but an analysis of solely T cells is desired, users can follow this presented workflow up until the cell type annotation step. After this step, the data can be filtered for cells which were annotated as T cells and the workflow can be repeated from the beginning. For more information, please see section “3 Extracting T cells using T chain receptor information” in the notebook.

BOX 4 **R code for extraction of T Cells using TCRs.**

**Table:** 

addTCRMetaData <- function(sce, tcr_filepath, clonotypes_filepath) { # Read in the information about the TCRs tcr <- read.csv(tcr_filepath) clonotypes <- read.csv(clonotypes_filepath) # Remove duplicated barcodes as the information is identical. tcr <- tcr[!duplicated(tcr$barcode)], # Subset to only barcode and raw clonotype column as we only use those. tcr <- tcr[, c(“barcode”, “raw_clonotype_id”)] # Rename column to match to the clonotypes file names(tcr)[names(tcr) == “raw_clonotype_id”] <- “clonotype_id” # Extract the TCR chain information from the clonotypes file through matching # of the clonotypes. tcr <- merge(tcr, clonotypes[, c(“clonotype_id”, “cdr3s_aa”)]) # Reorder columns, set barcodes as rownames (to match the scRNA data) # and remove the barcode column as it is no longer necessary. tcr <- tcr[, c(2, 1, 3)] rownames(tcr) <- tcr[, 1] tcr[, 1] <- NULL # Add the TCR chain and clonotype information as metadata to the data clonotype <- tcr$clonotype_id[match(colnames(sce), rownames(tcr))] sce$clonotype <- clonotype cdr3s_aa <- tcr$cdr3s_aa[match(colnames(sce), rownames(tcr))] sce$cdr3s_aa <- cdr3s_aa # filter out those cells without a clonotype because they are not of interest # for us sce <- sce[,!is.na(sce$clonotype)] return(sce) } # Add the information of the TCR chains and the clonotypes to our data sceRNA$iLN <- addTCRMetaData( sce = sceRNA$iLN, tcr_filepath = “./data/iLN/filtered_contig_annotations.csv”, clonotypes_filepath = “./data/iLN/clonotypes.csv” ) sceRNA$iLN sceRNA$mLN <- addTCRMetaData( sce = sceRNA$mLN, tcr_filepath = “./data/mLN/filtered_contig_annotations.csv”, clonotypes_filepath = “./data/mLN/clonotypes.csv” ) sceRNA$mLN sceRNA$skin <- addTCRMetaData( sce = sceRNA$skin, tcr_filepath = “./data/skin/filtered_contig_annotations.csv”, clonotypes_filepath = “./data/skin/clonotypes.csv” ) sceRNA$skin sceRNA$spleen <- addTCRMetaData( sce = sceRNA$spleen, tcr_filepath = “./data/spleen/filtered_contig_annotations.csv”, clonotypes_filepath = “./data/spleen/clonotypes.csv” ) sceRNA$spleen

BOX 5 **R code for harmonization of clonotypes.**

**Table:** 

# set up clonotype data frame df_clonotypes <- data.frame( clonotype = sceRNA$iLN$clonotype, clonotype_n = as.numeric(gsub(“clonotype”, ““, sceRNA$iLN$clonotype)), cdr3s_aa = sceRNA$iLN$cdr3s_aa ) df_clonotypes <- df_clonotypes[order(df_clonotypes$clonotype_n)], filter <-!duplicated(df_clonotypes$clonotype) df_clonotypes <- df_clonotypes[filter], # function to transform the clonotypes addClonotypesToDataFrame <- function(clonotypes_df, sce) { n_last_clonotype <- max(clonotypes_df$clonotype_n) for (i in 1:ncol(sce)) { chain <- sce$cdr3s_aa[[i]] # if there is no clonotype with the same chain, the clonotype is new # and should be added to the data frame if (!any(which(clonotypes_df$cdr3s_aa == chain))) { n_last_clonotype <- n_last_clonotype + 1 clonotypes_df <- rbind(clonotypes_df, c( paste(“clonotype”, n_last_clonotype, sep = ““), as.numeric(n_last_clonotype), chain )) } } # transform the clonotype number back to a numeric clonotypes_df$clonotype_n <- as.numeric(clonotypes_df$clonotype_n) return(clonotypes_df) } df_clonotypes <- addClonotypesToDataFrame(df_clonotypes, sceRNA$mLN) df_clonotypes <- addClonotypesToDataFrame(df_clonotypes, sceRNA$skin) df_clonotypes <- addClonotypesToDataFrame(df_clonotypes, sceRNA$spleen) # function to change clonotypes for all samples changeClonotypes <- function(sce, clonotypes_df) { for (i in 1:ncol(sce)) { chain <- sce$cdr3s_aa[[i]] new_clonotype <- clonotypes_df[which(clonotypes_df$cdr3s_aa == chain)[1]], sce$clonotype[[i]] <- new_clonotype$clonotype } return(sce) } sceRNA$mLN <- changeClonotypes(sceRNA$mLN, df_clonotypes) sceRNA$skin <- changeClonotypes(sceRNA$skin, df_clonotypes) sceRNA$spleen <- changeClonotypes(sceRNA$spleen, df_clonotypes)

### Per sample quality control and filtering of low-quality cells

A well-defined filtering strategy to select for high-quality cells is highly recommended before analysis. Different quality parameters and metrics can be used to filter out cells of low quality (24). In this workflow, we mainly use a combination of three quality parameters: the library size, the number of features and the percentage of mitochondrial DNA. All of these can be used to determine the quality of the cells. The library size is the sum of all counts in one cell, which should be sufficiently high for each cell. A small/low library size indicates possible cell death of the respective cell. However, an unusually large library size could also indicate doublets (i.e., multiple cells sequenced in one droplet). The number of detected features (in this case, genes) in each individual cell should as well be sufficiently high to ensure adequate sequencing of the cells. The last quality parameter, the percentage of mitochondrial DNA captures the percentage of reads in a cell that map to the mitochondrial genome. An unusually large number of reads assigned to mitochondrial genes in a cell indicates cell death and hence low-quality cells. For the quality control, it is advisable to operate on a per-sample level instead of applying the quality control metrics for all samples combined. The individual samples might have different levels of quality due to being sequenced or processed individually or different biological prerequisites such as tissue specific properties. Hence, only one run of quality control metrics combined on all samples could falsely indicate cells of low quality because of the above-mentioned characteristics. Furthermore, also samples that were generated in different batches should be handled separately. The sequencing properties of the individual batches can greatly differ and hence as well influence the resulting quality metrics (25). In our workflow, we use the *addPerCellQC()* function of the *scater* package (24), which follows a data-driven approach for determining adequate threshold values (**Box 6**). This function first determines the median across all cells for the above-mentioned quality control parameters. Following, for each cell the median absolute deviation (MAD) is calculated. If a quality control parameter of a cell deviates more than 3 MAD from the median in an undesired direction, the cell is considered an outlier. All cells which are considered outliers in at least one of the quality parameters are marked as low-quality cells.

**Figure 7:**
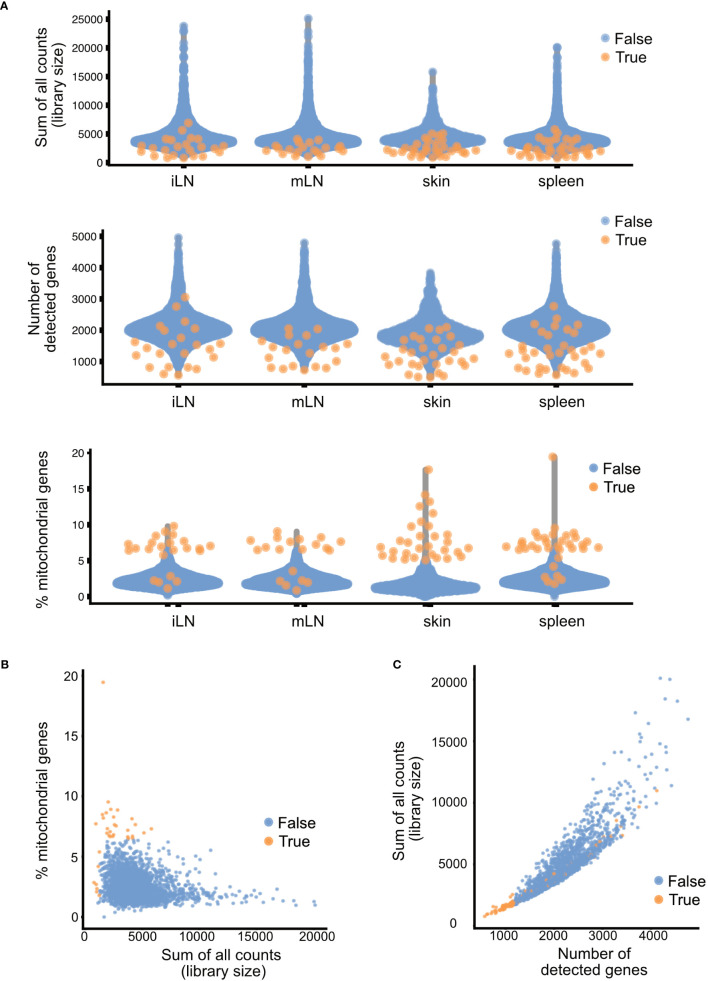
Summary of quality control metrics. **(A)** Plots of the library size, number of detected genes and mitochondrial content for each of the samples. **(B)** Scatter plots of the library size and mitochondrial content and **(C)** library size and number of detected genes. Each dot in the plot represents a cell, blue cells are of high quality, orange cells are of low quality and should be filtered out.

After identification of low-quality cells, these cells can either be removed from the data or just marked as such. The removal ensures that these cells do not interfere downstream analyses and interpretation. However, it could also be the case that interesting subpopulations of cells are marked as low-quality cells because they exhibit one of the quality control parameters. One of such examples would be hepatocytes. These cells are highly metabolically active and hence will have a high number of mitochondrial genes. Hence, it is important to check for accidental removal of high-quality cells by plotting the different quality metrics against each other and evaluating how well the different quality metrics correlate for each sample. In **Figure 7**, the different quality metrics of our samples are displayed. **Figure 7A** shows the different quality control metrics of each sample, first the library size, then the number of detected genes and lastly the number of mitochondrial genes in the data. In **Figure 7B**, we plotted for the skin sample the library size against the percentage of reads mapping to mitochondrial genes, while **Figure 7C** plots the number of genes detected against the library size. Such a multivariate approach by considering different metrics simultaneously, can lead to better decision on which cells to retain for further steps and which cells to remove. However, as the workflow presented in this paper is only of explorative nature, we will not exclude cells of low quality here. For more information, please see section “4 Per sample Quality Control and filtering of low-quality cells” in the notebook.

BOX 6 **R code for quality control and filtering (identical for all samples, showcase iLN).**

**Table:** 

iLN <- sceRNA$iLN rowData(iLN)$gene_name <- rownames(iLN) rowData(iLN)$location <- chr.loc iLN <- addPerFeatureQC(iLN) rowData(iLN) iLN <- addPerCellQC(iLN, subsets = list(Mito = is.mito)) qcstats <- perCellQCMetrics(iLN, subsets = list(Mito = is.mito)) filtered <- quickPerCellQC(qcstats, percent_subsets = “subsets_Mito_percent”) filtered colSums(as.data.frame(filtered)) table(filtered$low_n_features, filtered$high_subsets_Mito_percent) # Flag the low quality cells as discard iLN$discard <- filtered$discard # Plot the percent of mitochondrial RNA for each cell, color the cells by # whether they should be discarded or not plotColData(iLN, y = “subsets_Mito_percent”, colour_by = “discard”) # Plot the library size plotColData(iLN, y = “sum”, colour_by = “discard”) # Plot the number of detected genes plotColData(iLN, y = “detected”, colour_by = “discard”) # Plot mitochondrial RNA percentage against library size plotColData(iLN, x = “sum”, y = “subsets_Mito_percent”, colour_by = “discard”) + labs(x = “Sum of all counts (library size)”, y = “Percent mitochondrial genes”) # Plot library size against number of detected genes plotColData(iLN, x = “detected”, y = “sum”, colour_by = “discard”) + labs(x = “Number of detected genes”, y = “Sum of all counts (library size)”) # Assign the data back to our object sceRNA$iLN <- iLN # As this report is only for exploratory analyses we do not filter out any cells # Otherwise you could do # sceRNA$iLN <- iLN[,!discard]

### Quality metrics and their correlation with TCR calling

In our analysis, we were also interested in whether the quality control metrics differed between cells with TCR and cells without TCR. Especially the mitochondrial content could be of interest. Hence, we compared the cells with TCR with those without (**Table 8**). These data illustrate that around 70% or more cells of the samples have TCRs. One exception being the cells of the skin, where only around 30% of cells have associated TCRs. We can also see that the percentage of cells with a high mitochondrial content (i.e low quality cells) is nearly doubled in the cells without TCR compared to the cells with TCR. This shows that filtering of cells with associated TCR also seems to work as a way of quality control and filtering of low-quality cells. Since the VDJ library is generated from cDNA, results here also depend on the quality of the cDNA library.

**Table 8:** Different summary statistics on the input data such as number of cells per sample, number of cells with and without TCR and percentage of cells with high mitochondrial content in cells with and without TCR.

Organ	Identified cells	Cells with associated TCR	% cells with high mitochondrial content	Cells without TCR	% cells with high mitochondrial content
spleen	4,756	3,412	3.66	1,344	6.18
mLN	4,010	3,208	3.11	802	5.11
iLN	3,075	2,509	3.5	566	6.71
skin	3,339	989	7.89	2,350	16.47

### Doublet detection

In a single cell experiment, doublets are artificial observations in which two cells are sequenced as one cell. Those are especially common in droplet-based scRNA-seq protocols and usually arise from errors in cell sorting or capturing (26, 27). Doublets usually do not represent meaningful biological states and can influence the analysis of the data. For example, a mixture of two cells which were sequenced as one could be characterized as a transitionary state between two cell types or an intermediate population. The general approach for doublet detection in scRNA-seq data is the use of expression profiles of the cells. Based on their expression profile, doublets are computationally inferred from the data. In our workflow, we use the *scDblFinder()* function from the corresponding package (28) (**Box 7**). This function simulates expression profiles of possible doublets by randomly combining two cells of the data together before assigning each cell a doublet score based on its likelihood to be a double. Further details on the method and computation can be found in the *scDblFinder* documentation. Once doublets have been identified in the data, users can decide to either flag these cells or remove them completely from the data. In this context, it can be helpful to overlay the doublet classification over downstream computed clustering results to evaluate if the considered doublets are forming a distinguished cluster or display any relevant pattern. During the exploration of the data, we recommend to simply flag doublet cells but advocate for removal of the cells once the processed dataset is created. In **Figure 9C**, we can see that the identified doublets in our data to not follow a specific pattern. Overall, the number of detected doublets was also very low in our samples, less than 5% of all cells (see **Figure 9B**). For the doublet detection step, we refer readers to the provided notebook section “5 Doublet detection in the individual samples”.

BOX 7 **R code for doublet detection (identical for all samples, showcase iLN).**

**Table:** 

iLN <- sceRNA$iLN # Doublet detection iLN <- scDblFinder(iLN) # Print a statistics table table(iLN$scDblFinder.class) # Assign the object back to save the information sceRNA$iLN <- iLN # Or you can assign the object back without the cells marked as doublets # sceRNA$iLN <- iLN[, iLN$scDblFinder.class == “singlet”]

### Per-sample normalization

In scRNA-seq data, often differences in the sequencing coverage between libraries arise (29). The cause for these variations is typically technical variation in cDNA capture or PCR amplification efficiency. Since this variability does not depict true biological signal in the data, it can distort the interpretation of expression profiles. In order to prevent the influence of the technical variation on data analysis, the data is normalized (30, 31).

Usually, normalization is applied to the different batches of the data at hand. The data presented in this paper does not consist of different batches but only of different tissues. However, treating the different tissues as individual batches and normalization across tissues at this point would be detrimental to downstream analysis steps. Hence, we decided to postpone the across tissue normalization to a later point of the workflow. Nevertheless, there are intra-sample normalization methods which should be applied at this point in the analysis. One of these normalizations is a log-scaling of the expression values, as implemented in the *logNormCounts* function of the *scran* package (32) (**Box 8**). This is beneficial for downstream analysis steps such as dimensionality reduction and clustering, as the expression values become more comparable without having too extreme values. For the normalization of the counts see section “6 Per-Sample Normalization”.

BOX 8 **R code per-sample Normalization.**

**Table:** 

sceRNA <- lapply(sceRNA, logNormCounts)

### Feature selection

In an exploratory scRNA-seq analysis, characterization of heterogeneity across individual cells is often one of the major goals. In order to quantify the differences in gene expression between cells, a subset of genes is selected such that this set contains useful information about the biological variation, while removing random noise and technical differences. This process of feature selection majorly impacts the performance of downstream analyses and methods. A commonly used approach of feature selection is the selection of the most variable genes across the cells (32). The approach is based on the assumption that the biological variation of the data will manifest as an increased variation in the affected genes, hence overshadowing technical noise and irrelevant biological variation (8). In our workflow, we use the *modelGeneVar()* function of the *scran* package (32) for the computation of the variation in the genes (**Box 9**). We then use the *getTopHVGs()* function of the same package to extract the top 10% of highly variable genes (HVG) for each sample. These HVGs are then used as features for downstream steps. For the feature selection for each sample, see section “7 Feature Selection”.

BOX 9 **R code feature selection.**

**Table:** 

all.dec <- lapply(sceRNA, modelGeneVar) all.hvgs <- lapply(all.dec, getTopHVGs, prop = 0.1)

### Data integration and merging of samples

So far, we worked on each of our tissue samples individually as the presented steps yield more meaningful results if applied in a sample-specific manner. However, methods such as dimensionality reduction, clustering, marker gene detection and cell type annotation should be applied on the data set as a whole. This is why we will merge the individual *SingleCellExperiment* objects into one single object. For this, there are generally two approaches available: merging the samples without batch correction and merging after applying batch correction (33, 34). Usually, scRNA-seq data sets do not only contain different samples and tissues but also different batches. As previously discussed in this manuscript, there are technical differences between samples of different batches which can influence the results. We would like to filter out these technical differences to focus on biological variation between samples. In our workflow, we will present both approaches, batch-corrected and -uncorrected. In the uncorrected approach, we first apply the across sample normalization using the *multiBatchNorm()* function of the *batchelor* package (33). Afterwards, the metadata of the individual samples is synchronized before merging the objects into one *SingleCellExperiment* object (**Box 10**). In the batch-corrected approach, we use the *RunHarmony* function of the *harmony* package (https://CRAN.R-project.org/package=harmony), after transforming our *SingleCellExperiment* object to a *Seurat* object (**Box 11**). Here, the data is already merged at read-in and processed as a whole, following the usual Seurat workflow (19). When inspecting the data further after merging, we realized that the batch correction was too stringent on our data and overcorrected for reasonable and important biological characteristics of the skin sample (**Figures 8A, B**). Hence, we will use the uncorrected, merged *SingleCellExperiment*. The code for uncorrected as well as batch-corrected merging of the data is shown in section “8 Data integration and merging of samples”. To further showcase the effect of batch correction, we tried to integrate our data with a publicly available dataset presented in (15). From this dataset, we used the skin, spleen and LN sample to match the data presented in this paper. After downloading and reading the data as presented earlier in this paper, we tried to integrate and harmonize the two datasets using the *harmony* package. **Figures 8C, D** show the results of the integrated dataset. The code for these steps can be found in the notebook in section “8.3 Integration with publicly available data”.

BOX 10 **R code uncorrected integration.**

**Table:** 

# normalize counts across the samples rescaled <- multiBatchNorm(sceRNA) # extract the individual samples iLN <- rescaled$iLN mLN <- rescaled$mLN skin <- rescaled$skin spleen <- rescaled$spleen # combine the selected features combined.dec <- combineVar(all.dec) chosen.hvgs <- combined.dec$bio > 0 sum(chosen.hvgs) # Synchronizing the metadata for cbind()ing. rowData(iLN) <- rowData(iLN)[, c(“gene_name”, “location”)] rowData(mLN) <- rowData(mLN)[, c(“gene_name”, “location”)] rowData(skin) <- rowData(skin)[, c(“gene_name”, “location”)] rowData(spleen) <- rowData(spleen)[, c(“gene_name”, “location”)] # merge individual objects into one final object sce_merged <- cbind( iLN, mLN, skin, spleen )

BOX 11 **R code batch correction using harmony.**

**Table:** 

# Read in the data as seurat object as shown in “1 Create SingleCellExperiment” seurat <- NormalizeData(seurat) seurat <- FindVariableFeatures(seurat) seurat <- ScaleData(seurat) seurat <- RunPCA(seurat) DimPlot(seurat, reduction = “pca”) seurat <- RunHarmony(seurat, group.by.vars = “tissue”, plot_convergence = FALSE) seurat <- RunUMAP(seurat, reduction = ‘harmony’, dims = 1:20) seurat <- FindNeighbors(seurat, reduction = “harmony”, dims = 1:20) seurat <- FindClusters(seurat, resolution = 0.5) DimPlot(seurat, reduction = “umap”) DimPlot(seurat, reduction = “umap”, group.by = “tissue”, cols = c(“springgreen4”, “darkmagenta”, “tomato4”, “darkblue”))

**Figure 8:**
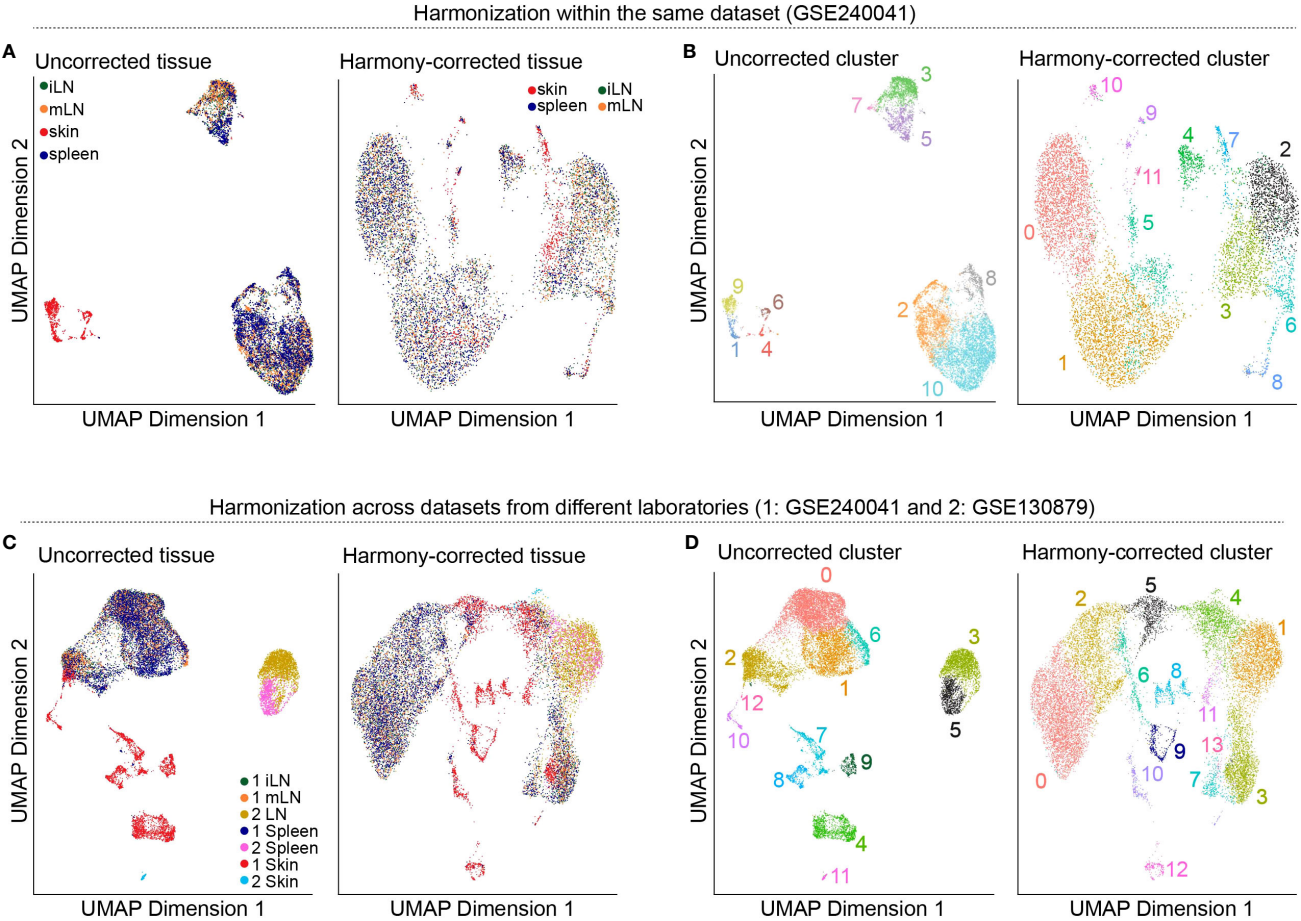
Harmonization of the data. **(A)** UMAP representation of the data before and after batch-correction using *harmony* colored by the tissue of the sample. **(B)** UMAP representation of the data before and after batch-correction using *harmony* colored by the clustering results. **(C)** UMAP representation of the integrated dataset with the publicly available data before and after batch-correction using *harmony* colored by the tissue of the sample. **(D)** UMAP representation of the integrated dataset with the publicly available data before and after batch-correction using *harmony* colored by the clustering results.

### Dimensionality reduction using principal component analysis

**Figure 9:**
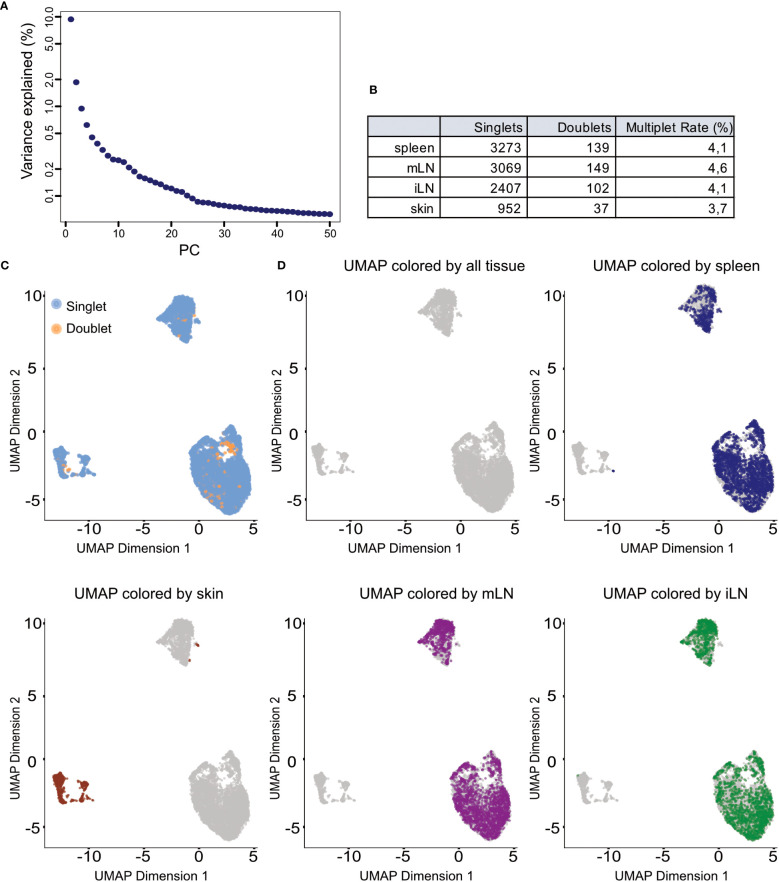
Dimensionality reduction and clustering results. **(A)** Scree plot of the variance explained by each of the calculated principal components (PC). **(B)** Summary table of detected doublets in each of the tissues. **(C)** UMAP representation of the data colored by doublet status of each cell. **(D)** UMAP representation of the data colored by the different tissue types in the input data.

**Figure 10:**
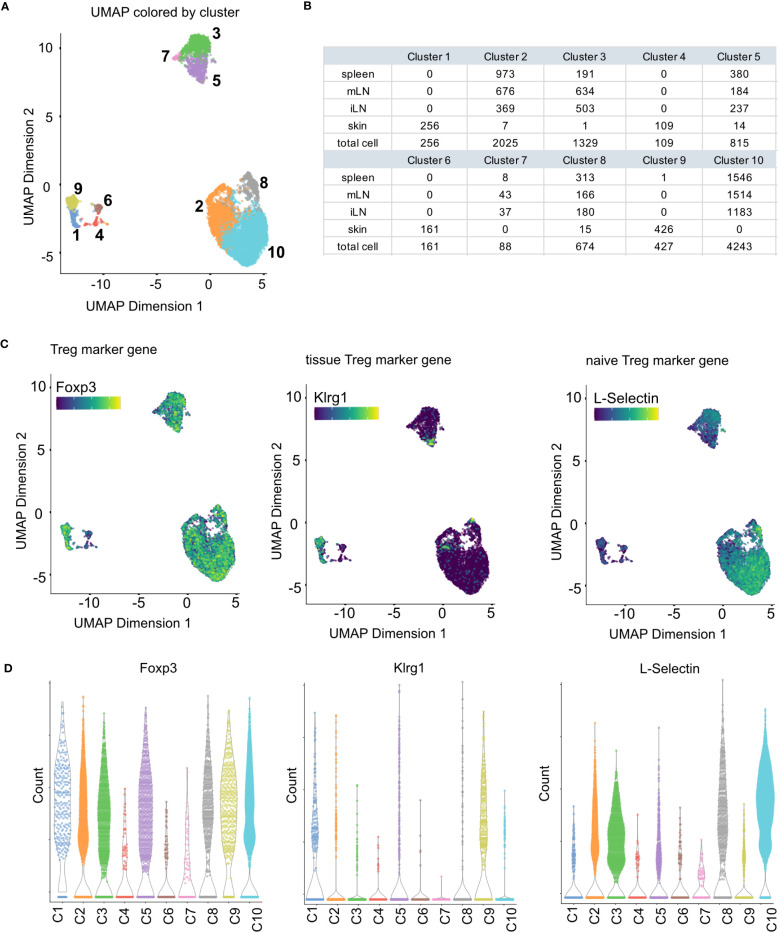
Clustering results and marker gene detection. **(A)** UMAP colored by the clusters found in the data. **(B)** Summary table of cluster composition. **(C)** UMAP representation of the data colored by the expression of different marker genes. **(D)** Violin plots showing the expression of different marker genes in the individual clusters.

In scRNA-seq analyses dimensionality reduction is used to achieve different objectives in the workflow. First, it greatly reduces the runtime of the following steps as calculations only need to be computed for a small number of dimensions compared to the large number of genes in the input data. Secondly, the procedure can reduce noise in the data by using average of genes rather than individual gene expression values. Lastly, it can also improve plotting of the data as 2/3-dimensional plots are usually easier to visualize and interpret as higher dimensional visualizations. A common approach for dimensionality reduction in scRNA-seq is Principal Component Analysis (PCA) (**Box 12**). As the first couple of principal components (PC) capture the largest amount of variance in the data, it can be assumed that these PC represent a considerable amount of biological variation of the data at hand. This way, the biological signal can be concentrated in a smaller number of PCs which can help with interpretation and visualization of the high-dimensional scRNA-seq data. In our analysis we use the *runPCA()* function from the *BiocSingular* package (8), https://doi.org/10.18129/B9.bioc.BiocSingular). The function calculates the principal components for the given data. In the shown code, we calculate the PCs based on the HVGs we determined previously, ensuring a reduced computation time while at the same time reducing the high-dimensional noise. A critical choice in the context of PCA is the choice of the number of top PCs used for downstream analyses. A helpful visualization to decide on this number is shown in **Figure 9A**. The figure plots the PCs against the percentage of variance each PC explains/captures. We see that there is a notable drop in the amount of variance explained by the PCs after the 25^th^ PC. Hence, we decided to use the first 25 PCs for downstream analyses as these capture most of the variance of our data at hand. For the PCA analysis see section “9 Dimensionality reduction using Principal Component Analysis”. Once dimensionality reduction is applied, we can also calculate a t-SNE or UMAP representation of our data (35, 36). Both visualization techniques are suitable for high-dimensional datasets such as scRNA-seq data. The t-stochastic neighborhood embedding (t-SNE) aims to find a low-dimensionality representation of the data that preserves the distances between points from the high-dimensionality space. Uniform manifold approximation and projection (UMAP, (36)) is another non-linear visualization technique for high-dimensionality data, similar to t-SNE. It should be mentioned that both methods are non-deterministic, meaning that they yield slightly different results each time the function is run on the data. We can prevent this by using the R function *set.seed()* using the same seed each time. In **Figures 9C, D** and **Figures 10A–C** we show the UMAP representation of our data colored by different properties of the data. We also calculated the t-SNE representations of our data colored by the same properties, the results are shown in the notebook accompanying this manuscript. For the plotting of the UMAP and t-SNE see **Box 13**.

BOX 12 **R code PCA.**

**Table:** 

set.seed(42) sce_merged <- runPCA(sce_merged, subset_row = chosen.hvgs, BSPARAM = BiocSingular::RandomParam()) # Plot scree plot of the variance explained by each PC percent.var <- attr(reducedDim(sce_merged), “percentVar”) plot(percent.var, log = “y”, xlab = “PC”, ylab = “Variance explained (%)”) # calculate UMAP and tSNE representation of the data set.seed(42) sce_merged <- runTSNE(sce_merged, dimred = “PCA”) set.seed(42) sce_merged <- runUMAP(sce_merged, dimred = “PCA”)

### Clustering

Clustering is adopted for scRNA-seq data to summarize the high-dimensional, complex data by dividing the cells into individual groups based on gene expression profiles. This greatly eases interpretation and exploration of the data, as the cells are then represented as discrete groups rather than the complex, high-dimensional space that is the origin of the data. In its nature, clustering is an explorative step of the analysis, possibly run in different iterations. In our workflow, we use the *buildSNNGraph()* function of the *scran* package (32) followed by the *cluster_walktrap()* function of the *igraph* package (**Box 13**). This function implements a graph-based clustering approach. Other approaches are for example Louvain clustering (37), vector quantization like k-means or hierarchical clustering (8). Once clusters have been calculated, they can be visualized as UMAP or t-SNE. In **Figure 10A**, we color the UMAP by the detected clusters. Together with **Figure 9B**, this shows that the skin cells form individual clusters which are clearly separated from the rest of the data. The remaining tissue types intermingle in their clusters with the separation being driven by factors other than tissue type. **Figure 10B** also highlights in a table that the skin forms exclusive clusters with only incidental, individual cells being part of tissue-mixed clusters. For more details on clustering, see section “10 Clustering” in the notebook.

BOX 13 **R code clustering.**

**Table:** 

# Calculate the clusters snn.gr <- buildSNNGraph(sce_merged, k = 25, use.dimred = “PCA”) clusters <- igraph::cluster_walktrap(snn.gr)$membership # See which tissue can be found in which cluster tab <- table(Cluster = clusters, Batch = sce_merged$tissue) tab # Set the cluster as colLabels of the SingleCellExperiment colLabels(sce_merged) <- factor(clusters) plotTSNE(sce_merged, colour_by = “label”) plotUMAP(sce_merged, colour_by = “label”) # color tSNE by tissue tsne <- plotTSNE(sce_merged, colour_by = “tissue”) # set custom colors, because with the original chosen colors of the method, # the individual tissues are hard to distinguish. tsne <- tsne + scale_fill_manual( values = c( skin = “tomato4”, spleen = “darkblue”, iLN = “springgreen4”, mLN = “darkmagenta” ), aesthetics = “colour” ) # plot the tSNE tsne # color UMAP by tissue umap <- plotUMAP(sce_merged, colour_by = “tissue”) # set custom colors, because with the original chosen colors of the method, # the individual tissues are hard to distinguish. umap <- umap + scale_fill_manual( values = c( skin = “tomato4”, spleen = “darkblue”, iLN = “springgreen4”, mLN = “darkmagenta” ), aesthetics = “colour” ) # plot the UMAP Umap # plot tSNE and UMAP colored by doublet identification of cells plotTSNE(sce_merged, colour_by = “scDblFinder.class”) plotUMAP(sce_merged, colour_by = “scDblFinder.class”)

### Marker gene detection

After clustering the data in the previous workflow step, the interpretation of the data can be further facilitated by characterizing marker genes (38). Marker genes are genes that drive the separation between the individual clusters, and the identification of such genes helps identifying possible functions and biological meaning of the individual clusters. The general strategy to determine marker genes of individual clusters is a pairwise comparison of all the clusters to calculate scores which quantify the differences in gene expression. In our analysis we use the *scoreMarkers()* function from the *scran* package (32) for this analysis step (**Box 14**). The function compares each of the clusters in pairs. Pairwise comparisons provide the advantage of providing more information about the markers which is beneficial to the interpretation. Also, in contrast to the approach of comparing one cluster against the average of all remaining cells, pairwise comparisons are more robust against population composition and uneven subpopulation sizes. The *scoreMarkers()* function calculates different effect size summaries to quantify the difference in gene expression between the clusters. The one we use in our workflow, visualized in **Figure 10D**, is the log fold-change, where we use the genes with the highest log fold-change between clusters as our marker genes for each cluster. There are also other metrics available in the function. For users interested in those, we refer to the documentation of the *scoreMarkers()* function. For the marker gene detection, see section “11 Marker gene det

BOX 14 **R code marker gene detection.**

**Table:** 

# score the marker genes between the individual pairs of clusters markerGenes <- scoreMarkers(sce_merged, colLabels(sce_merged)) # extract marker genes for cluster 1, 2 and 9 markerGenes_cluster1 <- as.data.frame(markerGenes[[1]]) markerGenes_cluster2 <- as.data.frame(markerGenes[[2]]) markerGenes_cluster9 <- as.data.frame(markerGenes[[9]]) # generate a data table of the top 20 marker for each of the selected clusters DT::datatable(head(markerGenes_cluster1[order(markerGenes_cluster1$mean.logFC.detected, decreasing = TRUE)], n = 20)) DT::datatable(head(markerGenes_cluster2[order(markerGenes_cluster2$mean.logFC.detected, decreasing = TRUE)], n = 20)) DT::datatable(head(markerGenes_cluster9[order(markerGenes_cluster9$mean.logFC.detected, decreasing = TRUE)], n = 20)) # plot the expression of the top 6 marker genes for each cluster in every of # the clusters plotExpression( sce_merged, features = head(rownames(markerGenes_cluster1)), x = “label”, colour_by = “label” ) plotExpression( sce_merged, features = head(rownames(markerGenes_cluster2)), x = “label”, colour_by = “label” ) plotExpression( sce_merged, features = head(rownames(markerGenes_cluster9)), x = “label”, colour_by = “label” )

### TCR repertoire diversity

TCR V(D)J sequencing coupled with single-cell RNA sequencing enables profiling of paired TCRα and TCRβ chains at single-cell resolution with coupled global gene expression in the same cell (39, 40). This analysis makes it possible to characterize T-cell clonal expansion in steady state and in disease, as well as tracking shared T-cell clonotypes between different tissues. In our analysis, we wanted to use this information to evaluate if there are shared TCR chains between different tissues as well as different clusters. We also wanted to evaluate for each tissue and cluster which chains were only found once compared to chains found multiple times.

**Figure 11:**
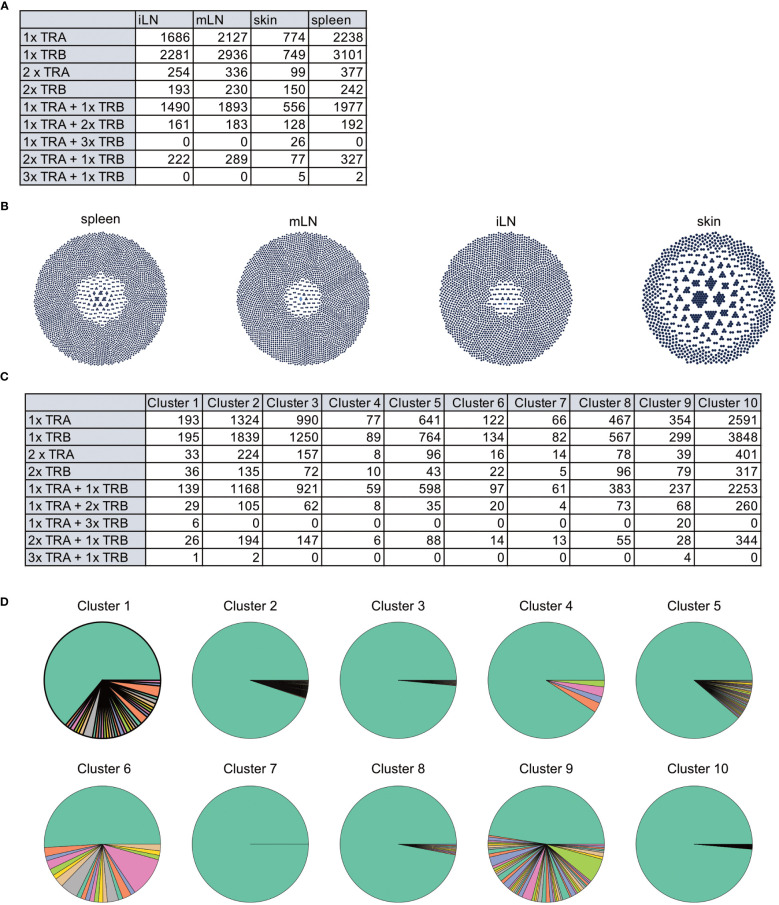
TCR diversity in tissue CD4+ T Cells of an individual animal. **(A)** Table of number of cells with different combinations of TCR chains in the individual tissues. **(B)** Visualization of the TCR diversity in the individual tissues. **(C)** Table of number of cells with different combinations of TCR chains in the individual clusters. **(D)** Pie charts visualizing the clonality of TCR in the clusters. TCR chains found only once per cluster were grouped and colored in green, while the remaining portion of the pie chart visualizes TCR chains with multiple occurrences.

**Figure 12:**
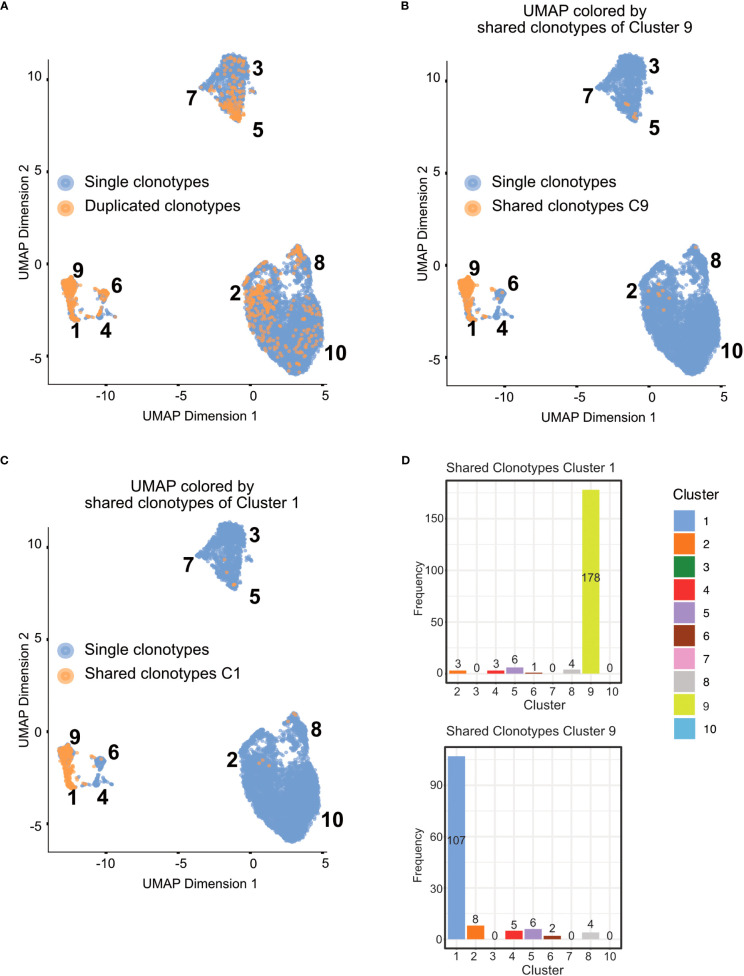
UMAP plotted by shared clonotypes. **(A)** UMAP representation of the data plotted by whether a clonotype is shared between cells of different tissues. The overlaying numbers represent the clusters of the cells shown in **Figure 9A**. **(B)** UMAP representation of the data colored by TCR chains shared with cells in cluster 9. **(C)** UMAP representation of the data colored by TCR chains shared with cells in cluster 1. **(D)** Barplot of the number of shared clonotypes of cluster 1 (upper) and 9 (lower) with the other remaining clusters. .

In **Figure 11**, we displayed several different statistics of the TCR chains in our data. **Figure 11A** shows a summary table of the occurrences of different combinations of TCR chains in the different tissues. We can see that most cells in our data have at least one TCRβ chain, followed by cells with at least one TCRα chain and cells with one TCRα and one TCRβ. **Figure 11B** visualizes the clonality of the TCR chains in the individual tissues. Here, we can see that most chains of the lymphoid organs only occur once, while some chains can be found multiple times. The highest TCR diversity can be found in the skin. **Figure 11C** shows a similar summary table as part **Figure 11A**, this time separated into the individual clusters calculated for our data set. Here, we can observe similar patterns as for the distribution of TCRs in the individual tissues. Lastly, **Figure 11D** shows pie charts of the clonality of the TCR in the individual clusters, in which we grouped all the TCR which occurred only once in the clusters. These are shown in green, while the remaining proportion of each pie chart is composed of TCR which have multiple occurrences in a cluster. Here, we can see that nearly each cluster has TCR chains which can be found more than once except for cluster 7. In a second step, we also wanted to analyze if there are TCR chains which were shared by cells of different tissues (**Box 15**). In **Figure 12A**, we plotted the UMAP representation of our data colored by whether TCR chains are shared by cells of different tissue origin. We can see that there are a lot of TCR chains shared between different tissues. In **Figures 12B, C**, we colored the UMAP by the occurrence of TCR chains found in cells of either cluster 9 or cluster 1 (**Box 16**). In **Figure 12B**, we can see that there are a lot of TCR chains shared between cluster 9 and 1 which are both composed of exclusively skin cells. However, there are also TCR shared with cells in cluster 5 and 2. **Figure 12C** shows that TCR chains of cluster 1 are also shared with cells in cluster 2, 5 and 8. **Figure 12D** shows the shared clonotypes of Cluster 1 and 9 between the other clusters. For the marker gene detection, see section “12 TCR repertoire diversity” in the notebook.

BOX 15 **R code TCR repertoire analysis.**

**Table:** 

chains_frequency <- table(sce_merged$cdr3s_aa) chains_duplicated <- chains_frequency > 1 is_duplicated_chains <- sapply(sce_merged$cdr3s_aa, function(x) chains_duplicated[[x]]) which_chain <- sapply(sce_merged$cdr3s_aa, function(x) if(chains_duplicated[[x]]){ x }else{NA}) sce_merged$duplicated_chains <- is_duplicated_chains sce_merged$which_duplicated_chain <- which_chain clonotype_frequency <- table(sce_merged$clonotype) clonotype_duplicated <- clonotype_frequency > 1 is_duplicated_clonotype <- sapply(sce_merged$clonotype, function(x) clonotype_duplicated[[x]]) which_clonotype <- sapply(sce_merged$clonotype, function(x) if(clonotype_duplicated[[x]]){ x }else{NA}) sce_merged$duplicated_clonotype <- is_duplicated_clonotype sce_merged$which_duplicated_clonotype <- which_clonotype plotUMAP(sce_merged, color_by = “duplicated_clonotype”, order_by = “duplicated_clonotype”)

BOX 16 **R code Clonotypes shared between clusters (identical for both, showcase cluster 1).**

**Table:** 

clonotype_cluster1 <- sce_merged[, colLabels(sce_merged) == “1”]$clonotype clono_cluster1_other_clusters <- sce_merged$clonotype %in% clonotype_cluster1 sce_merged$clonotype_cluster1_shared <- clono_cluster1_other_clusters # overlay shared clonotypes on the UMAP plotUMAP(sce_merged, color_by = “clonotype_cluster1_shared”, order_by = “clonotype_cluster1_shared”) # plot shared clonotypes as barplot data <- as.data.frame(table(sce_merged$clonotype_cluster1_shared, colLabels(sce_merged))) data <- data[data$Var1 == TRUE], data <- data[!data$Var2 == 1, c(2:3)] colnames(data) <- c(“Cluster”, “Frequency”) ggplot(data, aes(x = Cluster, y = Frequency, fill = Cluster, label = Frequency)) + geom_bar(stat = “identity”) + geom_text(size = 5, position = position_stack(vjust = 0.5)) + theme_bw()

### Cell type annotation

**Figure 13:**
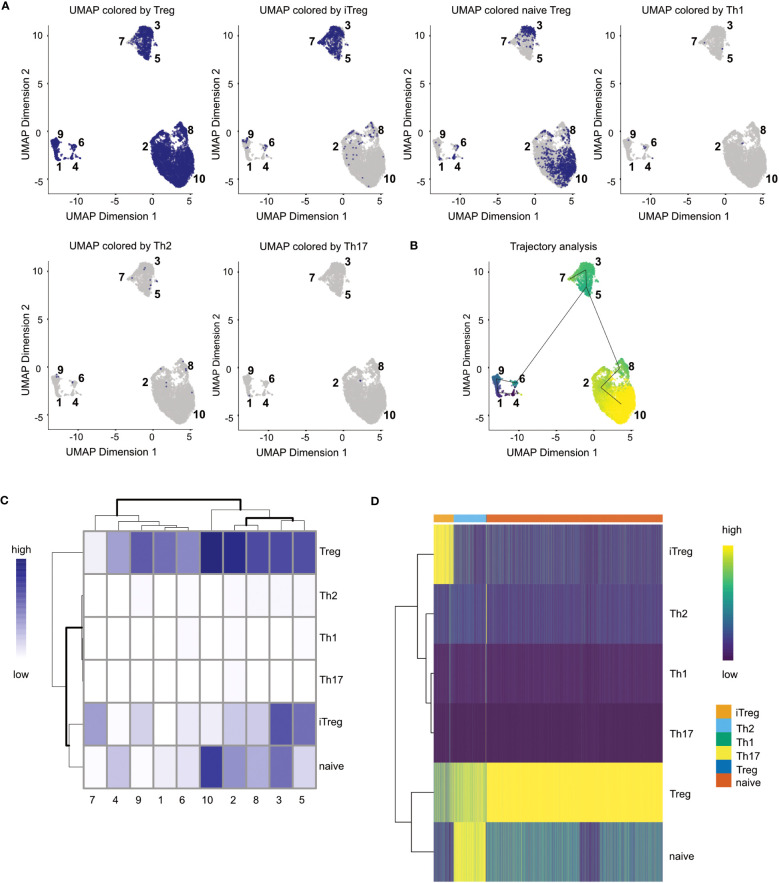
Cell type annotation results. **(A)** UMAP colored by assigned cell types for each cell. **(B)** Trajectory analysis of the data plotted on the UMAP **(C)** Heatmap of cell type distribution across clusters. **(D)** Heatmap of matching similarity of each cell to the different cell types in the reference.

Cell type annotation is arguably one of the most critical yet challenging step of a scRNA-seq analysis (41–43), as the concept of a cell type itself and the distinction of different cell types is a highly discussed topic (44, 45) Transcriptomic profiles of single cells still make it possible to assign cell types to the individual cells of a scRNA-seq data set (46). Usually, this is done using an appropriate reference data set with each cell being assigned a cell type based on the most similar cell in the reference data. In our workflow, we will present the methods of *SingleR* for cell type annotation (47) (**Box 17**). Technically, any published and carefully labeled bulk or single-cell RNA-seq data set can be used as reference data set. However, the quality of the resulting assigned cell types heavily depends on the compatibility of the data at hand and the reference data. Also, the reference data should ideally contain a variety of cells which comprises all the cell types expected in the scRNA-seq data at hand. A large variety of suitable reference data sets can be found in the R package *celldex* (47). In our workflow, we use an unpublished, in-house reference data set consisting of different T-cell subpopulations for cell type annotation and the visualizations shown in **Figure 13**. However, we also present in the HTML report how to use reference data sets from the *celldex* package (**Box 17**). After a suitable reference data set has been selected, the cell types can simply be annotated by calling the *SingleR()* function with the input data and the reference data as shown in our workflow. The results can be plotted in a heatmap as scores of the different labels to cells. An example can be seen in **Figure 13C**. Ideally, each cell should have one label with a high score compared to all other labels. **Figure 13D** we plot the composition of the individual clusters with the available cell types. We see that most clusters mainly consist of one to two cell types, with all clusters including Tregs. In **Figure 13** we plot the same results as an overlay over the UMAP representation of our data. Here as well we can see a nice distribution and clustering of the individual cell types, with all clusters having Treg cells. As mentioned above, another approach to cell type annotation is the use of marker genes (**Box 18**). In our workflow, we also did cell type annotation based on known marker genes for specific T cell subpopulations. In **Figure 10C** some of the markers are showcased and we can see the expression of individual selected marker genes in the UMAP representation of the data. **Figure 10C** shows violin expression plots of the marker genes in the individual samples. Combined with automated reference-based methods, this can support the interpretation and identification of cell types of the data at hand. For the cell type annotation see also section “12 Cell type annotation using reference data and custom markers” in the notebook.

BOX 17 **R code cell type annotation using a reference data set.**

**Table:** 

ref_annot_immgen <- ImmGenData() # Calculate cell type annotations celltype_immgen_main <- SingleR(test = sce_merged, ref = ref_annot_immgen, labels = ref_annot_immgen$label.main, BPPARAM = BiocParallel::MulticoreParam(6)) celltype_immgen_fine <- SingleR(test = sce_merged, ref = ref_annot_immgen, labels = ref_annot_immgen$label.fine, BPPARAM = BiocParallel::MulticoreParam(6)) # summarize cell type annotation results table(celltype_immgen_main$labels) table(celltype_immgen_fine$labels) # save results as meta data in the SingleCellExperiment object sce_merged$celltype_immgen_main <- celltype_immgen_main$labels sce_merged$celltype_immgen_fine <- celltype_immgen_fine$labels # plot UMAP and tSNE representation colored by the assigned cell types from the # main labels plotTSNE(sce_merged, colour_by = “celltype_immgen_main”, text_by = “celltype_immgen_main”) plotUMAP(sce_merged, colour_by = “celltype_immgen_main”, text_by = “celltype_immgen_main”) # plot UMAP and tSNE representation colored by the assigned cell types from the # fine labels plotTSNE(sce_merged, colour_by = “celltype_immgen_fine”, text_by = “celltype_immgen_fine”) plotUMAP(sce_merged, colour_by = “celltype_immgen_fine”, text_by = “celltype_immgen_fine”) # plot a heatmap of the degree of matching of the individual cells to the # available cell type labels in the reference data plotScoreHeatmap(celltype_immgen_main) # plot a heatmap of cluster to cell types, showing which cell type can found in # the individual clusters tab <- table(Assigned = celltype_immgen_main$pruned.labels, Cluster = colLabels(sce_merged)) # Adding a pseudo-count of 10 to avoid strong color jumps with just 1 cell. pheatmap(log2(tab + 10), color = colorRampPalette(c(“white”, “darkblue”))(101))

BOX 18 **R code cell type annotation using custom markers.**

**Table:** 

# set up a list of known marker genes for certain cell types, e.g. Treg cells treg <- c(“Foxp3”, “Il2”) # p Treg cells p_treg <- c(“Rorc”, “Gata3”) # t Treg cellst_treg <- c(“Ikzf2”) # Tissue Treg tissue_treg <- c(“Batf”, “Klrg1”, “Areg”, “Ccr8”, “Il10”) # Th1 cells th1 <- c(“Tbx21”, “Ifng”) # Naive T-cells naive <- c(“Ccr7”, “Sell”, “Irf4”) # repeat these two steps for all markers of interest plotExpression(sce_merged, features = “Foxp3”, x = “label”, colour_by = “label”) plotUMAP(sce_merged, color_by = “Foxp3”, order_by = “Foxp3”)

### Trajectory analysis

A large variety of biological processes can be represented as a continuum of biological changes in the cellular state. This is especially true of cell type differentiation which can for example be observed in different T-cell subpopulations. In our high dimensional scRNA-seq data, we want to characterize this process of differentiation by finding a trajectory. Associated with a trajectory is the pseudotime, which is the position of each cell along the trajectory and could for example represent the state of differentiation of a cell along a continuous process. Pseudotime helps us answer questions about the global population structure of our data. In our workflow, we use a cluster-based approach for identifying the trajectory in the data (**Box 19**). The *TSCAN* (48) algorithm implemented in the corresponding package first computes cluster centroids of the determined clusters before forming a minimum spanning tree (MST). **Figure 12B** shows the results of our trajectory analysis. The pseudotime ranges from dark to light colors, meaning cells with a dark blue color have an early pseudotime than yellow-colored cells. In the case of the presented data, a trajectory analysis might not yield too many additional insights on the data because of the overall composition of the data. However, in projects and datasets where continuous processes are under investigation, a trajectory analysis might yield additional insight of the data. For the trajectory analysis, see section “13 Trajectory Analysis” in the notebook.

BOX 19 **R code trajectory analysis.**

**Table:** 

by.cluster <- aggregateAcrossCells(sce_merged, ids = colLabels(sce_merged)) centroids <- reducedDim(by.cluster, “PCA”) # Set clusters = NULL as we have already aggregated above. mst <- createClusterMST(centroids, clusters = NULL) mst line.data <- reportEdges(by.cluster, mst = mst, clusters = NULL, use.dimred = “UMAP”) plotUMAP(sce_merged, colour_by = “label”) + geom_line(data = line.data, mapping = aes(x = dim1, y = dim2, group = edge)) map.tscan <- mapCellsToEdges(sce_merged, mst = mst, use.dimred = “PCA”) tscan.pseudo <- orderCells(map.tscan, mst) head(tscan.pseudo) common.pseudo <- averagePseudotime(tscan.pseudo) plotUMAP(sce_merged, colour_by = I(common.pseudo), text_by = “label”, text_colour = “red”) + geom_line(data = line.data, mapping = aes(x = dim1, y = dim2, group = edge))

## Methods – interactive data exploration using *iSEE*

**Figure 14:**
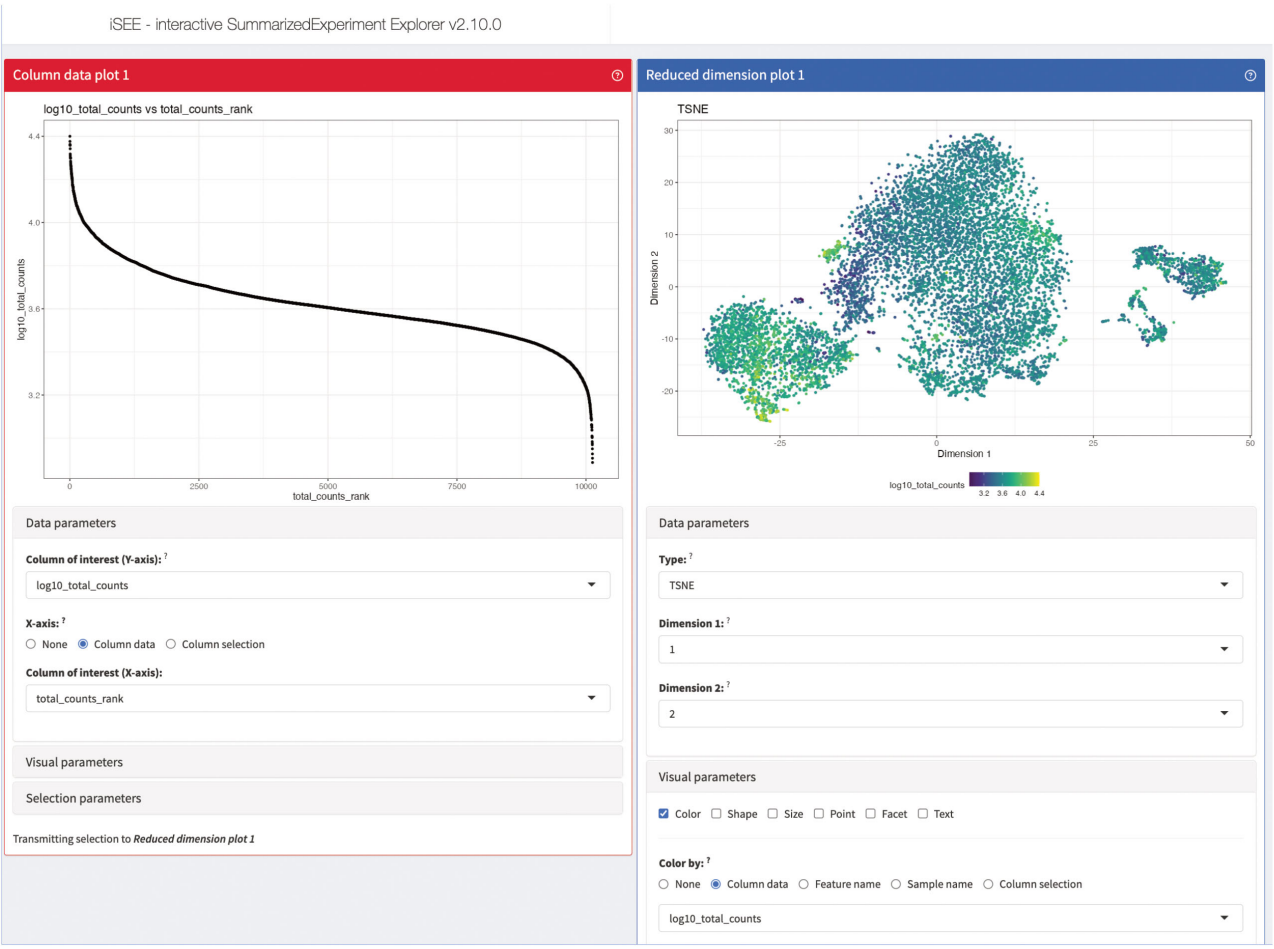
Quality control panels of our iSEE instance. The column data plot 1 on the left plots the library size of each cell in decreasing order. The reduced dimension plot 1 on the right shows the t-SNE presentation of our data colored by the log-normalized library size of each cell. Dark cells have a small library size, while yellow cells have a large library size.

For most data analysis workflows, one of the most crucial and time-consuming steps is the data exploration, usually accompanied by a lot of different data visualizations (49). This is also the case for scRNA-seq where the data usually is not only complex, but also large in size. Reiterating data exploration and visualizations steps can be beneficial to the data analysis and can help to compact and facilitate data interpretation. An excellent tool for interactive and iterative data exploration and visualization for scRNA-seq data is *iSEE* (50). *iSEE* provides a flexible framework which is compatible with a lot of different data types and can be dynamically adapted to the respective data set at hand. Each instance of *iSEE* can be customized to the individual data set by selecting the most suitable visualization and exploration techniques in form of different panels provided by *iSEE* (**Figures 14****–** **16**). As an input to *iSEE*, users have to provide a *SummarizedExperiment* object (*SingleCellExperiment* being a derivative class, with features tailored to single cell assays). This format is commonly returned by most packages in the *Bioconductor* ecosystem. In our workflow, the data is also already saved as a *SingleCellExperiment* object from the beginning, so the data presented here can easily and directly be explored with *iSEE*. In our workflow, we will present different panels of *iSEE* to demonstrate the possibilities of the application. For this, we present a customized panel layout that can be achieved using the code shown in “14 Interactive data exploration using iSEE”. The first two panels we add to our *iSEE* instance are quality control-related and plot the library size as well as a t-SNE of the log-normalized library size (**Figure 14**). The plots help to identify clusters of low-quality cells and can also be used to detect quality control or normalization errors.

**Figure 15:**
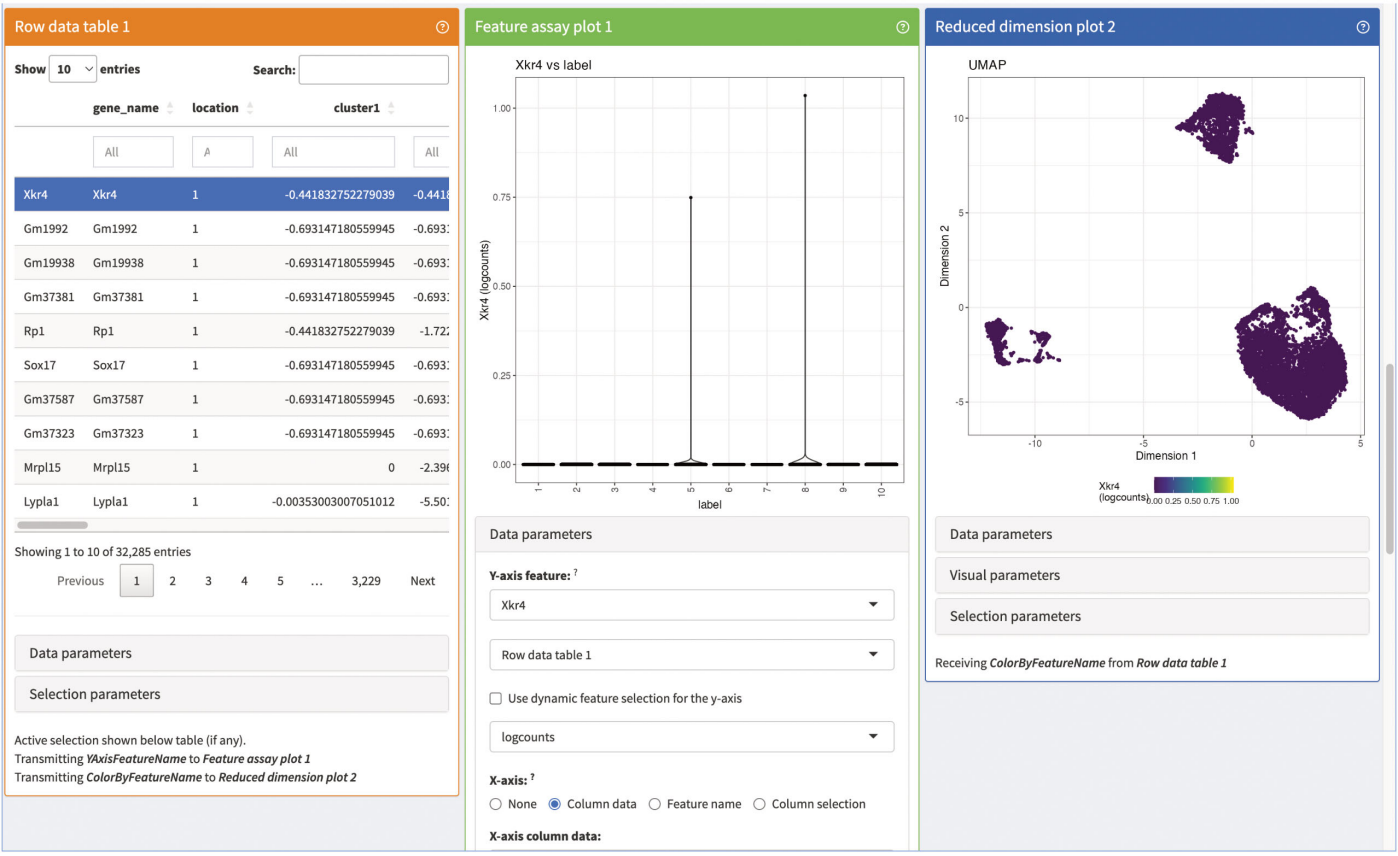
Marker gene panels of our iSEE instance. The row data Table 1 contains a table of the different marker genes of the individual clusters. The feature assay plot 1 shows a violin plot of the expression of the selected marker gene of the row data Table 1. Lastly, the reduced dimensions plot 2 shows the UMAP representation of our data colored by the expression of the selected marker in the row data Table 1.

**Figure 16:**
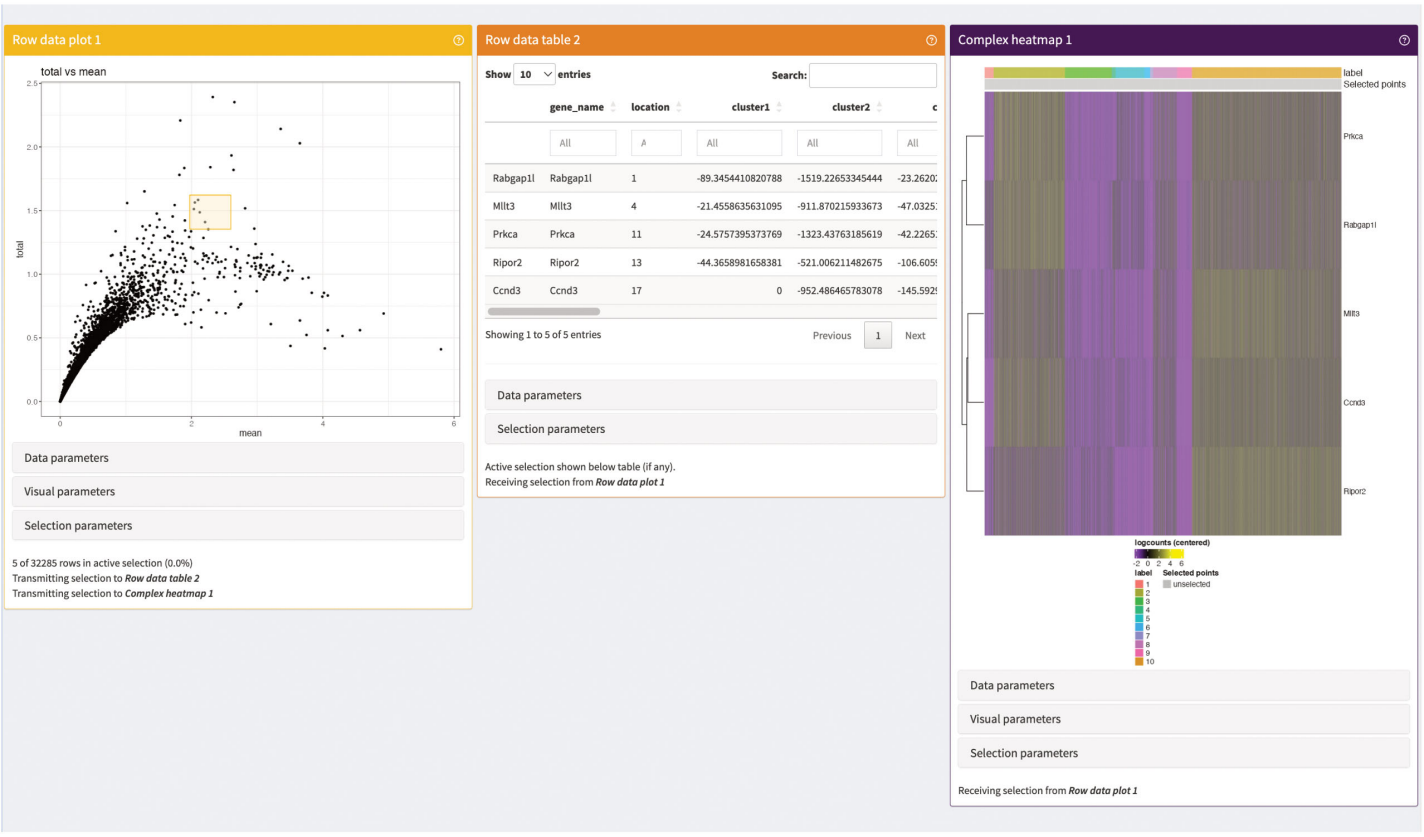
Gene summary panels of our iSEE instance. The Row data plot visualizes the mean-variances trend of the genes in our data. The Row data table is an interactive table displaying statistics on the genes selected in Row data plot (yellow square). The Complex heatmap shows a heatmap of the expression of the selected genes (yellow square) in the individual cells of the data.

Next, we add panels to visualize the marker genes of individual clusters (**Figure 15**). The panels consist of a summarization table, an expression plot of individual marker genes in the clusters as well as an UMAP of the expression of selected marker genes. All three panels are interactive and connected, so that users can evaluate different marker genes. Lastly, we present summaries on the counts of individual genes in the panels shown in **Figure 16**. The panels summarize the expression of the genes in the data as a table as well as an expression heatmap and can help explore different genes of interest in the data. As shown here, *iSEE* provides several different summary statistics and visualizations for the data. Besides the showcased panels here, there is a variety of other different panels available. This can greatly benefit the data analysis by being an interactive and reproducible way for data exploration and visualization.

## Data availability statement

Publicly available datasets analyzed in this manuscript can be found at the Gene Expression Omnibus (GEO) database, with accession code GSE240041. We included previously published datasets, also available at GEO under the accession code GSE130879. The code generated throughout this manuscript is available at the GitHub repository https://github.com/imbeimainz/scRNAseq_scTCRseq_TissueTcells. The rendered HTML notebooks accompanying the analyses presented can be found on Zenodo (https://zenodo.org/record/8338200).

## Ethics statement

Murine organs and tissues were harvested according to the regulations of the German Animal Welfare Act (§4 Tierschutzgesetz).

## Author contributions

Conceptualization: MD and FM; Methodology: ASN, SSH, FM, and MD; Software: ASN, SSH, KLB, MV, MD, and FM; Investigation and Resources: ASN, SSH, KLB, MD, and FM; Writing – Original Draft: ASN, SSH, MD, and FM; Writing – Review and Editing: ASN, SSH, MD, and FM; Visualization: ASN and SSH; Supervision: MD and FM; Project Administration: MD and FM; Funding Acquisition: MD and FM. All authors contributed to the article and approved the submitted version.
