# Supplementary material for: Using combined single-cell gene expression, TCR sequencing and cell surface protein barcoding to characterize and track CD4+ T cell clones from murine tissues
Source: Front Immunol. 2023 Oct 12;14:1241283. doi: 10.3389/fimmu.2023.1241283 (PMC10602882; doi:10.3389/fimmu.2023.1241283)
Supplement: Supplementary file 1 [file DataSheet_1.pdf]

1 Create SingleCellExperiment

1.1 Seurat

2 Gene level annotation

2.1 Seurat

3 Extracting T cells using T chain receptor information

3.1 Seurat

4 Per sample Quality Control and filtering of low-quality cells

4.1 iLN

4.2 mLN

4.3 skin

4.4 spleen

4.5 Seurat

5 Doublet detection in the individual samples

5.1 iLN

5.2 mLN

5.3 skin

5.4 spleen

5.5 Seurat

6 Per-sample Normalization

6.1 Seurat

7 Feature Selection

7.1 Seurat

8 Data integration and merging of the samples

8.1 Integration - uncorrected

8.2 Integration - Harmony

8.3 Integration with publicly available data

8.4 Seurat

9 Dimensionality reduction using Principal Component Analysis

9.1 Seurat

10 Clustering

10.1 Seurat

11 Marker gene detection
11.1 Seurat
11.1.1 Cluster 0
12 TCR repertoire diversity
13 Cell type annotation using reference data and custom markers
13.1 Reference data
13.1.1 ImmGen
13.1.2 Mouse Altlas
13.2 Use of Custom Markers
13.3 Seurat
14 Trajectory Analysis
14.1 Seurat
15 Interactive data exploration using iSEE
Session info

# Exploratory Data Analysis

CODE ▾

Using combined single-cell gene expression, TCR sequencing and cell surface protein barcoding to characterize and track CD4 T cell clones from murine tissues

Annekathrin Silvia Nedwed, Sara Salome Helbich, Kathrin Luise Braband, Michael Volkmar, Michael Delacher and Federico Marini
University Medical Center - Mainz (Germany)

11 August 2023

HIDE

```r
library("SingleCellExperiment")
library("stringr")
library("SingleR")
library("dplyr")
library("scater")
library("DT")
library("DropletUtils")
library("AnnotationHub")
library("scDblFinder")
library("scran")
library("BiocSingular")
library("batchelor")
library("iSEE")
library("pheatmap")
library("igraph")
library("TSCAN")
library("RColorBrewer")
library("harmony")
library("ensembldb")
```

# 1 Create SingleCellExperiment

In a first step, we need to import the resulting count data that we analyzed with the 10X suite. We previously aligned the sequencing data using CellRangerMulti and import the resulting feature-barcode matrix information using the `Read10X` function from the `Seurat` package.

This feature-barcode matrix information is composed of three files: the matrix.mtx file, the barcodes.tsv file and the features.tsv file. The matrix.mtx file contains the gene expression information as count matrix, while the barcodes.tsv file contains the barcode sequences of the cells. The features.tsv file maps feature id and name to feature type which can either be Gene Expression, Antibody Capture, CRISPR Guide Capture, Multiplexing Capture or CUSTOM. Reading in these files provides us the counts of our scRNA-seq data which we then use to generate a `SingleCellExperiment` object of each sample.

In our analysis, we work with four different tissue types, which all represent an individual sample: inguinal lymph nodes (iLN), mesenteric lymph nodes (mLN), skin and spleen. We read each sample in an individual `SingleCellExperiment` object. This facilitates downstream steps such as quality control which is ideally applied to each sample individually instead of once on all samples. Later on we will merge the objects for steps which are better executed on the whole data simultaneously.

In the workflow, we expect the data to be stored in a folder called data, which should contain one subfolder for each of the samples. In this subfolder, the respective matrix, features and barcodes file of each sample should be stored. Please keep in mind that the `Read10X` function is rather stringent concerning the names of these three files. If they are not named matrix.mtx, features.tsv and barcodes.tsv, the function will return an error stating it could not find the files. So when dealing with your own data, please remove any possible sample prefix and also rename the genes.tsv file to features.tsv (at the time of writing, the CellRangerMulti command (ver. 7.1) returns the features file as genes.tsv file, and the file needs to be renamed).

If you downloaded the data and script for this workflow from the respective GitHUb repository, everythings should already be in the correct folder structure.

HIDE

```
# Function to read in the data
# provide all the filepaths to the count data as a list
# as well as a list of the respective tissues
readDataset <- function(filepath_list, tissue) {
  sceRNA <- list()
  # iterate over each sample of the input data
  for (i in 1:length(filepath_list)) {
    # read the count data
    counts <- Read10X(filepath_list[[i]])
    # generate a SingleCellExperiment object
    sce <- SingleCellExperiment(assays = list(counts = counts))
    # Add the tissue type infotmation as meta data
    sce$tissue <- rep(tissue[[i]], ncol(sce))
    sceRNA <- c(sceRNA, sce)
  }
  # return the list of SingleCellExperiment objects
  return(sceRNA)
}

# input data is stored in a folder called data
filepaths <- c("../data/iLN",
               "../data/mLN",
               "../data/skin",
               "../data/spleen")

sceRNA <-
  readDataset(filepaths, tissue = c("iLN", "mLN", "skin", "spleen"))
# set the names of the objects in the list so that we can easily identify and
# access the different tissues
names(sceRNA) <- c("iLN", "mLN", "skin" , "spleen")
# Now have a look at the data
sceRNA
```

Alternatively, you can also read in the preprocessed `SingleCellExperiment` objects list, we prepared for this manuscript, which is available for download alongside the raw and aligned sequencing data.

HIDE

```
sceRNA <- readRDS("../data/sceRNA_manuscript.RDS")
```

# 1.1 Seurat

This script will also present how to process the data using the Seurat pipeline. For each section, we will show the respective Seurat code. Please note that the pipeline is more or less interchangeable between steps, by just transforming the data into the right format as shown below.

HIDE

```
library("Seurat")
# From SingleCellExperiment (sce) to Seurat (seurat)
seurat <- as.Seurat(sce)

# From Seurat (seurat) to SingleCellExperiment (sce)
sce <- as.SingleCellExperiment(seurat)
```

The respective Seurat code to read in the data is shown below. Concerning file names, storage and format, the same principles described above apply.

Here, we use the `Read10X` function as well to read the CellRangerMulti output to a count matrix. However, this time, we will use the `CreateSeuratObject` constructor of the `Seurat` package to read each of the samples into one Seurat object.

HIDE

```
# Function to read in the data
# provide all the filepaths to the count data as a list
# a well as a list of the respective tissues
readDataset <- function(filepath_list, tissue) {
  seuratRNA <- list()
  # iterate over each sample of the input data
  for (i in 1:length(filepath_list)) {
    # read the count data
    counts <- Read10X(filepath_list[[i]])
    # generate a Seurat object
    seurat = CreateSeuratObject(counts = counts)
    # Add the tissue type informtation as meta data
    seurat$tissue <- rep(tissue[[i]], ncol(seurat))
    seuratRNA <- c(seuratRNA, seurat)
  }
  # return the list of Seurat objects
  return(seuratRNA)
}

# input data is stored in a folder called data
filepaths <- c("../data/iLN",
               "../data/mLN",
               "../data/skin",
               "../data/spleen")

seuratRNA <-
  readDataset(filepaths, tissue = c("iLN", "mLN", "skin", "spleen"))
# set the names of the objects in the list so that we can easily identify and
# access the different tissues
names(seuratRNA) <- c("iLN", "mLN", "skin" , "spleen")
# Now have a look at the data
seuratRNA
```

# 2 Gene level annotation

In the next step, we do a gene level annotation. This annotation assigns each gene its respective gene name. This facilitates downstream analyses and plots, as gene names are usually easier to interpret and more widely known than the respective gene identifiers. Also we determine those genes that map to the mitochondrial genome. This is important for downstream analyses as a large proportion of mitochondrial genes indicate poor-quality cells, presumably because of loss of cytoplasmic RNA from perforated cells.

In order to map our gene identifiers to gene names, we simply take the first `SingleCellExperiment` in our list. As all samples should have the same list of genes included (as they were aligned to the same reference genome), we can use just one of the samples to match the gene names for all of the data.

In a second step we also search for all the genes mapping to the mitochondrial genome. This will be important for the quality control step.

When applying this step in your own analysis, please keep in mind that you need to choose the appropriate reference genome suiting your data at hand. Also, you could realize that there will be no genes mapping to the mitochondrial genome. This could be due to the fact, that the wrong pattern is searched for the mitochondrial genes. If all entries are NA, the pattern is most likely wrong. Please, check out different patterns and compare them to the names of your data at hand. The pattern ussually follows the lines of 'MT', 'Mt', 'mt', 'chrM', etc.

HIDE

```
sce <- sceRNA$iLN

# set up the annotation hub
ah <- AnnotationHub()
# extract the indentifiers and names for mouse data
query(ah, c("musculus", "Ensembl", "EnsDb"))
```

```
## AnnotationHub with 47 records
## # snapshotDate(): 2022-10-31
## # $dataprovider: Ensembl
## # $species: Mus musculus, Balaenoptera musculus, Mus musculus musculus, Mus
...
## # $rdataclass: EnsDb
## # additional mcols(): taxonomyid, genome, description,
## #   coordinate_1_based, maintainer, rdatadateadded, preparerclass, tags,
## #   rdatapath, sourceurl, sourcetype
## # retrieve records with, e.g., 'object[["AH53222"]]'
##
##            title
##   AH53222  | Ensembl 87 EnsDb for Mus Musculus
##   AH53726  | Ensembl 88 EnsDb for Mus Musculus
##   AH56691  | Ensembl 89 EnsDb for Mus Musculus
##   AH57770  | Ensembl 90 EnsDb for Mus Musculus
##   AH60788  | Ensembl 91 EnsDb for Mus Musculus
##   ...        ...
##   AH109651 | Ensembl 109 EnsDb for Mus musculus
##   AH109652 | Ensembl 109 EnsDb for Mus musculus
##   AH109653 | Ensembl 109 EnsDb for Mus musculus musculus
##   AH109654 | Ensembl 109 EnsDb for Mus musculus domesticus
##   AH109655 | Ensembl 109 EnsDb for Mus musculus
```

HIDE

```
ens.mm.v102 <- ah[["AH89211"]]
genes(ens.mm.v102)[, 2]
```

```
## GRanges object with 56305 ranges and 1 metadata column:
##                      seqnames            ranges strand |     gene_name
##                         <Rle>         <IRanges>  <Rle> |   <character>
##   ENSMUSG00000102693        1   3073253-3074322      + | 4933401J01Rik
##   ENSMUSG00000064842        1   3102016-3102125      + |       Gm26206
##   ENSMUSG00000051951        1   3205901-3671498      - |          Xkr4
##   ENSMUSG00000102851        1   3252757-3253236      + |       Gm18956
##   ENSMUSG00000103377        1   3365731-3368549      - |       Gm37180
##                   ...      ...               ...    ... .           ...
##   ENSMUSG00000095366        Y 90752427-90755467      - |       Gm21860
##   ENSMUSG00000095134        Y 90753057-90763485      + |      Mid1-ps1
##   ENSMUSG00000096768        Y 90784738-90816465      + |       Gm47283
##   ENSMUSG00000099871        Y 90837413-90844040      + |       Gm21742
##   ENSMUSG00000096850        Y 90838869-90839177      - |       Gm21748
##   -------
##   seqinfo: 118 sequences (1 circular) from GRCm38 genome
```

HIDE

```
# search for the mitochondrial genes
is.mito <- grepl("^mt-", rownames(sce))

chr.loc <- mapIds(
  ens.mm.v102,
  keys = rownames(sce),
  keytype = "GENENAME",
  column = "SEQNAME"
)
is.mito <- which(chr.loc == "MT")
is.mito
```

```
##  mt-Nd1  mt-Nd2  mt-Co1  mt-Co2 mt-Atp8 mt-Atp6  mt-Co3  mt-Nd3 mt-Nd4l  mt-N
d4
##   32196   32197   32198   32199   32200   32201   32202   32203   32204   322
05
##  mt-Nd5  mt-Nd6 mt-Cytb
##   32206   32207   32208
```

## 2.1 Seurat

The Gene Annotation step can be skipped in the Seurat pipeline, as Seurat objects will automatically have gene names as identifiers. Also the mitochondrial content of each cell is determined differently, hence we can skip this step in the Seurat pipeline.

# 3 Extracting T cells using T chain receptor information

In our experiment, we want to focus on T cells, specifically regulatory T cells (Tregs). As described in the accompanying manuscript, we also have the information of the VDJ library available in this analysis. In this step, we will use the information of the VDJ library to filter our dataset for cells with associated T-cell receptor (TCR). The TCR characterize the T cells of interest and is hence a good indicator for filtering.

In order to filter our data set for T cells, we add the information on the TCR chains and the clonotype of each cell to our `SingleCellExperiment` objects, before filtering out all cells without TCR chains.

HIDE

```
addTCRMetaData <- function(sce, tcr_filepath, clonotypes_filepath) {
  # Read in the information about the TCRs
  tcr <- read.csv(tcr_filepath)
  clonotypes <- read.csv(clonotypes_filepath)

  # Remove duplicated barcodes as the information is identical.
  tcr <- tcr[!duplicated(tcr$barcode),]

  # Subset to only barcode and raw clonotype column as we only use those.
  tcr <- tcr[, c("barcode", "raw_clonotype_id")]
  # Rename column to match to the clonotypes file
  names(tcr)[names(tcr) == "raw_clonotype_id"] <- "clonotype_id"

  # Extract the TCR chain information from the clonotypes file through matching
  # of the clonotypes.
  tcr <- merge(tcr, clonotypes[, c("clonotype_id", "cdr3s_aa")])

  # Reorder columns, set barcodes as rownames (to match the scRNA data)
  # and remove the barcode column as it is no longer necessary.
  tcr <- tcr[, c(2, 1, 3)]
  rownames(tcr) <- tcr[, 1]
  tcr[, 1] <- NULL

  # Add the TCR chain and clonotype information as metadata to the data
  clonotype <-
    tcr$clonotype_id[match(colnames(sce), rownames(tcr))]
  sce$clonotype <- clonotype
  cdr3s_aa <- tcr$cdr3s_aa[match(colnames(sce), rownames(tcr))]
  sce$cdr3s_aa <- cdr3s_aa

  # filter out those cells without a clonotype because they are not of interest
  # for us
  sce <- sce[,!is.na(sce$clonotype)]
  return(sce)
}

# Add the information of the TCR chains and the clonotypes to our data
sceRNA$iLN <- addTCRMetaData(
  sce = sceRNA$iLN,
  tcr_filepath = "../data/iLN/filtered_contig_annotations.csv",
  clonotypes_filepath = "../data/iLN/clonotypes.csv"
)
sceRNA$iLN
```

```
## class: SingleCellExperiment
## dim: 32285 2509
## metadata(0):
## assays(1): counts
## rownames(32285): Xkr4 Gm1992 ... AC234645.1 AC149090.1
## rowData names(0):
## colnames(2509): AAACCTGAGCGAAGGG-1 AAACCTGAGGGTTTCT-1 ...
##   TTTGTCACATACTCTT-1 TTTGTCAGTGCAGACA-1
## colData names(3): tissue clonotype cdr3s_aa
## reducedDimNames(0):
## mainExpName: NULL
## altExpNames(0):
```

HIDE

```
sceRNA$mLN <- addTCRMetaData(
  sce = sceRNA$mLN,
  tcr_filepath = "../data/mLN/filtered_contig_annotations.csv",
  clonotypes_filepath = "../data/mLN/clonotypes.csv"
)
sceRNA$mLN
```

```
## class: SingleCellExperiment
## dim: 32285 3217
## metadata(0):
## assays(1): counts
## rownames(32285): Xkr4 Gm1992 ... AC234645.1 AC149090.1
## rowData names(0):
## colnames(3217): AAACCTGAGGAATTAC-1 AAACCTGAGTCTCAAC-1 ...
##   TTTGTCATCGTCGTTC-1 TTTGTCATCTACCAGA-1
## colData names(3): tissue clonotype cdr3s_aa
## reducedDimNames(0):
## mainExpName: NULL
## altExpNames(0):
```

HIDE

```
sceRNA$skin <- addTCRMetaData(
  sce = sceRNA$skin,
  tcr_filepath = "../data/skin/filtered_contig_annotations.csv",
  clonotypes_filepath = "../data/skin/clonotypes.csv"
)
sceRNA$skin
```

```
## class: SingleCellExperiment
## dim: 32285 989
## metadata(0):
## assays(1): counts
## rownames(32285): Xkr4 Gm1992 ... AC234645.1 AC149090.1
## rowData names(0):
## colnames(989): AAACCTGAGTAGATGT-1 AAACCTGTCCATGAGT-1 ...
##   TTTGCGCAGAACTGTA-1 TTTGGTTTCCGTAGGC-1
## colData names(3): tissue clonotype cdr3s_aa
## reducedDimNames(0):
## mainExpName: NULL
## altExpNames(0):
```

HIDE

```
sceRNA$spleen <- addTCRMetaData(
  sce = sceRNA$spleen,
  tcr_filepath = "../data/spleen/filtered_contig_annotations.csv",
  clonotypes_filepath = "../data/spleen/clonotypes.csv"
)
sceRNA$spleen
```

```
## class: SingleCellExperiment
## dim: 32285 3412
## metadata(0):
## assays(1): counts
## rownames(32285): Xkr4 Gm1992 ... AC234645.1 AC149090.1
## rowData names(0):
## colnames(3412): AAACCTGAGATATACG-1 AAACCTGCAAGCCGTC-1 ...
##   TTTGTCAGTACTTCTT-1 TTTGTCATCACTTCAT-1
## colData names(3): tissue clonotype cdr3s_aa
## reducedDimNames(0):
## mainExpName: NULL
## altExpNames(0):
```

In our specific workflow, we also must transform the clonotypes as we have processed each sample individually using CellRanger. This means that for each sample, the clonotypes start with id 1 and cells with the same TCR chains across different tissues will not have the same clonotype id. In order to analyze shared clonotypes between tissues, we first apply a transformation step to assign identical TCR chains the same clonotype id across all tissues. Afterwards, we save the harmonized TCR chain and clonotype information as meta data in our `SingleCellExperiment` objects.

HIDE

```
# set up the clonotype dataframe using the first sample
df_clonotypes <- data.frame(
  clonotype = sceRNA$iLN$clonotype,
  clonotype_n = as.numeric(gsub("clonotype", "", sceRNA$iLN$clonotype)),
  cdr3s_aa = sceRNA$iLN$cdr3s_aa
)

# order the clonotypes by id number
df_clonotypes <- df_clonotypes[order(df_clonotypes$clonotype_n), ]
# filter out duplicated clonotypes
filter <- !duplicated(df_clonotypes$clonotype)
df_clonotypes <- df_clonotypes[filter, ]

# add all available clonotypes from all samples to one dataframe
addClonotypesToDataFrame <- function(clonotypes_df, sce) {
  n_last_clonotype <- max(clonotypes_df$clonotype_n)
  for (i in 1:ncol(sce)) {
    chain <- sce$cdr3s_aa[[i]]
    # if the clonotype is not available, this one is new and has to be added
    # to the dataframe
    # however we change the id of the clonotype to ensure a continous id
    # numeration
    if (!any(which(clonotypes_df$cdr3s_aa == chain))) {
      n_last_clonotype <- n_last_clonotype + 1
      clonotypes_df <- rbind(clonotypes_df,
                             c(
                               paste("clonotype", n_last_clonotype, sep = ""),
                               as.numeric(n_last_clonotype),
                               chain
                             ))
    }
  }
  # transform the clonotype number back to a numeric
  clonotypes_df$clonotype_n <-
    as.numeric(clonotypes_df$clonotype_n)
  return(clonotypes_df)
}

df_clonotypes <-
  addClonotypesToDataFrame(df_clonotypes, sceRNA$mLN)
df_clonotypes <-
  addClonotypesToDataFrame(df_clonotypes, sceRNA$skin)
df_clonotypes <-
  addClonotypesToDataFrame(df_clonotypes, sceRNA$spleen)


# change the clonotypes to their new id
changeClonotypes <- function(sce, clonotypes_df) {
  for (i in 1:ncol(sce)) {
    chain <- sce$cdr3s_aa[[i]]
    new_clonotype <-
```

```
      clonotypes_df[which(clonotypes_df$cdr3s_aa == chain)[1], ]
    sce$clonotype[[i]] <- new_clonotype$clonotype
  }
  return(sce)
}


sceRNA$mLN <- changeClonotypes(sceRNA$mLN, df_clonotypes)
sceRNA$skin <- changeClonotypes(sceRNA$skin, df_clonotypes)
sceRNA$spleen <- changeClonotypes(sceRNA$spleen, df_clonotypes)
```

# 3.1 Seurat

The TCR extraction and clonotype adapting is nearly identical in the Seurat pipeline.

HIDE

```
addTCRMetaData <-
  function(scRNA_data,
           tcr_filepath,
           clonotypes_filepath) {
    # Read in the information about the TCRs
    tcr <- read.csv(tcr_filepath)
    clonotypes <- read.csv(clonotypes_filepath)

    # Remove duplicated barcodes as the information is identical.
    tcr <- tcr[!duplicated(tcr$barcode),]

    # Subset to only barcode and raw clonotype column as we only use those.
    tcr <- tcr[, c("barcode", "raw_clonotype_id")]
    # Rename column to match to the clonotypes file
    names(tcr)[names(tcr) == "raw_clonotype_id"] <- "clonotype_id"

    # Extract the TCR chain information from the clonotypes file through matchin
g
    # of the clonotypes.
    tcr <- merge(tcr, clonotypes[, c("clonotype_id", "cdr3s_aa")])

    # Reorder columns, set barcodes as rownames (to match the scRNA data)
    # and remove the barcode column as it is no longer necessary.
    tcr <- tcr[, c(2, 1, 3)]
    rownames(tcr) <- tcr[, 1]
    tcr[, 1] <- NULL

    # Add the TCR chain and clonotype information as metadata to the data
    scRNA_data <- AddMetaData(object = scRNA_data, metadata = tcr)
    # Subset the data to only those cells with a TCR chain
    scRNA_data <- subset(scRNA_data, subset = clonotype_id != "NA")
    return(scRNA_data)
  }

# Add the information of the TCR chains and the clonotypes to our data
seuratRNA$iLN <- addTCRMetaData(
  scRNA_data = seuratRNA$iLN,
  tcr_filepath = "../data/iLN/filtered_contig_annotations.csv",
  clonotypes_filepath = "../data/iLN/clonotypes.csv"
)

seuratRNA$mLN <- addTCRMetaData(
  scRNA_data = seuratRNA$mLN,
  tcr_filepath = "../data/mLN/filtered_contig_annotations.csv",
  clonotypes_filepath = "../data/mLN/clonotypes.csv"
)

seuratRNA$skin <- addTCRMetaData(
  scRNA_data = seuratRNA$skin,
  tcr_filepath = "../data/skin/filtered_contig_annotations.csv",
  clonotypes_filepath = "../data/skin/clonotypes.csv"
```

```
)

seuratRNA$spleen <- addTCRMetaData(
  scRNA_data = seuratRNA$spleen,
  tcr_filepath = "../data/spleen/filtered_contig_annotations.csv",
  clonotypes_filepath = "../data/spleen/clonotypes.csv"
)
```

HIDE

```
df_clonotypes <- data.frame(
  clonotype = seuratRNA$iLN$clonotype,
  clonotype_n = as.numeric(gsub("clonotype", "", seuratRNA$iLN$clonotype)),
  cdr3s_aa = seuratRNA$iLN$cdr3s_aa
)
df_clonotypes <- df_clonotypes[order(df_clonotypes$clonotype_n),]
filter <- !duplicated(df_clonotypes$clonotype)
df_clonotypes <- df_clonotypes[filter,]


df_clonotypes <-
  addClonotypesToDataFrame(df_clonotypes, seuratRNA$mLN)
df_clonotypes <-
  addClonotypesToDataFrame(df_clonotypes, seuratRNA$skin)
df_clonotypes <-
  addClonotypesToDataFrame(df_clonotypes, seuratRNA$spleen)


seuratRNA$mLN <- changeClonotypes(seuratRNA$mLN, df_clonotypes)
seuratRNA$skin <- changeClonotypes(seuratRNA$skin, df_clonotypes)
seuratRNA$spleen <- changeClonotypes(seuratRNA$spleen, df_clonotypes)
```

Afterwards, we can merge all individual `Seurat` objects to one, as all downstream analysis steps can be applied to the data as whole.

HIDE

```
seurat_merged <- merge(seuratRNA$iLN,
                      y = c(seuratRNA$mLN,
                            seuratRNA$skin,
                            seuratRNA$spleen
                            ),
                      add.cell.ids = c("iLN",
                                       "mLN",
                                       "skin",
                                       "spleen"))
```

# 4 Per sample Quality Control and filtering of

# low-quality cells

In the next step, we want to remove low quality cells from our data. There are several different metrics that can be used to identify low quality cells. In this analysis, we will be using the following three:

1. library size : The library size is the sum of all counts in one cell, which should be sufficiently high for each cell. A small/low library size indicates possible cell death of the respective cell. However, an unusually large library size could also indicate doublets or multiple cells sequenced as one.
2. number of features: In comparison to the library size, the number of features on the other hand is the number of different features (i.e. genes) sequenced per cell. This should as well be sufficiently high to ensure a sufficient sequencing of the cells.
3. percentage of mitochondrial RNA: The last quality parameter, the percentage of mitochondrial DNA captures the percentage of genes in a cell that map to the mitochondrial genes. An unusually large number of mitochondrial genes in a cell indicates cell death and hence low quality cells.

In these QC steps, we assume that most of the dataset consists of high-quality cells. We then identify cells that are outliers for the various QC metrics (small library size, small number of features, high percentage of mitochondrial genes), based on the median absolute deviation (MAD) from the median value of each metric across all cells. By default, we consider a value to be an outlier if it is more than 3 MADs from the median in the “problematic” direction. This is loosely motivated by the fact that such a filter will retain 99% of non-outlier values that follow a normal distribution.

A cell that is an outlier for any of these metrics is considered to be of low quality and marked as cell to be discarded. This is captured in the discard column, which can be used for later filtering.

After identification of low quality cells, these cells can either be removed from the data or just marked as low quality cells. The removal ensures that these cells do not interfere downstream analyses and interpretation. However, it could also be that case that interesting subpopulations of cells are marked as low quality cells because they exhibit one of the quality control parameters. One of such examples would be hepatocytes. These cells are highly metabolically active and hence will have a high number of mitochondrial genes associated with them. Hence, it is important to check for accidental removal of high quality cells by plotting the different quality metrics against each other. Ideally the individual quality metrics should correlate to reinforce then assumptions that the cells are of low quality and should be excluded from the further analysis.

For the quality control, it is advisable to operate on a per-sample level instead of applying the quality control metrics for all samples combined. The individual samples might have different levels of quality due to being sequenced or processed individually or different biological prerequisites such as tissue specific properties. Hence, only one run of quality control metrics combined on all samples could falsely indicate cells of low quality because of the above-mentioned characteristics. Furthermore, also samples that were generated in different batches should be handled separately. The sequencing properties of the individual batches can greatly differ and hence as well influence the resulting quality metrics.

We will comment on the individual steps in the iLN sample case, as the steps are identical for all of the samples.

# 4.1 iLN

In a first step, we add the gene names and chromosome location of the individual genes in the data to the rowdata information, before we apply the `addPerFeatureQC` function from the `scater` package. This function is a compact wrapper that computes all the three above described quality control metrics automatically and appends the results to the existing data.

HIDE

```
iLN <- sceRNA$iLN

rowData(iLN)$gene_name <- rownames(iLN)
rowData(iLN)$location <- chr.loc
iLN <- addPerFeatureQC(iLN)

rowData(iLN)
```

```
## DataFrame with 32285 rows and 4 columns
##               gene_name    location        mean  detected
##             <character> <character>   <numeric> <numeric>
## Xkr4               Xkr4           1 0.000398565 0.0398565
## Gm1992           Gm1992           1 0.000000000 0.0000000
## Gm19938         Gm19938           1 0.000000000 0.0000000
## Gm37381         Gm37381           1 0.000000000 0.0000000
## Rp1                 Rp1           1 0.000000000 0.0000000
## ...                 ...         ...         ...       ...
## AC124606.1  AC124606.1  JH584299.1    0.000000    0.0000
## AC133095.2  AC133095.2  JH584299.1    0.000000    0.0000
## AC133095.1  AC133095.1  JH584299.1    0.000000    0.0000
## AC234645.1  AC234645.1  JH584303.1    0.000000    0.0000
## AC149090.1  AC149090.1  JH584304.1    0.532483   36.4289
```

Afterwards, we apply the `addPerCellQC` function of the `scater` package, which is also a wrapper around all three quality metrics. Then we prepare the data and display the different quality metrics in a data table. For each cell, this table displays all quality control metrics (low_lib_size, low_n_features, high_subsets_Mito_percent) and indicates whether this is applicable. The last column of the dataframe indicates whether a cell is of low quality and should be discarded.

HIDE

```
iLN <- addPerCellQC(iLN, subsets = list(Mito = is.mito))
qcstats <- perCellQCMetrics(iLN, subsets = list(Mito = is.mito))
filtered <-
  quickPerCellQC(qcstats, percent_subsets = "subsets_Mito_percent")
filtered
```

```
## DataFrame with 2509 rows and 4 columns
##         low_lib_size   low_n_features high_subsets_Mito_percent   discard
##     <outlier.filter> <outlier.filter>          <outlier.filter> <logical>
## 1              FALSE            FALSE                     FALSE     FALSE
## 2              FALSE            FALSE                     FALSE     FALSE
## 3              FALSE            FALSE                     FALSE     FALSE
## 4              FALSE            FALSE                     FALSE     FALSE
## 5              FALSE            FALSE                     FALSE     FALSE
## ...              ...              ...                       ...       ...
## 2505           FALSE            FALSE                     FALSE     FALSE
## 2506           FALSE            FALSE                     FALSE     FALSE
## 2507           FALSE            FALSE                     FALSE     FALSE
## 2508           FALSE            FALSE                     FALSE     FALSE
## 2509           FALSE            FALSE                     FALSE     FALSE
```

HIDE

```
colSums(as.data.frame(filtered))
```

```
##              low_lib_size            low_n_features high_subsets_Mito_percent 
##                        11                        66                        88 
##                   discard 
##                       143
```

We also show a compact summary of the number of cells with low library size and high percentage of mitochondrial DNA.

HIDE

```
table(filtered$low_n_features, filtered$high_subsets_Mito_percent)
```

```
##        
##         FALSE TRUE
##   FALSE  2366   77
##   TRUE     55   11
```

As this workflow is only of exploratory nature, we only flag the low quality cells rather than filtering out the cells. In your own analysis, you should plot the data before carefully deciding whether or not to filter out low quality cells.

We also highlight different ways of plotting the information on the quality of the cells. Besides plotting each individual metric, it can also be beneficial to plot two metrics against each other, as shown here. We first plot the library size against the mitochondrial content before plotting the number of features against the library size. In the plots, each dot is an individual cells colored by whether it should be discarded or not (i.e. it is of low quality). For the library size as well as for the number of detected genes, we expect the low-quality cells to gather at the bottom of the violin plot as it can be nicely observed in our data. For the percentage of genes mapping to the mitochondrial genome, on the other hand we expect the orange dots to gather at the upper end of the violin. This can as well be observed in our data. For the combined plots, we expect low quality cells to gather along the y-axis (library size vs. mitochondrial content) or to gather at the lower left corner (number of

features vs. library size). This can as well be observed in our data and indicates tha the sample is overall of high and sufficient quality.

HIDE

```
# Flag the low quality cells as discard
iLN$discard <- filtered$discard

# Plot the percent of mitochondrial RNA for each cell, color the cells by
# whether they should be discarded or not
plotColData(iLN, y = "subsets_Mito_percent", colour_by = "discard")
```

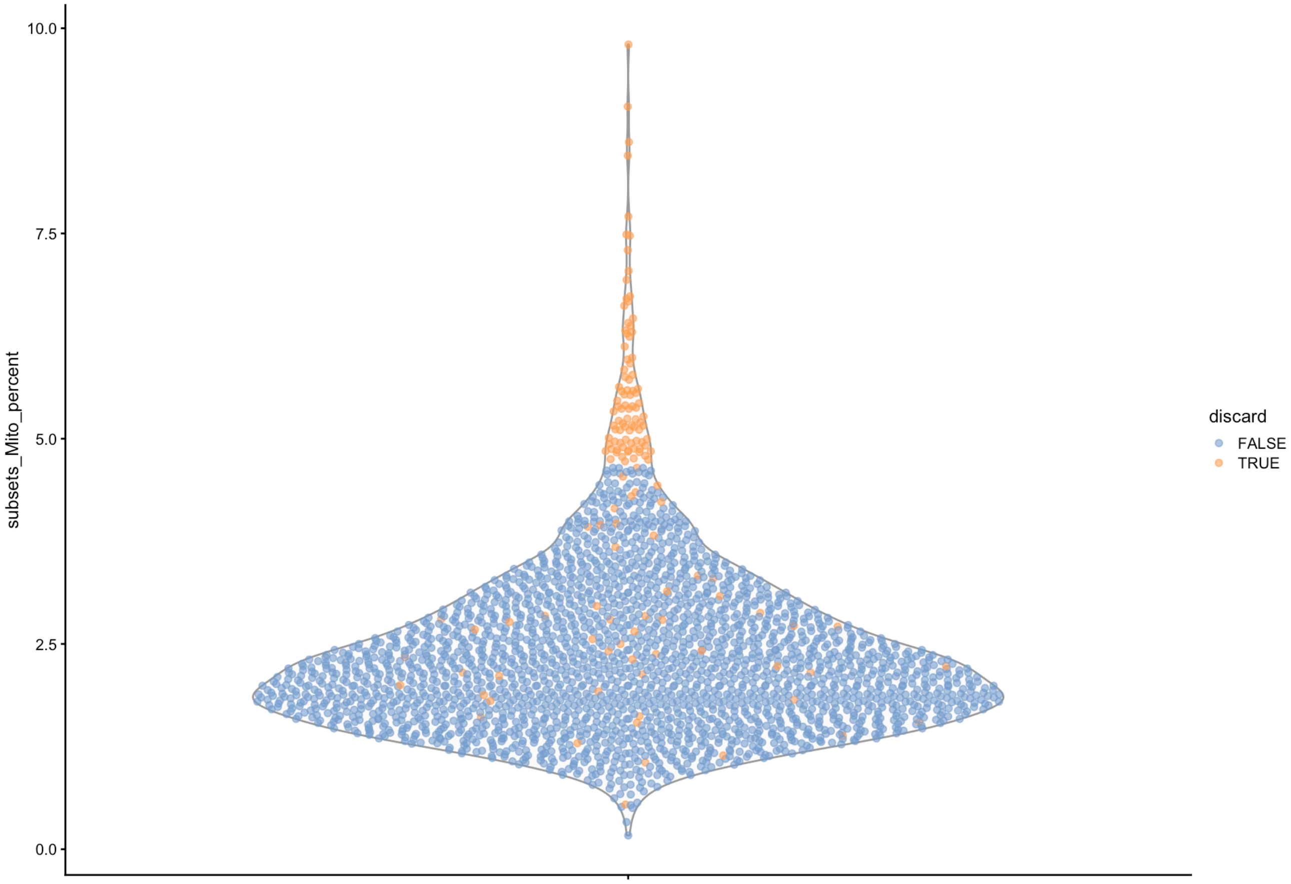


HIDE

```
# Plot the library size
plotColData(iLN, y = "sum", colour_by = "discard")
```

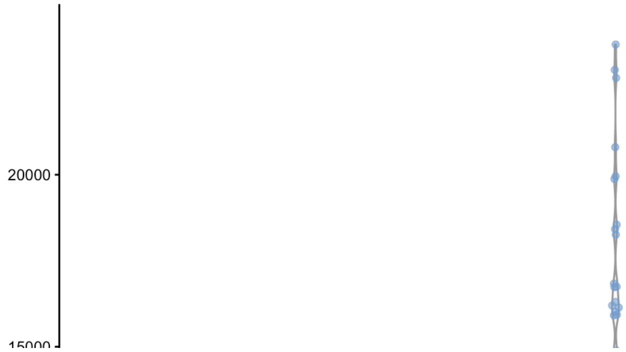

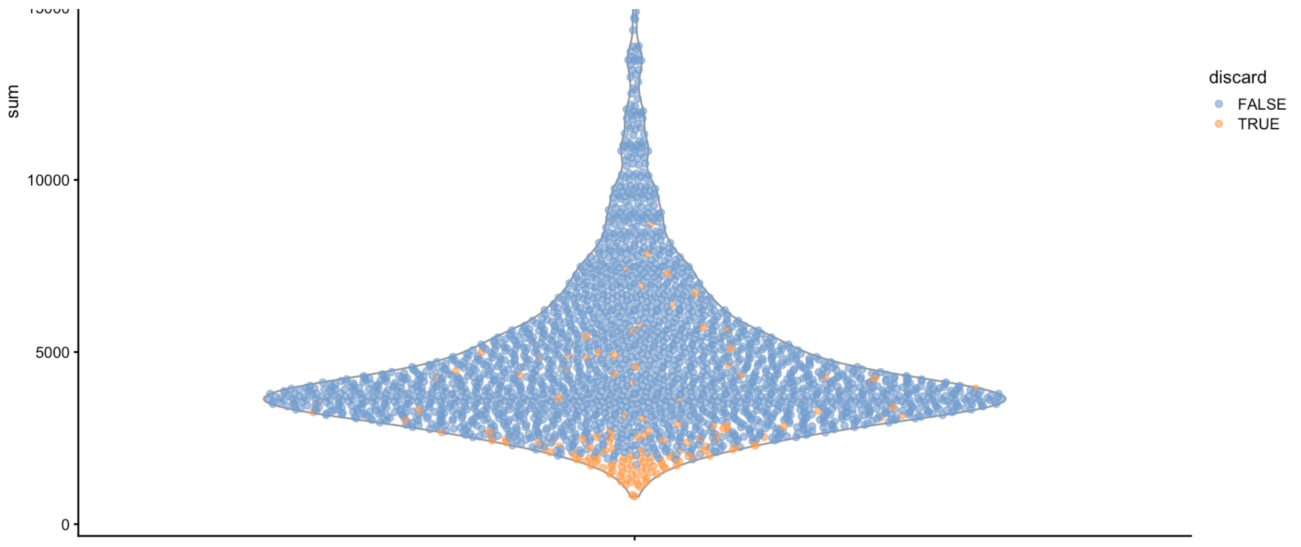


HIDE

```
# Plot the number of detected genes
plotColData(iLN, y = "detected", colour_by = "discard")
```

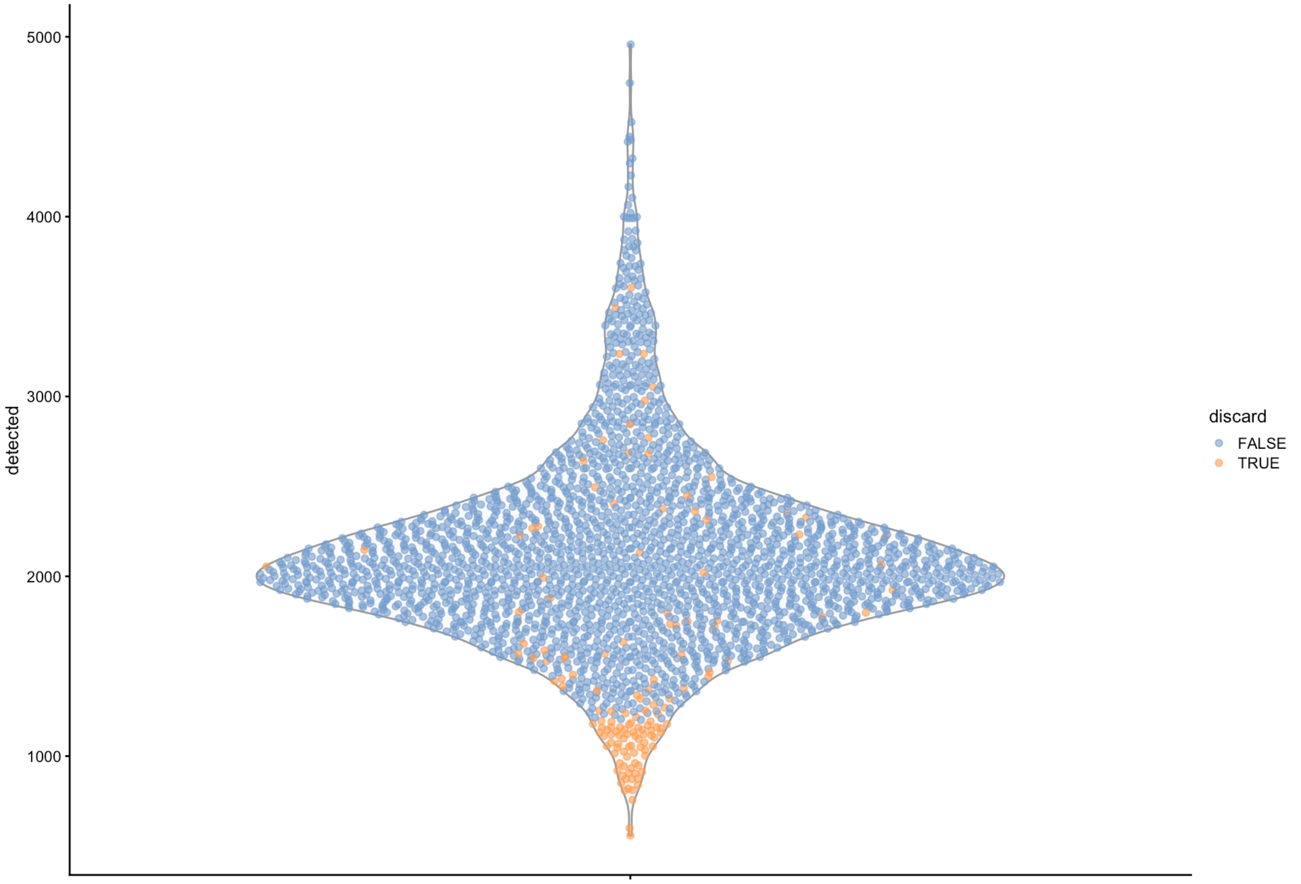


HIDE

```
# Plot mitochondrial RNA percentage against library size
plotColData(iLN,
            x = "sum",
            y = "subsets_Mito_percent",
            colour_by = "discard") +
  labs(x = "Sum of all counts (library size)",
       y = "Percent mitochondrial genes")
```

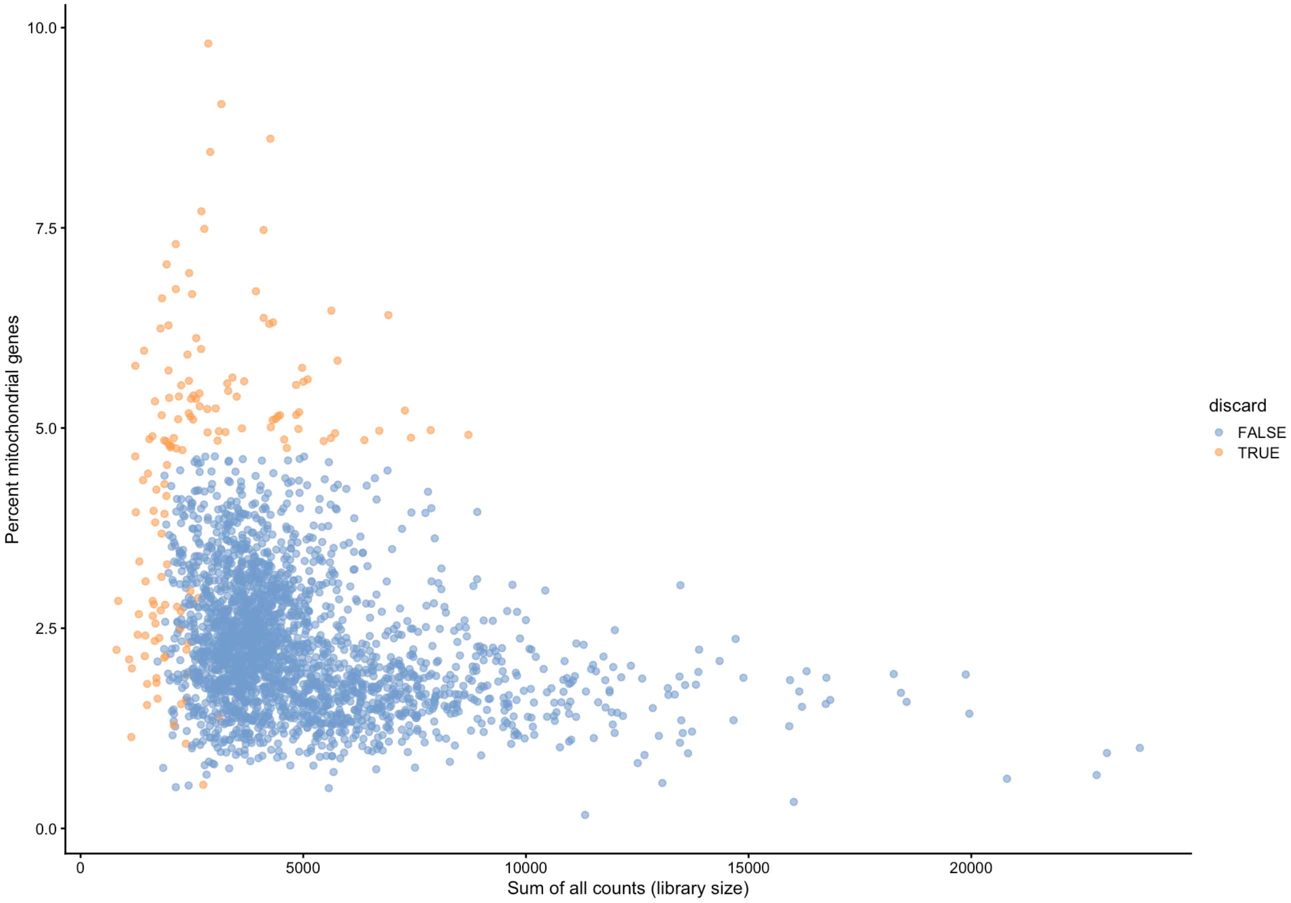


HIDE

```
# Plot library size against number of detected genes
plotColData(iLN,
            x = "detected",
            y = "sum",
            colour_by = "discard") +
  labs(x = "Number of detected genes",
       y = "Sum of all counts (library size)")
```

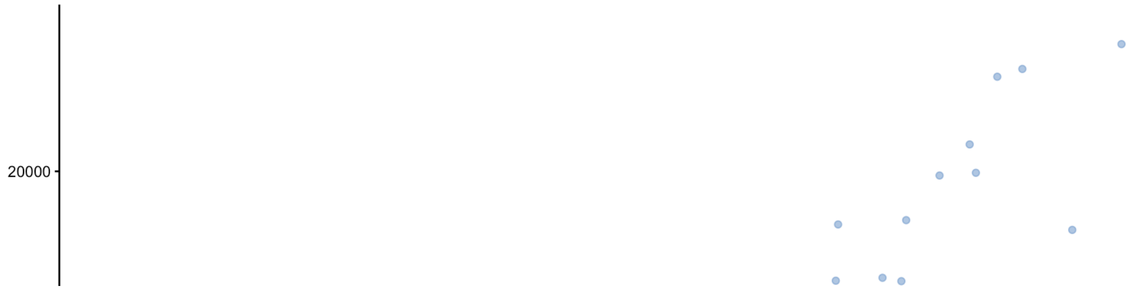

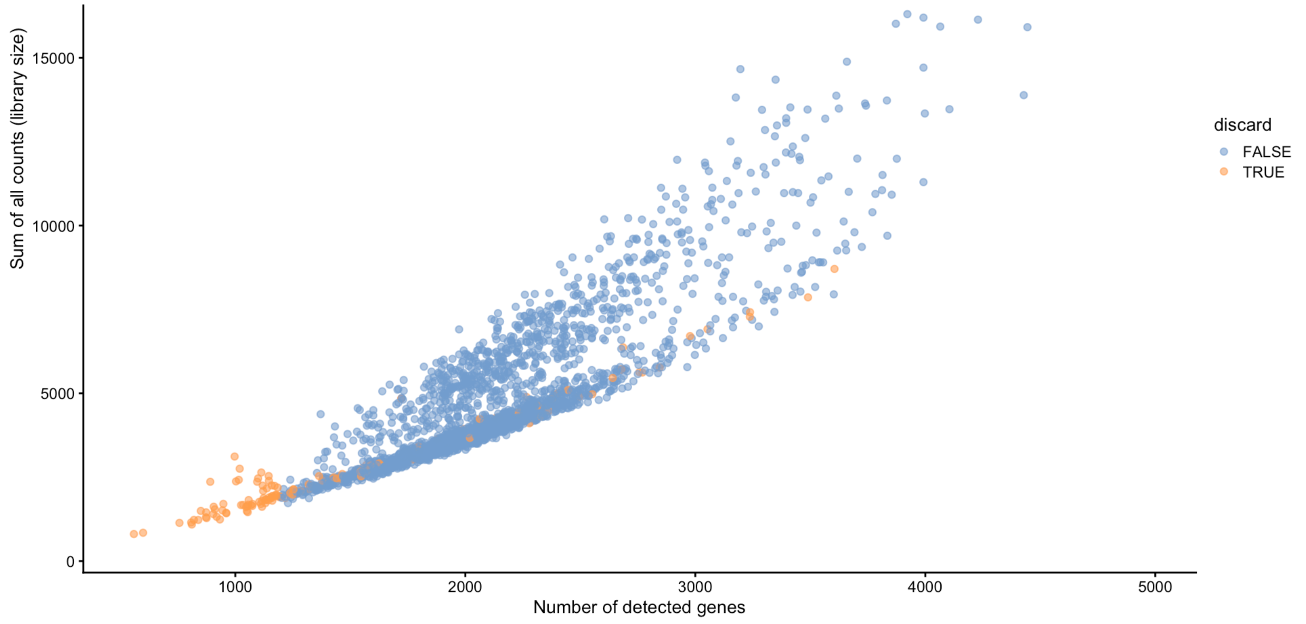


HIDE

```
# Assign the data back to our object
sceRNA$iLN <- iLN
# As this report is only for exploratory analyses we do not filter out any cells
# Otherwise you could do
# sceRNA$iLN <- iLN[, !discard]
```

# 4.2 mLN

HIDE

```
mLN <- sceRNA$mLN

rowData(mLN)$gene_name <- rownames(mLN)
rowData(mLN)$location <- chr.loc
mLN <- addPerFeatureQC(mLN)

rowData(mLN)
```

```
## DataFrame with 32285 rows and 4 columns
##              gene_name    location      mean  detected
##            <character> <character> <numeric> <numeric>
## Xkr4              Xkr4           1         0         0
## Gm1992          Gm1992           1         0         0
## Gm19938        Gm19938           1         0         0
## Gm37381        Gm37381           1         0         0
## Rp1                Rp1           1         0         0
## ...                ...         ...       ...       ...
## AC124606.1  AC124606.1  JH584299.1  0.000000    0.0000
## AC133095.2  AC133095.2  JH584299.1  0.000000    0.0000
## AC133095.1  AC133095.1  JH584299.1  0.000000    0.0000
## AC234645.1  AC234645.1  JH584303.1  0.000000    0.0000
## AC149090.1  AC149090.1  JH584304.1  0.569164   38.3587
```

HIDE

```
mLN <- addPerCellQC(mLN, subsets = list(Mito = is.mito))
qcstats <- perCellQCMetrics(mLN, subsets = list(Mito = is.mito))
filtered <-
  quickPerCellQC(qcstats, percent_subsets = "subsets_Mito_percent")
filtered
```

```
## DataFrame with 3217 rows and 4 columns
##          low_lib_size   low_n_features high_subsets_Mito_percent   discard
##      <outlier.filter> <outlier.filter>          <outlier.filter> <logical>
## 1               FALSE            FALSE                     FALSE     FALSE
## 2               FALSE            FALSE                     FALSE     FALSE
## 3               FALSE            FALSE                     FALSE     FALSE
## 4               FALSE            FALSE                     FALSE     FALSE
## 5               FALSE            FALSE                      TRUE      TRUE
## ...               ...              ...                       ...       ...
## 3213            FALSE            FALSE                     FALSE     FALSE
## 3214            FALSE            FALSE                     FALSE     FALSE
## 3215            FALSE            FALSE                     FALSE     FALSE
## 3216            FALSE            FALSE                     FALSE     FALSE
## 3217            FALSE            FALSE                     FALSE     FALSE
```

HIDE

```
colSums(as.data.frame(filtered))
```

```
##              low_lib_size            low_n_features high_subsets_Mito_percent
##                        12                        59                       100
##                   discard
##                       147
```

HIDE

```
table(filtered$low_n_features, filtered$high_subsets_Mito_percent)
```

```
##
##          FALSE TRUE
##    FALSE  3070   88
##    TRUE     47   12
```

HIDE

```
mLN$discard <- filtered$discard
plotColData(mLN, y = "subsets_Mito_percent", colour_by = "discard")
```

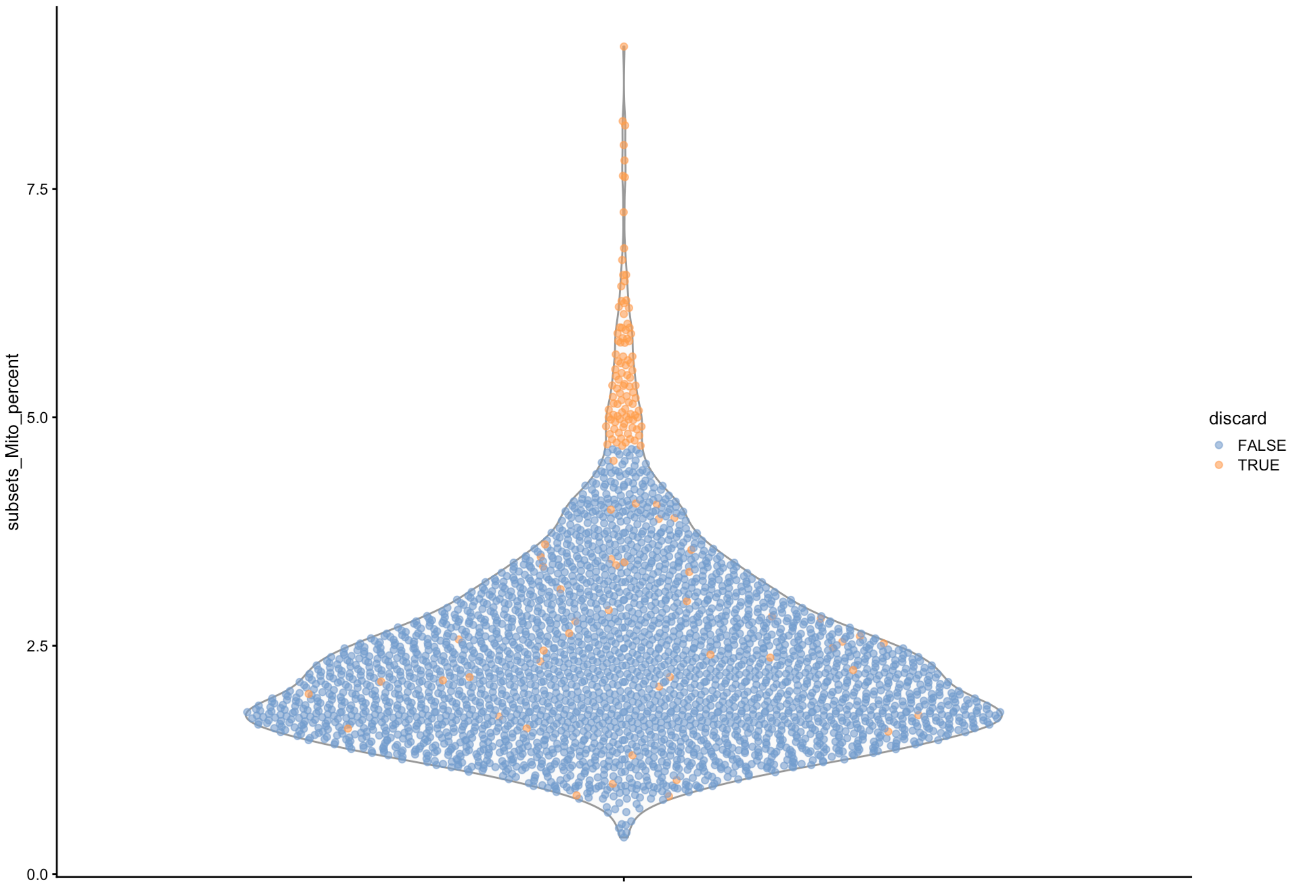


HIDE

```
plotColData(mLN, y = "sum", colour_by = "discard")
```

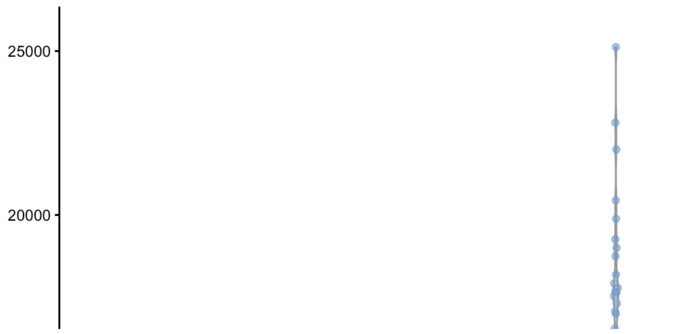

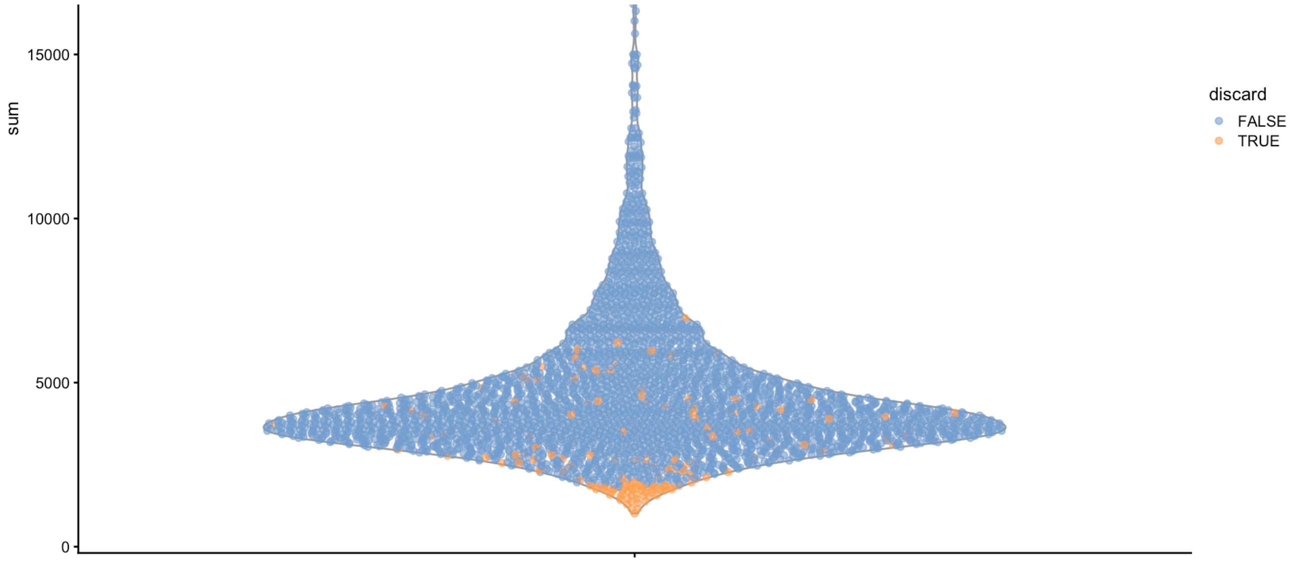


HIDE

```
plotColData(mLN, y = "detected", colour_by = "discard")
```

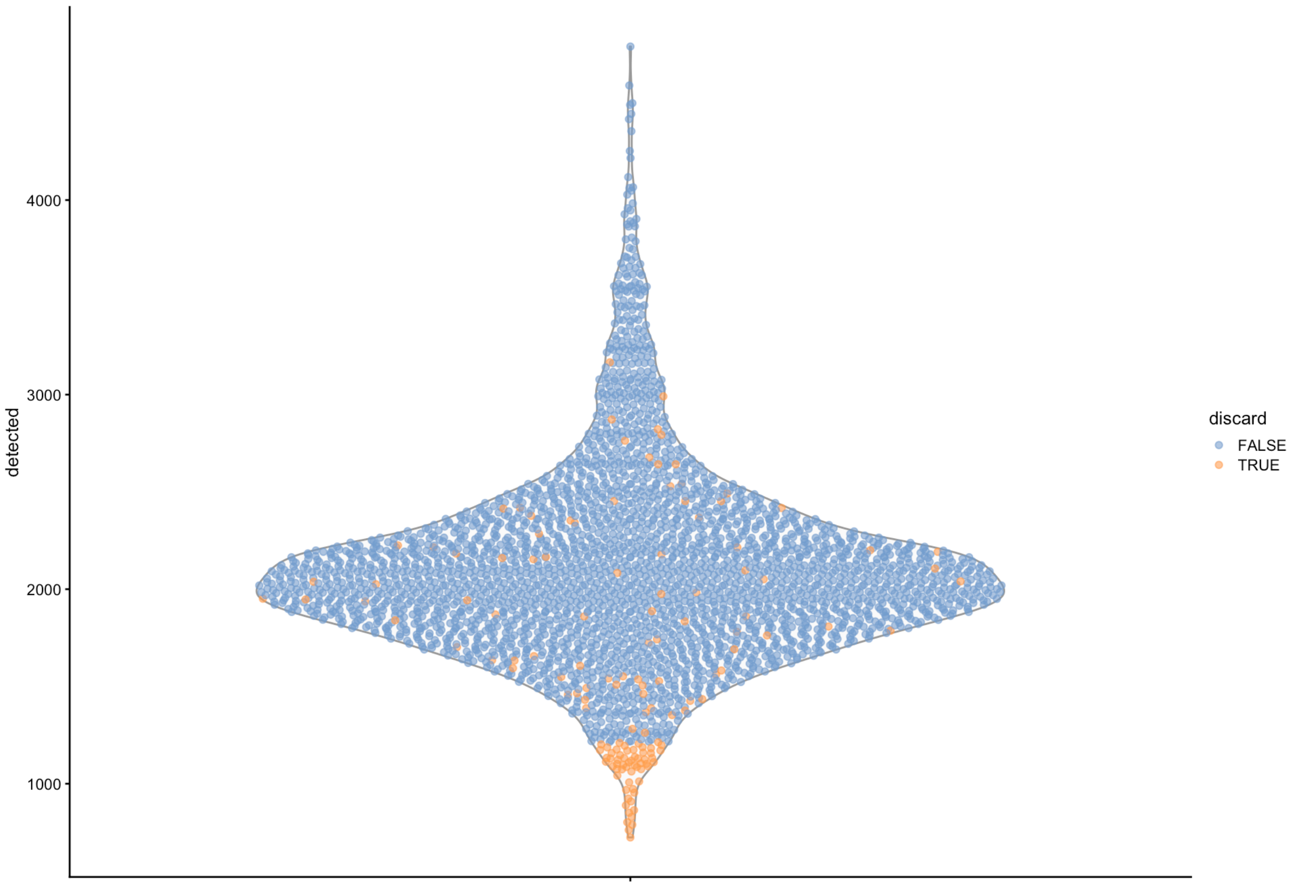


HIDE

```
plotColData(mLN,
            x = "sum",
            y = "subsets_Mito_percent",
            colour_by = "discard") +
  labs(x = "Sum of all counts (library size)",
       y = "Percent mitochondrial genes")
```

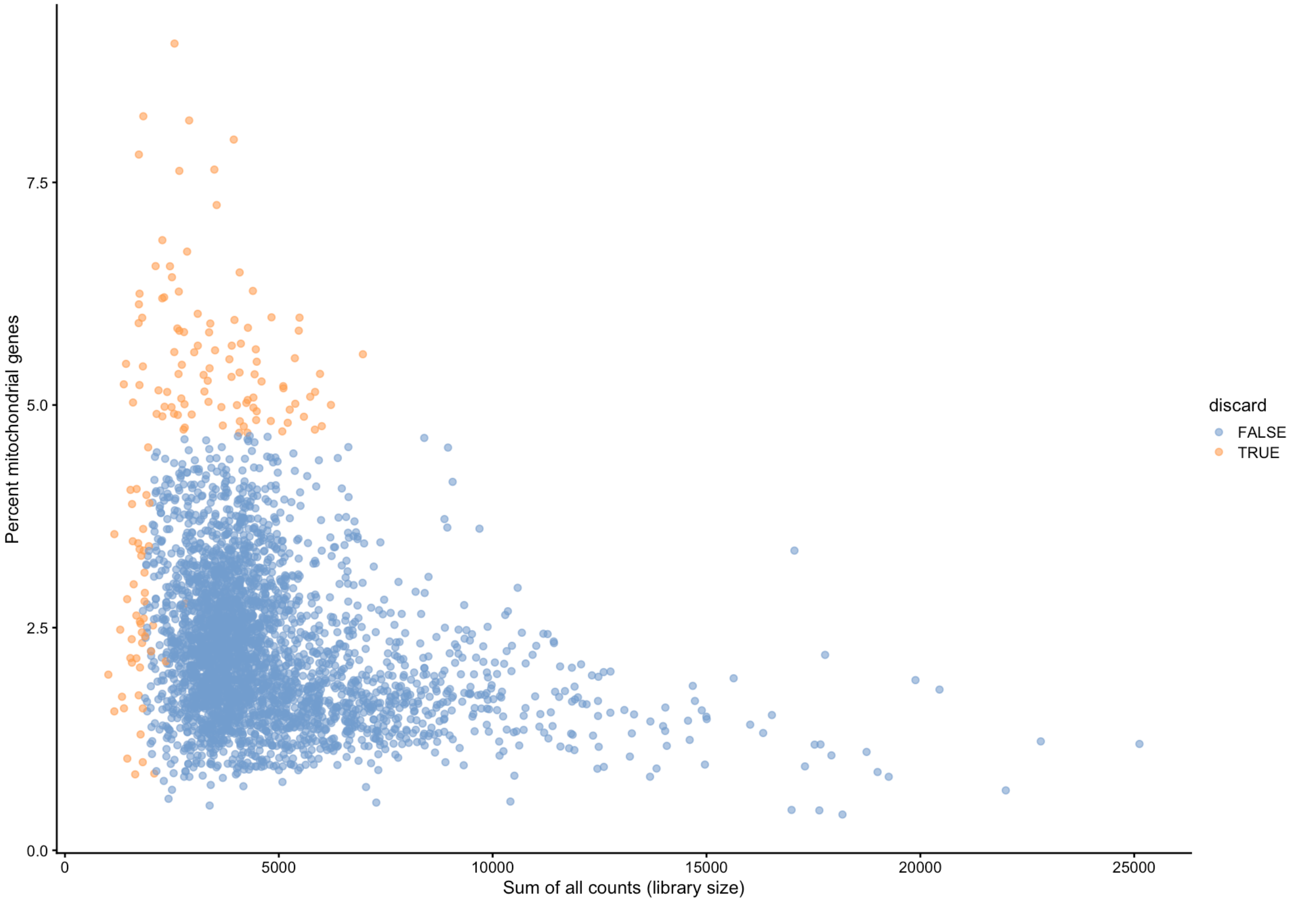


HIDE

```
plotColData(mLN,
            x = "detected",
            y = "sum",
            colour_by = "discard") +
  labs(x = "Number of detected genes",
       y = "Sum of all counts (library size)")
```

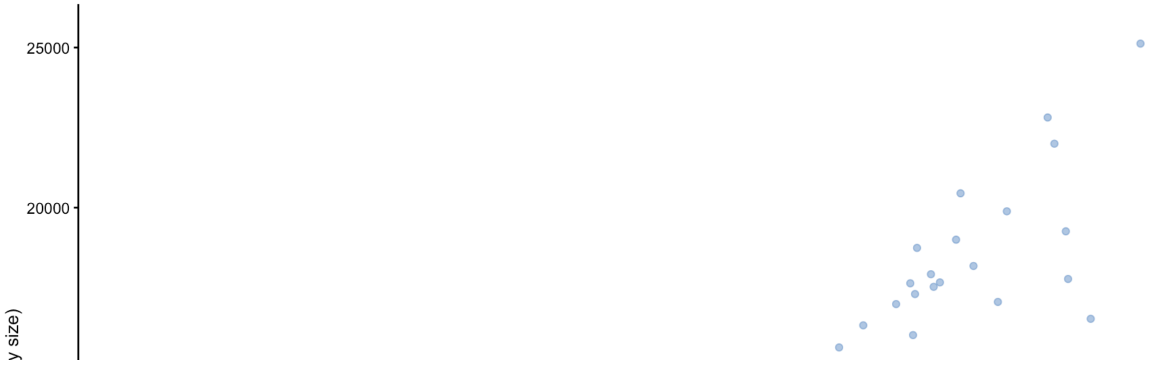

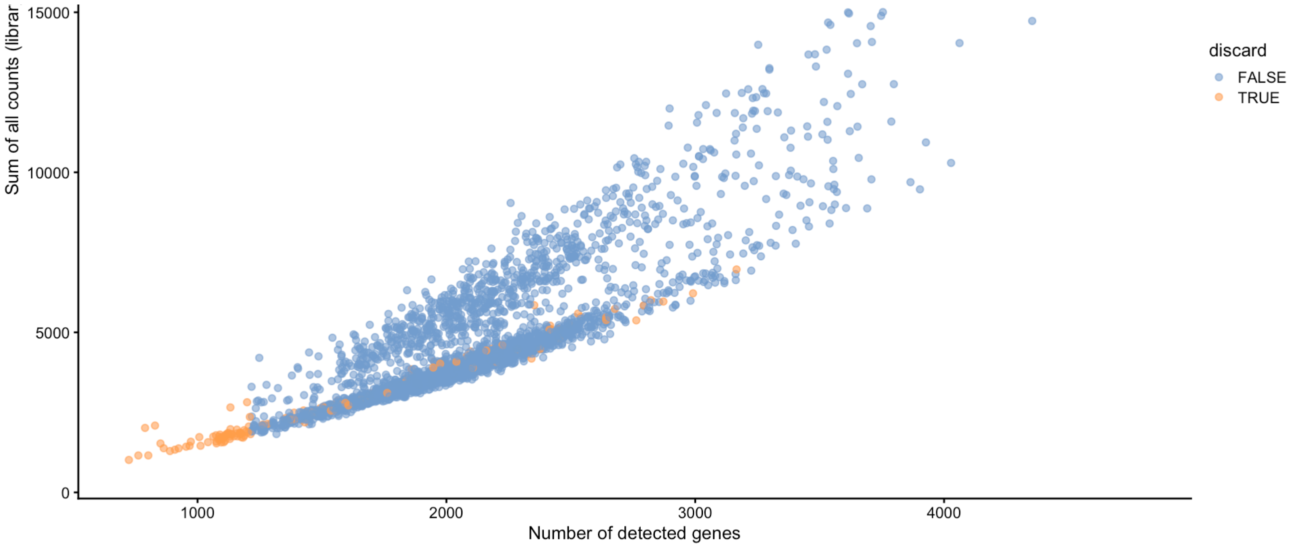


HIDE

```
sceRNA$mLN <- mLN

# As this report is only for exploratory analyses we do not filter out any cells
# Otherwise you could do
# sceRNA$mLN <- mLN[, !discard]
```

# 4.3 skin

HIDE

```
skin <- sceRNA$skin

rowData(skin)$gene_name <- rownames(skin)
rowData(skin)$location <- chr.loc
skin <- addPerFeatureQC(skin)

rowData(skin)
```

```
## DataFrame with 32285 rows and 4 columns
##              gene_name    location      mean  detected
##            <character> <character> <numeric> <numeric>
## Xkr4              Xkr4           1         0         0
## Gm1992          Gm1992           1         0         0
## Gm19938        Gm19938           1         0         0
## Gm37381        Gm37381           1         0         0
## Rp1                Rp1           1         0         0
## ...                ...         ...       ...       ...
## AC124606.1  AC124606.1  JH584299.1  0.000000     0.000
## AC133095.2  AC133095.2  JH584299.1  0.000000     0.000
## AC133095.1  AC133095.1  JH584299.1  0.000000     0.000
## AC234645.1  AC234645.1  JH584303.1  0.000000     0.000
## AC149090.1  AC149090.1  JH584304.1  0.574317    38.726
```

HIDE

```
skin <- addPerCellQC(skin, subsets = list(Mito = is.mito))
qcstats <- perCellQCMetrics(skin, subsets = list(Mito = is.mito))
filtered <-
  quickPerCellQC(qcstats, percent_subsets = "subsets_Mito_percent")
filtered
```

```
## DataFrame with 989 rows and 4 columns
##         low_lib_size   low_n_features high_subsets_Mito_percent   discard
##     <outlier.filter> <outlier.filter>          <outlier.filter> <logical>
## 1              FALSE            FALSE                     FALSE     FALSE
## 2              FALSE            FALSE                      TRUE      TRUE
## 3              FALSE            FALSE                     FALSE     FALSE
## 4              FALSE             TRUE                     FALSE      TRUE
## 5              FALSE            FALSE                     FALSE     FALSE
## ...              ...              ...                       ...       ...
## 985            FALSE            FALSE                     FALSE     FALSE
## 986            FALSE            FALSE                     FALSE     FALSE
## 987            FALSE            FALSE                     FALSE     FALSE
## 988            FALSE            FALSE                     FALSE     FALSE
## 989            FALSE            FALSE                     FALSE     FALSE
```

HIDE

```
colSums(as.data.frame(filtered))
```

```
##              low_lib_size            low_n_features high_subsets_Mito_percent
##                        16                        30                        78
##                   discard
##                        95
```

HIDE

```
table(filtered$low_n_features, filtered$high_subsets_Mito_percent)
```

```
##
##         FALSE TRUE
##   FALSE   894   65
##   TRUE     17   13
```

HIDE

```
skin$discard <- filtered$discard
plotColData(skin, y = "subsets_Mito_percent", colour_by = "discard")
```

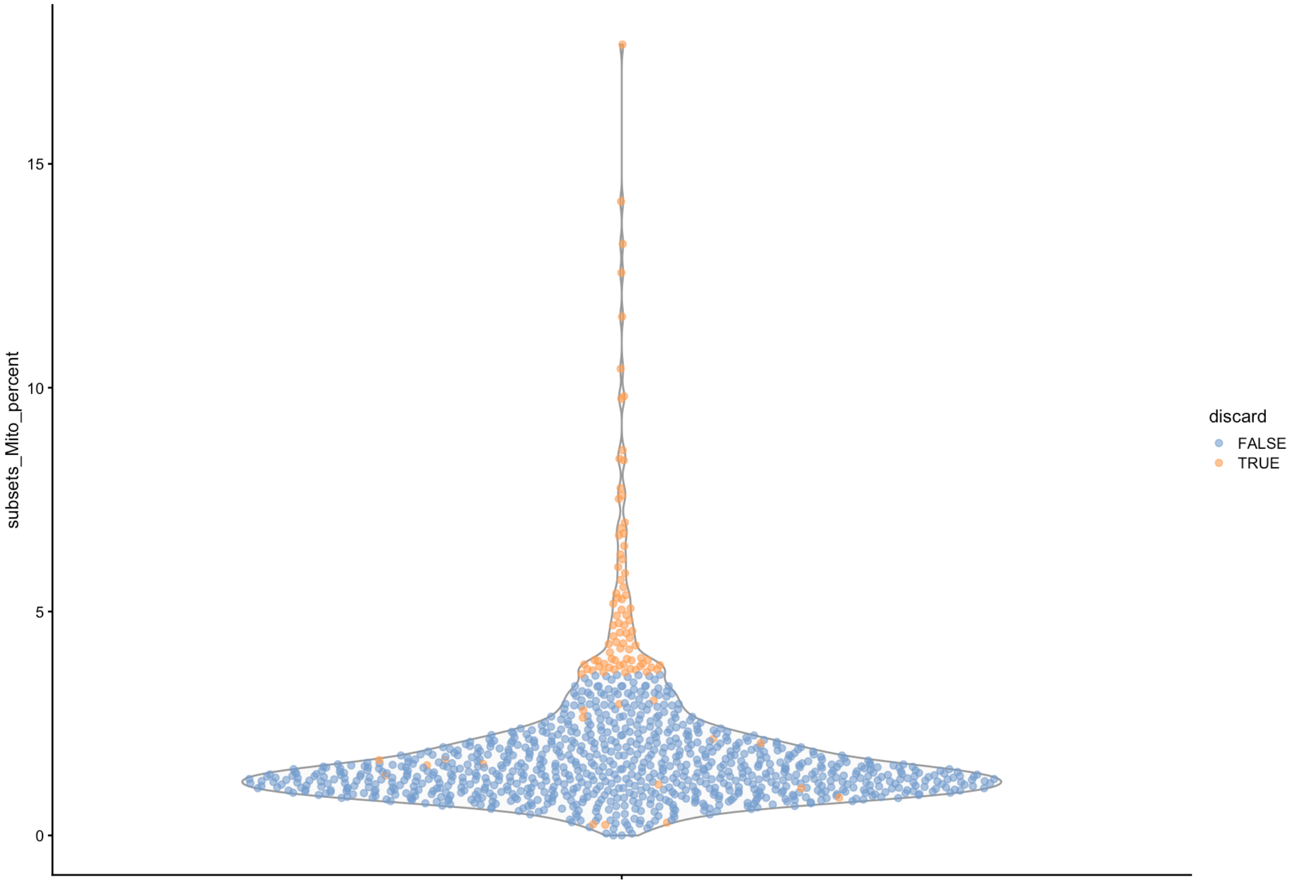


HIDE

```
plotColData(skin, y = "sum", colour_by = "discard")
```

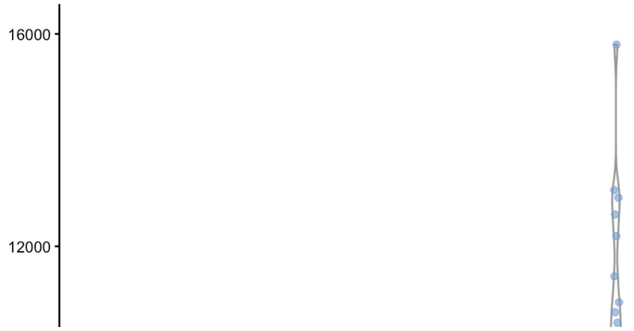

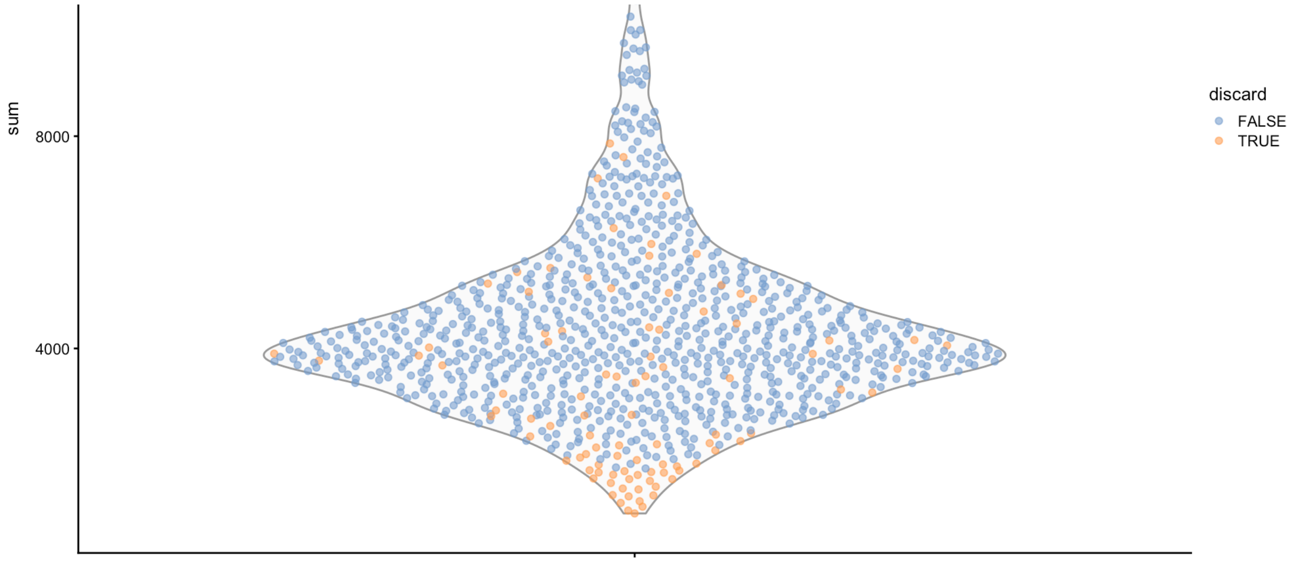


HIDE

```
plotColData(skin, y = "detected", colour_by = "discard")
```

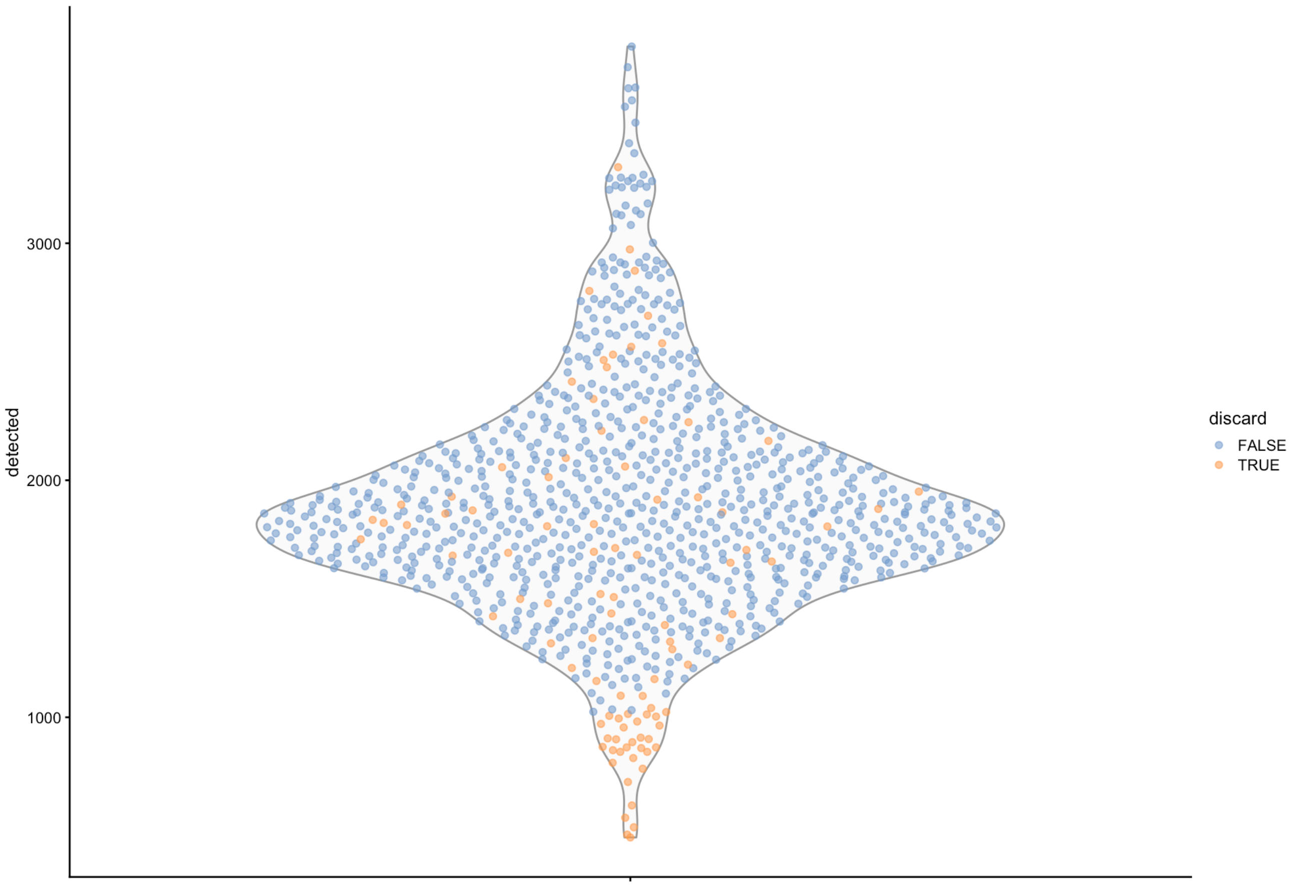


HIDE

```
plotColData(skin,
            x = "sum",
            y = "subsets_Mito_percent",
            colour_by = "discard") +
  labs(x = "Sum of all counts (library size)",
       y = "Percent mitochondrial genes")
```

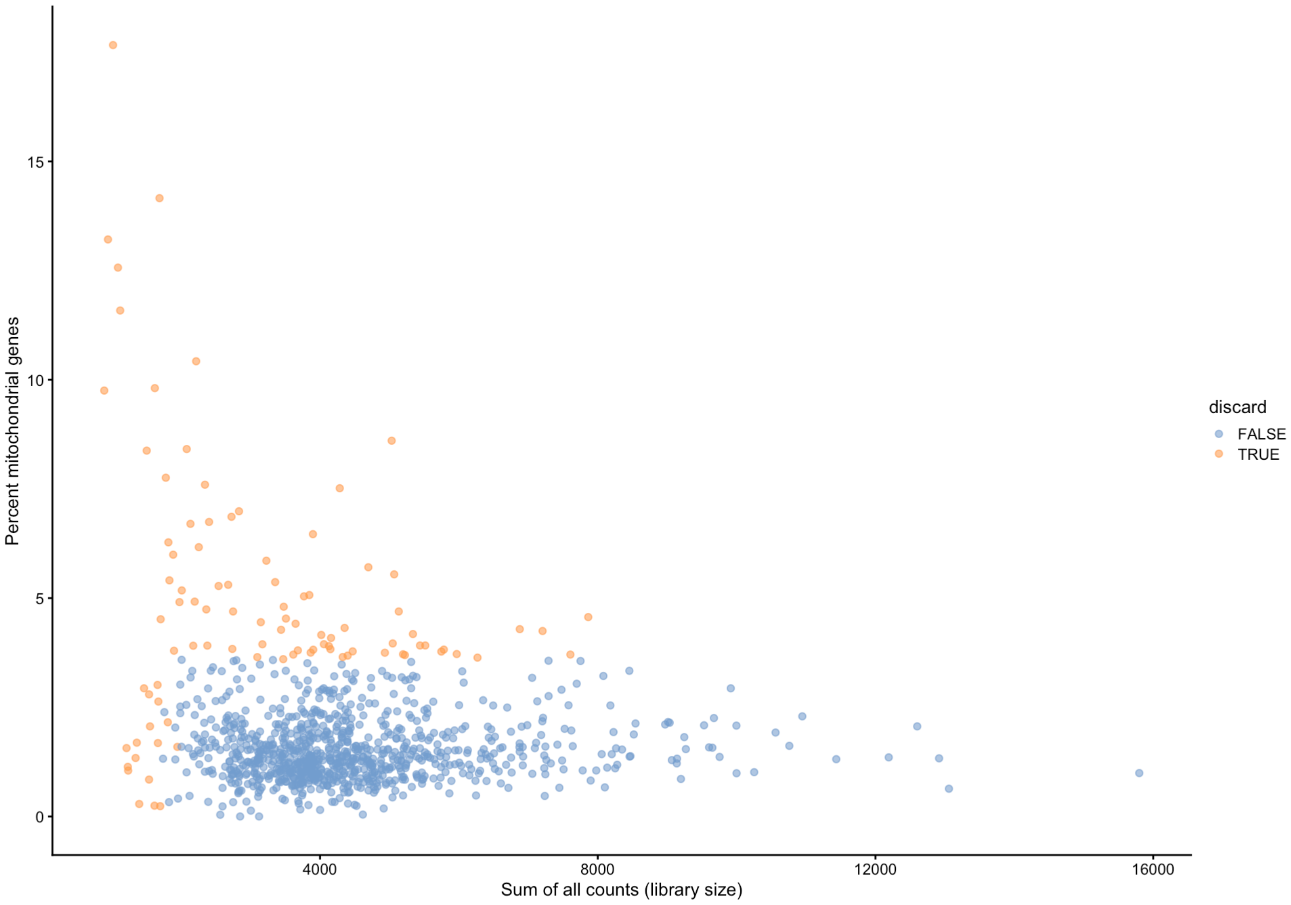


HIDE

```
plotColData(skin,
            x = "detected",
            y = "sum",
            colour_by = "discard") +
  labs(x = "Number of detected genes",
       y = "Sum of all counts (library size)")
```

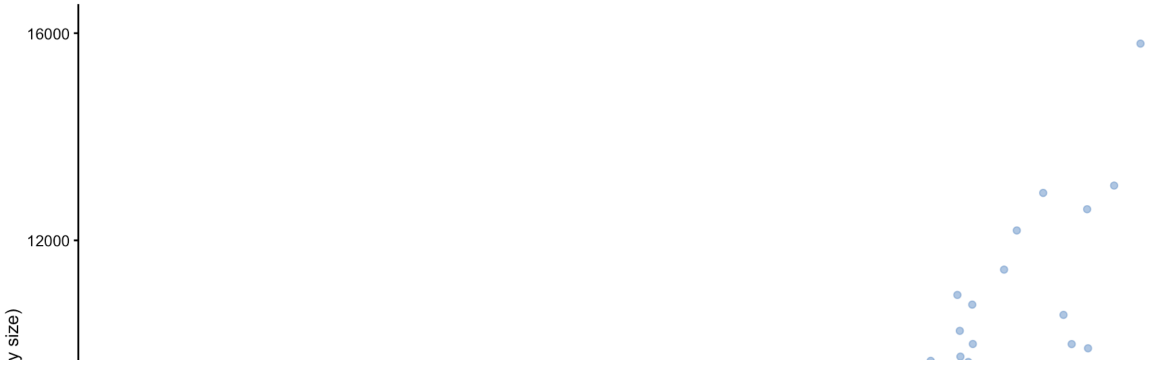

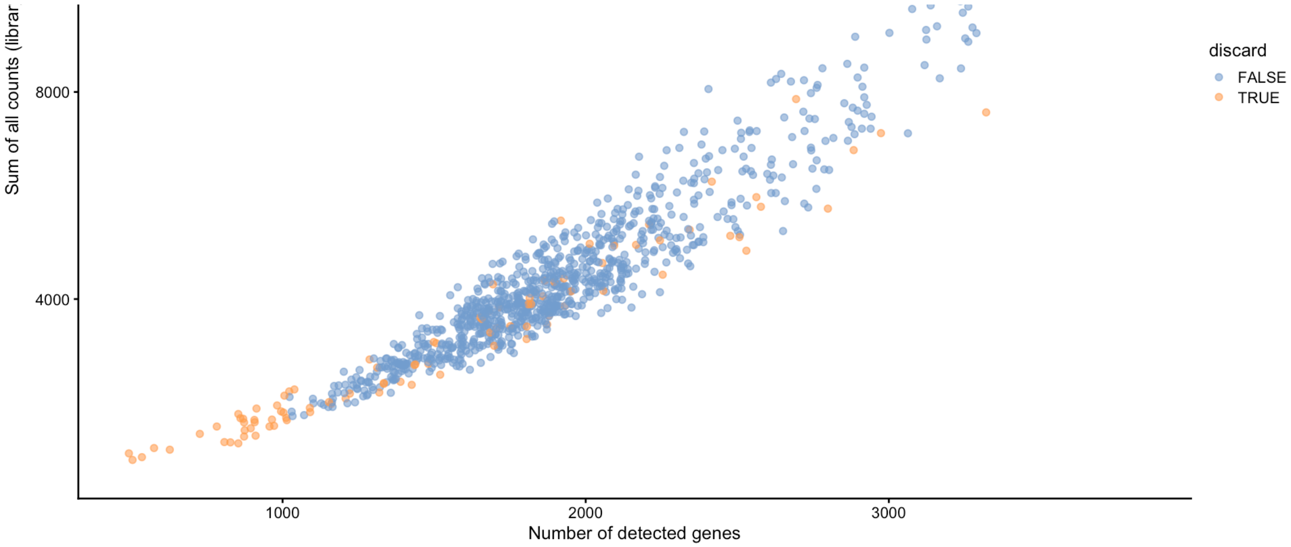


HIDE

```
sceRNA$skin <- skin

# As this report is only for exploratory analyses we do not filter out any cells
# Otherwise you could do
# sceRNA$skin <- skin[, !discard]
```

# 4.4 spleen

HIDE

```
spleen <- sceRNA$spleen

rowData(spleen)$gene_name <- rownames(spleen)
rowData(spleen)$location <- chr.loc
spleen <- addPerFeatureQC(spleen)

rowData(spleen)
```

```
## DataFrame with 32285 rows and 4 columns
##              gene_name    location        mean  detected
##            <character> <character>   <numeric> <numeric>
## Xkr4              Xkr4           1 0.000293083 0.0293083
## Gm1992          Gm1992           1 0.000000000 0.0000000
## Gm19938        Gm19938           1 0.000000000 0.0000000
## Gm37381        Gm37381           1 0.000000000 0.0000000
## Rp1                Rp1           1 0.000586166 0.0586166
## ...                ...         ...         ...       ...
## AC124606.1  AC124606.1  JH584299.1    0.000000    0.0000
## AC133095.2  AC133095.2  JH584299.1    0.000000    0.0000
## AC133095.1  AC133095.1  JH584299.1    0.000000    0.0000
## AC234645.1  AC234645.1  JH584303.1    0.000000    0.0000
## AC149090.1  AC149090.1  JH584304.1    0.652989   41.6764
```

HIDE

```
spleen <- addPerCellQC(spleen, subsets = list(Mito = is.mito))
qcstats <- perCellQCMetrics(spleen, subsets = list(Mito = is.mito))
filtered <-
  quickPerCellQC(qcstats, percent_subsets = "subsets_Mito_percent")
filtered
```

```
## DataFrame with 3412 rows and 4 columns
##          low_lib_size   low_n_features high_subsets_Mito_percent   discard
##      <outlier.filter> <outlier.filter>          <outlier.filter> <logical>
## 1               FALSE            FALSE                     FALSE     FALSE
## 2               FALSE            FALSE                     FALSE     FALSE
## 3               FALSE            FALSE                     FALSE     FALSE
## 4               FALSE            FALSE                     FALSE     FALSE
## 5               FALSE            FALSE                     FALSE     FALSE
## ...               ...              ...                       ...       ...
## 3408            FALSE            FALSE                     FALSE     FALSE
## 3409            FALSE            FALSE                     FALSE     FALSE
## 3410            FALSE            FALSE                     FALSE     FALSE
## 3411            FALSE            FALSE                     FALSE     FALSE
## 3412            FALSE            FALSE                     FALSE     FALSE
```

HIDE

```
colSums(as.data.frame(filtered))
```

```
##              low_lib_size            low_n_features high_subsets_Mito_percent
##                        33                        72                       125
##                   discard
##                       184
```

HIDE

```
table(filtered$low_n_features, filtered$high_subsets_Mito_percent)
```

```
##
##         FALSE TRUE
##   FALSE  3228  112
##   TRUE     59   13
```

HIDE

```
spleen$discard <- filtered$discard
plotColData(spleen, y = "subsets_Mito_percent", colour_by = "discard")
```

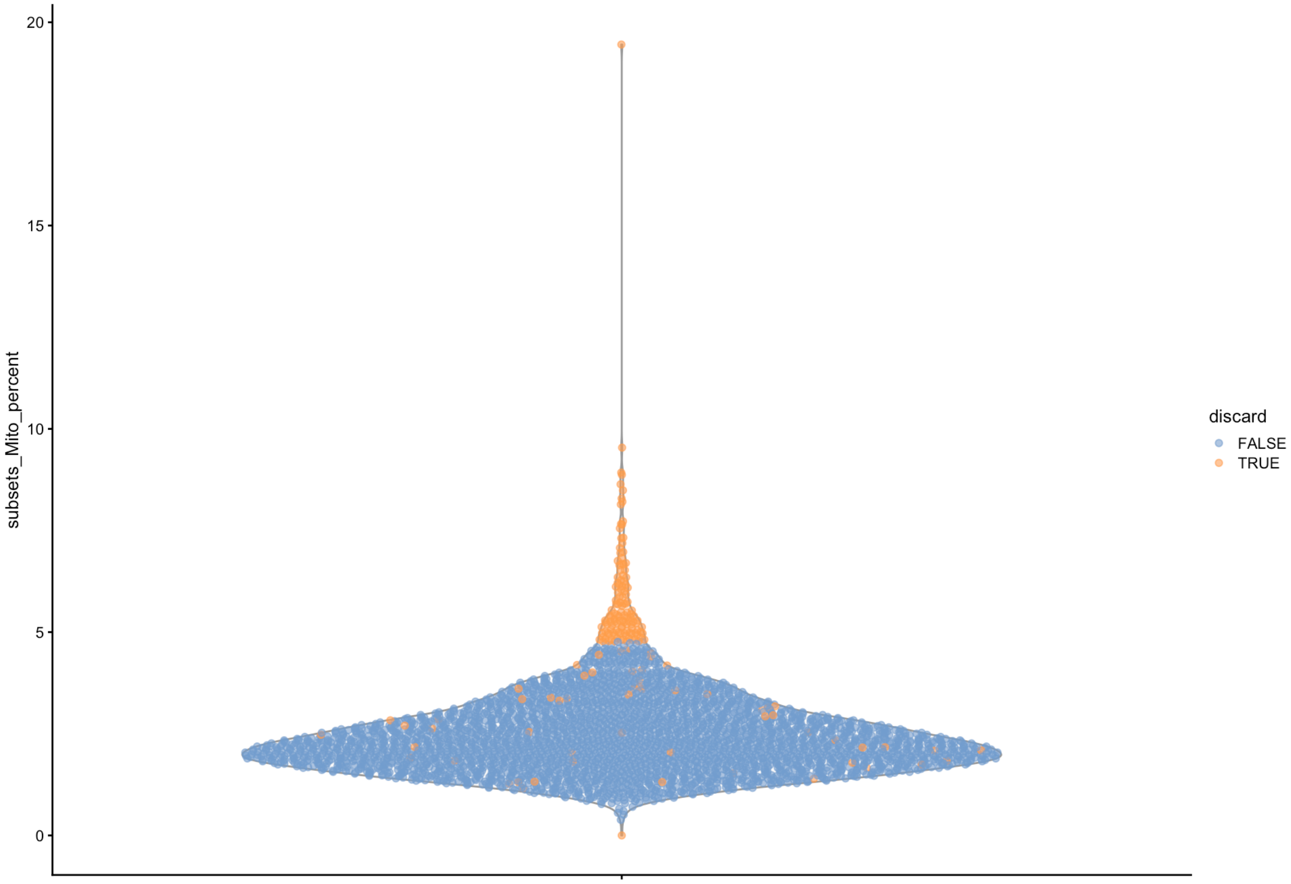


HIDE

```
plotColData(spleen, y = "sum", colour_by = "discard")
```

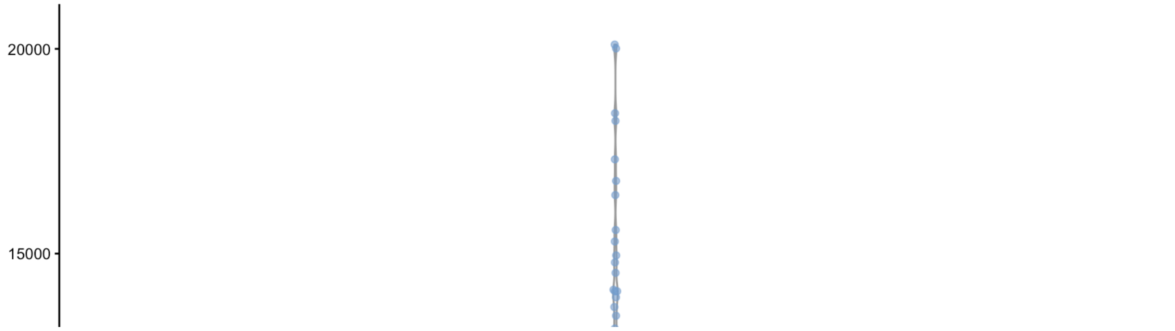

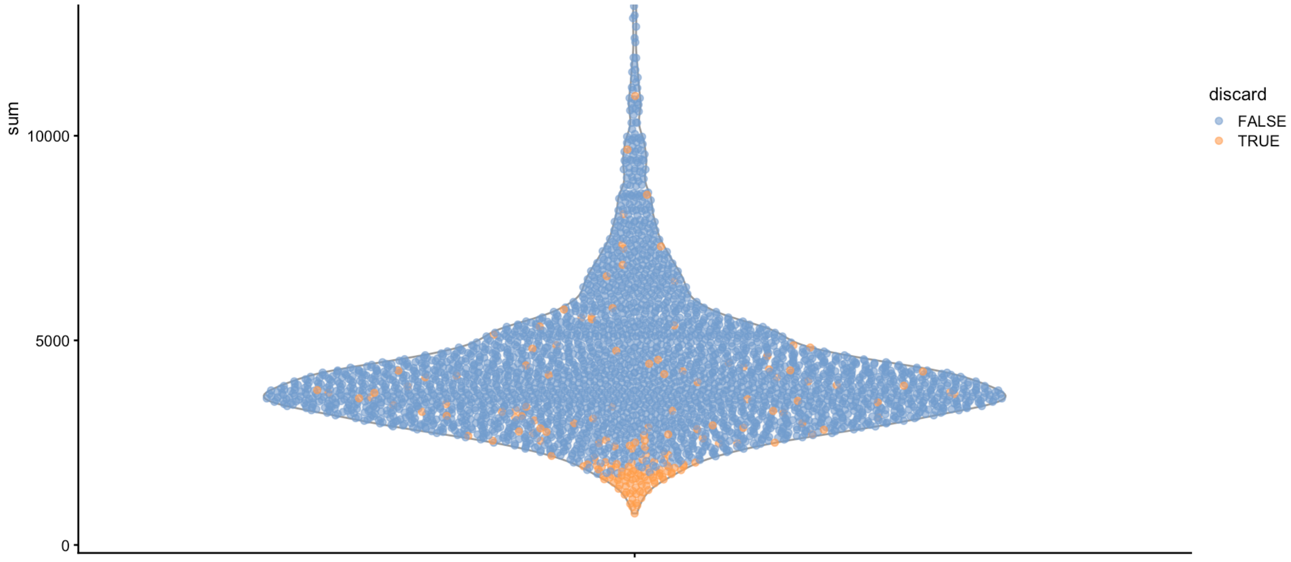


HIDE

```
plotColData(spleen, y = "detected", colour_by = "discard")
```

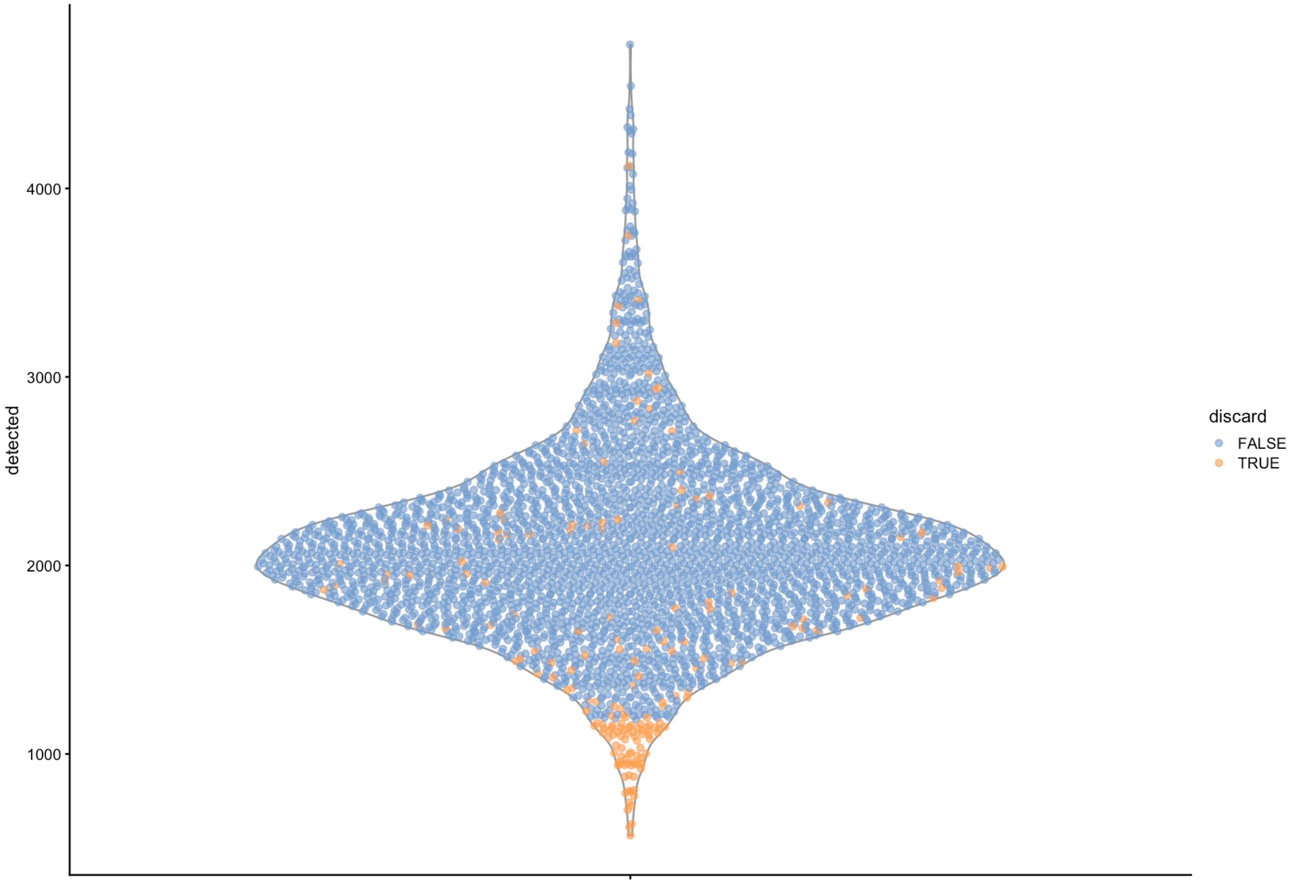


HIDE

```
plotColData(spleen,
            x = "sum",
            y = "subsets_Mito_percent",
            colour_by = "discard") +
  labs(x = "Sum of all counts (library size)",
       y = "Percent mitochondrial genes")
```

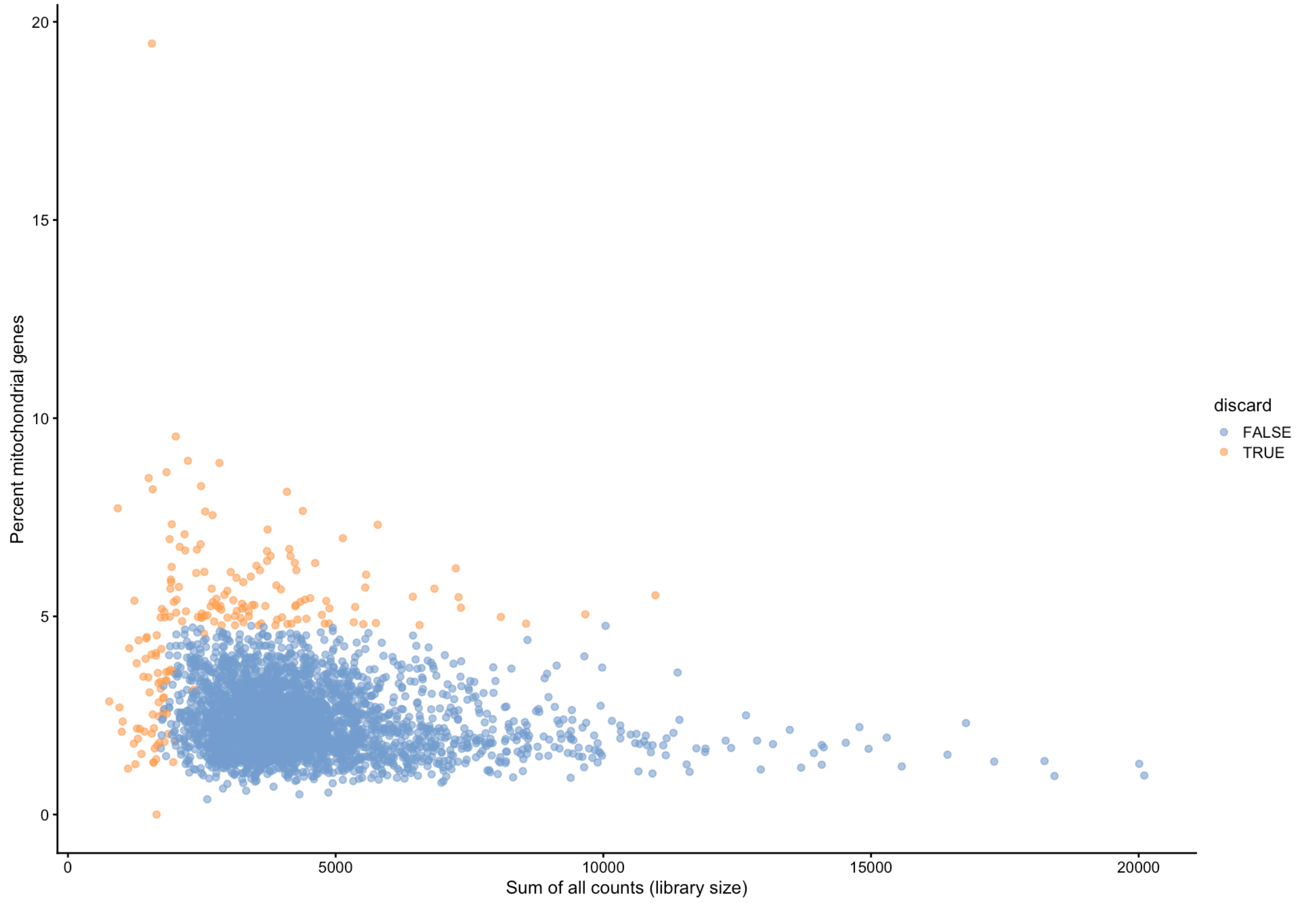


HIDE

```
plotColData(spleen,
            x = "detected",
            y = "sum",
            colour_by = "discard") +
  labs(x = "Number of detected genes",
       y = "Sum of all counts (library size)")
```

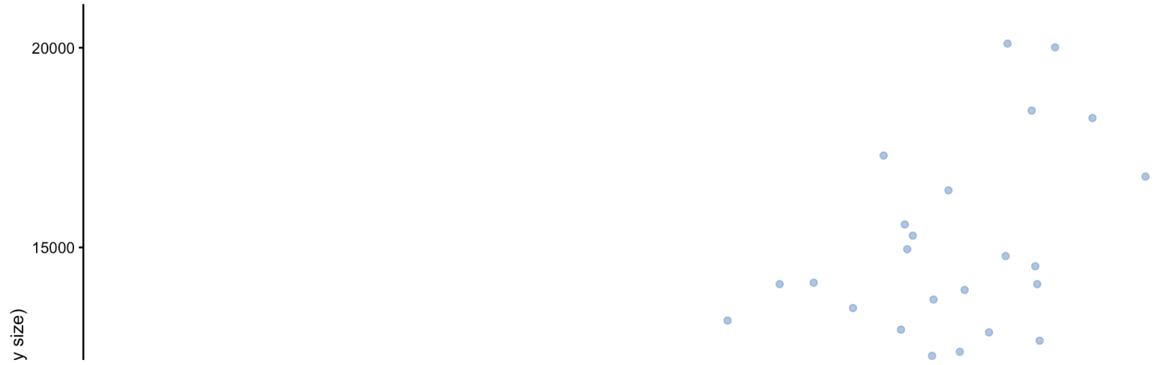

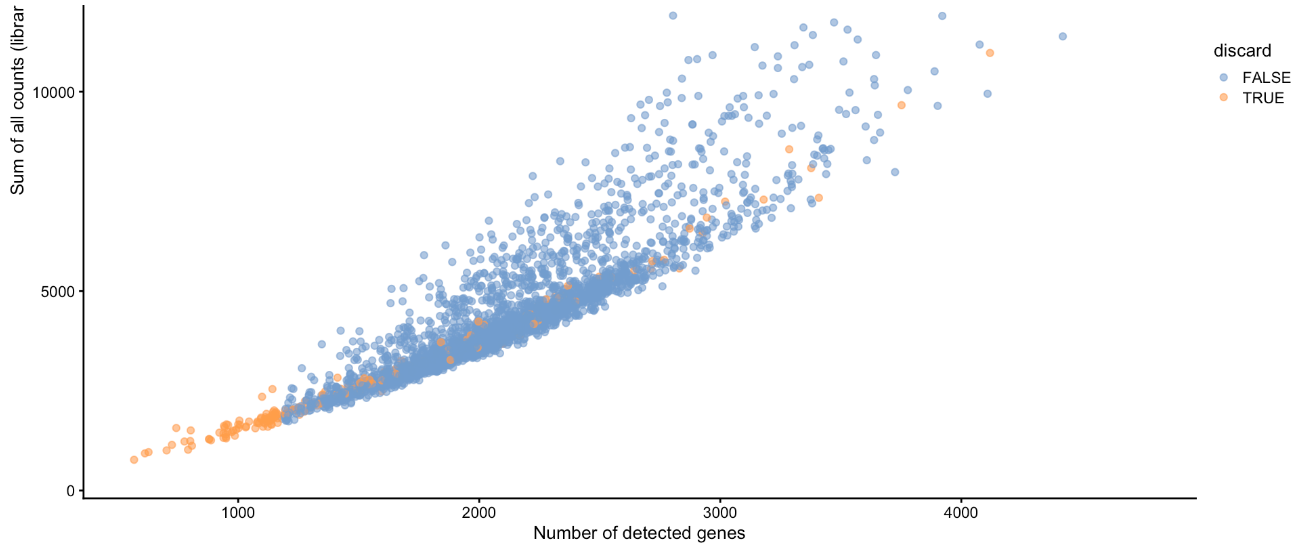


HIDE

```
sceRNA$spleen <- spleen

# As this report is only for exploratory analyses we do not filter out any cells
# Otherwise you could do
# sceRNA$spleen <- spleen[, !discard]
```

We can also plot the different quality control metrics of the individual tissues together. For this, we preliminary have to merge all `SingleCellExperiment` objects into one before we can proceed with the plotting. As this object is only preliminary for plotting purposes, we make sure to delete it afterwards.

HIDE

```
# Subset the rowData of each tissue to make it match
rowData(iLN) <-
  rowData(iLN)[, c("gene_name", "location")]
rowData(mLN) <-
  rowData(mLN)[, c("gene_name", "location")]
rowData(skin) <-
  rowData(skin)[, c("gene_name", "location")]
rowData(spleen) <-
  rowData(spleen)[, c("gene_name", "location")]

# Merge all sce together
prelim_sce_merged <- cbind(iLN,
                           mLN,
                           skin,
                           spleen)

# Plot all the QC metrics of each tissue together
gridExtra::grid.arrange(
  plotColData(
    prelim_sce_merged,
    x = "tissue",
    y = "sum",
    colour_by = "discard",
    other_fields = "tissue"
  )  + ggtitle("Sum of all counts (library size)"),
  plotColData(
    prelim_sce_merged,
    x = "tissue",
    y = "detected",
    colour_by = "discard",
    other_fields = "tissue"
  ) +  ggtitle("Number of detected genes"),
  plotColData(
    prelim_sce_merged,
    x = "tissue",
    y = "subsets_Mito_percent",
    colour_by = "discard",
    other_fields = "tissue"
  ) + ggtitle("Percent mitochondrial genes"),
  ncol = 1
)
```

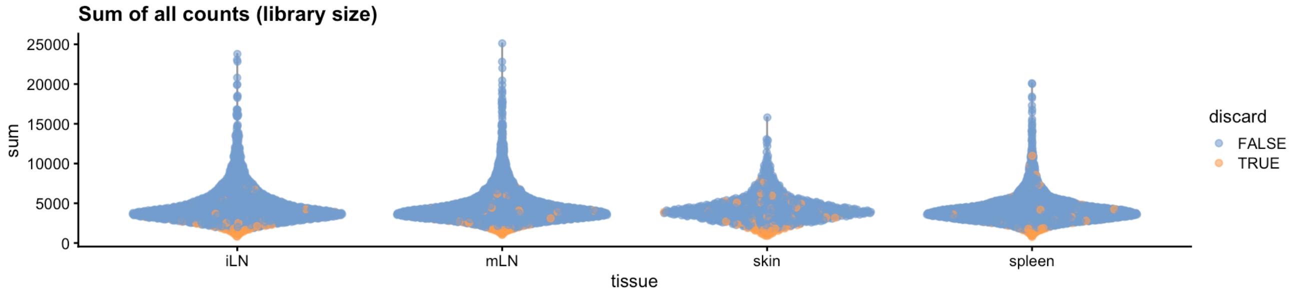


**Number of detected genes**

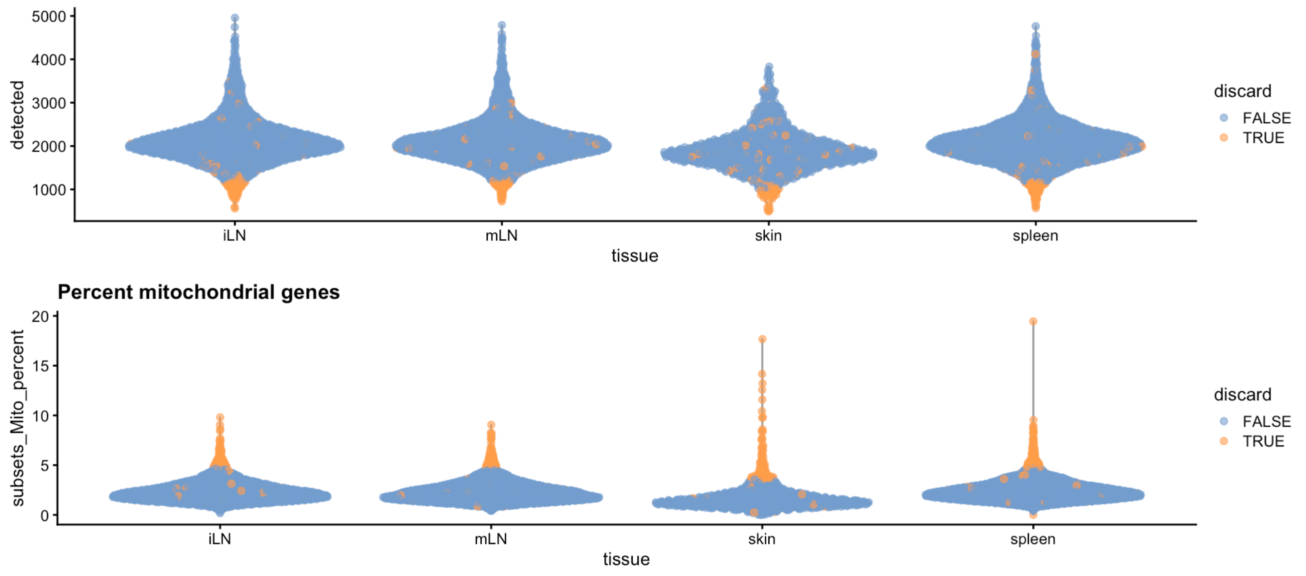


HIDE

```
rm(prelim_sce_merged)
```

# 4.5 Seurat

In the Seurat pipeline, everything from now on will be applied to all samples simultaneously.

The quality control and filtering in the Seurat pipeline operates on similar metrics described above.

First, we calculate the percentage of genes mapping to the mitochondrial genome. Afterwards, we plot all quality metrics as violin plots. We can also plot different metrics against each other as shown above in the `SingleCellExperiment` workflow.

In the Seurat workflow, the thresholds for low quality cells have to be selected by the users. Usually, we flag cells with less than 500 or more than 2500 and more than 5% of mitochondrial DNA as low quality cells and filter those from the data. However, as this workflow is only of exploratorive nature, we again do not filter cells from the data.

In your own data, we strongly advise you to test out differetn filter thresholds and adapt them to the respective data set at hand.

HIDE

```
# Calculate percentage mitochondrial DNA for each cell
seurat_merged[["percent.mt"]] <- PercentageFeatureSet(seurat_merged,
                                                      pattern = "^mt-")

# Plot the percent of mitochondrial DNA, feature number and RNA number side by s
ide
VlnPlot(seurat_merged, features = c("nFeature_RNA", "nCount_RNA", "percent.mt"),
ncol = 3)

# Plot scatter plots of
plot1 <- FeatureScatter(seurat_merged, feature1 = "nCount_RNA", feature2 = "perc
ent.mt")
plot2 <- FeatureScatter(seurat_merged, feature1 = "nCount_RNA", feature2 = "nFea
ture_RNA")
CombinePlots(plots = list(plot1, plot2))

# You could filter out low quality cells with the following command
# seurat_merged <- subset(seurat_merged, subset = nFeature_RNA > 500 & nFeature_
RNA < 2500 & percent.mt < 5)
```

# 5 Doublet detection in the individual samples

Doulets are usually and artificial observation where two or more cells are treated as one. As they do not represent meaningful data, we'd like to identify and filter them from our data.

Doublet detection is best done on the individual tissues as different factors are used to decide whether a cell is considered a doublet or not. These are very sample specific, hence doing doublet prediction of all samples combined could lead to false results.

In our workflow, we use the `scDblFinder()` function form the corresponding package. This function combines the cluster based identification of doublets with the in silico simulation of doublet gene expression profiles.

Afterwards, the doublets can either be removed from the data or marked accordingly. As we are only doing an explorative analysis in this workflow, we decided against filtering cells from the data.

## 5.1 iLN

HIDE

```
iLN <- sceRNA$iLN
# Doublet detection
iLN <- scDblFinder(iLN)
# Print a statistics table
table(iLN$scDblFinder.class)
```

```
##
## singlet doublet
##    2382     127
```

HIDE

```
# Assign the object back to save the information
sceRNA$iLN <- iLN

# Or you can assign the object back without the cells marked as doublets
# sceRNA$iLN <- iLN [, iLN $scDblFinder.class == "singlet"]
```

# 5.2 mLN

HIDE

```
mLN <- sceRNA$mLN
# Doublet detection
mLN <- scDblFinder(mLN)
table(mLN$scDblFinder.class)
```

```
##
## singlet doublet
##    3078     139
```

HIDE

```
sceRNA$mLN <- mLN

# Or you can assign the object back without the cells marked as doublets
# sceRNA$mLN <- mLN[, mLN$scDblFinder.class == "singlet"]
```

# 5.3 skin

HIDE

```
skin <- sceRNA$skin
# Doublet detection
skin <- scDblFinder(skin)
table(skin$scDblFinder.class)
```

```
##
## singlet doublet
##     948      41
```

HIDE

```
sceRNA$skin <- skin

# Or you can assign the object back without the cells marked as doublets
# sceRNA$skin <- skin[, skin$scDblFinder.class == "singlet"]
```

## 5.4 spleen

HIDE

```
spleen <- sceRNA$spleen
# Doublet detection
spleen <- scDblFinder(spleen)
table(spleen$scDblFinder.class)
```

```
##
## singlet doublet
##    3296     116
```

HIDE

```
sceRNA$spleen <- spleen

# Or you can assign the object back without the cells marked as doublets
# sceRNA$spleen <- spleen[, spleen$scDblFinder.class == "singlet"]
```

## 5.5 Seurat

In Seurat there is no specific doublet detection step applied. If you wish to apply doublet detection, you could transform your data to a `SingleCellExperiment` object as shown above, apply doublet detection and transform the data back to a `Seurat` object.

# 6 Per-sample Normalization

In scRNA-seq data, often differences in the sequencing coverage between libraries arise. The cause for these variations is typically technical variation in cDNA capture or PCR amplification efficiency. Since this variability does not depict true biological signal in the data, it can distort the interpretation of expression profiles. In order to prevent the influence of the technical variation on data analysis, the data is normalized.

Usually, normalization is applied to the different batches of the data at hand. The data presented in this paper does not consist of different batches but only of different tissues. However, treating the different tissues as individual batches and normalizing across tissues at this point would be detrimental to downstream analysis steps. Hence, we decided to postpone the across tissue normalization to a later point of the workflow.

Nevertheless, there are intra-sample normalization methods which should be applied at this point in the analysis. One of these normalizations is a log-scaling of the expression values, as implemented in the `logNormCounts` function of the `scran` package. This is beneficial for downstream analysis steps such as dimensionality reduction and clustering, as the expression values become more comparable without having

too extreme values.

HIDE

```
sceRNA <- lapply(sceRNA, logNormCounts)
```

## 6.1 Seurat

In the Seurat pipeline, the normalization is achived by using the `NormalizeData` function from the package. We use the default behavior of the function, which employs a global-scaling normalization that normalizes the gene expression of each cell by the total expression, multiplies 10,000 (scale factor, can be adapted), and log-transforms the result.

HIDE

```
seurat_merged <- NormalizeData(seurat_merged)
```

# 7 Feature Selection

In the next step we want to select a set of genes from the data that captures the useful biological variation in the data while excluding technical noise and uninteresting biological noise.

This feature selection step is important for downstream analyses such as dimensionality reduction and clustering. These methods aim to capture and characterize the heterogenity of the data. Preselecting a subset of the available genes in the input data not only ensures a reduced runtime for the following steps but also aims at capturing the interesting biological variation of the data in order to yield a more meaningful and easier interpretation of the downstream analyses steps.

A common approach in feature selection is to select the most variable genes in the data as these are likely to represent interesting biological variation. For this, we first use the `modelGeneVar` function to model the variation of the individual genes before selecting the top 10% of most variable genes.

The choice of how many highly variable genes to use is usually difficult. If the chosen set is too small, the risk of excluding meaningful biological variation occurs. Larger subsets will reduce this risk, while simultaneously increasing the noise of irrelevant genes which then might overshadow the relevant trends in the data. Users should therefore evaluate different percentages. 10% usually is a good cutoff between both, but we encourage users to evaluate different thresholds.

As this step is also highly depending on the structure of the individual samples of the data set, it is advised to do the feature selection on each sample individually as shown here.

HIDE

```
all.dec <- lapply(sceRNA, modelGeneVar)
all.hvgs <- lapply(all.dec, getTopHVGs, prop = 0.1)
```

## 7.1 Seurat

The feature selection in Seurat also detects highly variable genes in the data and applies a data scaling

afterwards. By default, Seurat returns the 2000 most variable features, which are afterwards used for downstream analyses.

Afterwards, we scale the data using the `ScaleData` function which shifts the expression of each gene, so that the mean expression across cells is 0 and the variance is 1. This ensures equal weight in downstream analyses and that highly-expressed genes do not dominate.

HIDE

```
seurat_merged <- FindVariableFeatures(seurat_merged)

# Extract top 10 variable genes
top10 <- head(VariableFeatures(seurat_merged), 10)
top10

# plot standardized variance against average expression and label top 10 variabl
e genes
plot <- VariableFeaturePlot(seurat_merged)
LabelPoints(plot, points = top10, repel = TRUE)

# data scaling
seurat_merged <- ScaleData(seurat_merged, features = rownames(seurat_merged))
```

# 8 Data integration and merging of the samples

In the previous steps, we analyzed each of our samples individually as all steps which were executed until now will result in more meaningful and refined results if applied to each sample individually. However, all the following downstream steps are best executed on the data as whole.

Hence, we now want to merge our data into a single `SingleCellExperiment` object. In order to do so, we can use two different approaches: In the first approach, we simply integrate the data as it is until now, just correctly and smoothing some of the meta data to avoid conflicts while merging.

However, in more complex data designs with different replicates and batches, it is not advisable to simply just merge the data. In such scenarios it is best to apply some batch effect correction additionally to the already applied normalization to ensure that all results calculated downstream are based on interesting biological effects and variation rather than technical noise and batch effects.

This is why we present both approaches here, the uncorrected and the batch corrected one. During the setup of this workflow, we tested both approaches and evaluated that for the presented data at hand the uncorrected approach is more suitable as the batch correction is rather vigorous on the data and removes interesting and logical biological variation. Nevertheless, we want to present how to apply batch correction and integration to scRNA-seq data. We designed the code in a way that both approaches are interchangeable.

## 8.1 Integration - uncorrected

In this approach, we present the integration without correction. For this, we simply apply a the `multiBatchNorm()` function to normalize the counts on the different samples. Afterwards, we synchronize

the meta data of each sample and then merge the data.

We also merge and synchronize the lists of selected features from the previous step as these will be used in downstream steps.

HIDE

```
# normalize counts across the samples
rescaled <- multiBatchNorm(sceRNA)

# extract the individual samples
iLN <- rescaled$iLN
mLN <- rescaled$mLN
skin <- rescaled$skin
spleen <- rescaled$spleen

# combine the selected features
combined.dec <- combineVar(all.dec)
chosen.hvgs <- combined.dec$bio > 0
sum(chosen.hvgs)
```

```
## [1] 11729
```

Next, we synchronize the individual metadata and combine the final object again.

HIDE

```
# Synchronizing the metadata for cbind()ing.
rowData(iLN) <-
  rowData(iLN)[, c("gene_name", "location")]
rowData(mLN) <-
  rowData(mLN)[, c("gene_name", "location")]
rowData(skin) <-
  rowData(skin)[, c("gene_name", "location")]
rowData(spleen) <-
  rowData(spleen)[, c("gene_name", "location")]

# merge individual objects into one final object
sce_merged <- cbind(
  iLN,
  mLN,
  skin,
  spleen
)
```

The final object `sce_merged` can now be used for the following downstream analyses.

# 8.2 Integration - Harmony

In this version we apply batch correction on the data using the `harmony` package.

In order to be able to use `RunHarmony`, we first have to transform our data set to a `Seurat` object. For this, we start at the read-in step of the data and apply the usual `Seurat` pipeline to the data.

Then we can apply the `RunHarmony` function and check out the batch-corrected data using a UMAP representation of the data. Here, we can see that the batch-correction corrects too strongly as the previously separated skin sample is now merged with the remaining data. However, we expect the skin sample to show different properties compared to the remaining data.

Hence, we decided against the correction and will be using the uncorrected version for downstream analyses. In your own workflow it is always important to compare the data before and after correction to eliminate such overcorrection.

HIDE

```
# Read in the data as seurat object as shown in "1 Create SingleCellExperiment"
# and filter for T cells as shown in "3 Extracting Tcells using T chain receptor
# information". After the merging in this step, you can proceed here.

seurat_merged <- NormalizeData(seurat_merged)
seurat_merged <- FindVariableFeatures(seurat_merged)
seurat_merged <- ScaleData(seurat_merged)
seurat_merged <- RunPCA(seurat_merged)
DimPlot(seurat_merged, reduction = "pca")

seurat_merged <-
  RunHarmony(seurat_merged,
             group.by.vars = "tissue",
             plot_convergence = FALSE)

seurat_merged@reductions

seurat_merged_embedded <- Embeddings(seurat_merged, "harmony")
seurat_merged_embedded[1:10, 1:10]

seurat_merged <- RunUMAP(seurat_merged,
                         reduction = 'harmony',
                         dims = 1:20)
seurat_merged <- FindNeighbors(seurat_merged,
                               reduction = "harmony",
                               dims = 1:20)
seurat_merged <- FindClusters(seurat_merged, resolution = 0.5)


DimPlot(seurat_merged, reduction = "umap")

DimPlot(
  seurat_merged,
  reduction = "umap",
  group.by = "tissue",
  cols = c("springgreen4", "darkmagenta", "tomato4", "darkblue")
)
```

## 8.3 Integration with publicly available data

To further showcase the effect of batch correction, we tried to integrate our data with a publicly available dataset (GSE130879). From this dataset, we used the skin, spleen and LN sample to match the data presented in this paper. After downloading and reading the data as presented earlier in this paper, we tried to integrate and harmonize the two datasets using the `harmony` package.

HIDE

```
# Function to read in the data
# provide all the filepaths to the count data as a list
# a well as a list of the respective tissues
readDataset <- function(filepath_list, tissue) {
  seuratRNA <- list()
  # iterate over each sample of the input data
  for (i in 1:length(filepath_list)) {
    # read the count data
    counts <- Read10X(filepath_list[[i]])
    counts <- counts[, colSums(counts) > 0]
    # generate a Seurat object
    seurat = CreateSeuratObject(counts = counts)
    # Add the tissue type informtation as meta data
    seurat$tissue <- rep(tissue[[i]], ncol(seurat))
    seuratRNA <- c(seuratRNA, seurat)
  }
  # return the list of Seurat objects
  return(seuratRNA)
}

filepaths <- c("../data/LN_public",
               "../data/Skin_public",
               "../data/Spleen_public",
               "../data/iLN",
               "../data/mLN",
               "../data/skin",
               "../data/spleen")

tissue <- c("LN", "Skin", "Spleen", "iLN", "mLN", "skin", "spleen")
seuratRNA <-
  readDataset(filepaths, tissue = tissue)
names(seuratRNA) <- tissue
# Now have a look at the data
seuratRNA

seurat_merged <- merge(seuratRNA$LN,
                      y = c(seuratRNA$Skin,
                            seuratRNA$Spleen,
                            seuratRNA$iLN,
                            seuratRNA$mLN,
                            seuratRNA$skin,
                            seuratRNA$spleen),
                      add.cell.ids = tissue)
seurat <- seurat_merged
```

HIDE

```
seurat[["percent.mt"]] <- PercentageFeatureSet(seurat, pattern = "^Mt-")
VlnPlot(seurat, features = c("nFeature_RNA", "nCount_RNA", "percent.mt"), ncol =
3)
plot1 <- FeatureScatter(seurat, feature1 = "nCount_RNA", feature2 = "percent.m
t")
plot2 <- FeatureScatter(seurat, feature1 = "nCount_RNA", feature2 = "nFeature_RN
A")
CombinePlots(plots = list(plot1, plot2))

seurat_sub <- subset(seurat, subset = nFeature_RNA > 500 & nFeature_RNA < 2500 &
percent.mt < 5)

seurat_sub <- NormalizeData(seurat_sub)
seurat_sub <- FindVariableFeatures(seurat_sub)
seurat_sub <- ScaleData(seurat_sub)
seurat_sub <- RunPCA(seurat_sub)
DimPlot(seurat_sub, reduction = "pca", group.by = "tissue")
seurat_sub <- RunUMAP(seurat_sub,
                  reduction = 'pca',
                  dims = 1:20)
seurat_sub <- FindNeighbors(seurat_sub,
                        reduction = "pca",
                        dims = 1:20)
seurat_sub <- FindClusters(seurat_sub, resolution = 0.5)


DimPlot(seurat_sub, reduction = "umap")

DimPlot(seurat_sub, reduction = "umap", group.by = "tissue")




seurat_sub <- RunHarmony(seurat_sub, group.by.vars = "tissue", plot_convergence
= FALSE)

seurat_sub@reductions

seurat_embedded <- Embeddings(seurat_sub, "harmony")
seurat_embedded[1:10, 1:10]

seurat_sub <- RunUMAP(seurat_sub,
                  reduction = 'harmony',
                  dims = 1:20)
seurat_sub <- FindNeighbors(seurat_sub,
                        reduction = "harmony",
                        dims = 1:20)
seurat_sub <- FindClusters(seurat_sub, resolution = 0.5)

DimPlot(seurat_sub, reduction = "umap")

DimPlot(seurat_sub, reduction = "umap", group.by = "tissue")
```

## 8.4 Seurat

Data integration can be omitted in the Seurat pipeline as the data was already merged before.

# 9 Dimensionality reduction using Principal Component Analysis

A common approach for dimensionality reduction in scRNA-seq is Principal Component Analysis (PCA). As the first couple of principal components (PC) capture the largest amount of variance in the data, hence the most dominant factors of heterogeneity, it can be assumed that these PC represent a considerable amount of biological variation of the data at hand. PCA has also the further advantage that it has been studied intensely which has shown that the low-rank approximation formed by the first couple PCs is the optimal representation of the data at hand. Hence, PCA can capture and concentrate the biological signal in the data while simultaneously reducing the computation time and technical noise.

The choice of number of PCs to use in downstream analyses is a critical one. Like the choice of the number of HVGs, the number of PCs considered can influence the results and data interpretation. Using many PCs retains a considerable amount of biological signal while simultaneously including noise and technical variation. On the other hand, a small number of PCs could exclude a lot of noise and uninteresting variation at the cost of excluding important biological signals. Common choices range between the top 10 and 50 PCs.

In order to evaluate how many PCs to use for the downstream analyses, we plot a scree plot, also know as elbow plot. The plot visualizes each PCs and the amount of variance explained by the respective PCs. A common method to chose the number of PCs is to search/look for the ‘elbow’ in the scree plot; commonly known as a point at which the amount of variance explained of each PCs noticeably drops. As the assumption behind the use of PCA is that the interesting biological variation is captured in the early PCs, a drastic drop in explained variance can point to a change from interesting biological variance to technical variance/noise.

HIDE

```
set.seed(42)
sce_merged <- runPCA(sce_merged,
                     subset_row = chosen.hvgs,
                     BSPARAM = BiocSingular::RandomParam())

# Plot scree plot of the variance explained by each PC
percent.var <- attr(reducedDim(sce_merged), "percentVar")
plot(percent.var,
     log = "y",
     xlab = "PC",
     ylab = "Variance explained (%)")
```

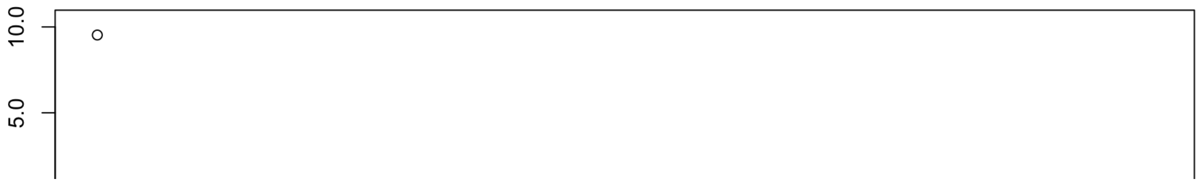

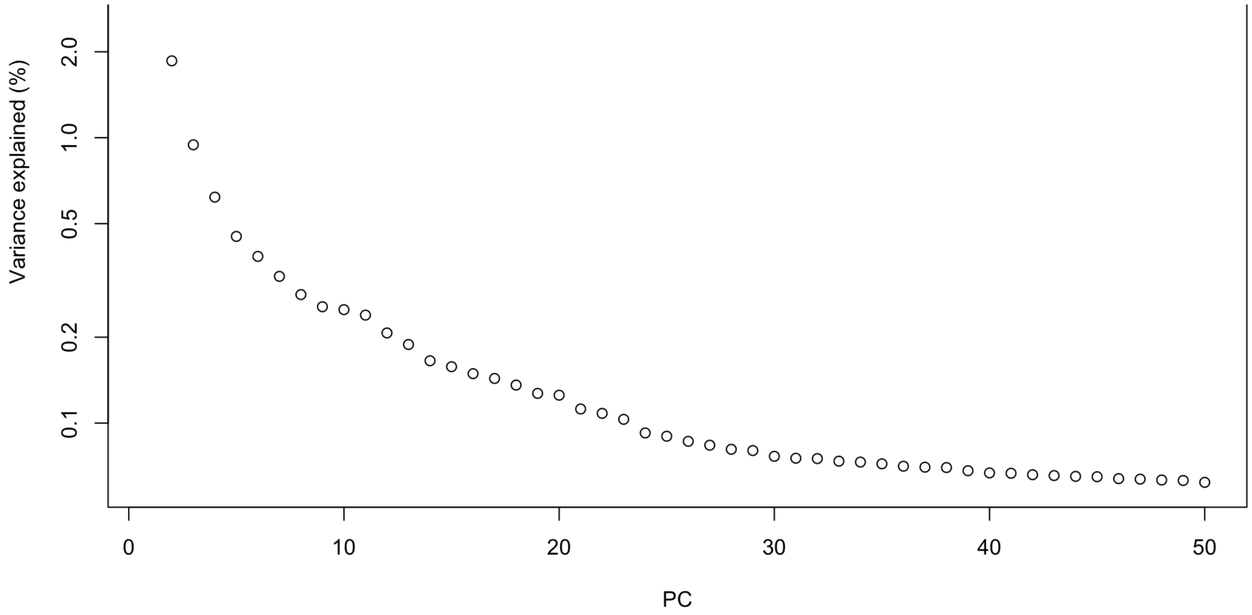


In our data set, we can observe a drop in variance after PC 25. Hence, we used the first 25 PCs for downstream analyses.

Once dimensionality reduction is applied, we can also calculate a t-SNE or UMAP representation of our data. Both visualization techniques are suitable for high-dimensional datasets such as scRNA-seq data. The t-stochastic neighborhood embedding (t-SNE) aims to find a low-dimensionality representation of the data that preserves the distances between points from the high-dimensionality space. Uniform manifold approximation and projection (UMAP) is another non-linear visualization technique for high-dimensionality data, similar to t-SNE. It should be mentioned that both methods are non-deterministic, meaning that they yield slightly different results each time the function is run on the data. We can prevent this by using the R function set.seed() using the same seed each time.

It should be noted that the distance in a t-SNE plot does not accurately present distances between cells and clusters. Hence, just because two clusters a and b are 'further' away from each than a and c, this should not be interpreted as a and c are more alike than a and b. This is a common mistake when intepreting a t-SNE plot which users of this workflow should not commit.

HIDE

```
# calculate UMAP and tSNE representation of the data
set.seed(42)
sce_merged <- runTSNE(sce_merged, dimred = "PCA")

set.seed(42)
sce_merged <- runUMAP(sce_merged, dimred = "PCA")
```

# 9.1 Seurat

In the Seurat pipeline we also use Principle Component Analysis for dimensionality reduction, as well as calculate a UMAP and t-SNE representation of the data. Here we use the first 25 PCs as well.

HIDE

```
seurat_merged <-
  RunPCA(seurat_merged, features = VariableFeatures(seurat_merged))
DimHeatmap(seurat_merged,
           dims = 1,
           cells = 500,
           balanced = TRUE)
ElbowPlot(seurat_merged)

seurat_merged <- RunUMAP(seurat_merged, dims = 1:25)
seurat_merged <- RunTSNE(seurat_merged, dims = 1:25)
```

# 10 Clustering

Clustering is an approach of an scRNA-seq analysis to summarize the high-dimensional, complex data by dividing the cells into individual groups based on gene expression profiles. This greatly eases interpretation and exploration of the data as the cells are then represented as discrete groups rather than the complex, high-dimensional space that is the origin of the data.

In our workflow, we use a graph-based clustering approach implemented in the `buildSNNGraph()` function, followed by the `cluster_walktrap()` function to identify closely connected communities in the graph which will later form the clusters.

HIDE

```
# Calculate the clusters
snn.gr <- buildSNNGraph(sce_merged,
                        k = 25,
                        use.dimred = "PCA")
clusters <- igraph::cluster_walktrap(snn.gr)$membership

# See which tissue can be found in which cluster
tab <- table(Cluster = clusters, Batch = sce_merged$tissue)
tab
```

```
##        Batch
## Cluster  iLN  mLN skin spleen
##      1     0    0  256      0
##      2   369  676    7    973
##      3   503  634    1    191
##      4     0    0  109      0
##      5   237  184   14    380
##      6     0    0  161      0
##      7    37   43    0      8
##      8   180  166   15    313
##      9     0    0  426      1
##      10 1183 1514    0   1546
```

HIDE

```
# Set the cluster as colLabels of the SingleCellExperiment
colLabels(sce_merged) <- factor(clusters)
```

Next, we plot the UMAP and tSNE representation of our data, colored by different characteristics of the data.

We start by coloring the data according to our calculated clusters.

HIDE

```
plotTSNE(sce_merged, colour_by = "label")
```

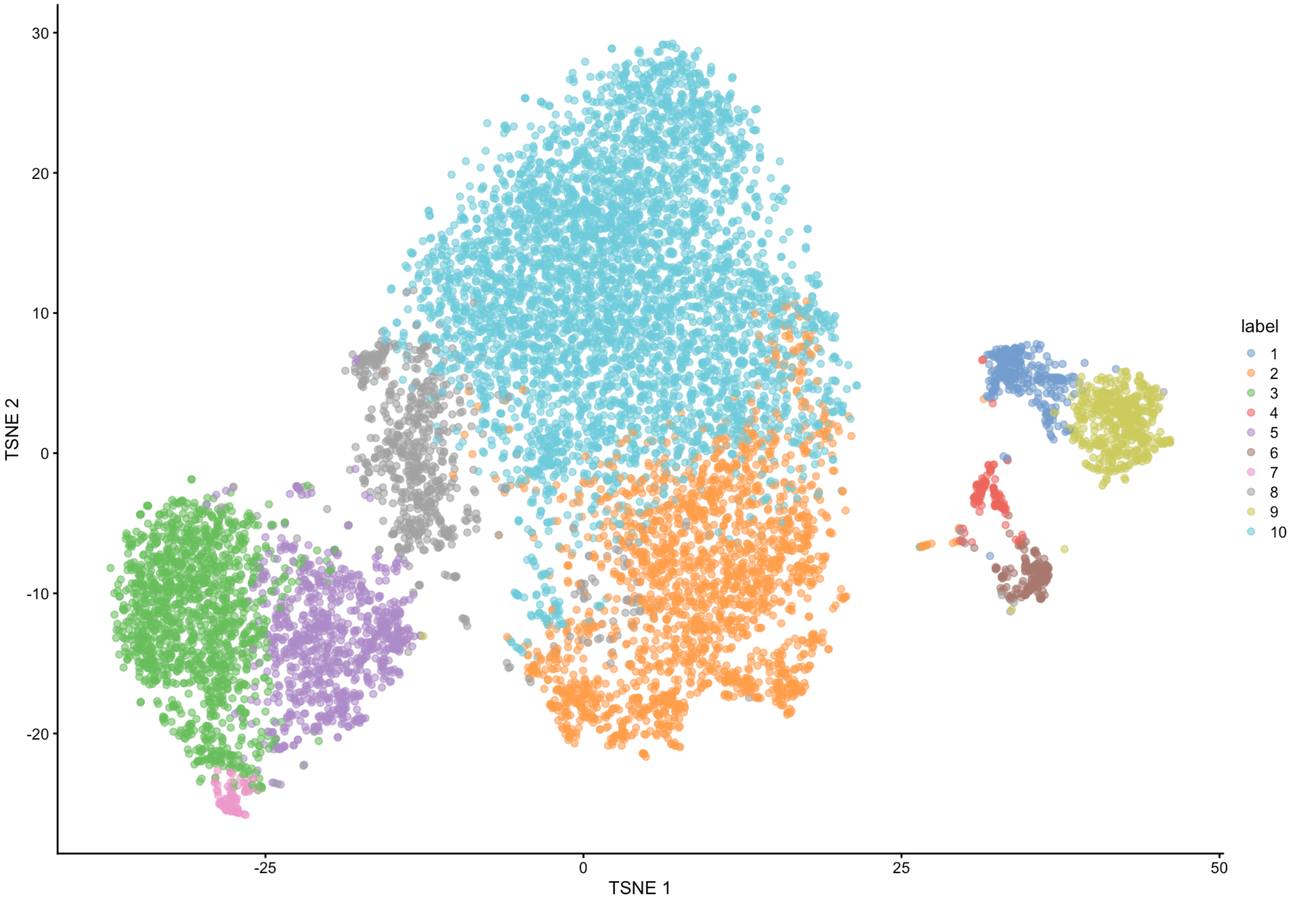


HIDE

```
plotUMAP(sce_merged, colour_by = "label")
```

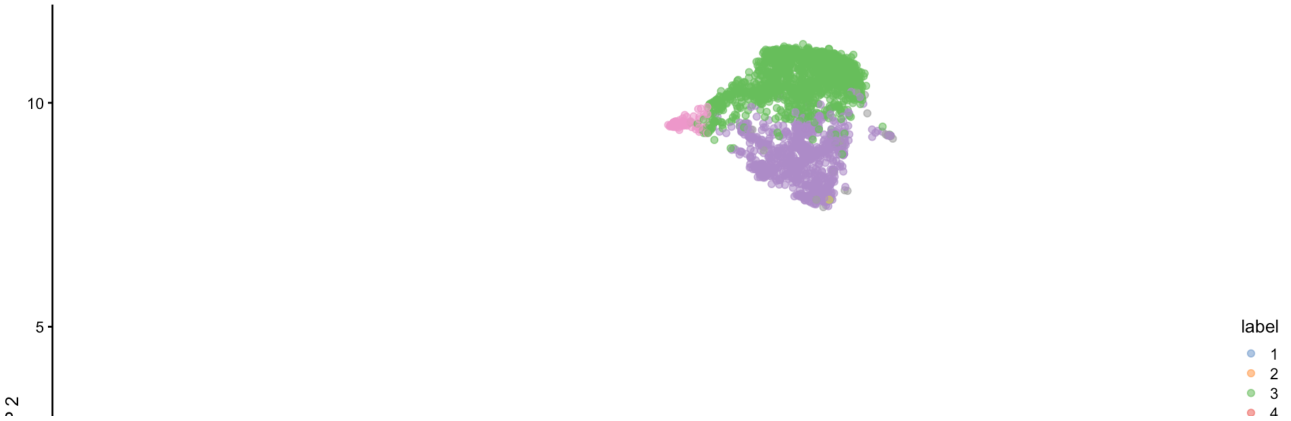

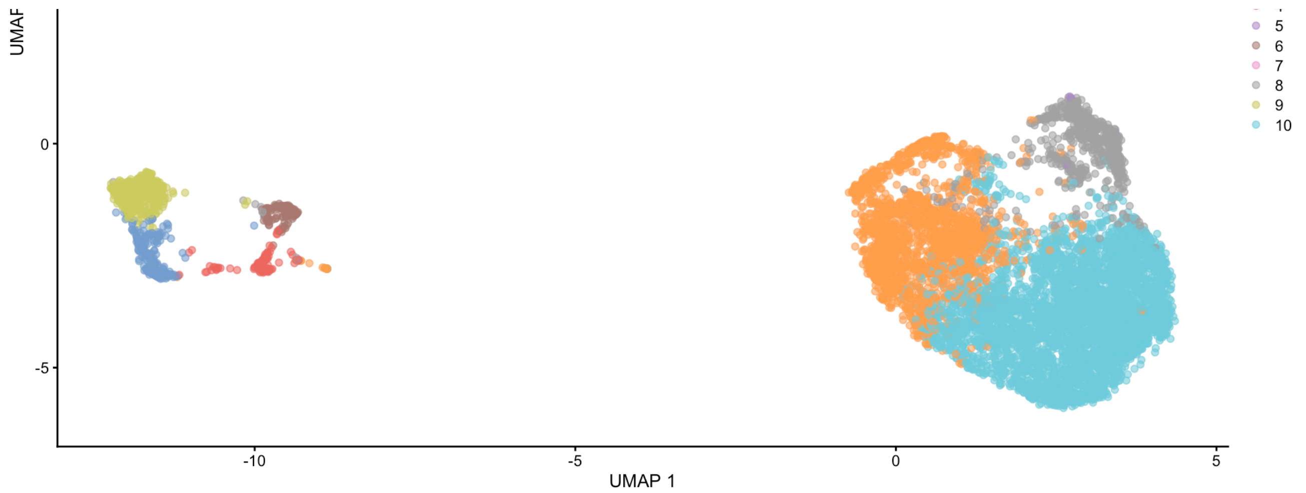


Next, we color by tissue type.

HIDE

```
# color tSNE by tissue
tsne <- plotTSNE(sce_merged, colour_by = "tissue")
# set custom colors, because with the original chosen colors of the method,
# the individual tissues are hard to distinguish.
tsne <- tsne + scale_fill_manual(
  values = c(
    skin = "tomato4",
    spleen = "darkblue",
    iLN = "springgreen4",
    mLN = "darkmagenta"
  ),
  aesthetics = "colour"
)
# plot the tSNE
tsne
```

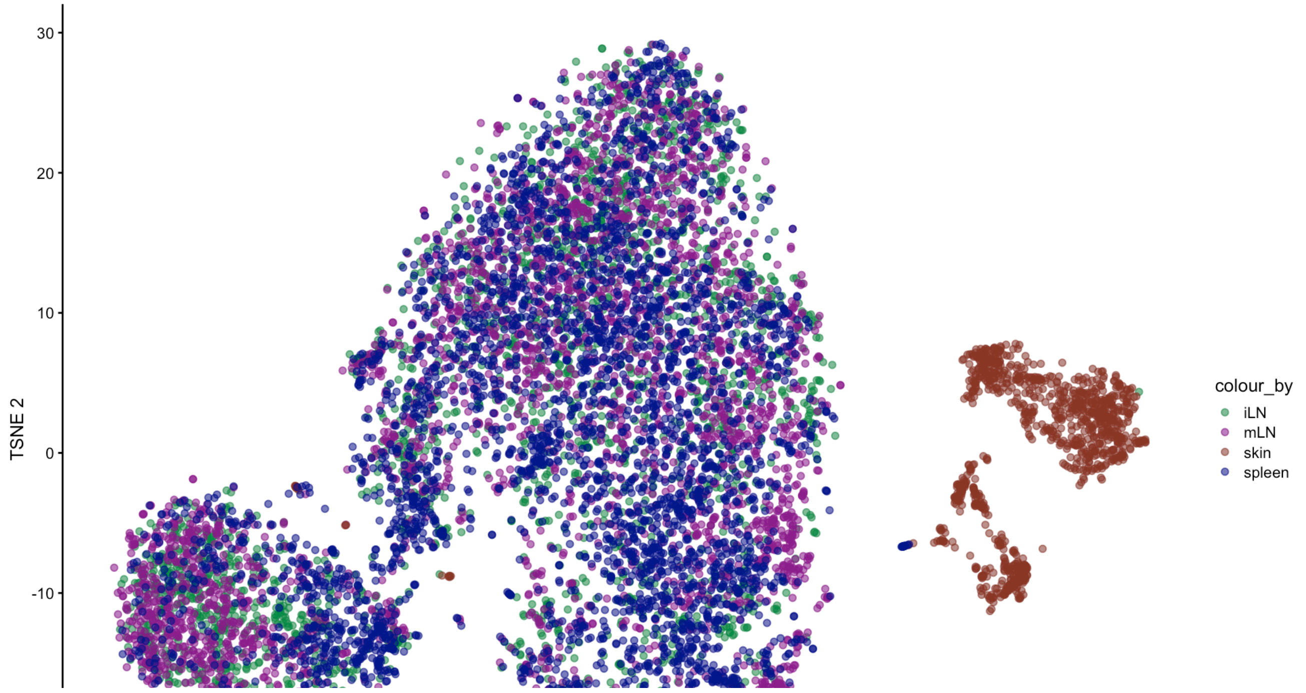

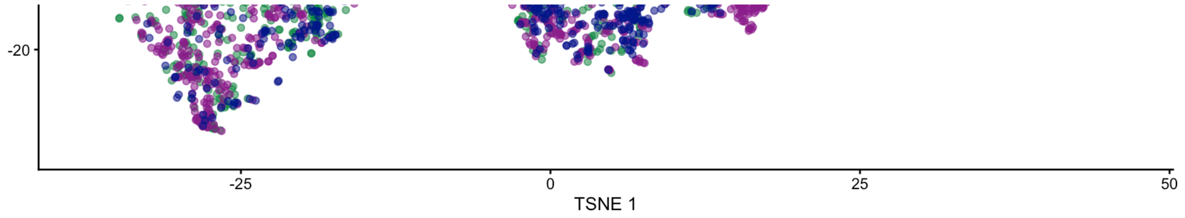


HIDE

```
# color UMAP by tissue
umap <- plotUMAP(sce_merged, colour_by = "tissue")
# set custom colors, because with the original chosen colors of the method,
# the individual tissues are hard to distinguish.
umap <- umap + scale_fill_manual(
  values = c(
    skin = "tomato4",
    spleen = "darkblue",
    iLN = "springgreen4",
    mLN = "darkmagenta"
  ),
  aesthetics = "colour"
)
# plot the UMAP
umap
```

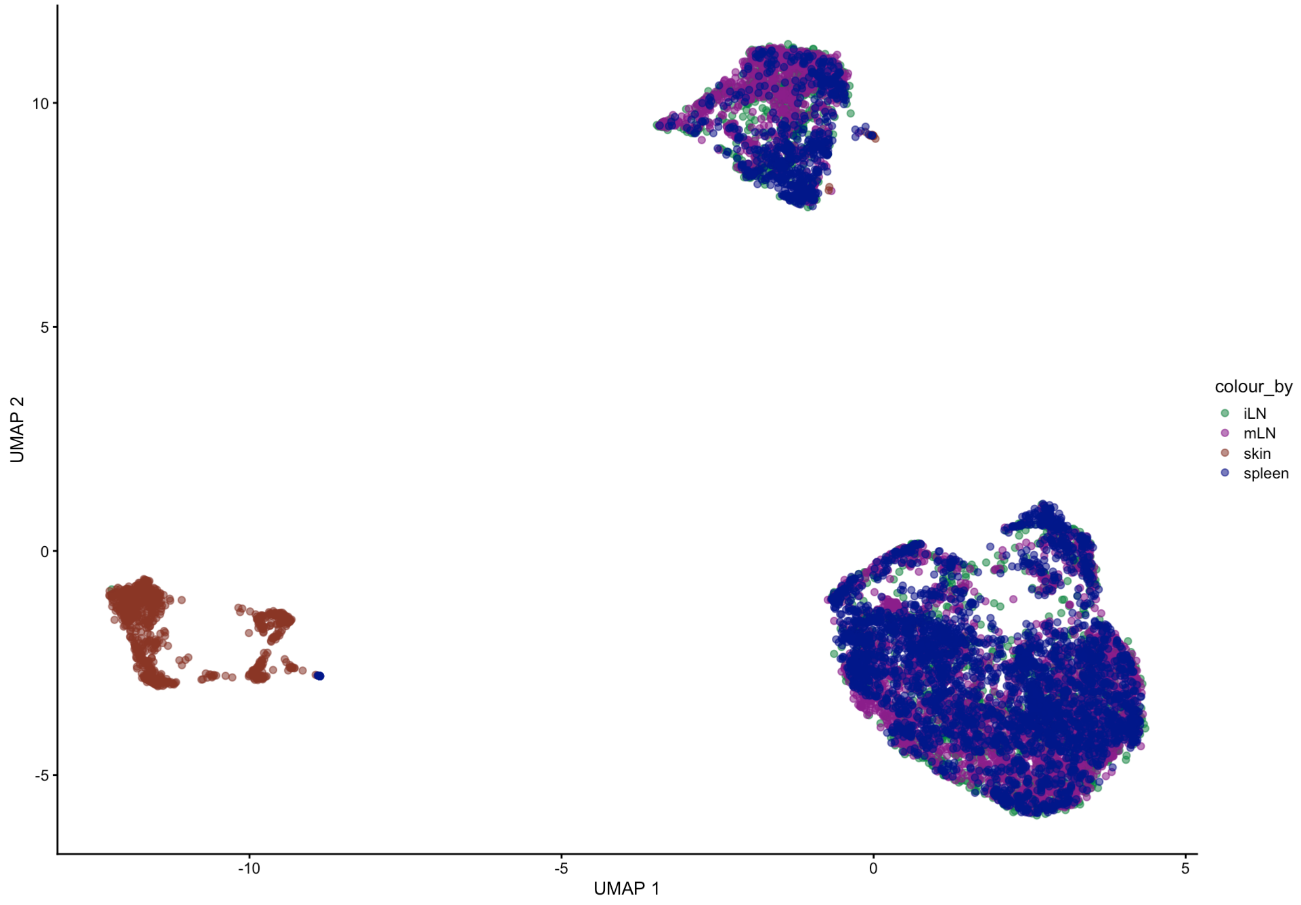


Next, we color the UMAP and tSNE by whether cells are considered a doublet or not.

HIDE

```
# plot tSNE and UMAP colored by doublet identification of cells
plotTSNE(sce_merged, colour_by = "scDblFinder.class")
```

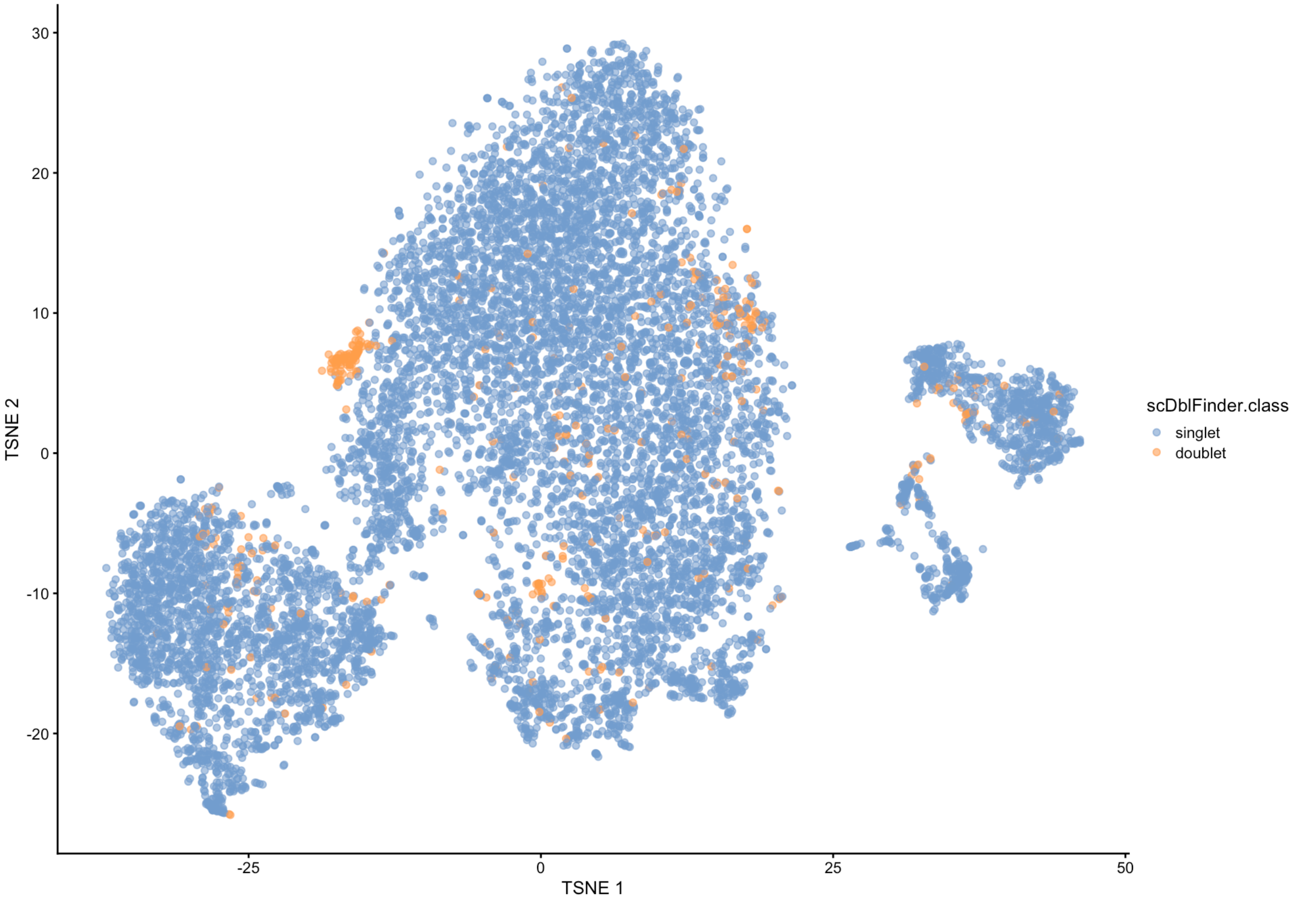


HIDE

```
plotUMAP(sce_merged, colour_by = "scDblFinder.class")
```

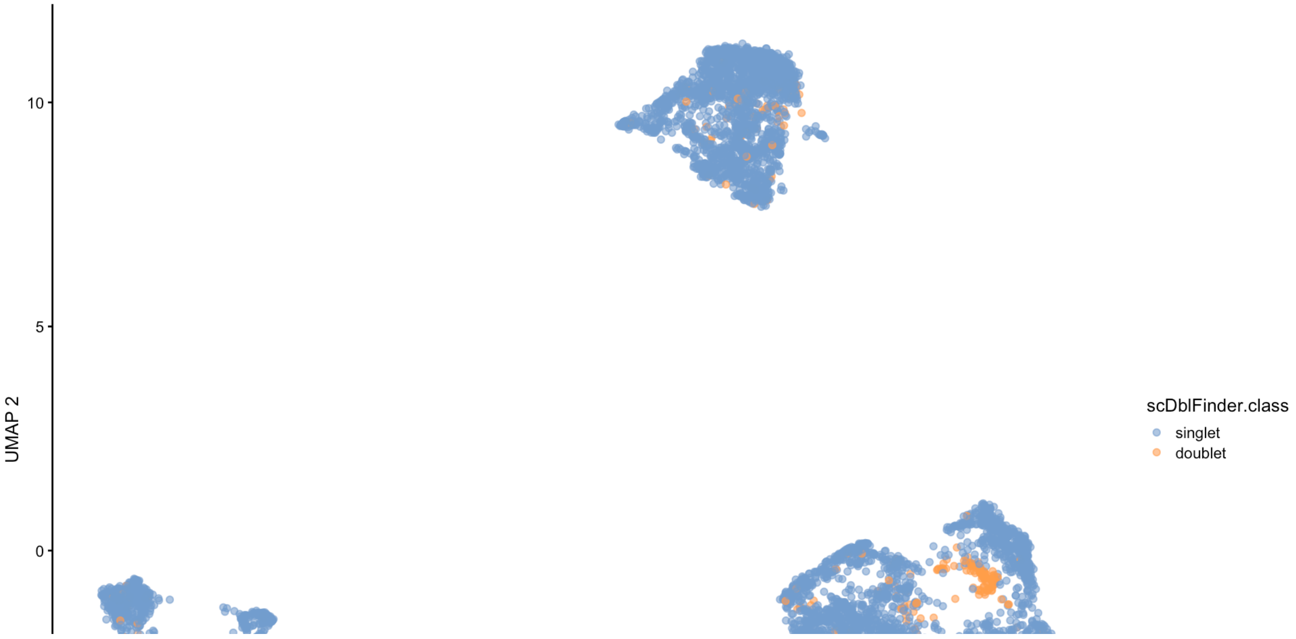

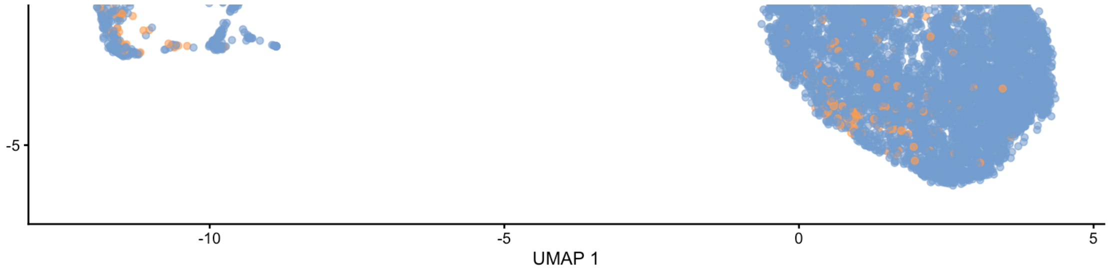


When comparing the UMAP colored by cluster with the one colored by whether or not a cell is considered to be a doublet, we can nicely see that the doublets do not form distinctive clusters. When the doublets form distinctive clusters or pattern, these cells should be further investigated before filtering.

# 10.1 Seurat

The Seurat way of clustering is also rather straightforward. A difference to the `SingleCellExperiment` pipeline is the choice of resolution that is available which will influence the results of the clustering. Here we will show a selection of different resolution options and how they change the cluster results.

HIDE

```
seurat_merged <- FindNeighbors(seurat_merged, dims = 1:25)
seurat_merged <- FindClusters(seurat_merged, resolution = c(0.1, 0.3, 0.5, 0.8,
1))
```

HIDE

```
# tSNEs
DimPlot(seurat_merged, group.by = "RNA_snn_res.0.1", label = T, reduction = "tsn
e")
DimPlot(seurat_merged, group.by = "RNA_snn_res.0.3", label = T, reduction = "tsn
e")
DimPlot(seurat_merged, group.by = "RNA_snn_res.0.5", label = T, reduction = "tsn
e")
DimPlot(seurat_merged, group.by = "RNA_snn_res.0.8", label = T, reduction = "tsn
e")
DimPlot(seurat_merged, group.by = "RNA_snn_res.1", label = T, reduction = "tsn
e")

# UMAPs

DimPlot(seurat_merged, group.by = "RNA_snn_res.0.1", label = T, reduction = "uma
p")
DimPlot(seurat_merged, group.by = "RNA_snn_res.0.3", label = T, reduction = "uma
p")
DimPlot(seurat_merged, group.by = "RNA_snn_res.0.5", label = T, reduction = "uma
p")
DimPlot(seurat_merged, group.by = "RNA_snn_res.0.8", label = T, reduction = "uma
p")
DimPlot(seurat_merged, group.by = "RNA_snn_res.1", label = T, reduction = "uma
p")
```

In the end, we decided to use a resolution of 0.5.

HIDE

```
Idents(sub_scRNA) <- "RNA_snn_res.0.5"
```

# 11 Marker gene detection

After we identified clusters in our data, we are naturally interested in what characteristics drove the separation and composition of the individual clusters. Basically this means which genes and gene expression patterns drove the composition and separation of the clusters.

A simple and straightforward approach to identify such genes is to use a differential expression analysis approach. As the clustering is based on gene expression, strongly differentially expressed genes have likely driven the separation between the individual clusters.

Hence, we first calculate the marker genes for each cluster by determining differentially expressed genes for a specific cluster compared to all other clusters. Then we identify the most interesting marker genes, by sorting all identified markers by their mean log2FoldChange across all cluster comparisons.

Suitable marker genes could even serve as an indication to assign specific cell types to individual cells and clusters. An easy way of assigning cell types to clusters and cells is by using a prior list of known cell type marker genes. However, if such genes are not available or the interest is rather on the genes that separate the clusters, differential expression analysis can be used to detect marker genes. As the clustering is based on the gene expression profiles of the individual cells, strongly differentially expressed genes between two clusters have very likely driven the separation of the two groups of cells.

For cluster 1, 2 and 9 we showcase the list of the top 20 marker genes found. We selected these clusters based on results from the following cell type annotation step.

HIDE

```
# score the marker genes between the individual pairs of clusters
markerGenes <- scoreMarkers(sce_merged, colLabels(sce_merged))

# extract marker genes for cluster 1, 2 and 9
markerGenes_cluster1 <- as.data.frame(markerGenes[[1]])
markerGenes_cluster2 <- as.data.frame(markerGenes[[2]])
markerGenes_cluster9 <- as.data.frame(markerGenes[[9]])

# generate a data table of the top 20 marker for each of the selected clusters
DT::datatable(head(markerGenes_cluster1[order(markerGenes_cluster1$mean.logFC.de
tected),], n = 20))
```

Show 10 entries Search:

| | self.average | other.average | self.detected | other.detected | mean.lo |
|---|---|---|---|---|---|
| Oprm1 | 0 | 0.05669795823992918 | 0 | 0.06661224383278276 | -0.3395 |

| | self.average | other.average | self.detected | other.detected | mean.l |
|---|---|---|---|---|---|
| Nudt8 | 0 | 0.04233092211362215 | 0 | 0.05908759918826953 | -0.3263 |
| Pradc1 | 0 | 0.0540777058271078 | 0 | 0.0690772424838894 | -0.3343 |
| Podnl1 | 0 | 0.05184285112150314 | 0 | 0.05298124289493251 | -0.3051 |
| 0610009B22Rik | 0 | 0.0378406929684247 | 0 | 0.0510219627649949 | -0.2969 |
| Zfp438 | 0 | 0.04963079025087411 | 0 | 0.04989920307245053 | -0.2930 |
| Kcnip2 | 0 | 0.04242193483167235 | 0 | 0.04735641136470294 | -0.2840 |
| Ccdc15 | 0 | 0.04562595903331355 | 0 | 0.04620374816420512 | -0.2795 |
| Zcwpw1 | 0 | 0.03033877354986378 | 0 | 0.04517028965935804 | -0.2637 |
| Dhfr | 0 | 0.0269323511140193 | 0 | 0.03683746850669942 | -0.250 |

Showing 1 to 10 of 20 entries Previous 1 2 Next

HIDE

```
DT::datatable(head(markerGenes_cluster2[order(markerGenes_cluster2$mean.logFC.de
tected),], n = 20))
```

Show 10 entries Search:

| | self.average | other.average | self.detected | other.detected |
|---|---|---|---|---|
| Gm37401 | 0 | 0.01178393651504471 | 0 | 0.01627532207552104 |
| Ccr10 | 0.004561509201838497 | 0.1061902429123117 | 0.004444444444444444 | 0.095520044662426 |
| Ninj1 | 0.02077975387638206 | 0.1063143213608783 | 0.02123456790123457 | 0.123980036670225 |
| Myadm | 0.02310525678536937 | 0.2148538455878246 | 0.0202469135802469 | 0.1821887421263756 |
| Crip2 | 0.006632333871608192 | 0.05975654094781094 | 0.005925925925925926 | 0.06231307575432805 |
| Gm20492 | 0 | 0.006426747199967035 | 0 | 0.008415220185580346 |
| Cks1b | 0.01075143516222847 | 0.06004427803996802 | 0.01037037037037037 | 0.0838310903610497 |
| Gm26887 | 0.003070700088679163 | 0.03910057674223737 | 0.002962962962962964 | 0.03902292361872623 |
| Sh3bp4 | 0.009209704911017467 | 0.1207177106289762 | 0.007407407407407409 | 0.1098117727864724 |

| | self.average | other.average | self.detected | other.detected |
|---|---|---|---|---|
| Ly6g5b | 0.00377098053068817 | 0.04677389791731314 | 0.003456790123456784 | 0.04686114379175289 |

Showing 1 to 10 of 20 entries

Previous 1 2 Next

HIDE

```
DT::datatable(head(markerGenes_cluster9[order(markerGenes_cluster9$mean.logFC.de
tected),], n = 20))
```

Show 10 entries

Search:

| | self.average | other.average | self.detected | other.detected |
|---|---|---|---|---|
| 2610204G07Rik | 0.002251152935078872 | 0.1275327126653699 | 0.002341920374707259 | 0.10725264841399 |
| P2ry10b | 0 | 0.04229949672985315 | 0 | 0.04382179978278 |
| Ms4a4c | 0.002921929754652695 | 0.1033290235524129 | 0.002341920374707259 | 0.09613940812878 |
| Apol7e | 0.002864520177917722 | 0.06670553507753328 | 0.002341920374707259 | 0.07367626028043 |
| 2900005J15Rik | 0 | 0.03644289173477436 | 0 | 0.04390647168562 |
| Tgfbr3 | 0.006142520012682015 | 0.1285905641820393 | 0.007025761124121776 | 0.11804409133980 |
| Gm49180 | 0 | 0.05086975144321165 | 0 | 0.04376479935264 |
| Wdr89 | 0.001837684964152636 | 0.0498714496431848 | 0.002341920374707259 | 0.05962239470825 |
| Gm47015 | 0.003169784037238691 | 0.05528941481868018 | 0.002341920374707259 | 0.05177613945028 |
| Mtx3 | 0 | 0.02490477706762827 | 0 | 0.02815117006720 |

Showing 1 to 10 of 20 entries

Previous 1 2 Next

HIDE

```
# plot the expression of the top 6 marker genes for each cluster in every of
# the clusters
plotExpression(
  sce_merged,
  features = head(rownames(markerGenes_cluster1)),
  x = "label",
  colour_by = "label"
)
```

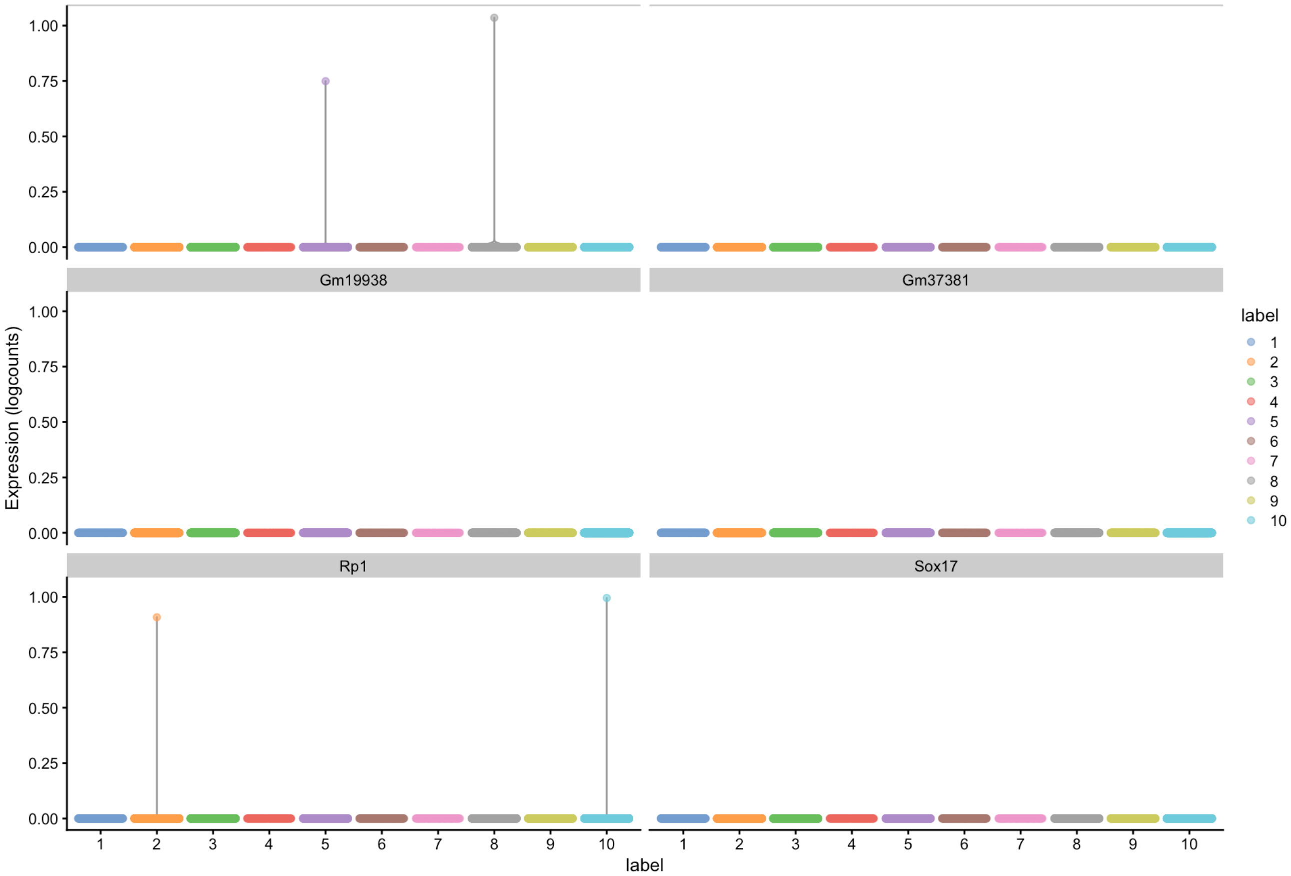


HIDE

```
plotExpression(
  sce_merged,
  features = head(rownames(markerGenes_cluster2)),
  x = "label",
  colour_by = "label"
)
```

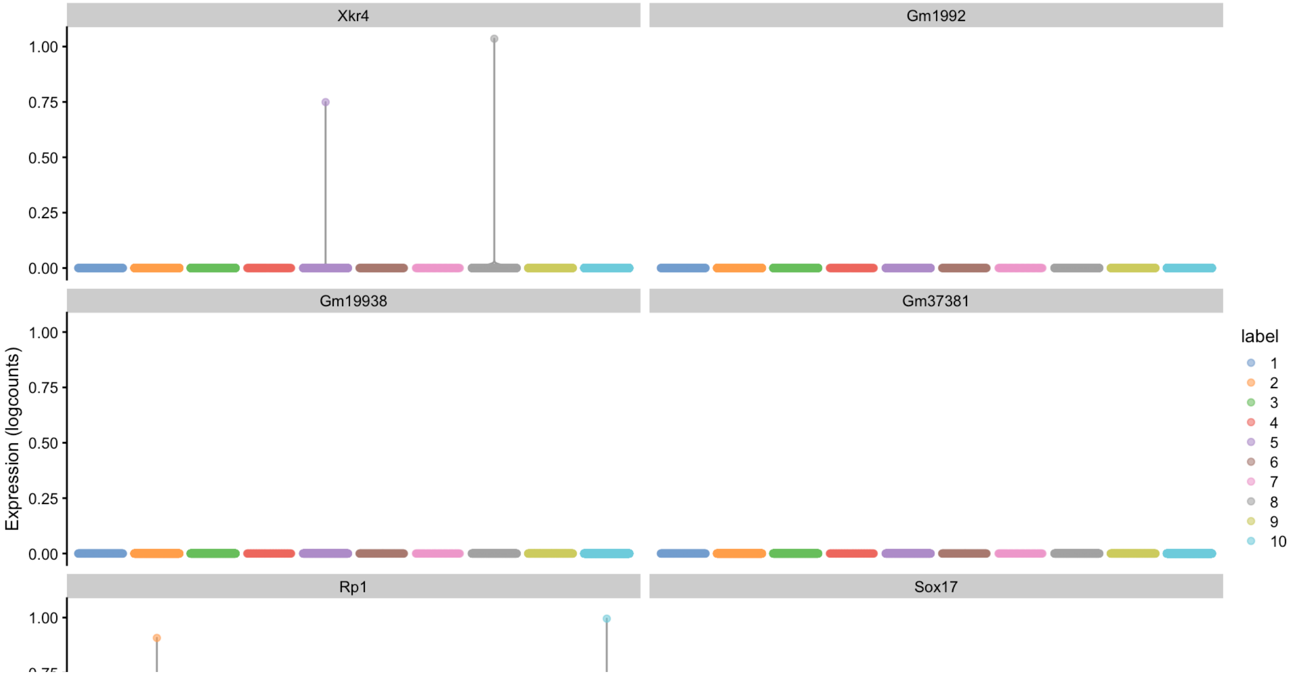

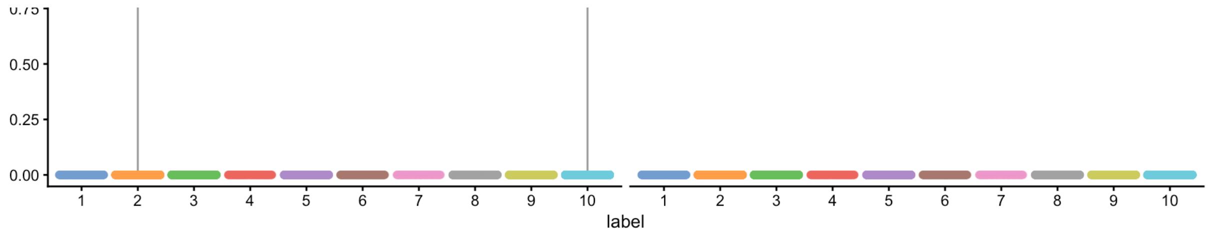


HIDE

```
plotExpression(
  sce_merged,
  features = head(rownames(markerGenes_cluster9)),
  x = "label",
  colour_by = "label"
)
```

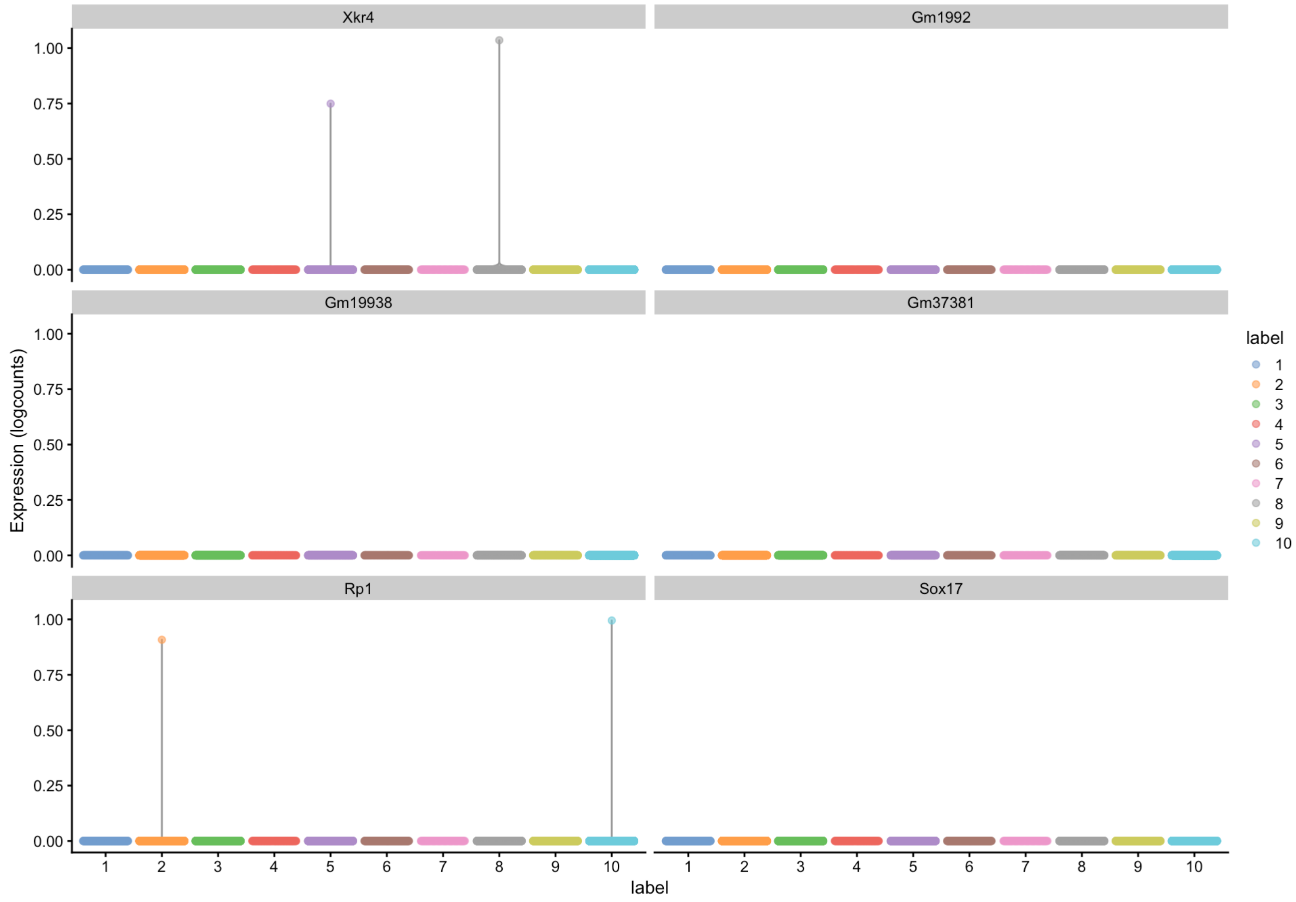


# 11.1 Seurat

We can also identify cluster markers in the Seurat pipeline. We showcase a resolution of 0.5 here and the results of one cluster.

HIDE

```
# Use only those markers that have a positive lFC
cluster.markers <- FindAllMarkers(seurat_merged, only.pos = TRUE, test.use = "bi
mod")
head(cluster.markers)

small_markers <- cluster.markers %>% group_by(cluster) %>% top_n(n = 10, wt = av
g_log2FC)
small_markers
```

### 11.1.1 Cluster 0

HIDE

```
VlnPlot(seurat_merged, features = small_markers[small_markers$cluster == 0, ]$ge
ne)
FeaturePlot(seurat_merged, features = small_markers[small_markers$cluster == 0,
]$gene)
```

# 12 TCR repertoire diversity

TCR V(D)J sequencing coupled with single-cell RNA sequencing enables profiling of paired TCRα and TCRβ chains at single-cell resolution with coupled global gene expression in the same cell. This analysis makes it possible to characterize T-cell clonal expansion in steady state and in disease, as well as tracking shared T-cell clonotypes between different tissues. In our analysis, we wanted to use this information to evaluate if there are shared TCR chains between different tissues as well as different clusters.

In a first step, we wanted to evaluate the TCR repertoire of the individual clusters. We first set up a data frame capture the TCR repertoire of each cluster. We use this information for the pie-charts, after suming all singleton TCR for each cluster. The piecharts will show the singletons of each cluster merged as one large green area, while all multiple occuring TCR will have their own section of the chart.

After plotting the piecharts,w e can see that each cluster, expect for cluster 7, has TCR chains that occur more than once.

HIDE

```
buildDFClonolarity <- function(sce, cluster = NULL){
  if(!is.null(cluster)){
    sce <- sce[, colLabels(sce) == cluster]
  }

  n_occur <- data.frame(table(sce$cdr3s_aa))
  singletons <- 0
  n_occur_sum <- as.data.frame(t(data.frame(singleton = c("placeholder", 0))))
  colnames(n_occur_sum) <- c("Var1", "Freq")

  for(i in 1:nrow(n_occur)){
    if(n_occur[i, ]$Freq == 1){
      singletons = singletons + 1
    }else{
      n_occur_sum <- rbind(n_occur_sum, n_occur[i, ])
    }
  }
  n_occur_sum["singleton", ] <- c("Singletons", singletons)
  colnames(n_occur_sum) <- c("TCR_chain", "Freq")
  return(n_occur_sum)
}


tcr_clono_1 <- buildDFClonolarity(sce_merged, cluster = 1)
tcr_clono_2 <- buildDFClonolarity(sce_merged, cluster = 2)
tcr_clono_3 <- buildDFClonolarity(sce_merged, cluster = 3)
tcr_clono_4 <- buildDFClonolarity(sce_merged, cluster = 4)
tcr_clono_5 <- buildDFClonolarity(sce_merged, cluster = 5)
tcr_clono_6 <- buildDFClonolarity(sce_merged, cluster = 6)
tcr_clono_7 <- buildDFClonolarity(sce_merged, cluster = 7)
tcr_clono_8 <- buildDFClonolarity(sce_merged, cluster = 8)
tcr_clono_9 <- buildDFClonolarity(sce_merged, cluster = 9)
tcr_clono_10 <- buildDFClonolarity(sce_merged, cluster = 10)

myPalette <- brewer.pal(36, "Set2")
pie(as.numeric(tcr_clono_1$Freq), col = myPalette, labels = NA)
```

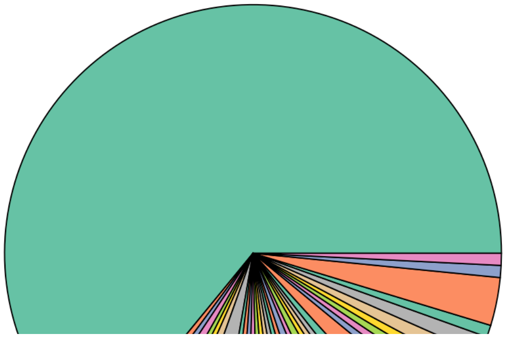

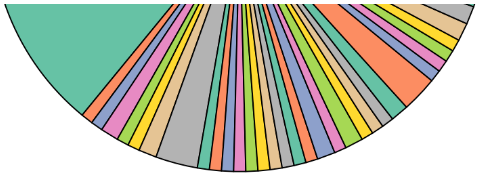

HIDE

```
myPalette <- brewer.pal(48, "Set2")
pie(as.numeric(tcr_clono_2$Freq), col = myPalette, labels = NA)
```

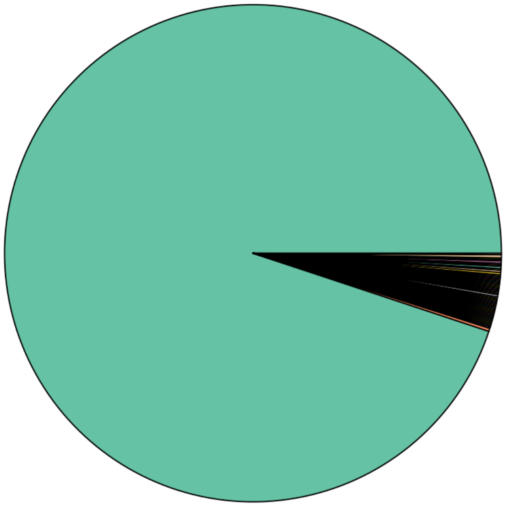

HIDE

```
myPalette <- brewer.pal(11, "Set2")
pie(as.numeric(tcr_clono_3$Freq), col = myPalette, labels = NA)
```

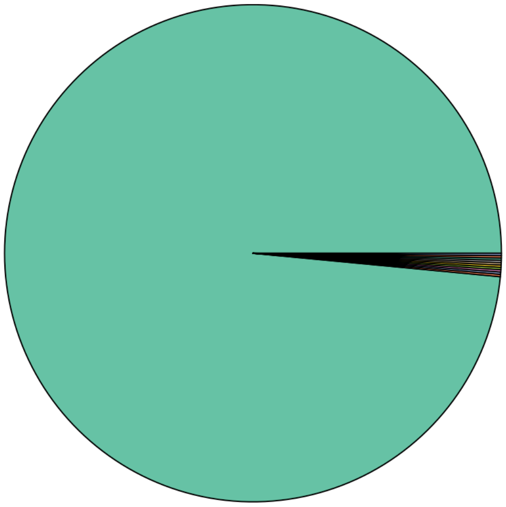

HIDE

```
myPalette <- brewer.pal(5, "Set2")
pie(as.numeric(tcr_clono_4$Freq), col = myPalette, labels = NA)
```

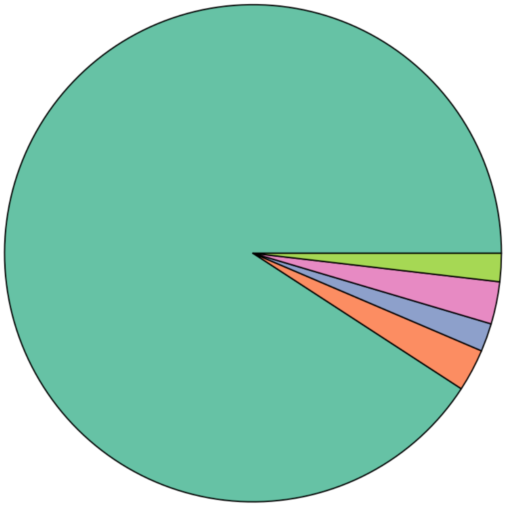

HIDE

```
myPalette <- brewer.pal(41, "Set2")
pie(as.numeric(tcr_clono_5$Freq), col = myPalette, labels = NA)
```

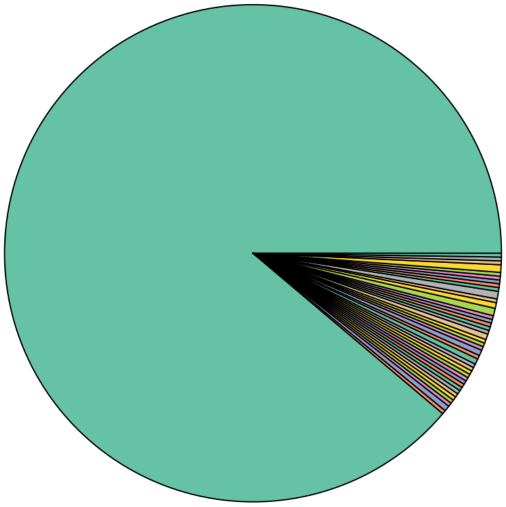

HIDE

```
myPalette <- brewer.pal(23, "Set2")
pie(as.numeric(tcr_clono_6$Freq), col = myPalette,labels = NA)
```

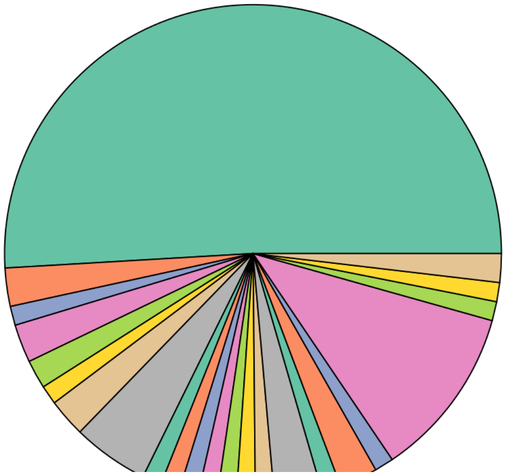

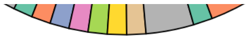

HIDE

```
myPalette <- brewer.pal(1, "Set2")
pie(as.numeric(tcr_clono_7$Freq), col = myPalette, labels = NA)
```

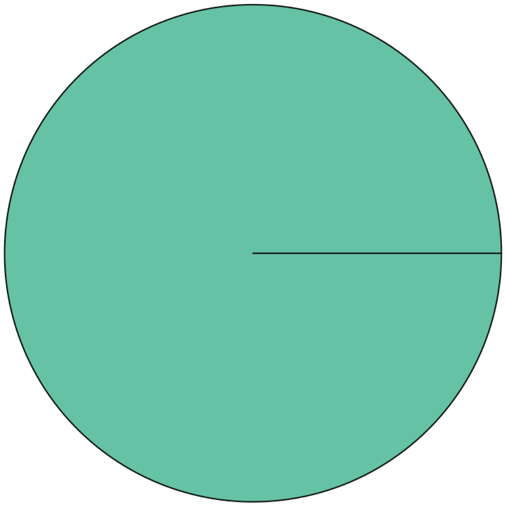

HIDE

```
myPalette <- brewer.pal(11, "Set2")
pie(as.numeric(tcr_clono_8$Freq), col = myPalette, labels = NA)
```

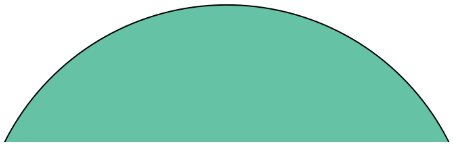

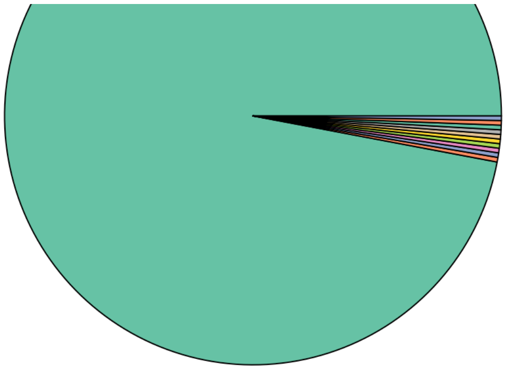

HIDE

```
myPalette <- brewer.pal(60, "Set2")
pie(as.numeric(tcr_clono_9$Freq), col = myPalette, labels = NA)
```

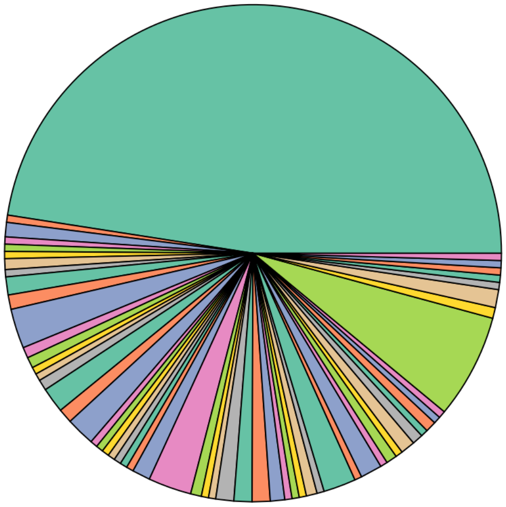

HIDE

```
myPalette <- brewer.pal(31, "Set2")
pie(as.numeric(tcr_clono_10$Freq), col = myPalette, labels = NA)
```

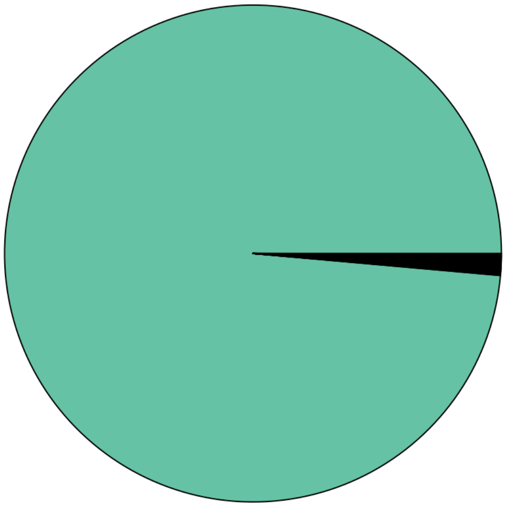

Afterwards, we evaluate which TCR chains are shared between cells of different tissue and overlay this information onf the UMAP of our data set.

HIDE

```
chains_frequency <- table(sce_merged$cdr3s_aa)
chains_duplicated <- chains_frequency > 1


is_duplicated_chains <- sapply(sce_merged$cdr3s_aa, function(x) chains_duplicate
d[[x]])

which_chain <- sapply(sce_merged$cdr3s_aa, function(x) if(chains_duplicated
[[x]]){
  x
}else{NA})

sce_merged$duplicated_chains <- is_duplicated_chains
sce_merged$which_duplicated_chain <- which_chain

clonotype_frequency <- table(sce_merged$clonotype)
clonotype_duplicated <- clonotype_frequency > 1

is_duplicated_clonotype <- sapply(sce_merged$clonotype, function(x) clonotype_du
plicated[[x]])

which_clonotype <- sapply(sce_merged$clonotype, function(x) if(clonotype_duplica
ted[[x]]){
  x
}else{NA})

sce_merged$duplicated_clonotype <- is_duplicated_clonotype
sce_merged$which_duplicated_clonotype <- which_clonotype

plotUMAP(sce_merged, color_by = "duplicated_clonotype", order_by = "duplicated_c
lonotype")
```

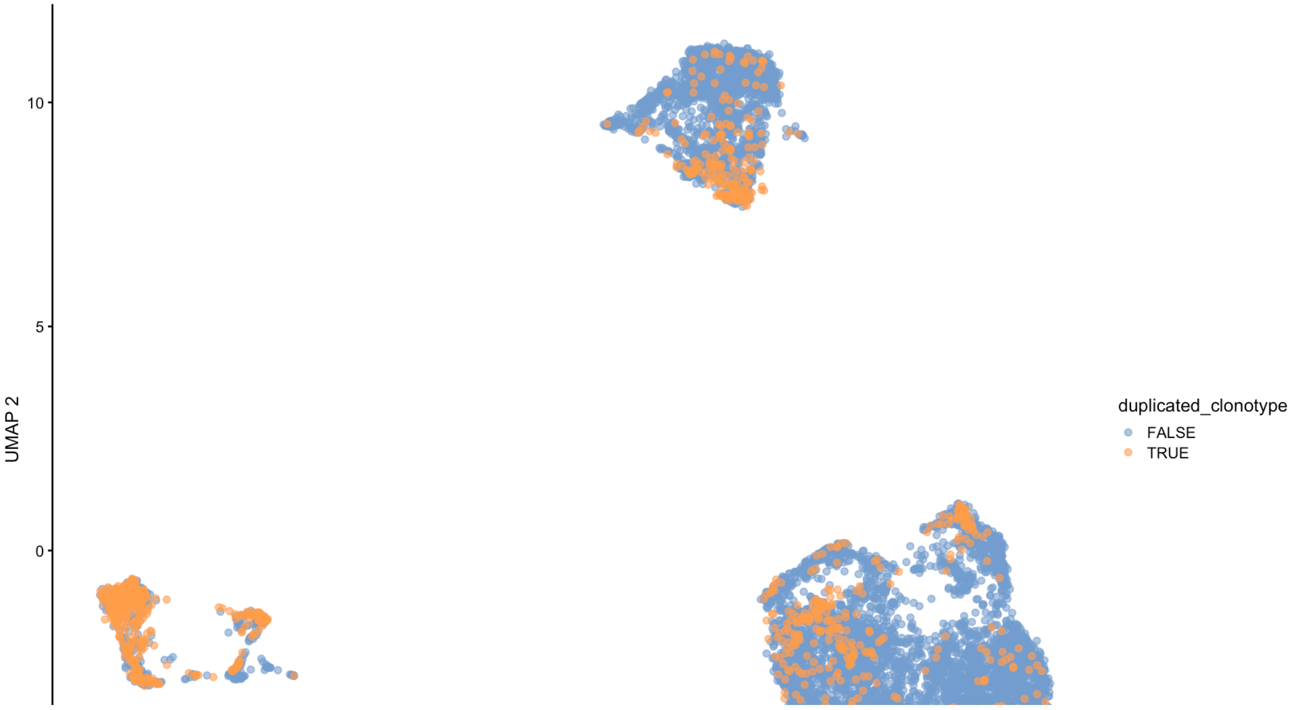

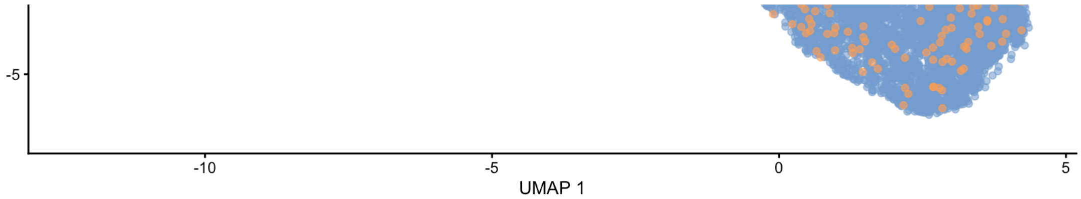


Lastly, we also wanted to evaluate which of the clonotypes in the cluster 1 and 9 are shared with other clusters. Cluster 1 and 9 exclusively consits of skin cells hence it is of great interest to us to analyze if there are shared clonotypes between cells of the skin and other tissue cells. We first overlay the information on the UMAP before plotting barplots of the shared clonotypes. Here, we can see that the clonotypes of cluster 1 and 9 are mainly shared between the 3 clusters but there are also other clusters that share clonotypes with cluster 1 and 9.

HIDE

```
clonotype_cluster1 <- sce_merged[, colLabels(sce_merged) == "1"]$clonotype
clono_cluster1_other_clusters <- sce_merged$clonotype %in% clonotype_cluster1
sce_merged$clonotype_cluster1_shared <- clono_cluster1_other_clusters

# overlay shared clonotypes on the UMAP
plotUMAP(sce_merged, color_by = "clonotype_cluster1_shared", order_by = "clonoty
pe_cluster1_shared")

# plot shared clonotypes as barplot
data <- as.data.frame(table(sce_merged$clonotype_cluster1_shared, colLabels(sce_
merged)))
data <- data[data$Var1 == TRUE, ]
data <- data[!data$Var2 == 1, c(2:3)]
colnames(data) <- c("Cluster", "Frequency")

ggplot(data, aes(x=Cluster, y=Frequency, fill = Cluster, label = Frequency)) +
  geom_bar(stat = "identity") +
  geom_text(size = 5, position = position_stack(vjust = 0.5)) +
  theme_bw()


# Repeat the same steps for cluster 9
clonotype_cluster9 <- sce_merged[, colLabels(sce_merged) == "9"]$clonotype
clono_cluster9_other_clusters <- sce_merged$clonotype %in% clonotype_cluster9
sce_merged$clonotype_cluster9_shared <- clono_cluster9_other_clusters

plotUMAP(sce_merged, color_by = "clonotype_cluster9_shared", order_by = "clonoty
pe_cluster9_shared")

data <- as.data.frame(table(sce_merged$clonotype_cluster9_shared, colLabels(sce_
merged)))
data <- data[data$Var1 == TRUE, ]
data <- data[!data$Var2 == 9, c(2:3)]
colnames(data) <- c("Cluster", "Frequency")

ggplot(data, aes(x=Cluster, y=Frequency, fill = Cluster, label = Frequency)) +
  geom_bar(stat = "identity") +
  geom_text(size = 5, position = position_stack(vjust = 0.5)) +
  theme_bw()
```

# 13 Cell type annotation using reference data and custom markers

In one of the last steps, we would like to assign cell types to our clusters to further ease the interpretation of the data. There are generally two approaches to cell type annotation: using a reference data set which is sufficiently annotated or using a set of known marker genes of cell types. However, the last approach only works if the general composition of cell types in the data is known.

In our data set, we know that the data mostly consist of T cells as we filtered for those using the TCR chain

information. As there is also a list of well studied and known marker genes of different T-cell types available, this is the more suitable approach here.

Nevertheless, we will demonstrate how to do cell type annotation using a reference data set and the `SingleR` package. For the reference data, we use the widely used ImmGen data set, as well as the mouse atlas.

In our analysis, we also realized that the cell type annotation based on our preselected marker genes could greatly benefit from the additional annotation information provided through the annotation using our reference data set of T-cell types. Especially in clusters and areas where there was so clear, exclusive expression of specific marker genes, the additional information was beneficial and supported the interpretation of the data.

Hence, we recommend a combined approach for cell type annotation based on suitable reference data and known cell type marker genes, if those are available.

# 13.1 Reference data

In the first step, we present how to do the cell type annotation using a reference data set. For this, we first load the reference data of the ImmGen data set and the Mouse Atlas.

HIDE

```
ref_annot_immgen <- ImmGenData()
ref_annot_mouse <- MouseRNAseqData()
```

Next, we do the cell type annotation for each data set. Reference data sets for scRNA-seq data usually have main and fine labels. Main labels usually are broad and general labels such as NKT, T-cell, B-cell etc., while the fine labels distinguish finer and more detailed into individual cell types. The degree of detailedness on the cell types is fairly depending on the research question at hand, which is why some analyses might benefit from the fine labels, while others are already satisfied with the main labels.

After, we calculated the cell type annotation, we print a summary table for both the main and fine labels and assign both results as meta data to our `SingleCellExperiment` object to save the information.

We also plot for each reference data set the UMAP and t-SNE representation of the data colored by the assigned cell types. Afterwards, we also plot a heatmap of each cell colored by their degree of matching to the individual cell types in the reference data. The degree of matching ranks from low to high. Ideally, each cell in the data should only have one cell type with a high matching degree. Additionally, we plot a heatmap of cluster to cell types to visualize which cell types can mainly be found in the individual cluster of our data.

## 13.1.1 ImmGen

HIDE

```
# Calculate cell type annotations
celltype_immgen_main <- SingleR(test = sce_merged,
                                ref = ref_annot_immgen,
                                labels = ref_annot_immgen$label.main,
                                BPPARAM = BiocParallel::MulticoreParam(6))
celltype_immgen_fine <- SingleR(test = sce_merged,
                                ref = ref_annot_immgen,
                                labels = ref_annot_immgen$label.fine,
                                BPPARAM = BiocParallel::MulticoreParam(6))
```

HIDE

```
# summarize cell type annotation results
table(celltype_immgen_main$labels)
```

```
##
##        ILC Mast cells        NKT    T cells        Tgd
##         25          1        817       9258         26
```

HIDE

```
table(celltype_immgen_fine$labels)
```

```
##
##                 DC (DC.PDC.8-)                         ILC (ILC2)
##                              1                                  5
##              ILC (LPL.NCR+CNK)                      NKT (NKT.4-)
##                              1                                  4
##                   NKT (NKT.4+)               NKT (NKT.44+NK1.1-)
##                             35                                  9
##            NKT (NKT.44+NK1.1+)                 T cells (T.4.Pa)
##                             44                                  6
##              T cells (T.4.PLN)             T cells (T.4FP3+25+)
##                              3                               4517
##               T cells (T.4Mem)                 T cells (T.4MEM)
##                            145                                 15
##         T cells (T.4MEM44H62L) T cells (T.4MEM49D+11A+.D30.LCMV)
##                             50                                 13
##               T cells (T.4NVE)      T cells (T.4NVE44-49D-11A-)
##                             79                                  1
##             T cells (T.4SP24-)  T cells (T.8EFF.OT1.12HR.LISOVA)
##                              2                                  5
## T cells (T.8EFF.OT1.48HR.LISOVA)      T cells (T.8EFF.OT1.D10LIS)
##                              2                                  1
##   T cells (T.8EFF.OT1.D5.VSVOVA)  T cells (T.8EFF.TBET-.OT1LISOVA)
##                              2                                  1
## T cells (T.8MEM.OT1.D106.VSVOVA)   T cells (T.8MEM.OT1.D45.LISOVA)
##                              1                                  1
##               T cells (T.8Mem)                T cells (T.CD4.1H)
##                              1                                 13
##             T cells (T.CD4.24H)               T cells (T.CD4.48H)
##                             22                                 21
##              T cells (T.CD4.5H)               T cells (T.CD4.96H)
##                            103                                 94
##             T cells (T.CD4.CTR)            T cells (T.CD4+TESTDB)
##                              1                                 13
##         T cells (T.CD4+TESTNA)            T cells (T.CD4CONTROL)
##                             50                                112
##           T cells (T.CD4TESTCJ)             T cells (T.CD4TESTJS)
##                           1056                                 12
##              T cells (T.CD8.1H)                T cells (T.CD8.5H)
##                              2                                  5
##             T cells (T.CD8.96H)                 T cells (T.Tregs)
##                              1                               3619
##         Tgd (Tgd.mat.VG1+VD6+)              Tgd (Tgd.mat.VG2+)
##                              3                                 50
##              Tgd (Tgd.mat.vg3.)                Tgd (Tgd.mat.vg3)
##                              1                                  5
```

HIDE

```
# save results as meta data in the SingleCellExperiment object
sce_merged$celltype_immgen_main <- celltype_immgen_main$labels
sce_merged$celltype_immgen_fine <- celltype_immgen_fine$labels
```

HIDE

```
# plot UMAP and tSNE representation colored by the assigned cell types from the
# main labels
plotTSNE(sce_merged,
         colour_by = "celltype_immgen_main",
         text_by = "celltype_immgen_main")
```

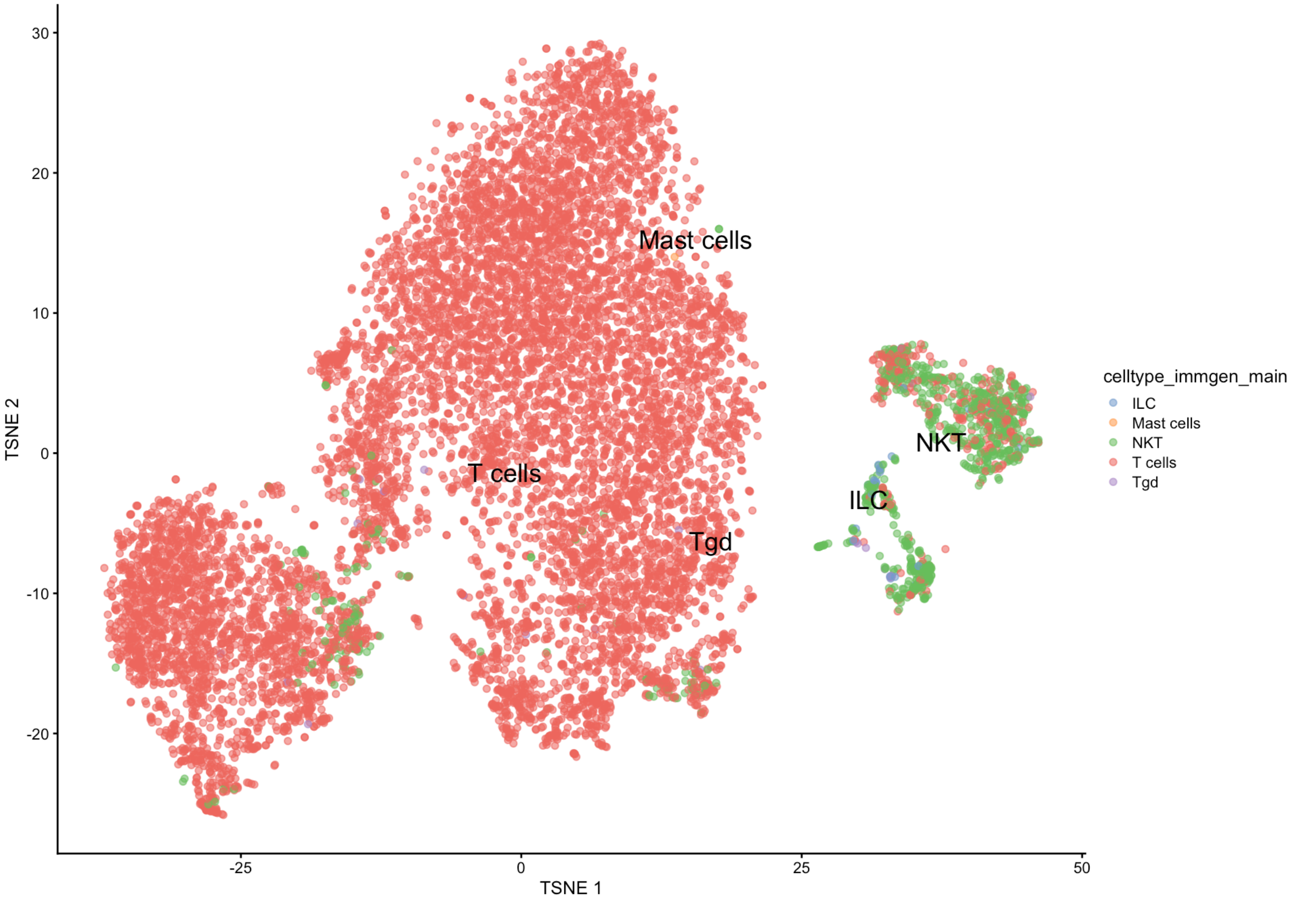


HIDE

```
plotUMAP(sce_merged,
         colour_by = "celltype_immgen_main",
         text_by = "celltype_immgen_main")
```

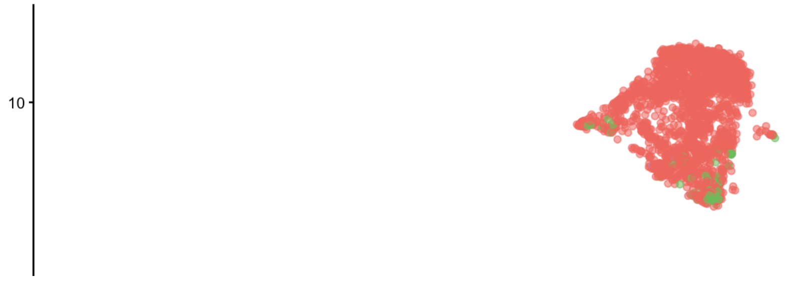

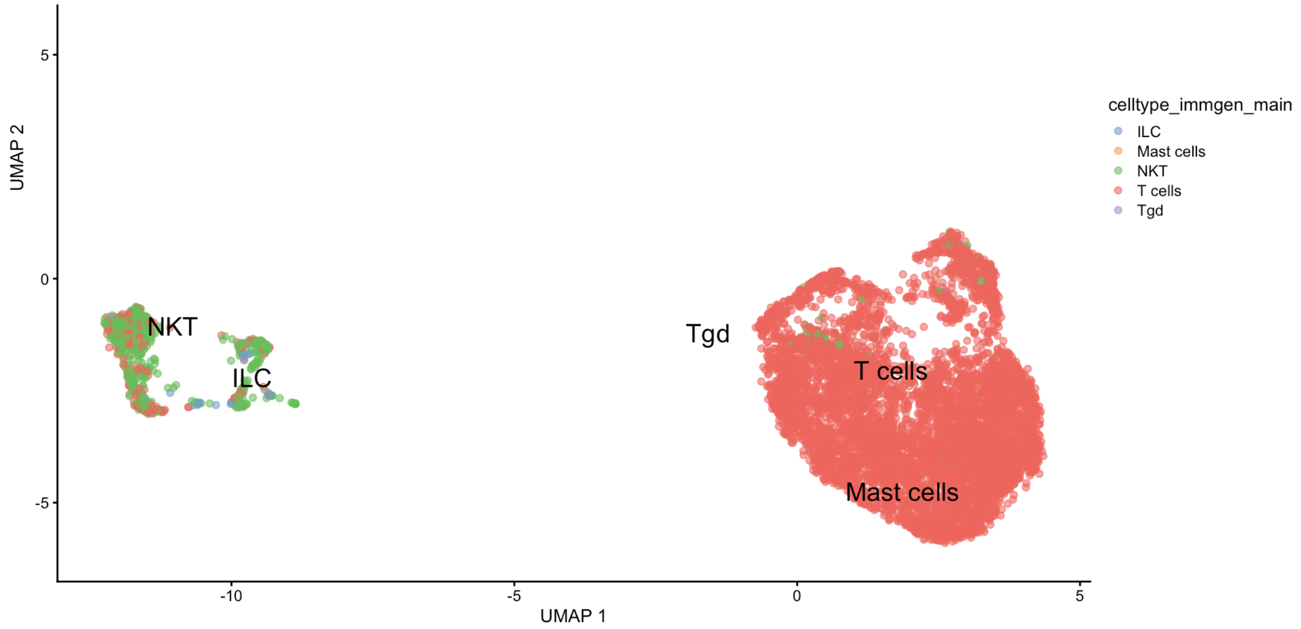


HIDE

```
# plot UMAP and tSNE representation colored by the assigned cell types from the
# fine labels
plotTSNE(sce_merged,
         colour_by = "celltype_immgen_fine",
         text_by = "celltype_immgen_fine")
```

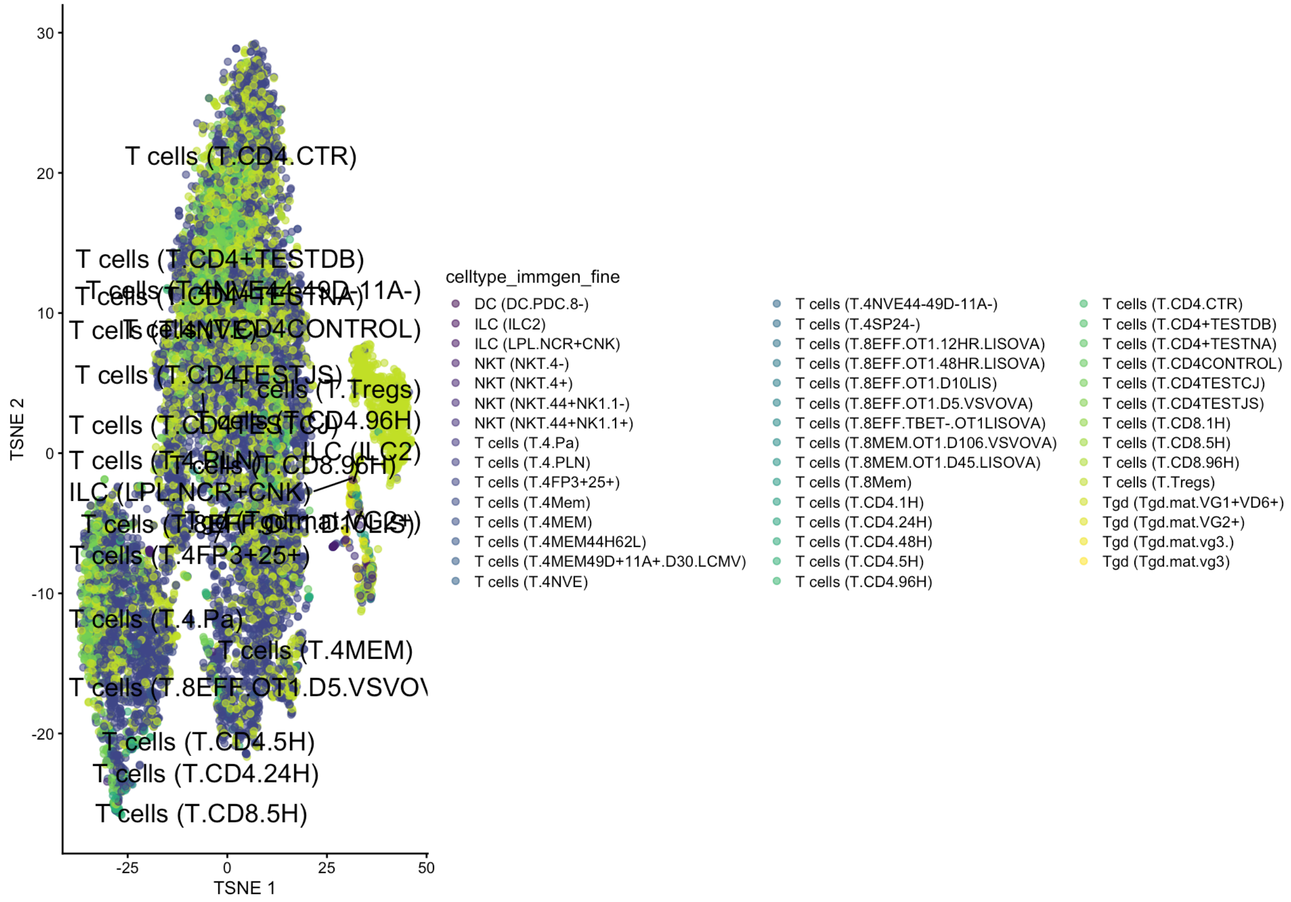

HIDE

```
plotUMAP(sce_merged,
         colour_by = "celltype_immgen_fine",
         text_by = "celltype_immgen_fine")
```

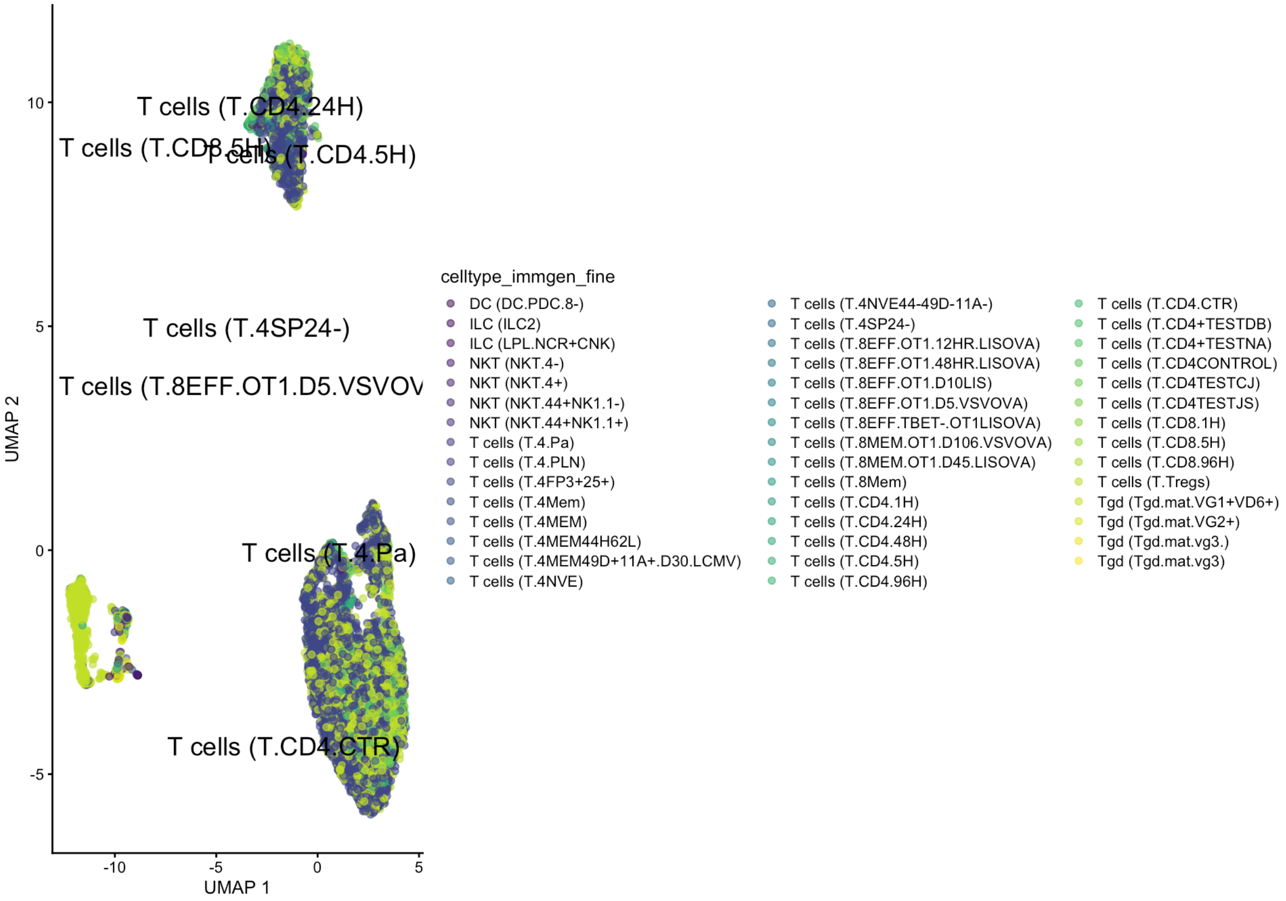


HIDE

```
# plot a heatmap of the degree of matching of the individual cells to the
# available cell type labels in the reference data
plotScoreHeatmap(celltype_immgen_main)
```

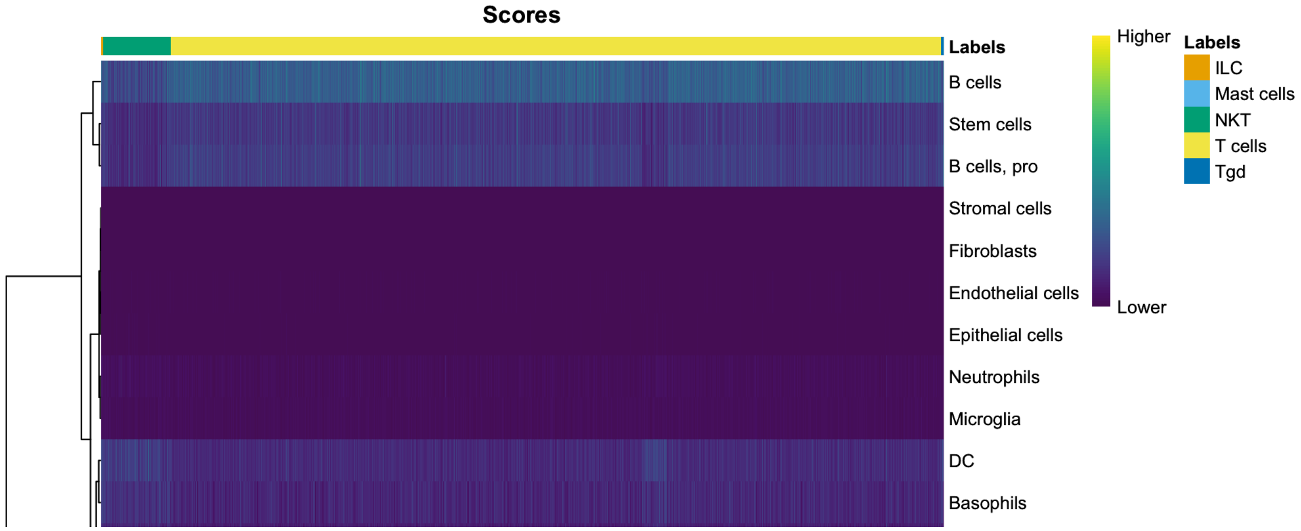

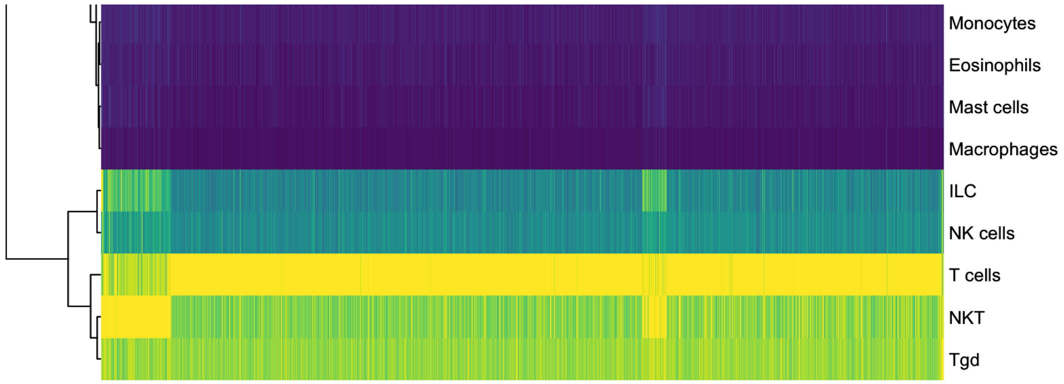


HIDE

```
# plot a heatmap of cluster to cell types, showing which cell type can found in
# the individual clusters
tab <- table(Assigned = celltype_immgen_main$pruned.labels,
             Cluster = colLabels(sce_merged))
# Adding a pseudo-count of 10 to avoid strong color jumps with just 1 cell.
pheatmap(log2(tab + 10),
         color = colorRampPalette(c("white", "darkblue"))(101))
```

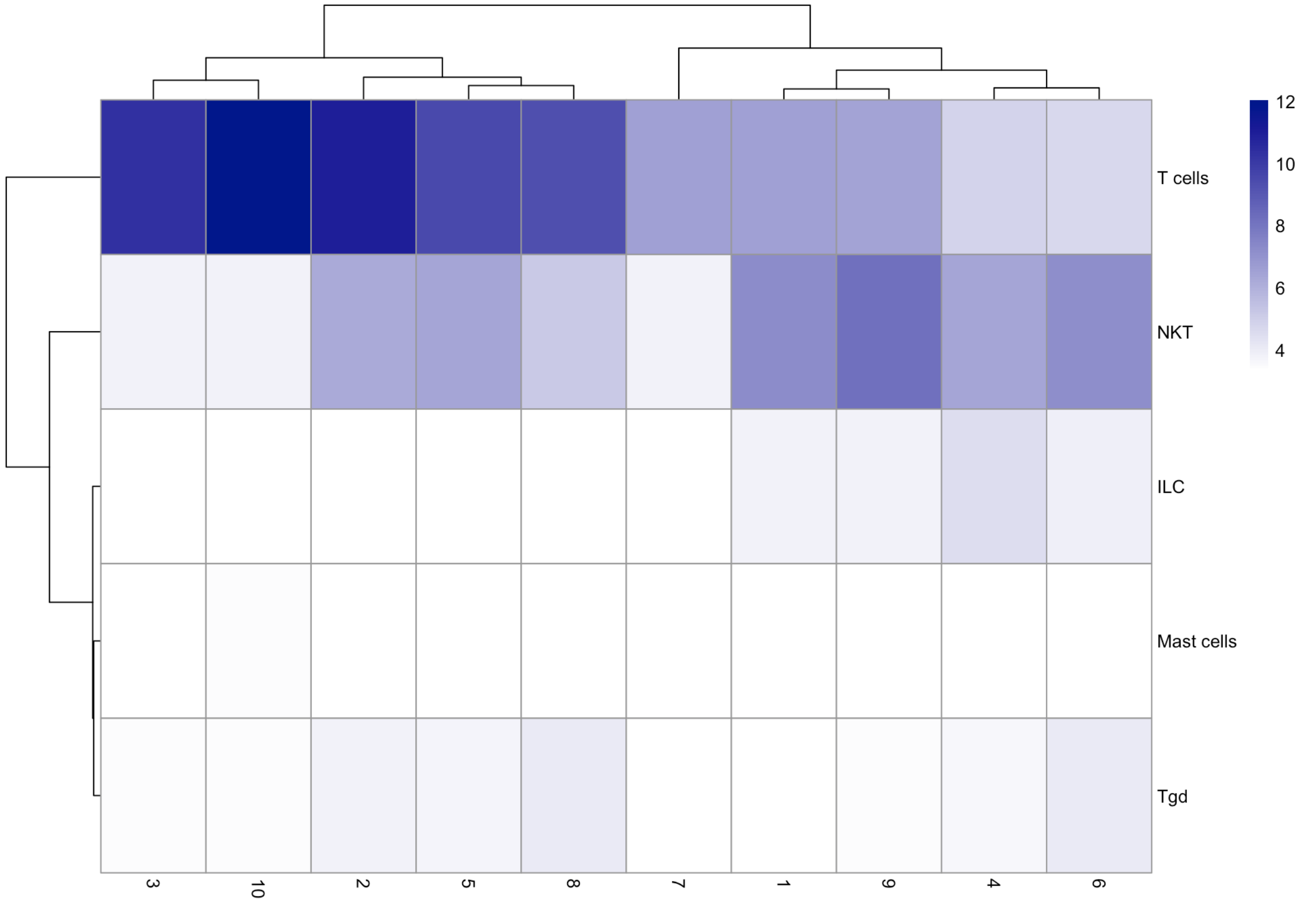


## 13.1.2 Mouse Altlas

HIDE

```
celltype_mouse_main <- SingleR(test = sce_merged,
                               ref = ref_annot_mouse,
                               labels = ref_annot_mouse$label.main,
                               BPPARAM = BiocParallel::MulticoreParam(6))
celltype_mouse_fine <- SingleR(test = sce_merged,
                               ref = ref_annot_mouse,
                               labels = ref_annot_mouse$label.fine,
                               BPPARAM = BiocParallel::MulticoreParam(6))
```

HIDE

```
table(celltype_mouse_main$labels)
```

```
##
## NK cells  T cells
##        8    10119
```

HIDE

```
table(celltype_mouse_fine$labels)
```

```
##
## NK cells  T cells
##       10    10117
```

HIDE

```
sce_merged$celltype_mouse_main <- celltype_mouse_main$labels
sce_merged$celltype_mouse_fine <- celltype_mouse_fine$labels
```

HIDE

```
plotTSNE(sce_merged,
         colour_by = "celltype_mouse_main",
         text_by = "celltype_mouse_main")
```

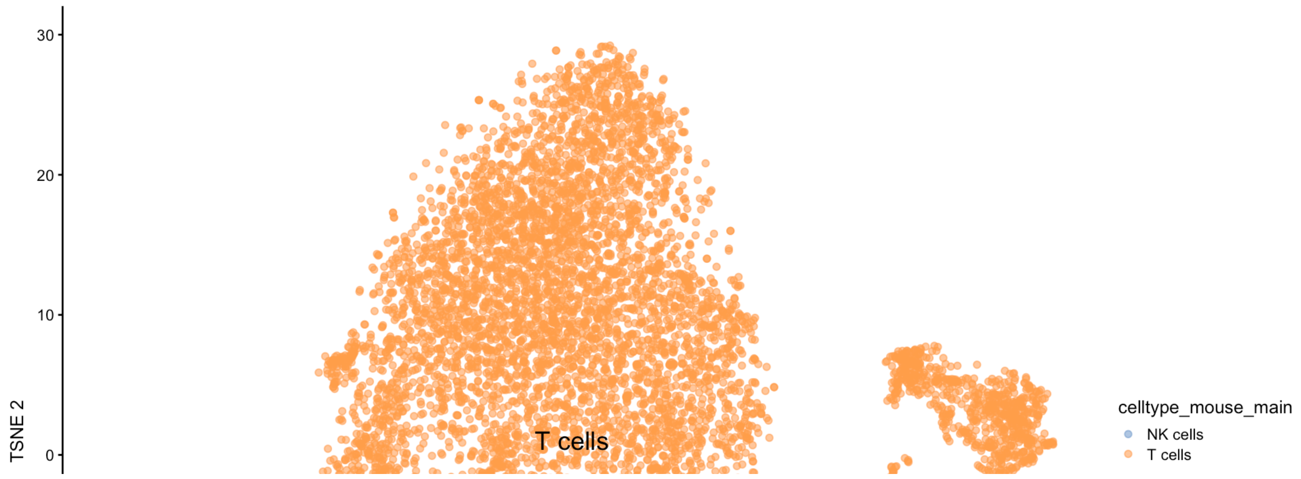

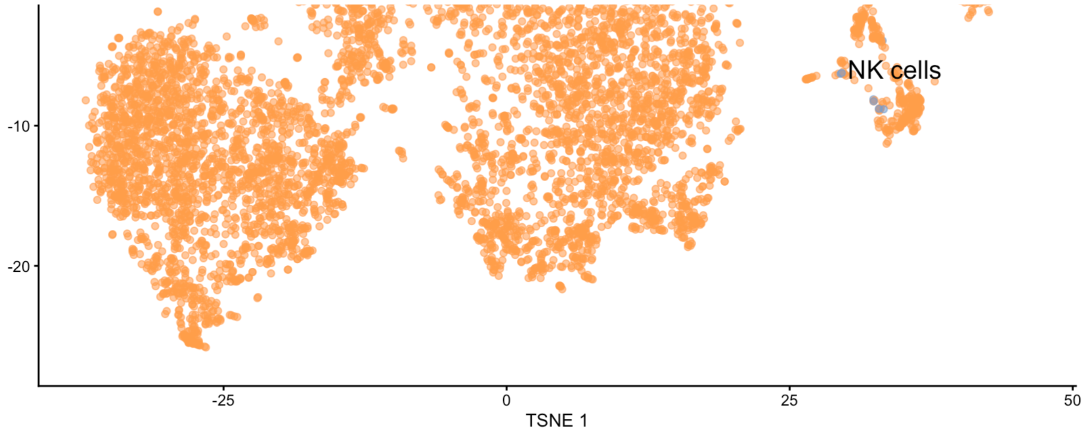


HIDE

```
plotUMAP(sce_merged,
         colour_by = "celltype_mouse_main",
         text_by = "celltype_mouse_main")
```

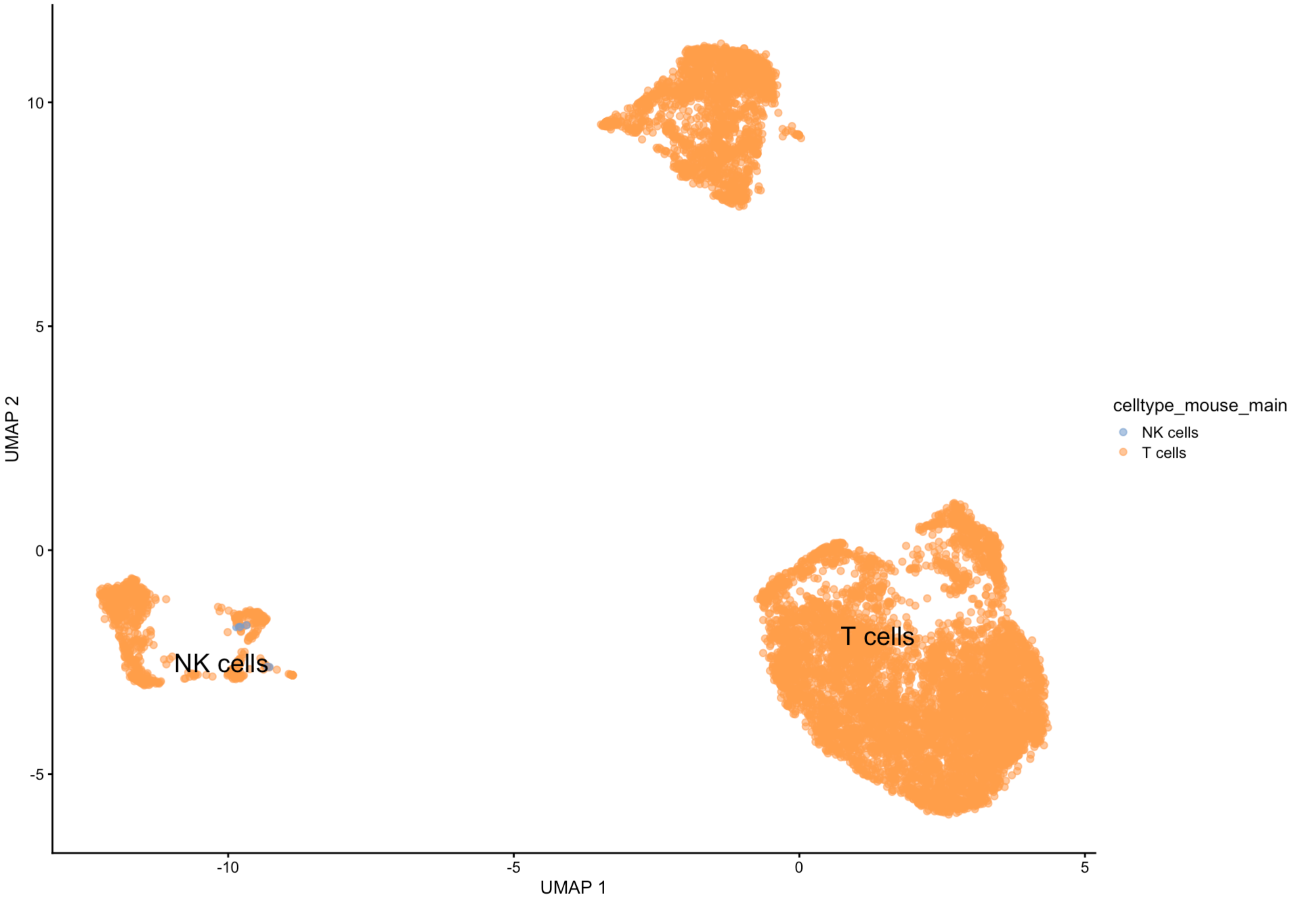


HIDE

```
plotTSNE(sce_merged,
         colour_by = "celltype_mouse_fine",
         text_by = "celltype_mouse_fine")
```

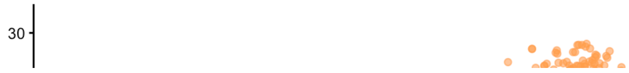

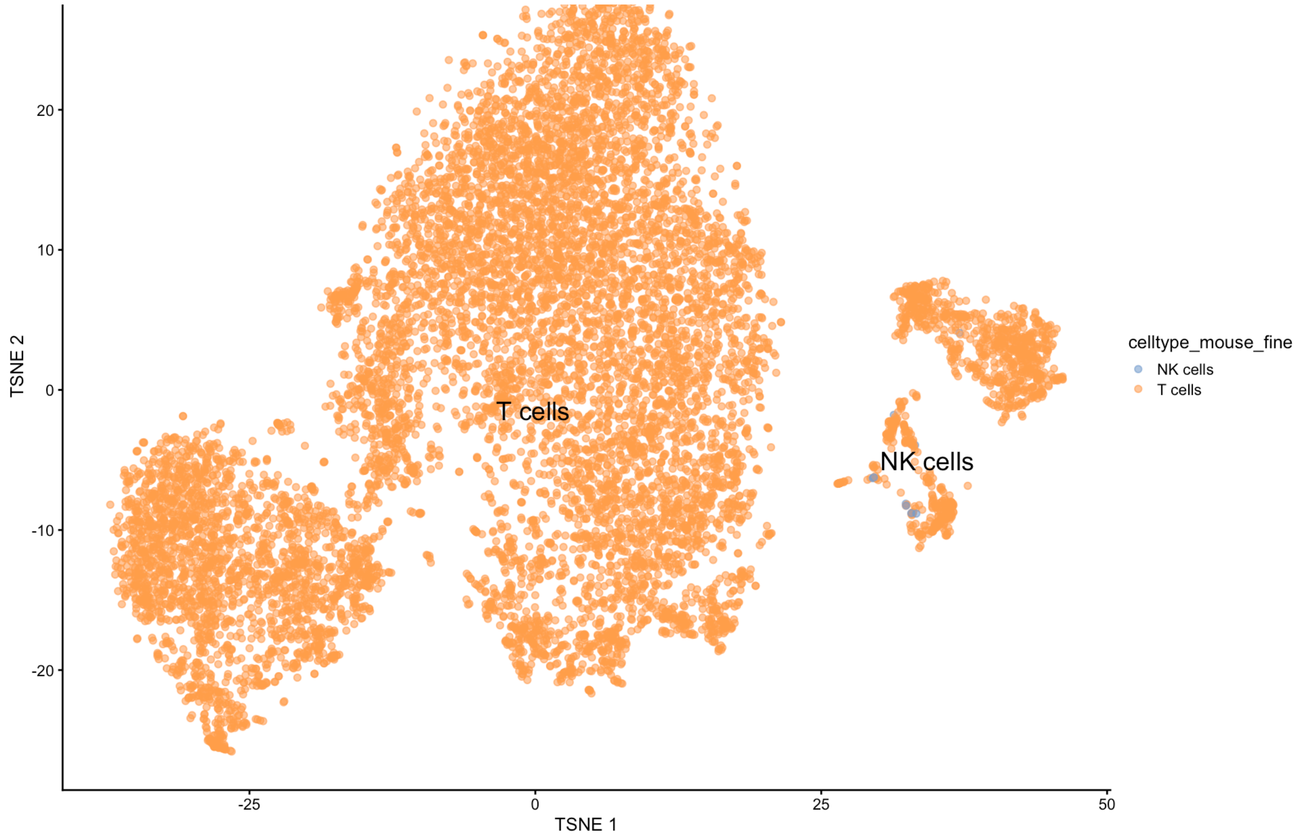


HIDE

```
plotUMAP(sce_merged,
         colour_by = "celltype_mouse_fine",
         text_by = "celltype_mouse_fine")
```

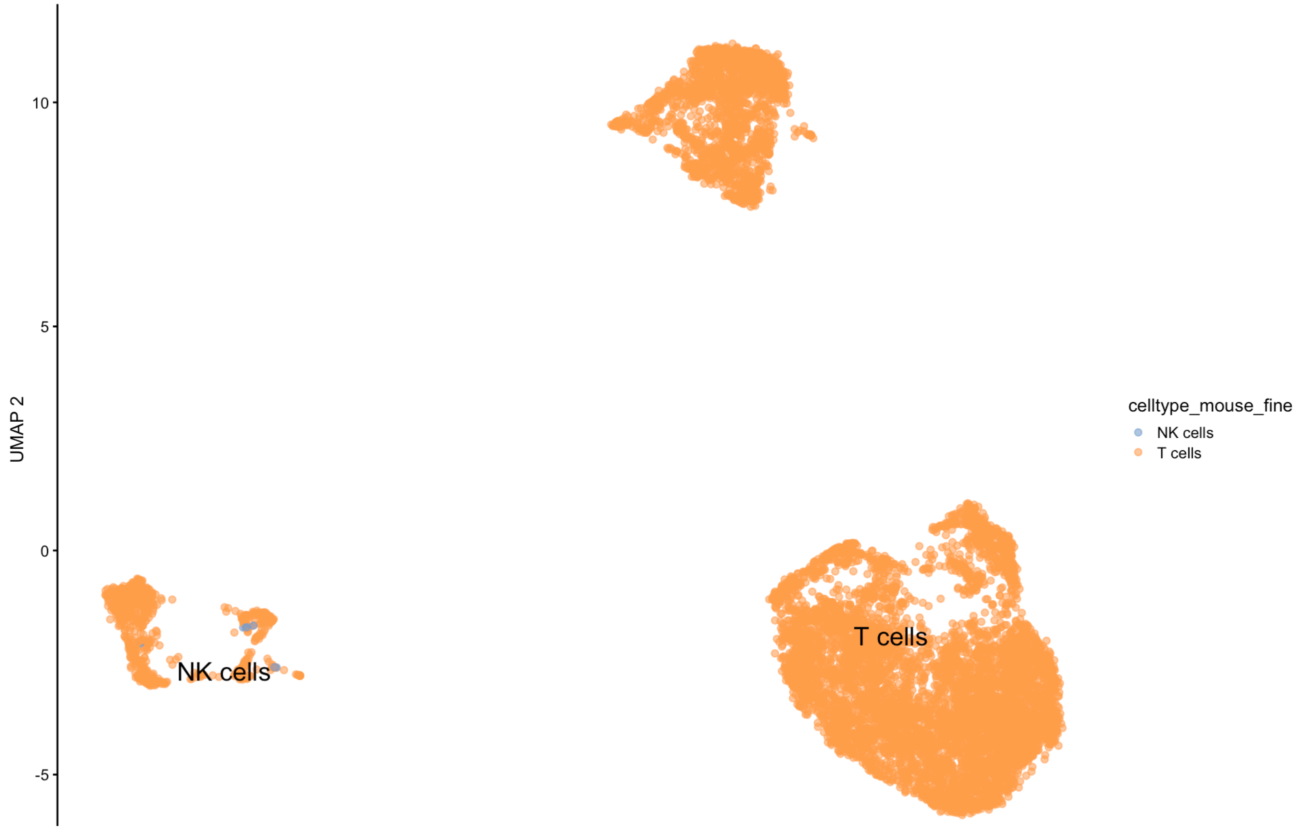

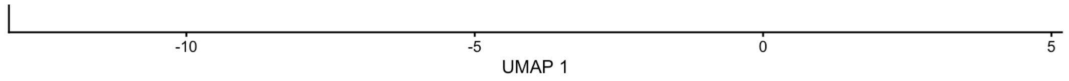


HIDE

```
plotScoreHeatmap(celltype_mouse_main)
```

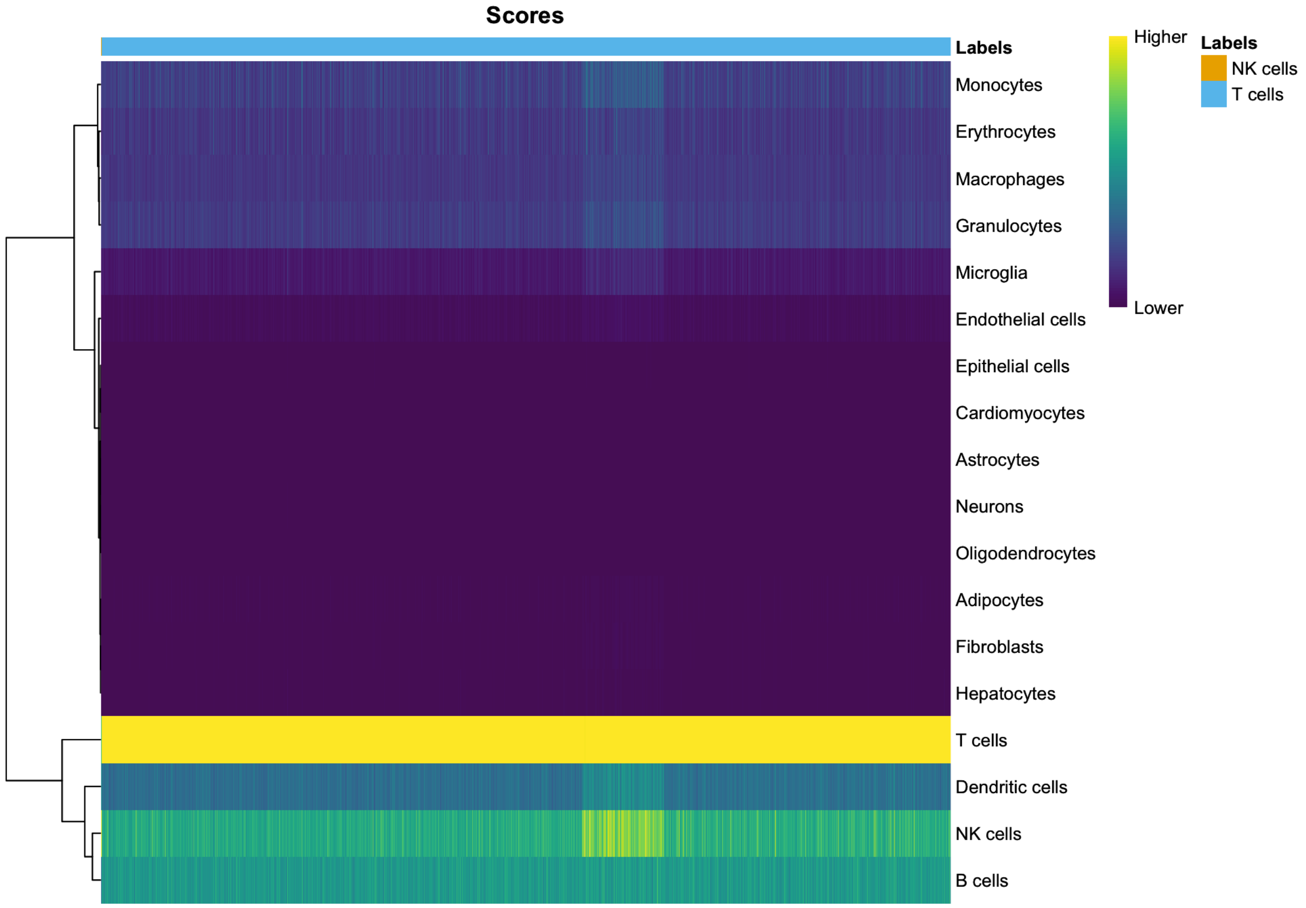


HIDE

```
tab <- table(Assigned = celltype_mouse_main$pruned.labels,
             Cluster = colLabels(sce_merged))
pheatmap(log2(tab + 10),
         color = colorRampPalette(c("white", "darkblue"))(101))
```

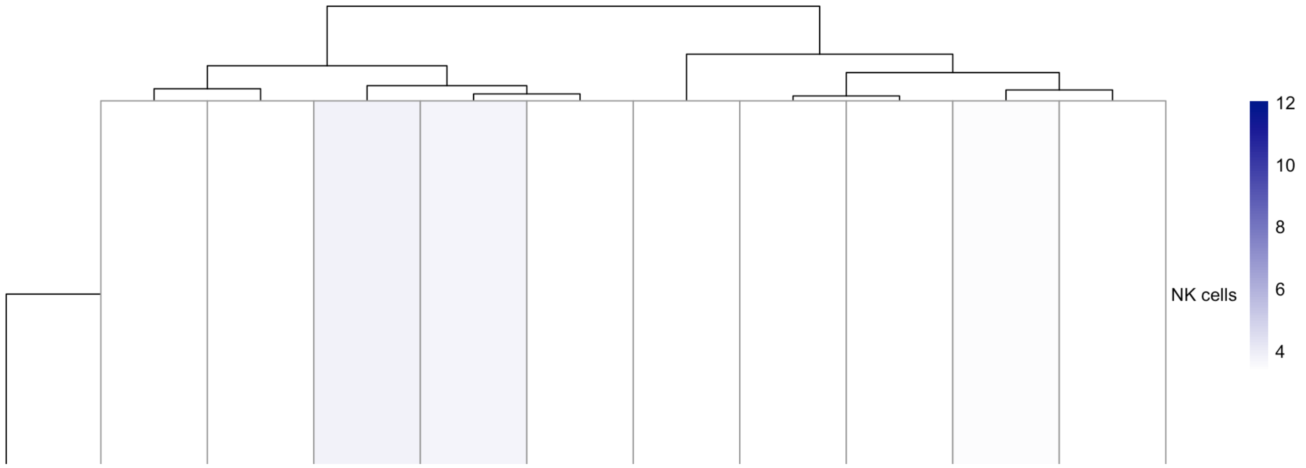

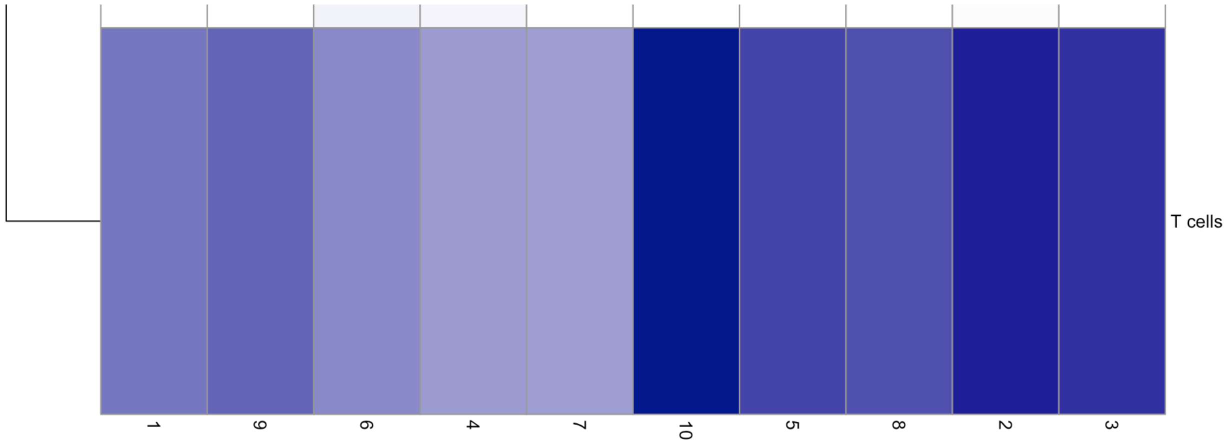


# 13.2 Use of Custom Markers

In the second approach, we use a selected list of known marker genes and a selected list of cell types for the cell type annotation.

For each of the used marker genes, we plot the expression of the genes in the different clusters as well as the UMAP presentation of the data colored by the expression of the respective marker genes used.

Additionally to the cell type annotation done in the first step, this approach can support in the interpretation of the data and the assignment of cell types to clusters.

HIDE

```
# set up a list of known marker genes for certain cell types
# T regulatory cells (Treg)
treg <- c("Foxp3", "Il2")
#
p_treg <- c("Rorc", "Gata3")
#
t_treg <- c("Ikzf2")
# Tissue Treg
tissue_treg <- c("Batf", "Klrg1", "Areg", "Ccr8", "Il10")
# Th1 cells
th1 <- c("Tbx21", "Ifng")
# Naive T-cells
naive <- c("Ccr7", "Sell", "Irf4")


# How to plot some of the markers.
# You can also select any other marker from the above list, by exchanging the
# gene name.
plotExpression(sce_merged, features = "Foxp3",
    x = "label", colour_by = "label")
```

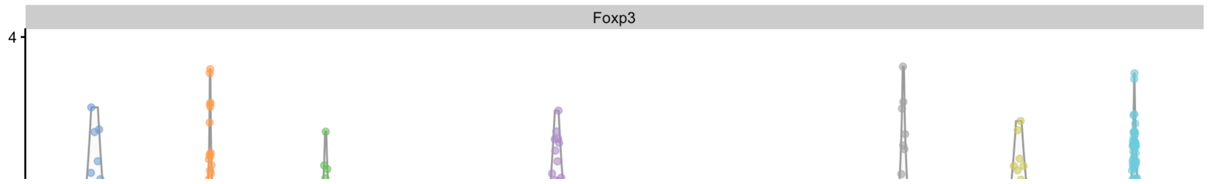

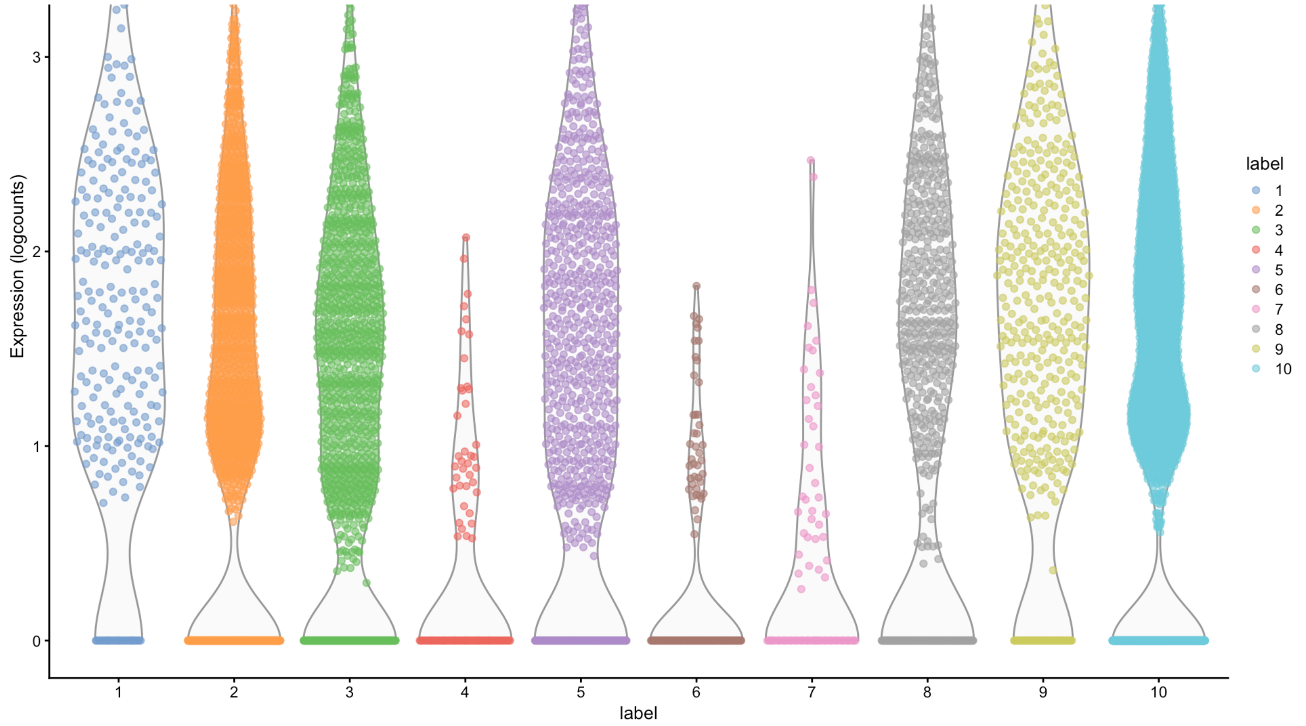


HIDE

```
plotUMAP(sce_merged, color_by = "Foxp3", order_by = "Foxp3")
```

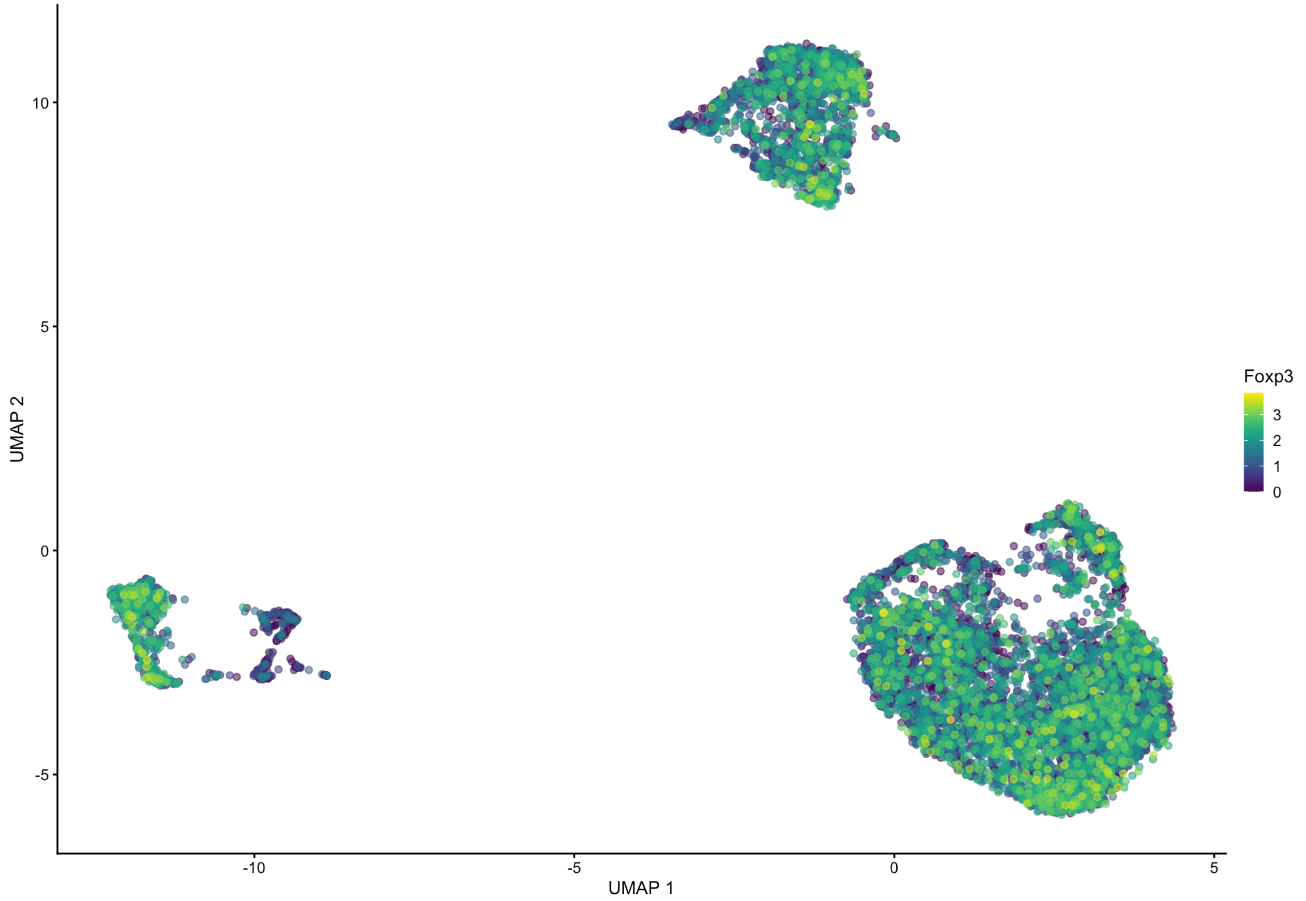


HIDE

```
plotExpression(sce_merged, features = "Klrg1",
    x = "label", colour_by = "label")
```

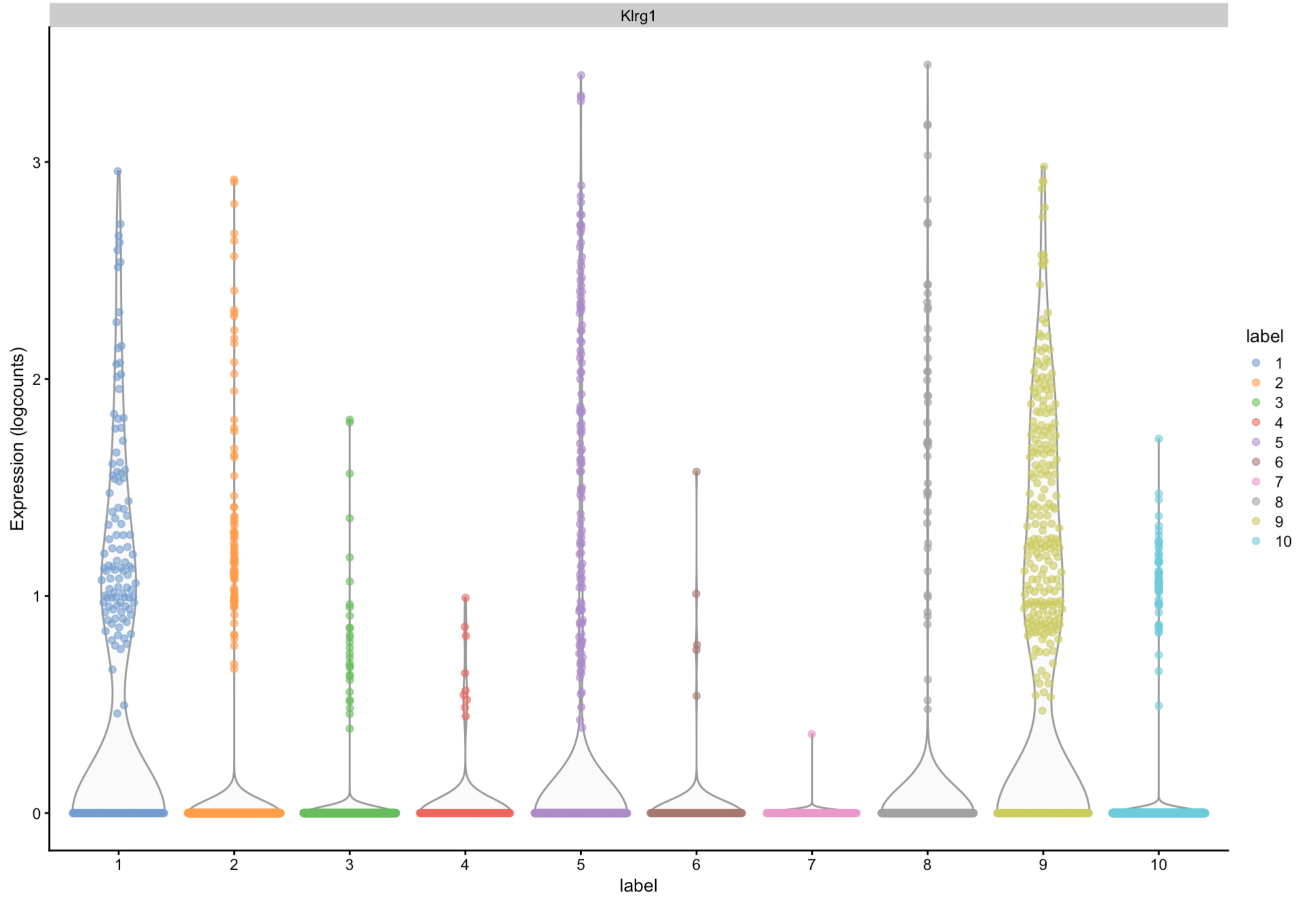


HIDE

```
plotUMAP(sce_merged, colour_by = "Klrg1", order_by = "Klrg1")
```

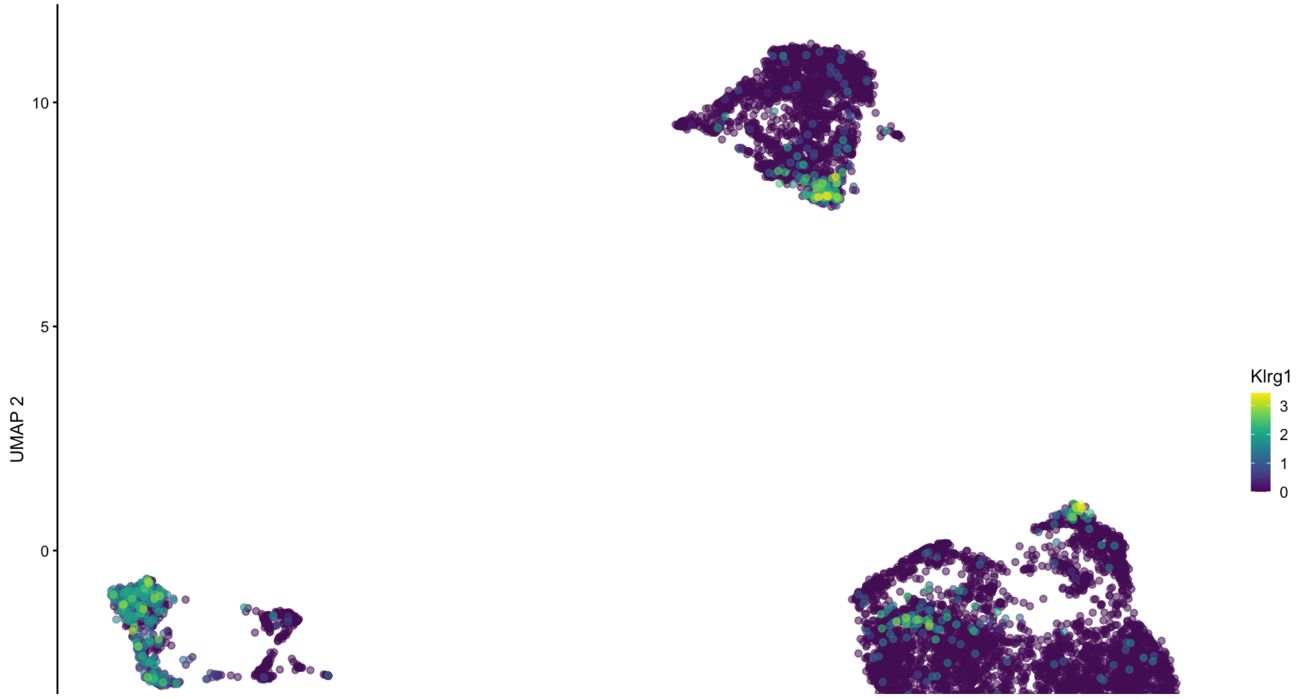

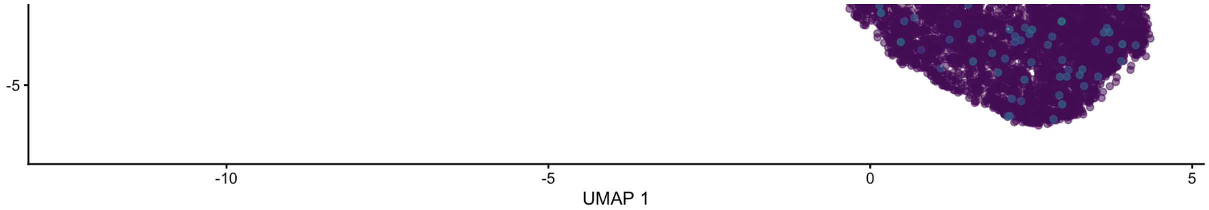


HIDE

```
plotExpression(sce_merged, features = "Sell",
    x = "label", colour_by = "label")
```

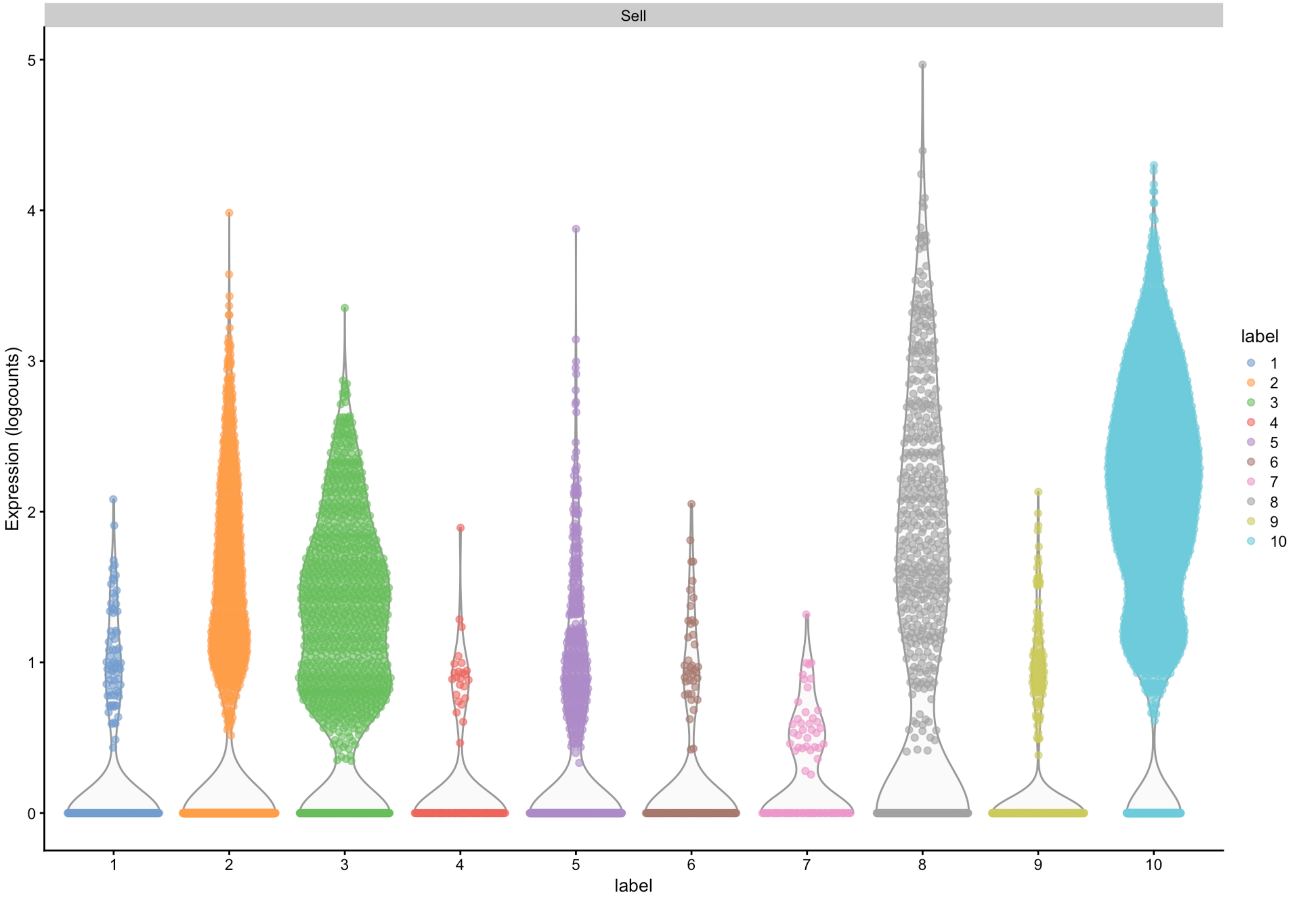


HIDE

```
plotUMAP(sce_merged, colour_by = "Sell", order_by = "Sell")
```

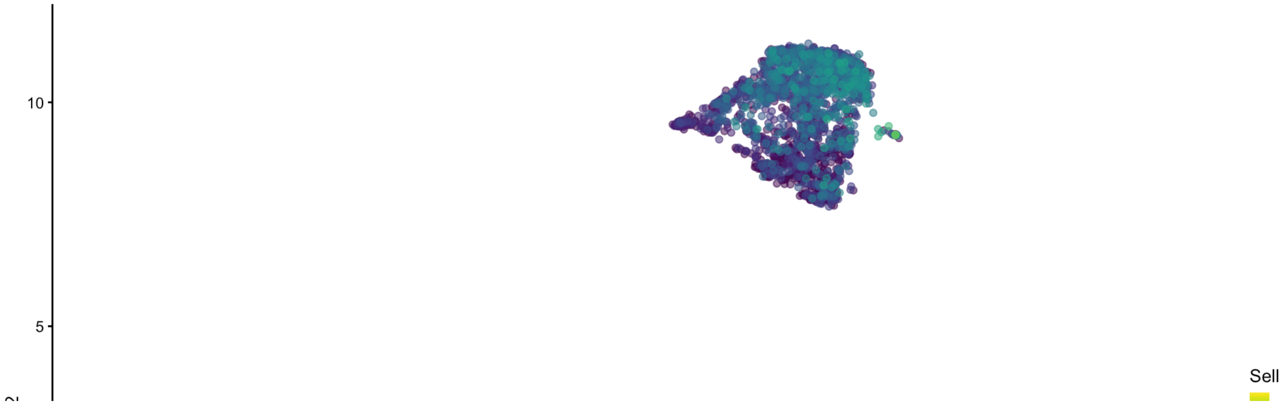

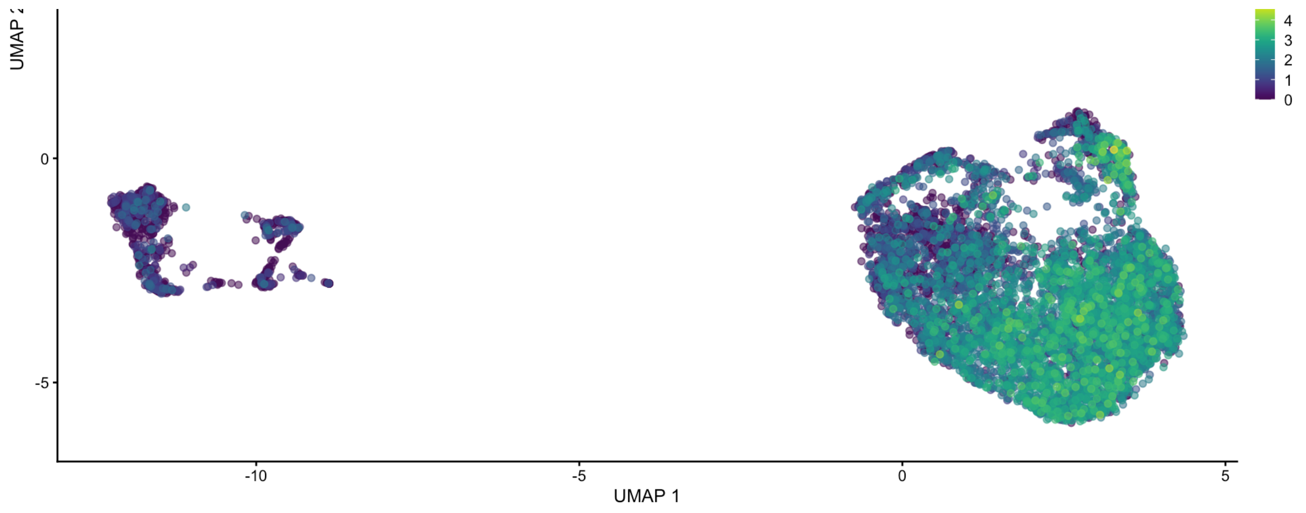


## 13.3 Seurat

For the cell type annotation, we also use the `SingleR` package in our `Seurat` pipeline. Hence we transform the Seurat object to a `SingleCellExperiment` and then run the code shown above for the cell type annotation.

HIDE

```
sce_merged <- as.SingleCellExperiment(seurat_merged)
```

# 14 Trajectory Analysis

A large variety of biological processes can be represented as a continuum of biological changes in the cellular state. This is especially true of cell type differentiation which can for example be observed in different T-cell subpopulations. In our high dimensional scRNA-seq data, we want to characterize this process of differentiation by finding a trajectory. Associated with a trajectory is the pseudotime, which is the position of each cell along the trajectory and could for example represent the state of differentiation of a cell along a continuous process. Pseudotime helps us answer questions about the global population structure of our data.

In our workflow, we use a cluster-based approach for identifying the trajectory in the data. The TSCAN algorithm implemented in the corresponding package first computes cluster centroids of the calculated clusters before forming a minimum spanning tree (MST).

The MST is an acyclic, undirected graph which passes through each cluster centroid exactly once and hence captures the transition between clusters. This is the trajectory of our data. The pseudotime can be calculated by simply projecting the single cells onto the MST. The pseudotime of a cell is the distance from the root of the MST to the position of the cell.

HIDE

```
by.cluster <- aggregateAcrossCells(sce_merged,
                                   ids = colLabels(sce_merged))
centroids <- reducedDim(by.cluster, "PCA")

# Set clusters = NULL as we have already aggregated above.
mst <- createClusterMST(centroids, clusters = NULL)
mst
```

```
## IGRAPH 54da3d0 UNW- 10 9 --
## + attr: name (v/c), coordinates (v/x), weight (e/n), gain (e/n)
## + edges from 54da3d0 (vertex names):
## [1] 1--4  1--9  2--8  2--10 3--5  3--7  5--6  5--8  6--9
```

HIDE

```
line.data <- reportEdges(by.cluster,
                         mst = mst,
                         clusters = NULL,
                         use.dimred="UMAP")

plotUMAP(sce_merged, colour_by = "label") +
    geom_line(data = line.data,
              mapping = aes(x = dim1,
                            y = dim2,
                            group = edge))
```

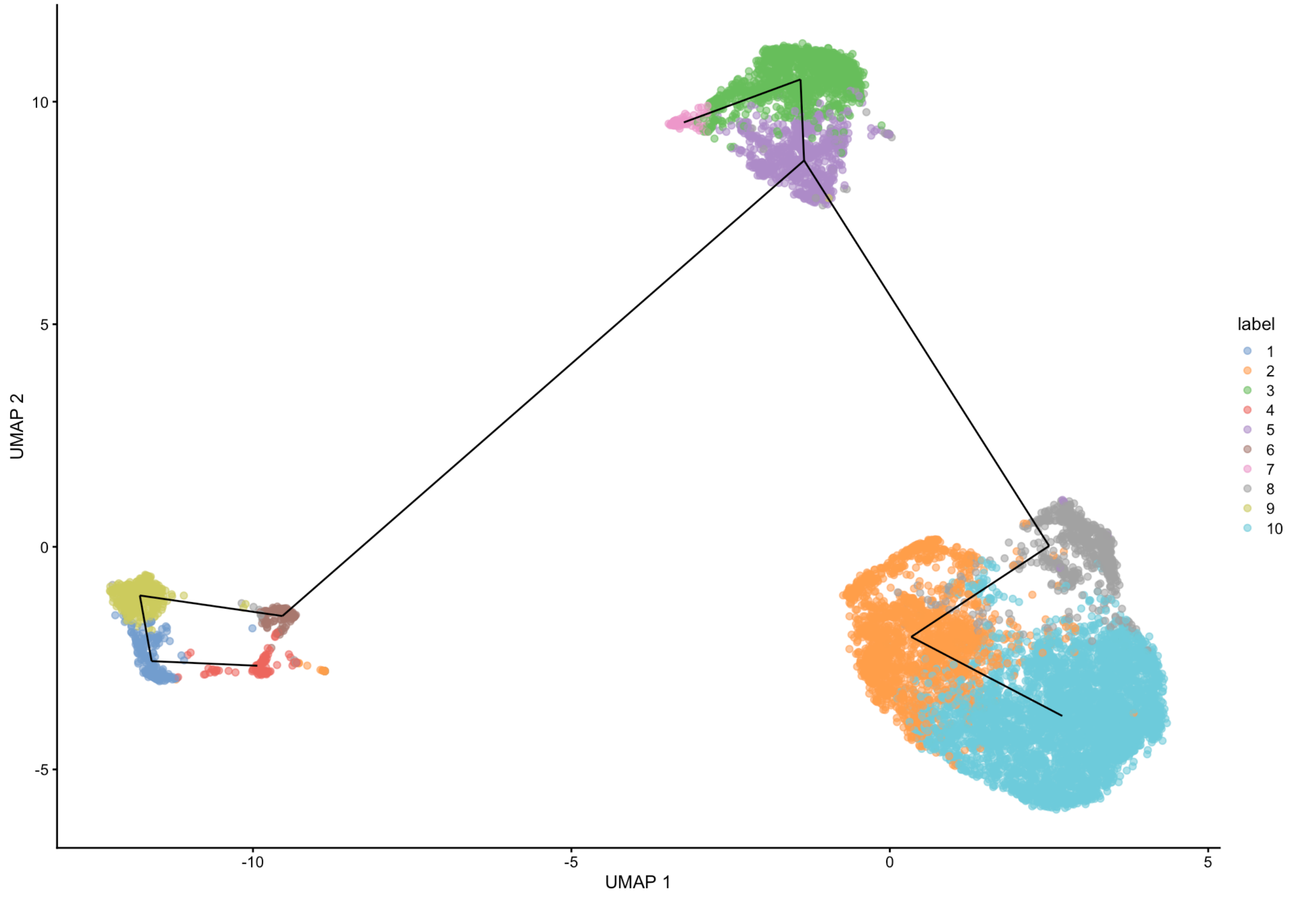

HIDE

```
map.tscan <- mapCellsToEdges(sce_merged,
                             mst = mst,
                             use.dimred = "PCA")
tscan.pseudo <- orderCells(map.tscan, mst)
head(tscan.pseudo)
```

```
## class: PseudotimeOrdering
## dim: 6 2
## metadata(1): start
## pathStats(1): ''
## cellnames(6): AAACCTGAGCGAAGGG-1 AAACCTGAGGGTTTCT-1 ...
##   AAACCTGTCTTACCTA-1 AAACGGGAGGGAAACA-1
## cellData names(4): left.cluster right.cluster left.distance
##   right.distance
## pathnames(2): 7 10
## pathData names(0):
```

HIDE

```
common.pseudo <- averagePseudotime(tscan.pseudo)

plotUMAP(sce_merged, colour_by = I(common.pseudo),
        text_by = "label", text_colour = "red") +
    geom_line(data = line.data, mapping = aes(x = dim1, y = dim2, group = edge))
```

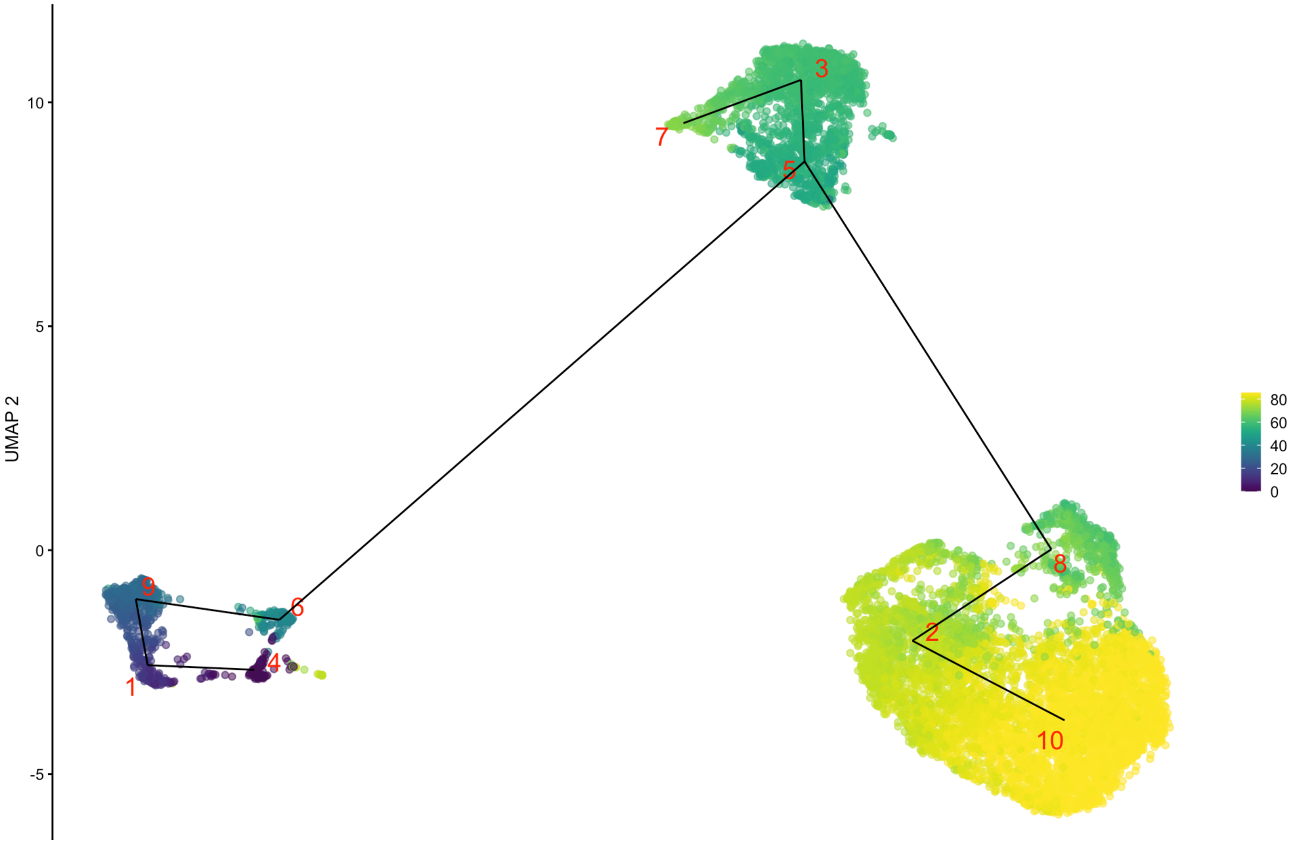

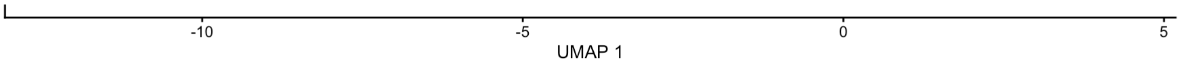


## 14.1 Seurat

For the trajectory analysis as well we use the same pipeline as presented above. If not done so yet, please return back to the cell annotation step and transform your `Seurat` object to a `SingleCellExperiment` object.

# 15 Interactive data exploration using iSEE

In many data analyses, especially in scRNA-seq, data visualization and exploration are crucial steps of the analysis which are usually reiterated and refined throughout the process. We also have mentioned in the text of this workflow that we reiterated and refined results from analyses steps using the output of a different part.

An excellent tool to support this repeating reiteration and refinement of data exploration and visualization is `iSEE`. `iSEE` is an R-package which implements an Shiny app for interactive and reproducible exploration and visualization of scRNA-seq data. On top of that it is easy to use and a rather self-learning experience supported by interactive tours in the app to guide new users.

`iSEE` can be easily incorporated in an scRNA-seq analyses workflow, with as little code as `iSEE(sce)` (with sce being a `SingleCellExperiment` object of the data).

The default layout of iSEE when launched usually contains eight panels, each highlighting and visualizing a particular aspect of the data. The layout of the panels can be changed and adapted as described later. Different panels can be added and removed from the layout to focus on different aspects of the scRNA-seq data. For users who are unfamiliar with the iSEE interface, the application provides interactive tours explaining the different options and possibilities provided. The tour can be accessed via the button with the question mark icon in the upper right corner.

In our workflow, we present a custom set-up of iSEE where we preselected and arranged the individual panels of the app compared to the default version. However, both versions are equally powerful in supporting the analysis of the data.

HIDE

```
# Copy the original object in order to not change something there
sce_copy <- sce_merged
# Set up the data for the individual panels
sce_copy$log10_total_counts <- log10(sce_copy$total)
sce_copy$total_counts_rank <- rank(-sce_copy$total)

# find marker genes
markers.up <- findMarkers(sce_copy, direction = "up",
    log.p = TRUE, sorted = FALSE)

# Collate the log-p-value for each marker in a single table
all.p <- lapply(markers.up, FUN = "[[", i = "log.p.value")
all.p <- DataFrame(all.p, check.names = FALSE)
colnames(all.p) <- paste0("cluster", colnames(all.p))

# Store the table of results as row metadata
rowData(sce_copy) <- cbind(rowData(sce_copy), all.p)

dec <- modelGeneVarByPoisson(sce_copy)
rowData(sce_copy) <- cbind(rowData(sce_copy), dec)

# set up the state of the app
initial.state <- list(
    # Configure a "Column data plot" panel
    ColumnDataPlot(YAxis = "log10_total_counts",
        XAxis = "Column data",
        XAxisColumnData = "total_counts_rank",
        DataBoxOpen = TRUE,
        PanelId = 1L),

    # Configure a "Reduced dimension plot " panel
    ReducedDimensionPlot(
        Type = "TSNE",
        VisualBoxOpen = TRUE,
        DataBoxOpen = TRUE,
        ColorBy = "Column data",
        ColorByColumnData = "log10_total_counts",
        SelectionBoxOpen = TRUE,
        ColumnSelectionSource = "ColumnDataPlot1"),

    RowDataTable(PanelId=1L),

    # Configure a "Feature assay plot" panel
    FeatureAssayPlot(
        YAxisFeatureSource = "RowDataTable1",
        XAxis = "Column data",
        XAxisColumnData = "label",
        Assay = "logcounts",
        DataBoxOpen = TRUE
    ),
```

```
    # Configure a "Reduced dimension plot" panel
    ReducedDimensionPlot(
        Type = "UMAP",
        ColorBy = "Feature name",
        ColorByFeatureSource = "RowDataTable1",
        ColorByFeatureNameAssay = "logcounts"
    ),

    RowDataPlot(
        YAxis = "total",
        XAxis = "Row data",
        XAxisRowData = "mean",
        PanelId = 1L
    ),

    RowDataTable(
        RowSelectionSource = "RowDataPlot1"
    ),

    # Configure a "ComplexHeatmap" panel
    ComplexHeatmapPlot(
        RowSelectionSource = "RowDataPlot1",
        CustomRows = FALSE,
        ColumnData = "label",
        Assay = "logcounts",
        ClusterRows = TRUE,
        PanelHeight = 800L,
        AssayCenterRows = TRUE
    )

)

# Prepare the app
app <- iSEE(sce_copy, initial = initial.state)

# You can start the app by uncommenting the following line
#app
```

# Session info

HIDE

```
sessionInfo()
```

```
## R version 4.2.0 (2022-04-22)
## Platform: x86_64-apple-darwin17.0 (64-bit)
## Running under: macOS Big Sur/Monterey 10.16
##
## Matrix products: default
## BLAS:   /Library/Frameworks/R.framework/Versions/4.2/Resources/lib/libRblas.
0.dylib
## LAPACK: /Library/Frameworks/R.framework/Versions/4.2/Resources/lib/libRlapac
k.dylib
##
## Random number generation:
##  RNG:     L'Ecuyer-CMRG
##  Normal:  Inversion
##  Sample:  Rejection
##
## locale:
## [1] en_US.UTF-8/en_US.UTF-8/en_US.UTF-8/C/en_US.UTF-8/en_US.UTF-8
##
## attached base packages:
## [1] stats4    stats     graphics  grDevices utils     datasets  methods
## [8] base
##
## other attached packages:
##  [1] celldex_1.8.0               ensembldb_2.22.0
##  [3] AnnotationFilter_1.22.0     GenomicFeatures_1.50.4
##  [5] AnnotationDbi_1.60.2        harmony_0.1.1
##  [7] Rcpp_1.0.11                 RColorBrewer_1.1-3
##  [9] TSCAN_1.36.0                TrajectoryUtils_1.6.0
## [11] igraph_1.5.0.1              pheatmap_1.0.12
## [13] iSEE_2.10.0                 batchelor_1.14.1
## [15] BiocSingular_1.14.0         scran_1.26.2
## [17] scDblFinder_1.12.0          AnnotationHub_3.6.0
## [19] BiocFileCache_2.6.1         dbplyr_2.3.3
## [21] DropletUtils_1.18.1         DT_0.28
## [23] scater_1.26.1               ggplot2_3.4.2
## [25] scuttle_1.8.4               dplyr_1.1.2
## [27] SingleR_2.0.0               stringr_1.5.0
## [29] SingleCellExperiment_1.20.1 SummarizedExperiment_1.28.0
## [31] Biobase_2.58.0              GenomicRanges_1.50.2
## [33] GenomeInfoDb_1.34.9         IRanges_2.32.0
## [35] S4Vectors_0.36.2            BiocGenerics_0.44.0
## [37] MatrixGenerics_1.10.0       matrixStats_1.0.0
## [39] knitr_1.43
##
## loaded via a namespace (and not attached):
##   [1] utf8_1.2.3                    shinydashboard_0.7.2
##   [3] R.utils_2.12.2                tidyselect_1.2.0
##   [5] RSQLite_2.3.1                 htmlwidgets_1.6.2
##   [7] grid_4.2.0                    combinat_0.0-8
##   [9] BiocParallel_1.32.6           Rtsne_0.16
##  [11] munsell_0.5.0                 ScaledMatrix_1.6.0
```

```
##  [13] codetools_0.2-19              statmod_1.5.0
##  [15] xgboost_1.7.5.1               miniUI_0.1.1.1
##  [17] withr_2.5.0                   colorspace_2.1-0
##  [19] fastICA_1.2-3                 filelock_1.0.2
##  [21] highr_0.10                    rstudioapi_0.15.0
##  [23] shinyWidgets_0.7.6            labeling_0.4.2
##  [25] GenomeInfoDbData_1.2.9        farver_2.1.1
##  [27] bit64_4.0.5                   rhdf5_2.42.1
##  [29] vctrs_0.6.3                   generics_0.1.3
##  [31] xfun_0.39                     R6_2.5.1
##  [33] doParallel_1.0.17             ggbeeswarm_0.7.2
##  [35] clue_0.3-64                   rsvd_1.0.5
##  [37] locfit_1.5-9.8                bitops_1.0-7
##  [39] rhdf5filters_1.10.1           cachem_1.0.8
##  [41] shinyAce_0.4.2                DelayedArray_0.24.0
##  [43] promises_1.2.0.1              BiocIO_1.8.0
##  [45] scales_1.2.1                  beeswarm_0.4.0
##  [47] gtable_0.3.3                  beachmat_2.14.2
##  [49] rlang_1.1.1                   GlobalOptions_0.1.2
##  [51] splines_4.2.0                 lazyeval_0.2.2
##  [53] rtracklayer_1.58.0            BiocManager_1.30.21.1
##  [55] yaml_2.3.7                    crosstalk_1.2.0
##  [57] httpuv_1.6.11                 tools_4.2.0
##  [59] ellipsis_0.3.2                gplots_3.1.3
##  [61] jquerylib_0.1.4               plyr_1.8.8
##  [63] progress_1.2.2                sparseMatrixStats_1.10.0
##  [65] zlibbioc_1.44.0               purrr_1.0.1
##  [67] RCurl_1.98-1.12               prettyunits_1.1.1
##  [69] GetoptLong_1.0.5              viridis_0.6.4
##  [71] cowplot_1.1.1                 fontawesome_0.5.1
##  [73] ggrepel_0.9.3                 cluster_2.1.4
##  [75] magrittr_2.0.3                data.table_1.14.8
##  [77] ResidualMatrix_1.8.0          circlize_0.4.15
##  [79] colourpicker_1.2.0            ProtGenerics_1.30.0
##  [81] hms_1.1.3                     shinyjs_2.1.0
##  [83] mime_0.12                     evaluate_0.21
##  [85] xtable_1.8-4                  XML_3.99-0.14
##  [87] mclust_6.0.0                  gridExtra_2.3
##  [89] shape_1.4.6                   biomaRt_2.54.1
##  [91] compiler_4.2.0                tibble_3.2.1
##  [93] KernSmooth_2.23-22            crayon_1.5.2
##  [95] R.oo_1.25.0                   htmltools_0.5.5
##  [97] mgcv_1.9-0                    later_1.3.1
##  [99] DBI_1.1.3                     ExperimentHub_2.6.0
## [101] ComplexHeatmap_2.14.0         MASS_7.3-60
## [103] rappdirs_0.3.3                BiocStyle_2.26.0
## [105] Matrix_1.6-0                  cli_3.6.1
## [107] R.methodsS3_1.8.2             parallel_4.2.0
## [109] metapod_1.6.0                 pkgconfig_2.0.3
## [111] GenomicAlignments_1.34.1      xml2_1.3.5
## [113] foreach_1.5.2                 vipor_0.4.5
```

```
## [115] bslib_0.5.0                   dqrng_0.3.0
## [117] XVector_0.38.0                digest_0.6.33
## [119] RcppAnnoy_0.0.21              Biostrings_2.66.0
## [121] rmarkdown_2.23                rintrojs_0.3.2
## [123] uwot_0.1.16                   edgeR_3.40.2
## [125] DelayedMatrixStats_1.20.0     restfulr_0.0.15
## [127] curl_5.0.1                    shiny_1.7.4.1
## [129] Rsamtools_2.14.0              gtools_3.9.4
## [131] rjson_0.2.21                  lifecycle_1.0.3
## [133] nlme_3.1-162                  jsonlite_1.8.7
## [135] Rhdf5lib_1.20.0               BiocNeighbors_1.16.0
## [137] viridisLite_0.4.2             limma_3.54.2
## [139] fansi_1.0.4                   pillar_1.9.0
## [141] lattice_0.21-8                KEGGREST_1.38.0
## [143] fastmap_1.1.1                 httr_1.4.6
## [145] interactiveDisplayBase_1.36.0 glue_1.6.2
## [147] png_0.1-8                     iterators_1.0.14
## [149] bluster_1.8.0                 BiocVersion_3.16.0
## [151] bit_4.0.5                     stringi_1.7.12
## [153] sass_0.4.7                    HDF5Array_1.26.0
## [155] blob_1.2.4                    caTools_1.18.2
## [157] memoise_2.0.1                 irlba_2.3.5.1
```
